# Precisely patterned nanofibres made from extendable protein multiplexes

**DOI:** 10.1038/s41557-023-01314-x

**Published:** 2023-09-04

**Authors:** Neville P. Bethel, Andrew J. Borst, Fabio Parmeggiani, Matthew J. Bick, TJ Brunette, Hannah Nguyen, Alex Kang, Asim K. Bera, Lauren Carter, Marcos C. Miranda, Ryan D. Kibler, Mila Lamb, Xinting Li, Banumathi Sankaran, David Baker

**Affiliations:** 1 Department of Biochemistry, University of Washington, Seattle, WA USA; 2 Institute for Protein Design, University of Washington, Seattle, WA USA; 3 Howard Hughes Medical Institute, University of Washington, Seattle, WA USA; 4 School of Chemistry, University of Bristol, Bristol, UK; 5 School of Biochemistry, University of Bristol, Bristol, UK; 6 Bristol Biodesign Institute, University of Bristol, Bristol, UK; 7 Berkeley Center for Structural Biology, Molecular Biophysics and Integrated Bioimaging, Lawrence Berkeley Laboratory, Berkeley, CA USA

**Keywords:** Protein folding, Protein design, Nanoscale materials

## Abstract

Molecular systems with coincident cyclic and superhelical symmetry axes have considerable advantages for materials design as they can be readily lengthened or shortened by changing the length of the constituent monomers. Among proteins, alpha-helical coiled coils have such symmetric, extendable architectures, but are limited by the relatively fixed geometry and flexibility of the helical protomers. Here we describe a systematic approach to generating modular and rigid repeat protein oligomers with coincident *C*_2_ to *C*_8_ and superhelical symmetry axes that can be readily extended by repeat propagation. From these building blocks, we demonstrate that a wide range of unbounded fibres can be systematically designed by introducing hydrophilic surface patches that force staggering of the monomers; the geometry of such fibres can be precisely tuned by varying the number of repeat units in the monomer and the placement of the hydrophilic patches.

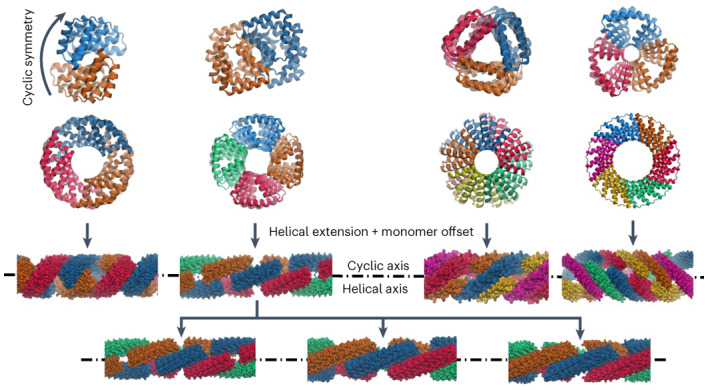

## Abstract (web summary)

Molecular systems with coincident cyclic and superhelical symmetry axes have considerable advantages for materials design as they can be lengthened or shortened by changing the length of the monomers. Now a systematic approach to generate modular repeat protein oligomers with combined symmetry that can be extended by repeat propagation has been developed.

## Main

**Fig. 1:**
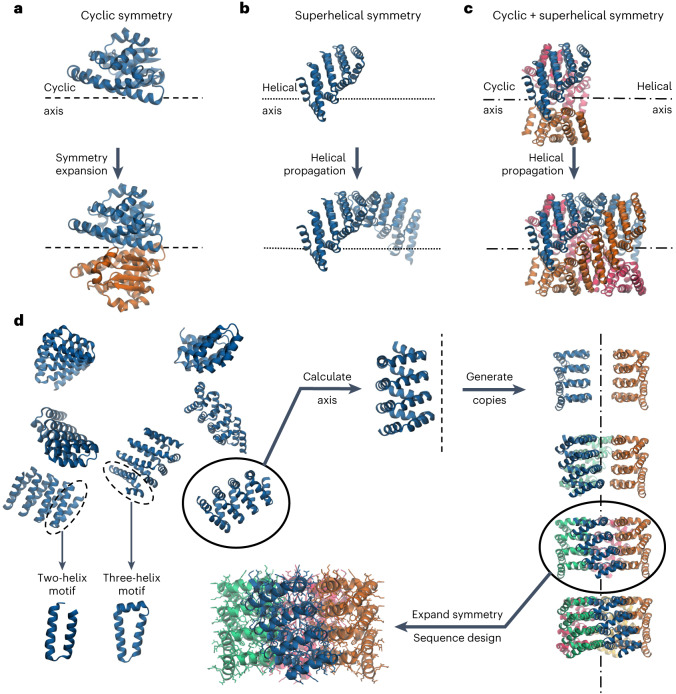
CHR concept and design approach. **a**–**c**, Examples of cyclic symmetry, superhelical symmetry and the combination of both cyclic and superhelical symmetry. **a**, Cyclic symmetry allows for copies of a monomeric chain to be propagated around a single axis. **b**, For helical propagation, asymmetric units can be stacked along an axis with a defined twist and spacing. **c**, Combining superhelical symmetry and cyclic symmetry allows for the indefinite extension along the helix axis while maintaining and extending the interface of the symmetric copies. **d**, Multiplexes are created by first generating de novo repeat protein monomers through backbone fragment assembly. A library of four-repeat monomers is generated, where a single repeat is consists of a two- or three-helix structural motif asymmetric. The monomer superhelical axis is calculated, and evenly spaced copies are generated around this axis. Different cyclic symmetries are attempted, and specific symmetries are selected according to contact number and clash score, and the designs are computationally filtered and experimentally characterized. In this panel only one backbone is selected for clarity, but in practice, all monomer backbones are run through the same design pipeline. Source data

Both cyclic symmetry and superhelical symmetry are frequent in nature, but few systems have both cyclic and internal superhelical symmetry with coincident symmetry axes (Fig. 1a–c). This geometry has the advantage that the individual protomer can be readily extended based on the internal superhelical symmetry such that the newly added portion makes the same interactions with its cyclic symmetric counterparts as the original protomer made with its counterparts. Among protein systems, coiled coils and the collagen triple helix have this very useful property, which has been widely exploited in natural biological systems and in protein engineering^1^. This geometry has been exploited in protein design to create helical hairpins that pair with parallel or antiparallel partners to form heterodimers^2^, and these ‘base pairing’ interactions can be further expanded to create higher-order dimensional designs like cages and two-dimensional lattices^3,4^. However, the geometry of these structures has limitations: the monomers are flexible and not readily amenable to protein fusion^5^, the assemblies are restricted to a narrow range of twist and radius values and cannot readily be stacked along the axis of extension due to steric constraints^6,7^. The superhelical symmetry necessary for forming such structures is also found in helical repeat proteins, both natural and designed, composed of a globular protein unit that is tandemly repeated to form a rigid structure^8^. De novo helical repeat proteins (DHRs) have potential advantages as protomers over single helices, as they are rigid and amenable to protein fusion, can adopt a wide variety of geometries^9^ and can stack in a head-to-tail fashion by non-covalent interactions, like DNA double helices with single-stranded overhangs. However, while homo-oligomers have been generated using DHRs^10^, the cyclic axes of the oligomer and the superhelical axes of the monomers have not been coincident, so extending the monomer does not extend the homo-oligomeric interface as is the case in coiled coils and double-stranded nucleic acids. We set out to systematically generate protein nanostructures with shared cyclic and superhelical symmetry axes based on cyclic helical repeat proteins (CHRs).

## Results and discussion

### CHR multiplexes are validated to high resolution

Repeat proteins are composed of an asymmetric structural motif that is concatenated several times in tandem within a single protein chain. We began by using fragment assembly to generate a wide variety of repeat protein monomers with repeat units with a square two-helix geometry or triangular three-helix geometry and four repeats in total (Fig. 1d). The superhelix traced out by the centroids of the repeat units was computed, and from two to eight copies of the monomer were placed around the superhelical axis. Cyclic assemblies lacking backbone clashes and with extensive helix–helix intermolecular contacts were then computationally assigned sequences. We initially used Rosetta methods such as FastDesign and PackRotamers^11^ but obtained better experimental success rates using proteinMPNN, a message-passing neural net trained to predict sequences that will fold into a given protein structure^12^. We selected subsets of designed multiplexes that had backbone configurations that closely matched predictions from either AlphaFold2 or AlphaFold multimer^13,14^, and obtained synthetic genes for experimental characterization.

**Fig. 2:**
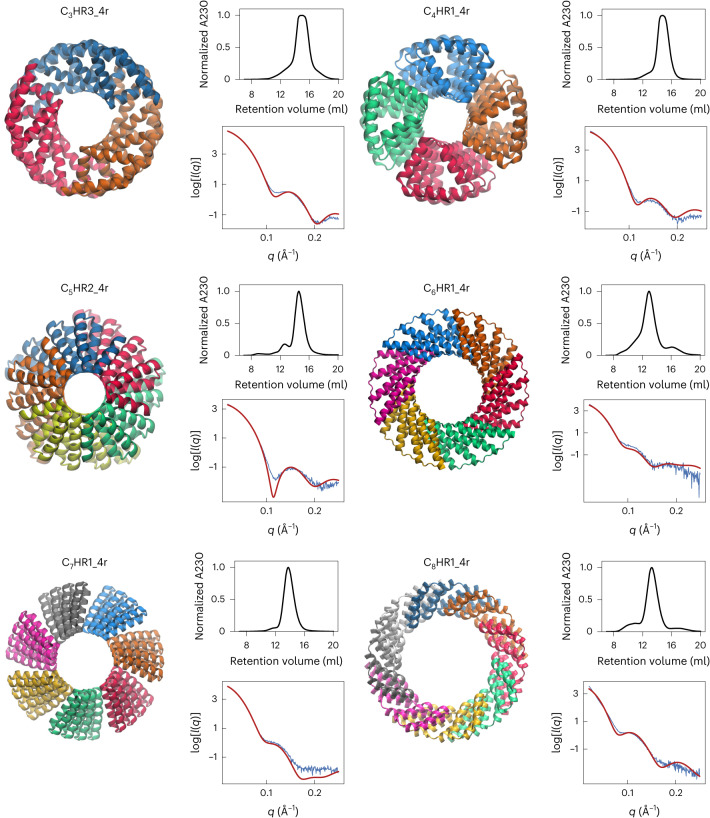
Experimentally validated multiplexes from *C*_3_ to *C*_8_ symmetry. The design models are shown with their cyclic axes pointing into the page. For each design, the top-right panel shows the SEC curve after IMAC purification, and the bottom-right panel shows the experimental (blue line) and model fit (red line) SAXS curves. For the SEC curves, A230 is the sample absorbance 230 nm. For the SAXS curves *q* is the wave vector transfer and *I* is the scattering intensity. All multiplexes follow the naming convention C_*s*_HR*N*_*R*r where *s* is the cyclic symmetry, *R* is the number of repeats in a single chain and *N* is an index to differentiate between multiplexes of the same symmetry. Source data

**Extended Data Fig. 1:**
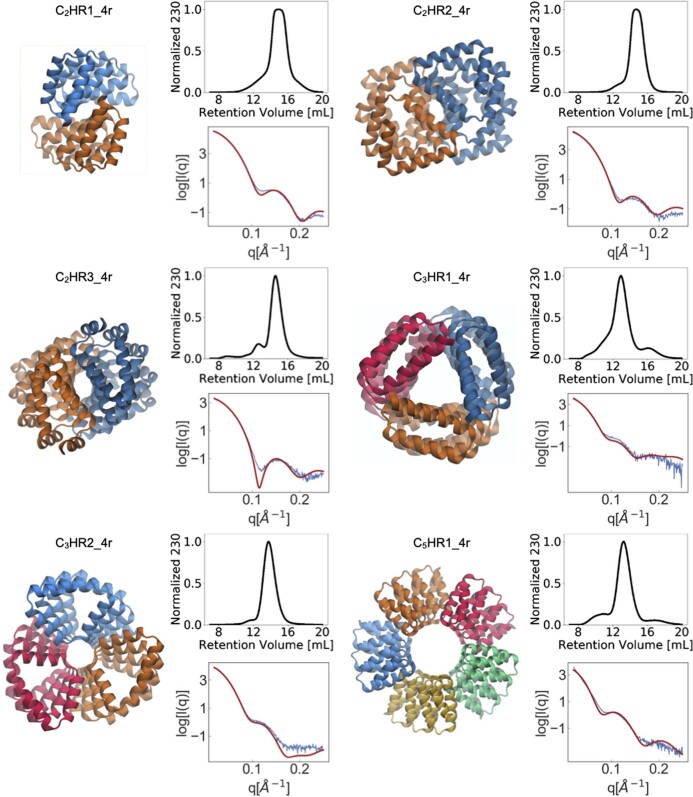
The remaining experimentally validated four repeat multiplexes. The design models are shown with their cyclic axes pointing into the page. For each design, the top right panel shows size exclusion chromatography curve after IMAC purification and the bottom panel shows experimental (blue line) and model fit (red line) SAXS curves. C_2_HR1_4r was designed using Rosetta while all other multiplexes were designed using proteinMPNN. Source data

**Extended Data Fig. 2:**
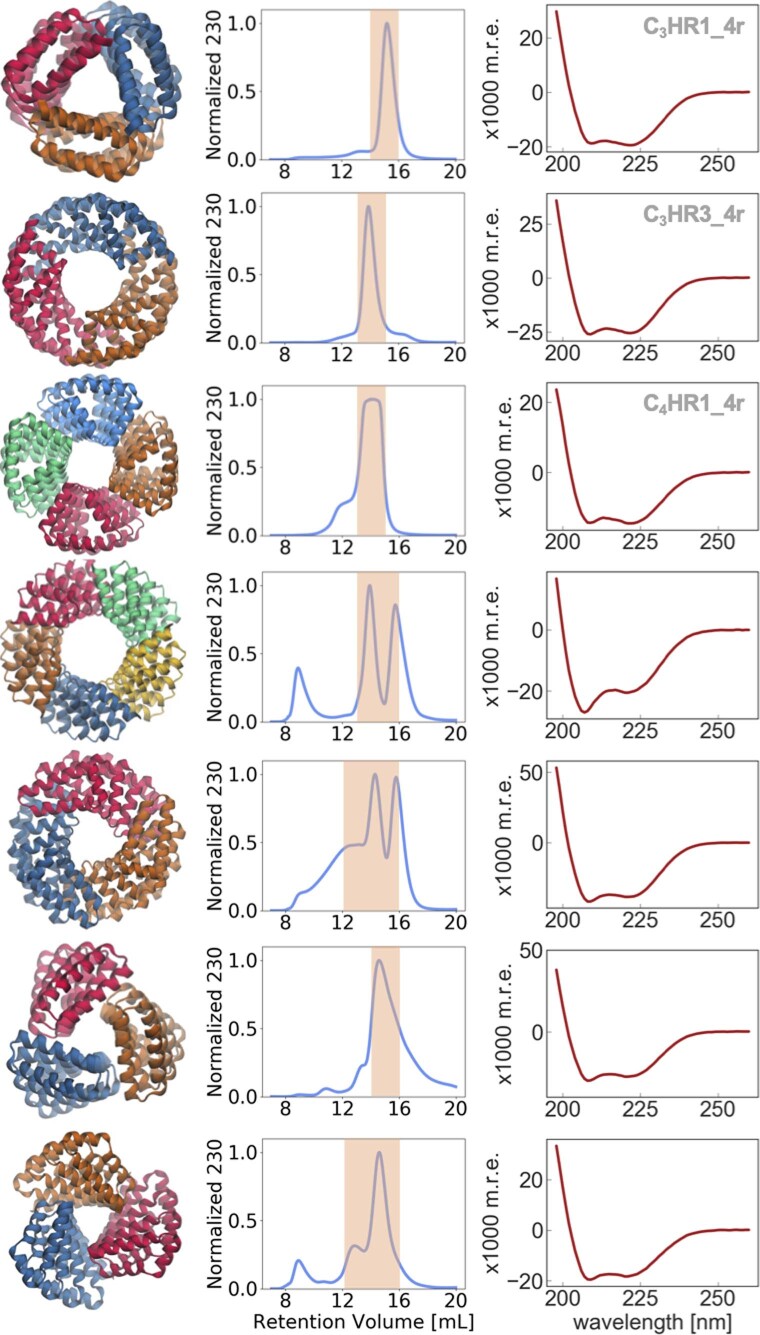
Circular dichroism data for a sample of monodisperse (top three) and polydisperse (bottom four) designs. The left column shows the designs colored by chain, the middle column is the SEC curves with pooled fractions highlighted orange, and the right column shows each CD curve measured from the pooled fractions. Source data

**Extended Data Fig. 3:**
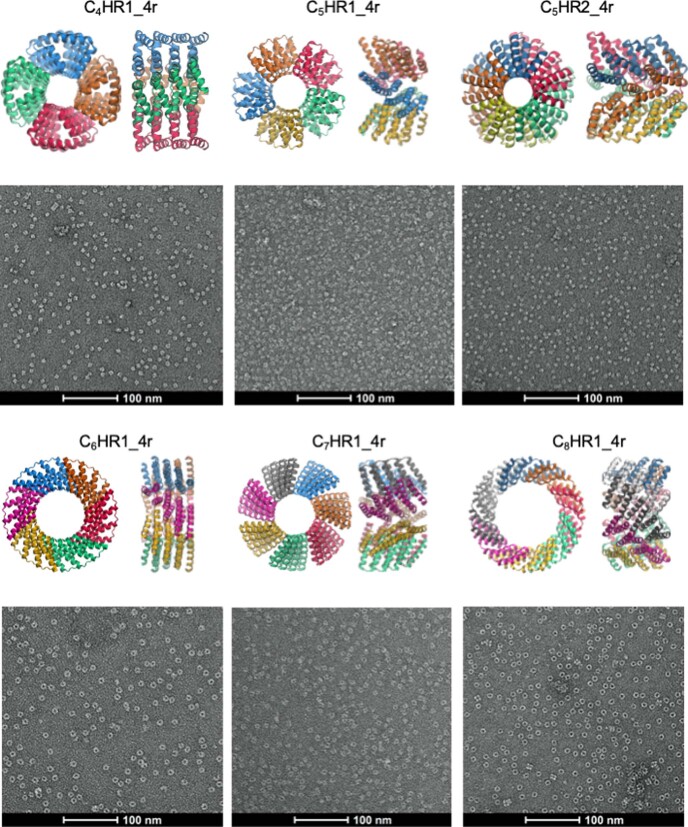
Negative stain electron micrographs of C_4_-C_8_ multiplex assemblies and corresponding design models. All micrographs were imaged at 36,000 magnification. Source data

We expressed 67 of the proteinMPNN-designed multiplexes in *Escherichia coli*, and characterized their oligomerization state by size exclusion chromatography (SEC). Of these designs, 60 were soluble and 11 of the 67 were monodisperse with elution profiles consistent with the oligomerization state. The oligomerization state of these 11 designs and one dimer designed by Rosetta were further confirmed by size exclusion chromatography-multi-angle light scattering (SEC-MALS) measurements (Fig. 2, Extended Data Fig. 1 and Supplementary Table 1). We measured the circular dichroism spectra of four polydisperse designs, and three had profiles consistent with an alpha-helical secondary structure, indicating that off-target oligomerization is likely the common failure mode (Extended Data Fig. 2). Small-angle X-ray scattering (SAXS) profiles of the monodisperse multiplexes (Fig. 2) were close to those computed from the computational design models^15–17^. The volatility ratio (*V*_r_) for each pair of curves (Supplementary Table 2 (ref. ^18^), a better determinator of goodness of fit than a simple difference of observed and expected variables (*χ*^2^) since it is less dominated by the fitting at the Guinier region) was less than 12.6 in the range of values determined for previously designed protein oligomers that have been confirmed by X-ray crystallography^19^. The *C*_4_ to *C*_8_ designs were also imaged by negative stain electron microscopy, which further confirmed that the particles are monodisperse with the correct shape and size (Extended Data Fig. 3).

**Fig. 3:**
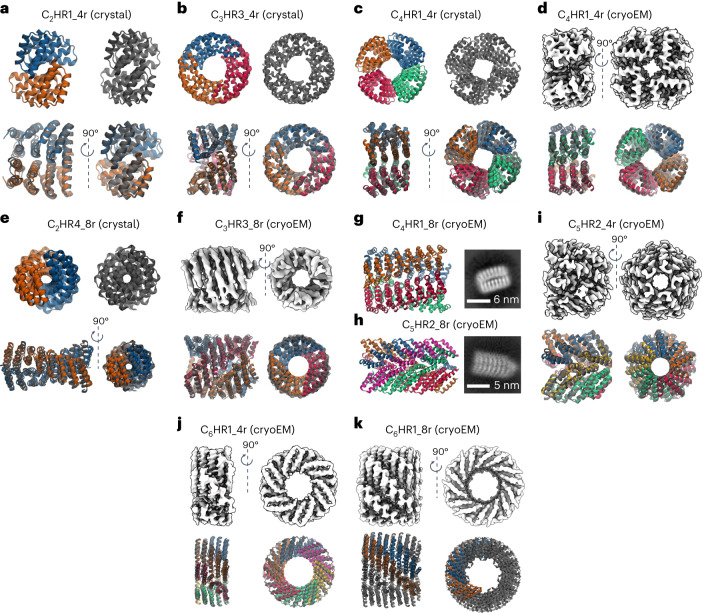
High-resolution structure determination of four- and eight-repeat multiplexes. **a**, Crystal structure of C_2_HR1_4r aligned to design models by backbone r.m.s.d. **b**, Crystal structure of C_3_HR3_4r. **c**, Crystal structure of C_4_HR1_4r. **d**, CryoEM structure of C_4_HR1_4r. **e**, Crystal structure of C_2_HR4_8r. **f**,CryoEM structure of C_3_HR3_8r. **g**, C_4_HR1_8r design model is shown as a side view with a corresponding cryoEM class average shown on the right. **h**, C_5_HR2_8r is shown as a side view with a corresponding cryoEM class average shown on the right. **i**, CryoEM structure of C_5_HR2_4r. **j**, CryoEM structure of C_6_HR1_4r. **k**, CryoEM structure of C_6_HR1_8r. Since the C_6_HR1_8r cryoEM model is *C*_7_ instead of *C*_6_, only two chains of the design model were superimposed. For all displayed structures, the experimentally determined structures are shown in grey while the backbone-aligned design models are coloured by chain. Source data

**Extended Data Fig. 4:**
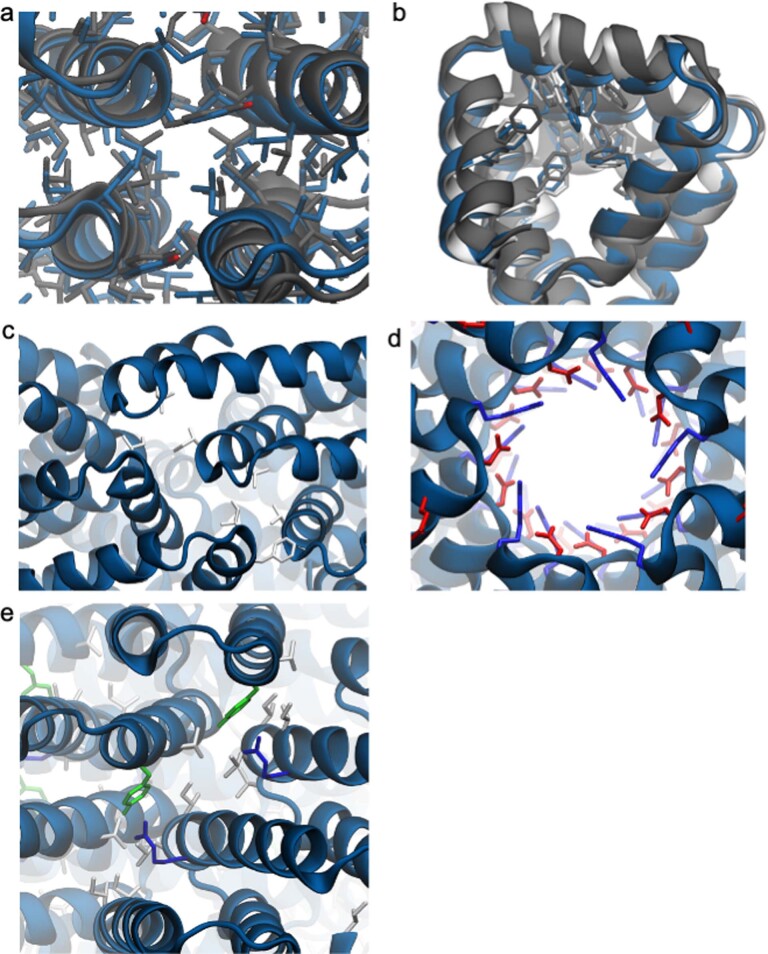
Sidechain rotamers of higher resolution structures. a. C_2_HR1_4r design model (blue) overlaid with crystal structure (gray) zoomed in on the C_2_ interface. The core, interfacial residues for both are shown. b. Buried phenylalanine residues for single chain for C_4_HR1_4r design model (blue), crystal structure (gray) and cryoEM model (white). c. Interfacial residues for C_3_HR3_4r. d. Salt bridging residues at the inner radius of C_5_HR2_4r. Residues are colored blue for basic and red for acidic. e. Interfacial residues for C_6_HR1_4r. Source data

We determined the high-resolution structures of five designs from *C*_2_ to *C*_6_ symmetry by X-ray crystallography, cryogenic electron microscopy (cryoEM) or both (Fig. 3 and Supplementary Table 3). All multiplexes follow the naming convention C_*s*_HR*N*_*R*r where *s* is the cyclic symmetry, HR stands for helical repeat, *R* is the number of repeats in a single chain and *N* is an index to differentiate between multiplexes of the same symmetry. The crystal structure of C_2_HR1_4r matches the design model with an overall backbone alpha carbon (C-alpha) root mean square deviation (r.m.s.d.) of 1.54 Å. C_2_HR1_4r has a large repetitive interface composed primarily of leucines (Extended Data Fig. 4a). For C_3_HR3_4r, the overall C-alpha r.m.s.d. between the crystal structure and design model is 1.21 Å. In contrast to C_2_HR1_4r, C_3_HR3_4r has a large twist, causing the interface to become staggered, with the top two repeats packing on the bottom two repeats of the adjacent monomer (Extended Data Fig. 4b). C_4_HR1_4r was solved at 3.3 Å by X-ray crystallography and ~3.7 Å by cryoEM; the two experimental structures are very close to the design model and to each other (r.m.s.d. values of 1.46 Å and 1.38 Å, respectively) with triangular-shaped repeat monomers with inner cavities lined by phenylalanines (Extended Data Fig. 4c). The C_5_HR2_4r interface is focused near the inner radius of the structure, and is composed of a thin strip of hydrophobic residues along the helical axis; desolvated salt bridges also line the inner radius of C_5_HR2_4r (Extended Data Fig. 4d). C_5_HR2_4r has the largest helical rise parameter of the structures, with an average helical rise of 1.1 nm per repeat. C_5_HR2_4r matches the design model with an overall C-alpha r.m.s.d. of 2.11 Å and has a *V*_r_ of 12.6, higher than that of the other designs, further suggesting that all 12 designs are close to the correct structure. C_6_HR1_4r matches the design model with an overall r.m.s.d. of 1.97 Å. Like C_5_HR2_4r and many of the other two-helix repeat oligomers, the monomers of C_6_HR1_4r interact at the inner radius of the oligomer, but the repeats fan out towards the outer radius. The C_6_HR1_4r interface may be stabilized by a repetitive cation–pi interaction between tyrosine and arginine side chains of adjacent monomers (Extended Data Fig. 4e). The high-resolution structure of C_6_HR1_4r is the widest with an outer radius of 92 Å. The inner radius is 41 Å, which is large enough to fit a C_2_HR dimer.

### Designed CHR multiplexes are extendable

Extendability is in principle a major advantage of helical repeat protein oligomers that have aligned superhelical and cyclic symmetry axes. Like the DNA duplex, they can geometrically be extended by propagating the number of repeats, and the interfacial contacts should increase with each additional repeat (Fig. 1c). To investigate such extendability, we designed eight-repeat versions of four of the validated four-repeat multiplexes. The backbones of these proteins were propagated parametrically, and the sequences were designed similarly to the original four-repeat versions. The SEC-purified proteins form monodisperse particles with the expected size as determined by SEC-MALS, and the expected shape as confirmed by negative stain electron microscopy (nsEM) and cryoEM (Fig. 3f–k). The structures of the C_3_HR3_8r and C_6_HR1_8r were further analysed by cryoEM three-dimensional reconstruction. Like C_3_HR3_4r, C_3_HR3_8r closely matches the design model with an overall C-alpha r.m.s.d. of 2.04 Å. SEC-MALS indicates a mixture of *C*_6_/*C*_7_ oligomers for C_6_HR1_8r with majority *C*_6_; while both states are apparent in cryoEM, we were able to successfully reconstruct only the *C*_7_ state, which is the largest of all the monodisperse designs with a total size of 305 kDa. The r.m.s.d. of the single monomer is 1.44 Å and of two adjacent monomers is 2.74 Å, indicating that only subtle shifts in rotation and translation at the interface were required to accommodate the extra monomer. We also expressed a *C*_2_ dimer directly as an eight-repeat duplex (C_2_HR4_8r), and the structure by X-ray crystallography to be close to the design model (r.m.s.d. compared with design model = 3.78 Å; Fig. 3e). The twist of the crystal structure is approximately 22.5° per repeat or 16 repeats per turn, and hence by propagating this structure from 9 to 17 repeats, the angle between the N and C terminal repeats can be modulated from 180° to 360°.

### Design of patterned fibres by surface redesign

In principle, the monomers of the presented multiplexes could be extended indefinitely, but increasing monomer length would also result in off-target oligomers since the individual monomers can shift farther and farther along the helical axis. Instead, self-assembly of monomers with smaller interfaces would limit slippage and favour the assembly of the desired oligomers^20^. The top and bottom surfaces of the CHR monomers are primarily hydrophilic residues in the bounded multiplexes presented in previous sections. We attempted to replace these with the hydrophobic residues found on the core repeats, but most of these ‘uncapped’ designs did not express in *E. coli*, likely due to the increased hydrophobicity and lack of specificity in forming the intended fibre geometry^21^.

**Fig. 4:**
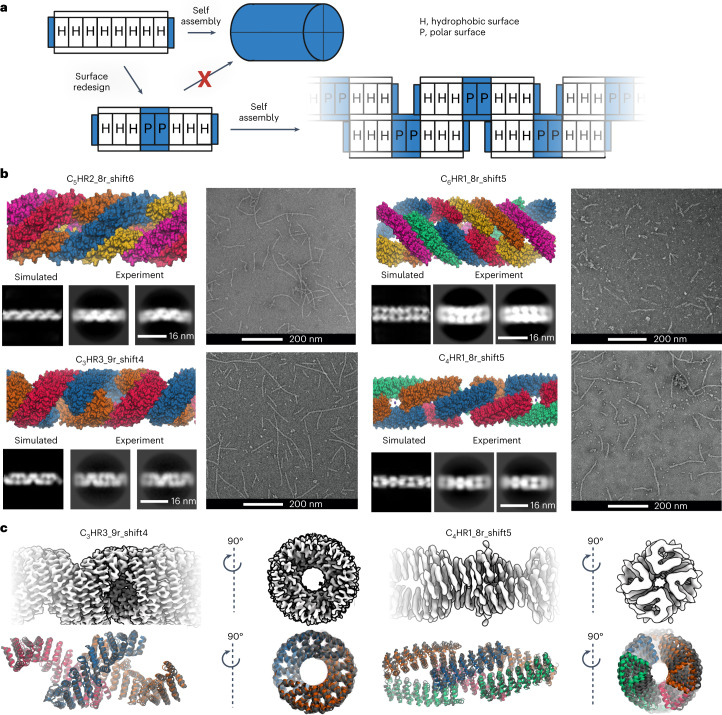
Patterned fibres. **a**, Staggered fibre design concept. A single CHR monomer has two sides with hydrophobic surfaces. Installing a central hydrophilic patch forces a staggered formation, triggering fibre assembly. **b**, Examples of successfully assembled patterned fibres. Design models are shown on the upper left; negative stain micrographs are shown on the right; and two-dimensional class averages from negative stain along with simulated class averages generated by the CryoSPARC software suite are shown on the lower left. The fibres follow the same naming convention as the original multiplexes, appended with the term shift*N* where *N* corresponds to the register shift between adjacent monomers. For example, shift5 means a register shift of 5 repeats between adjacent monomers. **c**, Three-dimensional reconstructions from cryoEM data for C_3_HR3_9r_shift4 (left) and C_4_HR1_8r_shift5 (right). The upper row shows cryoEM densities of the symmetry-expanded fibres. The lower row shows the design models aligned to the cryoEM structures. The cryoEM models are coloured grey while the design models are coloured by chain; alignment is based on the backbone r.m.s.d. of the middle, blue chain. Source data

We instead adopted a ‘Lincoln Logs’ approach that alternates non-polar patches that favour close subunit–subunit interactions with charged polar patches that disfavor burial. We hypothesized this would generate offset arrangements of the monomers, generating fibers with empty ‘pores’ in the regions of the polar patches (Fig. 4a). We generated such fiber designs from the *C*_3_ to *C*_6_ designs described above. The surface alternates from hydrophobic to hydrophilic to hydrophobic, which reduces non-specific interfacial shifting and increases overall solubility.

**Extended Data Fig. 5:**
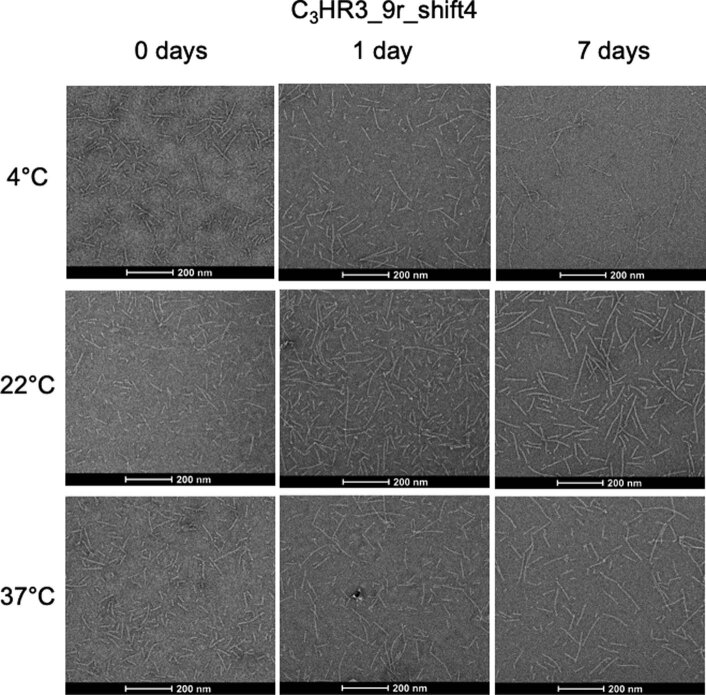
Fiber growth over time at 4 °C, 22 °C, 37 °C for C_3_HR3_9r_shift4. All micrographs were imaged at 36,000 magnification. For each temperature an image was taken at t = 0, t = 1 day and t = 7 days. Source data

**Extended Data Fig. 6:**
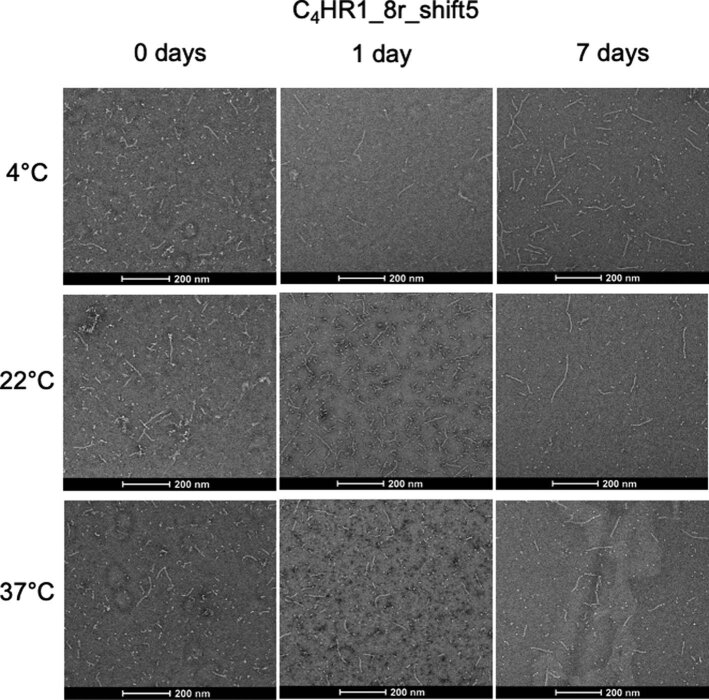
Fiber growth over time at 4 °C, 22 °C, 37 °C for C_4_HR1_8r_shift5. All micrographs were imaged at 36,000 magnification. For each temperature an image was taken at t = 0, t = 1 day and t = 7 days. Source data

We expressed and characterized 58 fibres redesigned from the four CHRs verified to be extendable. A total of 25 of the tested fibres assembled into 50 to 800 nm fibres readily observable by nsEM. The fibres are soluble, and we purified them through conventional immobilized metal affinity chromatography (IMAC) and screened the elution fractions. The fibres grow over time (Extended Data Figs. 5 and 6): for example, C_3_HR3_9r_shift4 (shift*N* means a register shift of *N* repeats between adjacent monomers) increases from approximately 50 nm to 300 nm after seven days of incubation at 37 °C; there are evidently kinetic traps during fibre assembly that can be overcome with sufficient time or heating. We selected one fibre from each of the four CHRs for further characterization by nsEM (Fig. 4b). The diameters of the fibres are closely consistent with the computational models, and the designed surface patterning closely matches the two-dimensional class averages derived from nsEM.

We characterized the three-dimensional structure of two of the fibres by cryoEM (Fig. 4c). As with the bounded designs, the cryoEM model backbones closely match the design models. C_4_HR1_8r_shift5 has an overall *C*_2_ symmetry; the fibre structure resembles chain links with each corresponding to a *C*_2_ unit. The pore size is approximately 880 Å^2^ (two repeats). The fibre has a twist of 42.6° per monomer, or nearly 90° for every two monomers, and this feature along with the *C*_2_ symmetry can be exploited to generate square lattices of fibres. The C_3_HR3_9r_shift4 was solved at 3.8 Å resolution, permitting more precise helix and side-chain assignment. The two interfaces of C_3_HR3_9r_shift4 are about the same size with approximately 38 carbon–carbon contacts (or three contacting repeats) per interface. The pore size of C_3_HR3_9r_shift4 is slightly larger (~1,020 Å^2^). Like C_4_HR1_8r_shift5, the pore size and twist closely match the design; the twist is −142.8° per monomer, or 71.4° per repeat. Notably, C_3_HR3 was verified to high resolution for the bounded four-repeat, bounded eight-repeat and unbounded fibre designs.

**Fig. 5:**
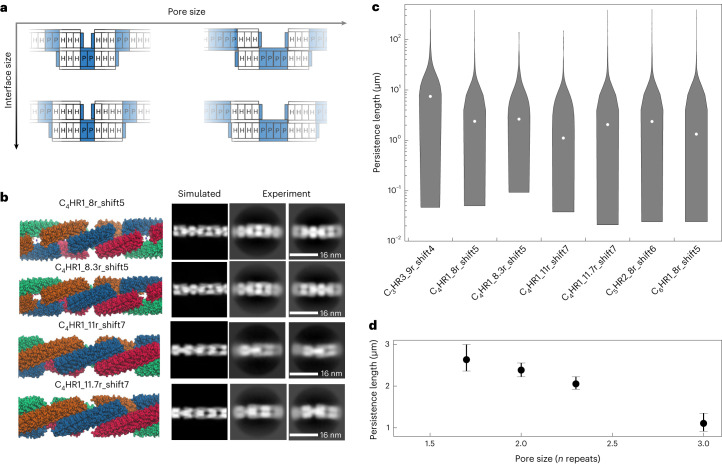
Sculpting the structures and persistence length of the patterned fibres. **a**, Schematic showing that by changing the monomer size and spacing, fibres with different pore and interface sizes can be created. **b**, Variations of C_4_HR1 fibres. Chain length and spacing are varied between the fibres. Negative stain class averages are shown on the right along with simulated class averages generated by CryoSPARC. **c**, Violin plots of persistence lengths of fibres calculated from negative stain micrographs. The white circles represent the median values. From left to right, the violin plots represent 1,924, 2,457, 436, 181, 1,432, 3,105 and 1,099 independent fibres, each measured from a single negative stain data collection. **d**, The persistence length can be systematically tuned by varying the pore size. This panel shows the same data of the C_4_HR1 fibres presented in **c**. The dots represent the median values and the error bars are 90% confidence intervals calculated from bootstrap Monte Carlo: larger pore sizes lead to a lower persistence length. Source data

For materials engineering, a very useful aspect of our fibre design strategy is that the properties of the fibres can be tuned simply by changing the number of repeat units on the monomeric subunits, and the size of the hydrophilic spacer between the hydrophobic units forming the interface: the more hydrophobic units, the larger the subunit–subunit interface between monomers, and the larger the hydrophilic spacer, the larger the pores in the resulting fibres (Fig. 5a). We explored varying both properties and found that it is possible to lengthen the monomer one helix at a time, enabling control of the pore size of the fibre with single-helix precision. Two-dimensional class averages indicate pore size and spacing consistent with the design models, with C_4_HR1_11r_shift7 having the largest pore size (Fig. 5b). We calculated the persistence lengths of the fibres from the nsEM data (Fig. 5c,d) using the SPRING electron microscopy software suite^22^. Most of the fibres have persistence lengths around 2 μm, which is between the persistence lengths of intermediate filaments (500 nm) and actin (17.7 μm). The stiffest fibre is C_3_HR3_9r_shift4 with a persistence length of 7.44 μm. While the radii of all fibres are comparable, C_3_HR3 has the largest repeat size; thus, the mechanical stiffness of the monomer may be responsible for the increase in stiffness. For C_4_HR1, we expected that the stiffness would decrease with increasing pore size. Across the four C_4_HR1 variations, we find that this is indeed the case, with C_3_HR3_11r_shift7 having the lowest persistence length as measured by springEM and as visualized by nsEM two-dimensional class averaging of these assemblies.

## Conclusions

Our designed assemblies with coincident cyclic and superhelical symmetry axes open up new frontiers in protein nanomaterial design. The designs span a wide range of monomer configurations and are readily extendable by repeat propagation. By alternating the non-polar monomer–monomer interaction regions with charged/polar surfaces that have very large solvation-free energy penalties for burial, protein filaments with different porosity and geometry can be robustly generated. The resulting porous structures could provide platforms for biomineralization analogous to collagen. The pores can also serve as binding sites for ligands containing one or two repeat units, enabling decoration of the fibres with molecules fused to these ligands at a readily tunable spacing. While here we primarily explore the assembly of one-dimensional protein fibres, it should be possible to extend our approach to two-dimensional and three-dimensional materials. For example, the filaments could be resurfaced to form three-dimensional lattices, or the bounded rings could be stacked in two dimensions to form extendable sheets.

We show that the mechanical properties of the fibres can be modulated by changing the pore size of the fibres. Smaller pore sizes result in stiffer fibres, and this mechanism can be used to tune the mechanical properties of higher-order materials built from the fibres. The tunability of mechanical properties could be useful for protein-based hydrogels, where the bulk moduli can be systematically changed by using fibres of different porosity with applications in tissue engineering and food products. The robust thermostability and high soluble yield of the designs enables large-scale manufacture using standard procedures: the proteins are produced in *E. Coli* with low cost materials (salts, yeast extract, sugar and so on) with a yield of milligrams from 50 ml cultures, which would likely scale to grams using a standard bioreactor set-up. Properties such as charge and aromaticity can be specified by the mutation of surface residues, and this can be exploited to design interfaces with materials such as graphene or silicon to generate bioelectronics. Designs with a specific twist, oligomeric state and radius can be generated to bind to other helical molecules like DNA and carbon nanotubes, openning up a wide range of application to biomedical and materials challenges.

## Methods

### Computational design of four-repeat multiplexes

The design protocol for the repeat protein multiplexes is an adapted and expanded protocol derived from Brunette et al. (2015; ref. ^10^). Repeat protein monomers were generated using the Rosetta RemodelMover algorithm. A blueprint file containing the specified secondary structure for a single repeat was provided, and the mover was specified to propagate and link this repeat four total times. Permutations of helix lengths between 9 and 22 and loop lengths between 1 and 4 were attempted for both two- and three-helix repeat structural motifs. Additional constraints were placed to ensure helix–helix contacts between neighbouring repeats. The FixAllLoops mover was used to replace any distorted loops that may have been introduced during the remodel step. All backbone monomers were filtered by motif score^10^ and the worst9mer filter. For the motif score filter, a threshold of −3.5 was used. For worst9mer, a cut-off of 0.15 for helices and 0.4 overall was used.

Satisfactory backbones were propagated to 12 repeats and aligned so that the helical axis of the monomer was aligned with the *z* axis. Once aligned, the monomer was copied around the *z* axis. Two to eight total copies were attempted, and copy numbers where there were no clashes but that had helix–helix contacts were selected. Sequences were first painted onto the backbones using the Rosetta FastDesign mover. Helical symmetry was enforced to maintain an identical sequence and backbone conformation between repeats and other chains. Once the fully symmetric sequence was designed, the system was cut down to approximately four repeats per chain. For the two-helix repeats, the chain could be cut at either the first or the second loop at the start and the end of the monomer. All four permutations were generated. For the three-helix repeats, all nine permutations were generated. A final sequence design of the protein surface was done to remove any hydrophobic patches exposed after backbone truncation. For this step, only cyclic symmetry was applied. Additionally, the surface aggregation potential score constraint was used to further minimize hydrophobic patches on the surface. For the Rosetta-designed multiplexes, 56 were tested. Of these 56, 27 were soluble, four were confirmed to have the correct oligomeric state by SEC-MALS and one (C2HR1_4r) was validated by SAXS (Supplementary Table 4). In order to increase the success rate of our designed multiplexes, we used machine learning methods to redesign the sequences.

### Protein design rescue by proteinMPNN

Designs output from the method described in the previous section were redesigned by the machine learning method proteinMPNN^12^. Tie constraints, which enforce that pairs of residues are identical, were used such that repeat symmetry of the buried core residues and cyclic symmetry across chains was maintained. Four sequences for each backbone were generated. All designs except C2HR1_4r and C2HR4_8r were redesigned using proteinMPNN.

### Protein folding validation by AlphaFold2 and AlphaFold multimer

To verify that the designed sequences would fold into the correct structure, each protein structure was predicted by AlphaFold multimer. Model 1 was used for all predictions. The alpha carbon r.m.s.d of the predictions to the design model was used to select designs for experimental screening. The *C*_8_ designs were generally too large to be reliably predicted using AlphaFold multimer. For these assemblies, we used AlphaFold2 with three chains. We input the design model as the initial guess, as this helps to find a correct solution for predicting multiple chains with AlphaFold2. For AlphaFold2, the default model 4 was used. All models give largely the same answer for these designs, so the choice of model 4 was arbitrary. Designs were again selected according to a 2 Å alpha carbon r.m.s.d. relative to the design model.

### Extension of multiplexes from four to eight repeats

A subset of the four-repeat designs that were experimentally validated was redesigned as eight-repeat versions. The backbone was first propagated to ten repeats, and then the chain was cut to approximately eight repeats. All permutations of different cut points were attempted, as was done for the four-repeat multiplexes. For all extensions, the entire sequence was redesigned using proteinMPNN. The internal repeat symmetry and cyclic symmetry were enforced. Additionally, the four inner repeats were subjected to full repeat symmetry. This was done to enable the propagation of these assemblies by copying the internal sequence without any computational redesign of the sequence. All designs were evaluated by either AlphaFold2 or AlphaFold multimer before ordering for experimental characterization.

### Patterned fibre design

To generate patterned fibres, multiplexes were extended to lengths between six and twelve repeats. Adjacent monomers were offset along the helical axis in increments of repeat height and rotation. Using these staggered monomers as a reference, helical symmetry was applied to generate copies that extend unbounded along the fibre axis. Once the fibre geometry was established, proteinMPNN was used to generate sequences while maintaining internal repeat symmetry with each monomer and helical symmetry across monomers. Fibres with suitable helix–helix contacts and absent clashes were selected for experimental characterization.

### Preparation of genes from computational designs

Monomers were reverse translated using domesticator (https://github.com/rdkibler/domesticator). These genes were ordered either by Integrated DNA Technologies or Genscript and inserted in pET29b+ vector at NdeI and XhoI restriction sites.

### Buffers and media

The lysogeny broth (LB) contained the following: 1.2% (w/v) tryptone, 2.4% (w/v) yeast extract, 0.4% (v/v) glycerol, 17 mM KH_2_PO_4_ and 72 mM K_2_HPO_4_.

The TBM-5052 media contained the following: 2.4% (w/v) yeast extract, 1.2% (w/v) tryptone, 0.5% (w/v) glycerol, 0.05% (w/v) d-glucose, 0.2% (w/v) d-lactose, 25 mM Na_2_HPO_4_, 25 mM KH_2_PO_4_, 50 mM NH_4_Cl, 5 mM Na_2_SO_4_, 2 mM MgSO_4_, 10 μM FeCl_3_, 4 μM CaCl_2_, 2 μM MnCl_2_, 2 μM ZnSO_4_, 400 nM CoCl_2_, 400 nM NiCl_2_, 400 nM CuCl_2_, 400 nM Na_2_MoO_4_, 400 nM Na_2_SeO_3_ and 400 nM H_3_BO_3_.

The lysis buffer contained the following: 25 mM Tris buffer (pH 8), 300 mM NaCl and 20 mM imidazole.

The elution buffer contained the following: 25 mM Tris (pH 8), 300 mM NaCl and 500 mM imidazole.

The SEC running buffer contained the following: 25 mM Tris (pH 8) and 300 mM NaCl.

### Protein expression and purification

Plasmids were transformed into either lemo21 or bl21de3 expression-competent *E. coli* cells. Transformed colonies were expressed by 50 ml, 24 h autoinduction. The cultures were lysed by sonication and purified using Ni-NTA immobilized metal affinity columns. Monodisperse designs that were identified as soluble by SDS polyacrylamide gel electrophoresis were purified further by SEC. For SEC, an ÄKTA machine was used with a GE Superdex 200 30×100 GL.

### Characterization by SEC-MALS and SAXS

Multiplexes identified as soluble and monodisperse by SEC were further characterized by SEC-MALS. A volume of 100 μl was injected into an Agilent 1200 high-performance liquid chromatography system fitted with a Wyatt Heleos DAWN light scattering detector and a Wyatt Optilab rEX refractive index detector. A GE Superdex 200 10×300 was used with Pierce 20 mM Tris, 150 mM NaCl, pH 8 running buffer, and ASTRA 7.0 was used for analysis.

SAXS measurements were carried out by the SYBYLIS group. Frameslice was used to preprocess the SAXS scattering data. To get a more realistic matching to experiment, histidine (HIS) tags were added to the protein structures using AlphaFold multimer, model 1. AlphaFold multimer, model 1 returned non-physical, backbone clashing solutions for the *C*_5_–*C*_8_ multiplexes. Model 3 returned non-clashing solutions for C5HR1_4r, C5HR2_4r and C7HR1_4r, so these predicted structures were used in lieu of the model 1 prediction. For C6HR1_4r and C8HR1_4r, no models produced non-clashing solutions. For these designs, the single-chain predictions were generated using AlphaFold2. The generated monomers were copied and aligned to the original design models. The SAXS curves for each HIS-tagged model was calculated using the command line implementation of FoXS.

### X-ray crystallography

SEC-purified samples were concentrated to 15–50 mg ml^–1^, and crystallization plates were set up using a Mosquito from SPT Labtech, then imaged using UVEX microscopes and UVEX PS-600 from JAN Scientific. Initial trials were carried out using JCSG I–IV, JCSG+, Morpheus and Classics1–2, as well as (+/–)-2-Methyl-2,4-pentanediol (MPD) screens, and then optimized as needed. For C2HR1_4r, crystals were grown in 0.1 M MES buffer, pH 6.0, and 3.2 M ammonium sulfate, and diffraction data were collected at the Berkeley Center for Structural Biology at the Advanced Light Source (ALS). For C4HR1_4r, crystals were grown in 12.5% (w/v) polyethylene glycol (PEG) 1000, 12.5% (w/v) PEG 3350, 12.5% (v/v) MPD, 0.02 M carboxylic acid and 0.1 M MOPS/HEPES-Na buffer (pH 7.5); and for C3HR3_4r, crystals were grown in 0.1 M imidazole HCl, pH 8.0, 15% (w/v) MPD and 5% (w/v) PEG 4000. Diffraction data were collected at the Northeastern Collaborative Access Team (NE-CAT) facility at the Advanced Photon Source (APS) at Argonne National Laboratory. For C2HR4_8r, crystals were grown using sitting drop vapour diffusion by mixing protein and crystallization solution (0.1 M Tris-HCl pH 8.5 and 25% (w/v) PEG 3000) in a 1:1 ratio.

X-ray intensities and data reduction were evaluated and integrated using XDS^23^ and merged/scaled using Pointless/Aimless in the CCP4 program suite^24^. Structure determination and refinement starting phases were obtained by molecular replacement using Phaser^25^ using the design model for the structures. Following molecular replacement, the models were improved using phenix.autobuild^26^; efforts were made to reduce model bias by setting rebuild-in-place to false, and by using simulated annealing and prime-and-switch phasing. Structures were refined in Phenix^26^. Model building was performed using Coot^27^. The final model was evaluated using MolProbity^28^. Details of data collection and refinement can be found in Supplementary Table 5.

### Negative stain electron microscopy

SEC-purified samples were diluted to ~0.01 mg ml^–1^ using SEC buffer immediately before sample application to glow discharged Gilder grids overlaid with a thin layer of carbon (Electron Microscopy Sciences). Grids were then stained using 2% uranyl formate for 2 minutes. Dried grids were screened on a 120 kV Talos L120C transmission electron microscope. The E. Pluribus Unum software (FEI Thermo Scientific) was used for automated data collection. Two-dimensional class averages and three-dimensional maps were generated using CryoSPARC^29^.

### CryoEM sample preparation, data collection and analysis

Protein samples were prepared by diluting or concentrating to 0.5–2.0 mg ml^–1^. For C4HR1_4r, C5HR2_4r and C6HR1_4r, 2.0 μl sample was applied to glow discharged CF-2/2-4C-T grids (Electron Microscopy Sciences). For C4HR1_8r, C5HR2_8r, C6HR1_8r, C3HR3_9r_shift4 and C3HR1_8r_shift5, 3.0 μl sample was applied to glow discharged 300 mesh copper quantifoil R 2/2 UT grids (Electron Microscopy Sciences). Using a Vitrobot Mark IV (FEI Thermo Scientific), samples were blotted with either −1 or 0 N blot forces from 0.5 to 7.5 s and plunge frozen in liquid ethane. All grids were screened and collected on a 200 kV Glacios transmission electron microscope (FEI Thermo Scientific) fitted with a Gatan K3 Summit direct electron detector. Videos were collected using the automated software serialEM^30^, at 0.05 frames per second for 99 frames with a dose of 50 electrons per square angstrom (Supplementary Tables 6 and 7).

Data processing of the cryoEM micrographs was carried out using CryoSPARC^29^. Videos were motion corrected using ‘Patch frame motion correction’, and contrast transfer functions were calculated using ‘Patch CTF estimation’. Images were manually curated to remove images with poor contrast transfer function fits and ice quality. For the bounded designs, particles were first selected using ‘Blob picker’, and then resulting class averages were used as templates for the ‘Template picker’. Class averages were obtained using the ‘2D class’ function. Selected two-dimensional classes were used as input for ‘3D ab initio’ reconstructions, then passed to ‘Non uniform refinement’ with symmetry applied to obtain the final maps. For the fibres, the ‘Filament tracer’ function was used to pick fibres from the images. The fibres were then class averaged using the ‘2D class’ function, and then initial filament reconstructions were generated using the ‘Helical refinement’ tool. Helical parameters were estimated using the ‘Symmetry search utility’, and these parameters were input for a final round of ‘Helical refinement’ with symmetry applied. Local resolution estimates were determined in CryoSPARC using an Fourier shell correlation (FSC) threshold of 0.143.

### CryoEM model building and validation

The de novo predicted design models for each design (reported here) were used as initial references for building the final cryoEM structures. The models were manually edited and trimmed using Coot^27,31^. We further refined each structure in Rosetta using density-guided protocols^32^. Electron microscopy density-guided molecular dynamics simulations were next performed using Interactive Structure Optimization by Local Direct Exploration (ISOLDE)^33^, with manual local inspection and guided correction of rotamers and clashes throughout simulated iterations. ISOLDE runs were performed at a simulated 25 K, with a round of Rosetta density-guided relaxation performed afterwards. This process was repeated iteratively until convergence, and high agreement with the map was achieved. Multiple rounds of relaxation and minimization were performed on each design, followed by human inspection for errors after each step. Throughout this process, we applied strict non-crystallographic symmetry constraints in Rosetta^34^. Phenix real-space refinement was subsequently performed as a final step before the final model quality was analysed using Molprobity^28^ and EMRinger^35^. The only deviation from this pipeline was with C6HR1_8r, which deviated substantially from the design model. Monomers for C6HR1_8r were first rigid-body docked individually into the *C*_7_ cryoEM map using Chimera^36^, followed by an initial round of Rosetta using density-guided protocols. Following this, the model was iterated and finalized similarly to the other six structures. Figures were generated using either UCSF Chimera or UCSF ChimeraX^37^.

## Online content

Any methods, additional references, Nature Portfolio reporting summaries, source data, extended data, supplementary information, acknowledgements, peer review information; details of author contributions and competing interests; and statements of data and code availability are available at 10.1038/s41557-023-01314-x.

### Supplementary information

Supplementary Information Supplementary Figs. 1–8 and Tables 1–7.

### Source data

Source Data Fig. 1 Protein model images.

Source Data Fig. 2 Protein model images, SEC source data and SAXS source data.

Source Data Fig. 3 Protein model images, crystal structure images and cryoEM map images.

Source Data Fig. 4 Schematic image, nsEM images, cryoEM map images and protein model images.

Source Data Fig. 5 Schematic image, nsEM images and persistence length source data.

Source Data Extended Data Fig. 1 Protein images, SEC source data and SAXS source data.

Source Data Extended Data Fig. 2 Protein images, SEC source data and circular dichroism source data.

Source Data Extended Data Fig. 3 The nsEM images.

Source Data Extended Data Fig. 4 Protein images.

Source Data Extended Data Fig. 5 The nsEM images.

Source Data Extended Data Fig. 6 The nsEM images.

## Data Availability

Data, atomic coordinates and structure factors for the crystal structures reported in this paper have been deposited in the Protein Data Bank (PDB) with the accession codes C2HR1_4r (8EOV), C3HR3_4r (8EOZ), C3HR1_4r (8EOX) and C2HR4_8r (8ERW). Data, atomic coordinates and structure factors for the cryoEM structures reported in this paper have been deposited in the PDB with the accession codes C4HR1_4r (8GA9), C5HR2_4r (8GAQ), C6HR1_4r (8GAA) and C3HR3_9r_shift4 (8G8I), and in the Electron Microscopy Data Bank (EMDB) with accession codes C4HR1_4r (EMD-29894), C5HR2_4r (EMD-29904), C6HR1_4r (EMD-29849), C3HR3_8r (EMD-29847), C6HR1_8r (EMD-29680), C3HR3_9r_shift4 (EMD-29856) and C4HR1_8r_shift5 (EMD-29851). All cryoEM models with associated maps can be found here: https://figshare.com/articles/dataset/cryoEM_maps_models_tar_gz/22233706. Source data are provided with this paper.

## Code availability

The code used to generate the designs presented in the paper can be found at the following repository: https://github.com/nbethel/CHR_multiplexes.
