# Supplementary material for: Precisely patterned nanofibres made from extendable protein multiplexes
Source: Nat Chem. 2023 Sep 4;15(12):1664–71. doi: 10.1038/s41557-023-01314-x (PMC10695826; doi:10.1038/s41557-023-01314-x)
Supplement: Supplementary file 1 — Supplementary Figs. 1–8 and Tables 1–7. [file 41557_2023_1314_MOESM1_ESM.pdf]

nature chemistry

Article https://doi.org/10.1038/s41557-023-01314-x

Precisely patterned nanofibres made from extendable protein multiplexes

In the format provided by the authors and unedited

Supplementary Figures

Supplementary Fig. 1: Representative x-ray crystallography electron densities maps.

Supplementary Fig. 2: CryoEM micrographs and processing plots for C_4HR1_4r.

Supplementary Fig. 3: CryoEM micrographs and processing plots for C_5HR2_4r.

Supplementary Fig. 4: CryoEM micrographs and processing plots for C_6HR1_4r.

Supplementary Fig. 5: CryoEM micrographs and processing plots for C_3HR3_8r.

Supplementary Fig. 6: CryoEM micrographs and processing plots for C_6HR1_8r.

Supplementary Fig. 7: CryoEM micrographs and processing plots for C_3HR1_9r_shift4.

Supplementary Fig. 8: CryoEM micrographs and processing plots for C_4HR1_8r_shift5.

Supplementary Tables

Supplementary Table 1: SEC-MALS measurements of bounded multiplexes.

Supplementary Table 2: Analysis of SAXS scattering curves.

Supplementary Table 3: RMSDs for design models vs high resolution structures.

Supplementary Table 4: Symmetry, sequences, solubility and oligomerization checks for presented CHR multiplexes.

Supplementary Table 5: Data collection and refinement statistics (molecular replacement).

Supplementary Table 6. CryoEM Data Collection Statistics for Bounded Designs

Supplementary Table 7. CryoEM Data Collection Statistics for Unbounded Designs.

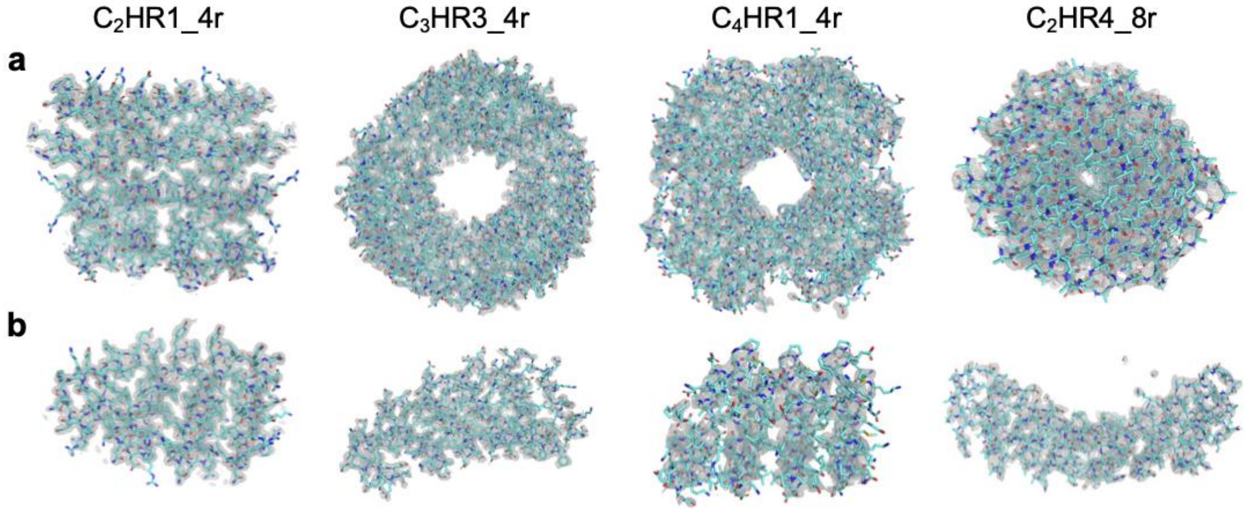

Supplementary Fig. 1: Representative x-ray crystallography electron densities maps. a. 2Fo-Fc contoured at 1 s.d. calculated using the final refined model. The full assemblies are superimposed. b. Single chain of models superimposed into the 2Fo-Fc maps.

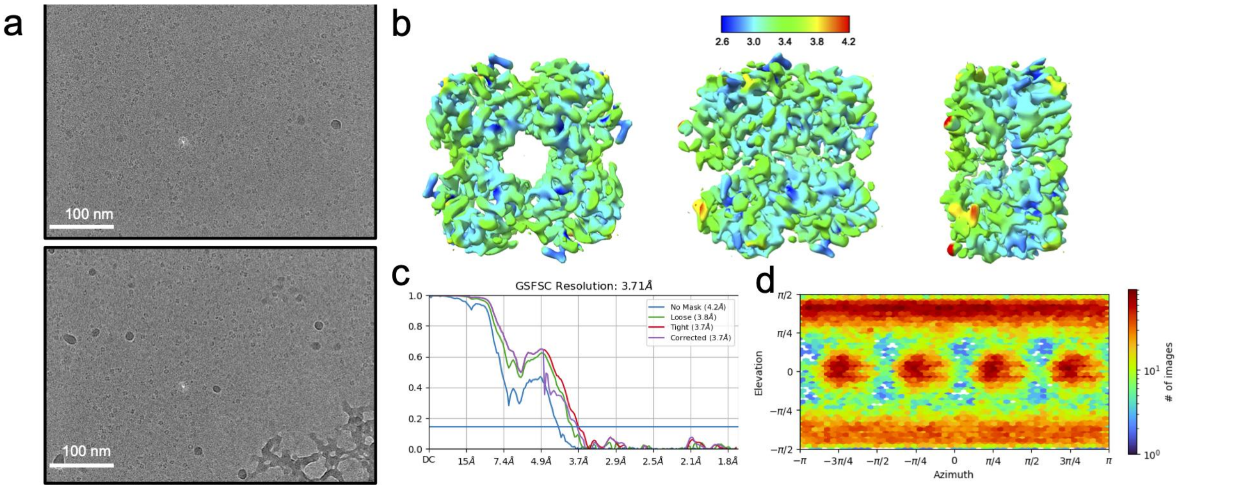

Supplementary Fig. 2: CryoEM micrographs and processing plots for C_4HR1_4r. a. Representative raw micrographs. b. Local resolution overlaid on density isosurface. c. Gold standard Fourier shell correlation curves. d. Viewing direction distribution.

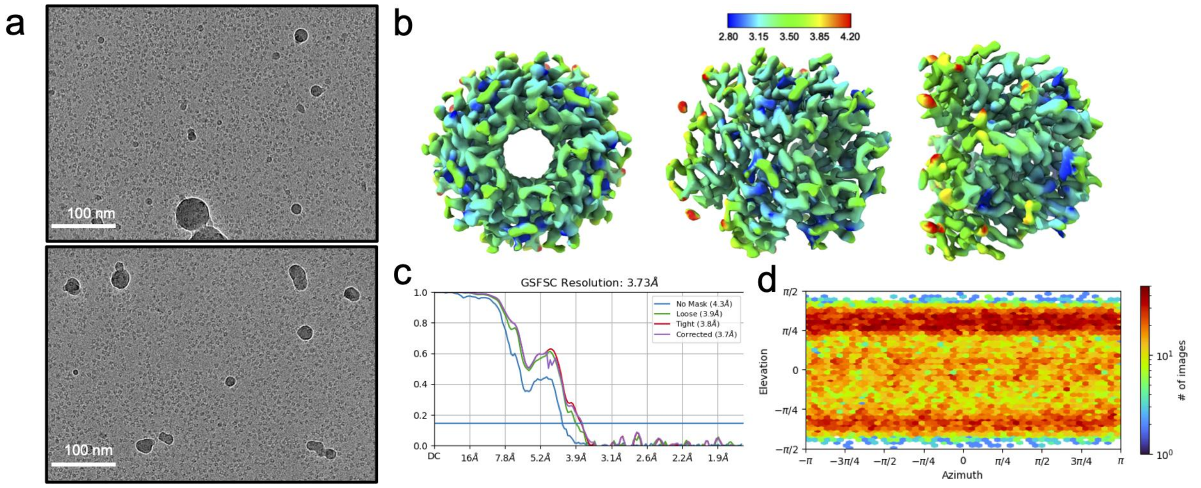

Supplementary Fig. 3: CryoEM micrographs and processing plots for C_5HR2_4r. a. Representative raw micrographs. b. Local resolution overlaid on density isosurface. c. Gold standard Fourier shell correlation curves. d. Viewing direction distribution.

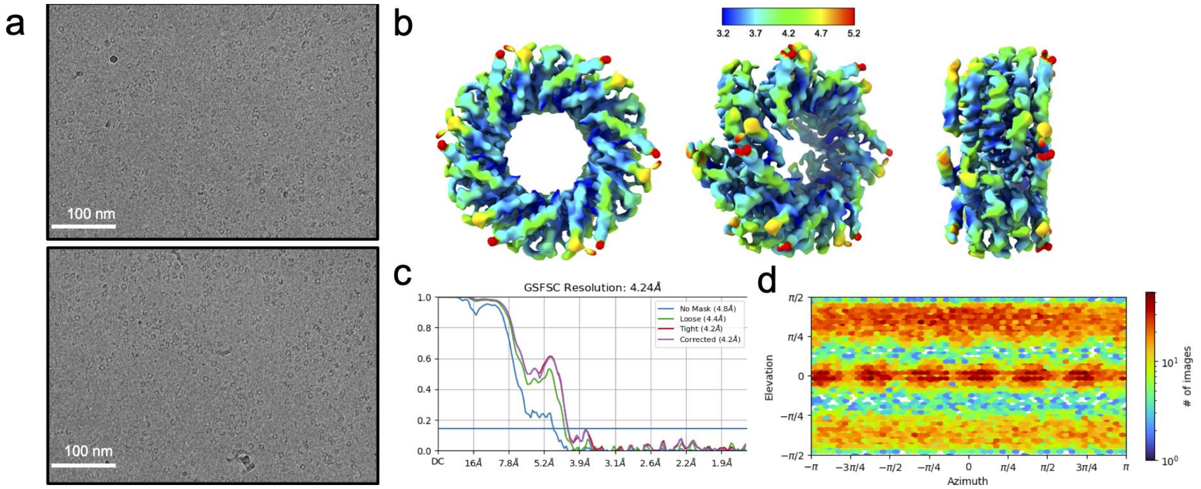

Supplementary Fig. 4: CryoEM micrographs and processing plots for C_6HR1_4r. a. Representative raw micrographs. b. Local resolution overlaid on density isosurface. c. Gold standard Fourier shell correlation curves. d. Viewing direction distribution

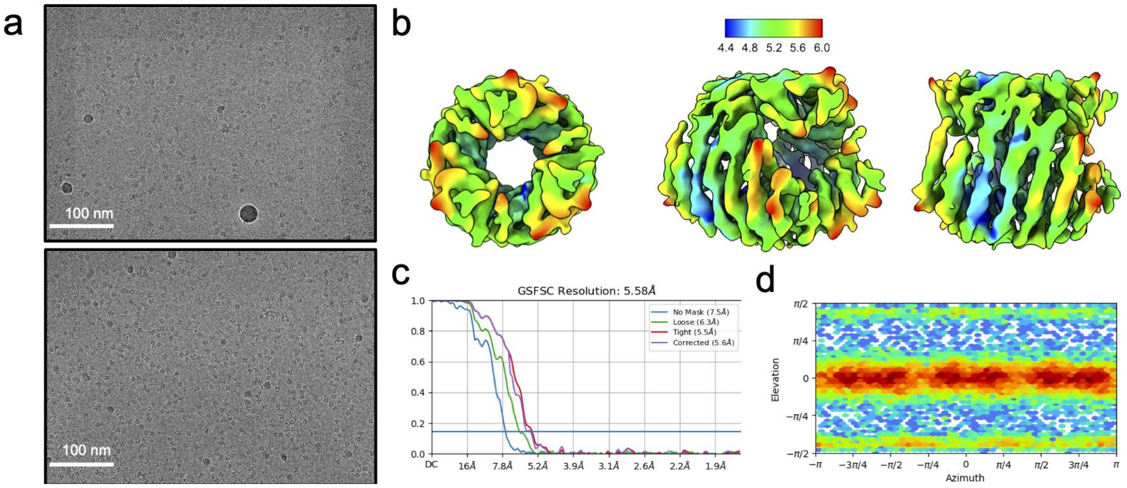

Supplementary Fig. 5: CryoEM micrographs and processing plots for C_3HR3_8r. a. Representative raw micrographs. b. Local resolution overlaid on density isosurface. c. Gold standard Fourier shell correlation curves. d. Viewing direction distribution

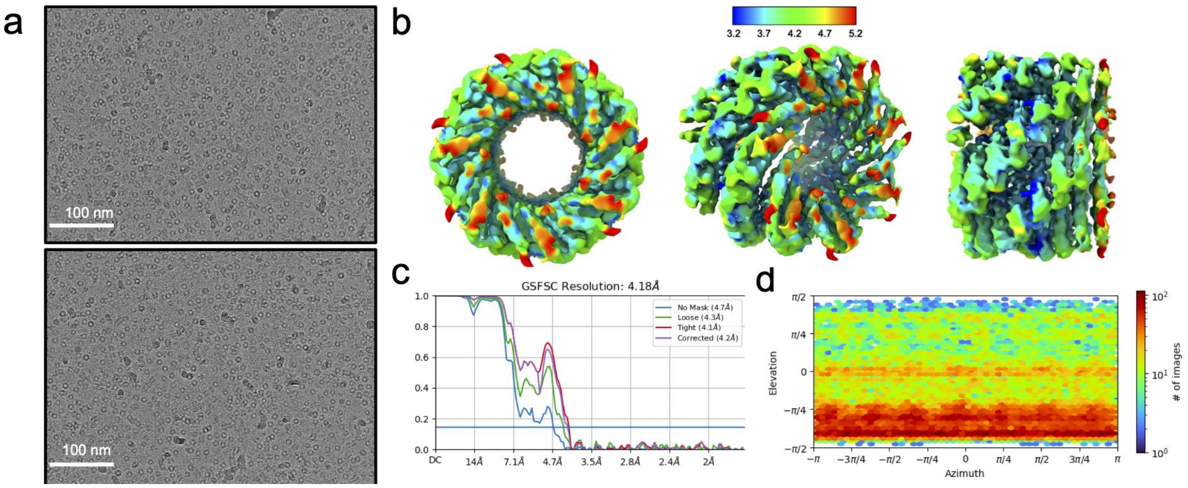

Supplementary Fig. 6: CryoEM micrographs and processing plots for C_6HR1_8r. a. Representative raw micrographs. b. Local resolution overlaid on density isosurface. c. Gold standard Fourier shell correlation curves. d. Viewing direction distribution.

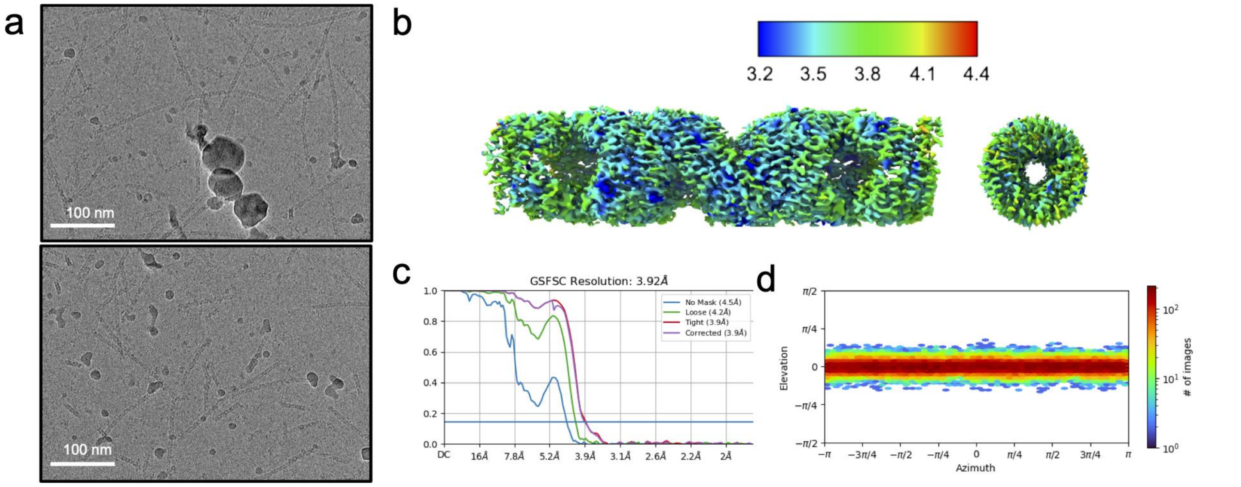

Supplementary Fig. 7: CryoEM micrographs and processing plots for C_3HR1_9r_shift4. a. Representative raw micrographs. b. Local resolution overlaid on density isosurface. c. Gold standard Fourier shell correlation curves. d. Viewing direction distribution.

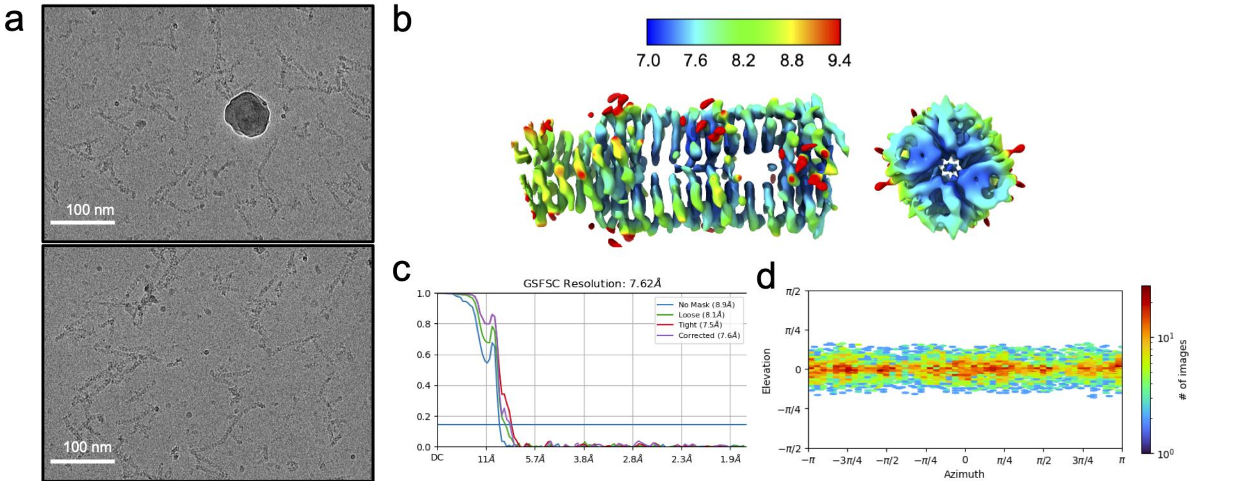

Supplementary Fig. 8: CryoEM micrographs and processing plots for C_4HR1_8r_shift5. a. Representative raw micrographs. b. Local resolution overlaid on density isosurface. c. Gold standard Fourier shell correlation curves. d. Viewing direction distribution.

Supplementary Table 1: SEC-MALS measurements of bounded multiplexes.

	**Expected [kDa]**	**Observed [kDa]**
**C_2HR1_4r**	36.8	41.17 (±10.211%)
**C_2HR2_4r**	46.1	40.59 (±20.023%)
**C_2HR3_4r**	58.7	56.94 (±19.229%)
**C_3HR1_4r**	61.2	53.82 (±18.653%)
**C_3HR2_4r**	67.6	68.27 (±21.962%)
**C_3HR3_4r**	84.7	77.47 (±19.192%)
**C_4HR1_4r**	88.2	94.1 (±23.838%)
**C_5HR1_4r**	99.0	93.4 (±20.785%)
**C_5HR2_4r**	92.5	89.71 (±19.229%)
**C_6HR1_4r**	145.3	143.3 (±14.278%)
**C_7HR1_4r**	160.7	146.2 (±19.392%)
**C_8HR1_4r**	144.2	135.2 (±17.696%)
**C_3HR3_8r**	141.1	129.4 (±18.400%)
**C_4HR1_8r**	171.6	168.0 (±16.541%)
**C_5HR2_8r**	159.5	146.9 (±15.155%)
**C_6HR1_8r**	261.4	262.2 (±14.237%)

Supplementary Table 2: Analysis of SAXS scattering curves.

	Radius of gyration (model) [Å]	Radius of gyration (experiment) [Å]	I(0) [a.u.]	V_r	Chi^2
C_2HR1_4r	19.74	26.1	6.709	6.511	0.445
C_2HR2_4r	22.313	28.302	29.978	7.973	1.997
C_2HR3_4r	22.317	25.319	30.194	3.087	0.623
C_3HR1_4r	22.552	25.904	106.523	4.044	2.777
C_3HR2_4r	28.436	24.530	23.417	6.164	0.175
C_3HR3_4r	27.742	29.494	102.630	3.964	1.447
C_4HR1_4r	26.879	32.472	78.983	4.393	1.495
C_5HR1_4r	28.123	31.799	32.880	6.194	0.384
C_5HR2_4r	26.587	32.513	32.390	12.223	0.933
C_6HR1_4r	35.314	41.314	45.863	6.317	1.514
C_7HR1_4r	33.968	37.277	60.060	5.173	0.556
C_8HR1_4r	39.128	55.374	52.849	8.808	7.031

Supplementary Table 3: RMSDs for design models vs high resolution structures.

Design name (PDB)	Structure determination method	Resolution [Å]	Monomer C-alphas	Monomer C-alpha RMSD [Å]	Overall C-alphas	Overall C-alpha RMSD [Å]
**C_2HR1_4r (8EOV)**	crystallography	1.6	139	1.15	278	1.54
**C_3HR3_4r (8EOZ)**	crystallography	3.0	227	1.04	681	1.21
**C_4HR1_4r (8EOX)**	crystallography	3.3	164	1.14	656	1.46
**C_4HR1_4r (8GA9)**	cryoEM	3.7	165	1.03	660	1.38
**C_5HR2_4r (8GAQ)**	cryoEM	3.7	147	1.50	735	2.11
**C_6HR1_4r (8GAA)**	cryoEM	4.2	197	1.06	1182	1.97
**C_3HR3_8r**	cryoEM	5.6	389	1.44	1167	2.04
**C_6HR1_8r**	cryoEM	4.2	368	1.44	736 (2 chains)	2.74 (2 chains)
**C_2HR4_8r (8ERW)**	crystallography	2.9	260	2.85	509	3.78
**C_3HR3_9r_ shift4 (8G8I)**	cryoEM	3.9	465	1.10	930 (2 chains)	1.29 (2 chains)
**C_4HR1_8r_ shift5**	cryoEM	7.6	341	2.41	682 (2 chains)	2.49 (2 chains)

Supplementary Table 4: Symmetry, sequences, solubility and oligomerization checks for presented CHR multiplexes. A selection of 10 proteinMPNN (mpnn1-10) and 10 rosetta (rosetta1-10) designed sequences for designs the solubility, oligomeric state or SAXS validation are also included, for comparison.

	Cyclic symmetry	Sequence	Soluble	Correct oligomeric state
**C_2HR1_4r**	2	MDYQEALLELIERLLRKLNVDPRDIKRIEQQLRDLDIYQIALLLLIILLLRKLNVDPRDIKRILQQLIDLDIYQIALLLLIILLLHKLNVDPRDIKRILQQLIDLDIEQIAELLLRILELRKRNEDPRDIKRELQQLIDDDLEHHHHHH	yes	yes
**C_2HR2_4r**	2	MMREELLAKLVPESLRWMRELLRETCERLVERGDEVAQELLDVMEALEEDEERFLEHLKLVPLSEQWMEMLLFLTCLALVEEGDEVARKLLEVHVELLKDKERFRELLKLVPSSLRWMEQLLELTCLSLVMEGDEVAREALEVLIELREDEEFFRELLELVPRGLRWLEILFFLTCLALMREGDEEARALLRERIELLRDREAFLRYLKEVPLERRTEELLERIRRLRKRMGSWLEHHHHHH	yes	yes
**C_2HR3_4r**	2	MMYEEFFERLRAEDPRLRELERKEEEVREKDPEIAFAYESTKENIDVLIPEEYKKWLLDELFNDPRLRELFVKAKEVEVENPKIAIAYNSTIVNLLVLIPKEYRKWFLDLLKNDPRLRELFVKFIEVAVENPKIAIAYLSTIVNLLVLKPEEELEKELNALFNDPVLREEFVEEIRVAVENPKEAIKKKIEEVIKLLEGSWLEHHHHHH	yes	yes
**C_3HR1_4r**	3	MERVKEVRETMDEFIAVMAGRDPEEIKKVVEKLKELHKTGTPTTRVIIVDKVMEAFRMVMAGRDPESIKHVVELLKWLHKHGDPRHRVIIVRKTMDTFIDVMAGQDPESIKHVVELLKWLYEHGTPNERVIIRRKVREAAEIVKEGGDPESLAAVEELLAWLEEGSWLEHHHHHH	yes	yes
**C_3HR2_4r**	3	MELEKLKEMWEKSCSPGMVEAFALLDELLTPEERAAIKRIAENMDEELQKLFLMYLLSCSIGEIYAEILLLKELTPEERKAIFEFIENTDKELMKLLLMYKLSRSIGMLYAYLELLRELTPEERKAIFDLLLNMDKEEMEDFLLKLLSQSIGEIYRELRKLRELSPEERREIFKKKEGSWLEHHHHHH	yes	yes
**C_3HR3_4r**	3	MEEVKKKLEEVWKKAKEDAGDNEKFLELLELILENPEILEILELYVFINKEDVVEKLFDVIKKAVEDAGDNEKFLELLKEMLSNPEIFEILLEYVYIKKEDVVEKLFEVIKQAVEDAGDNPVFLKLLKKMISNPEIFEILLEYVYIGKEEVVKKFFEVIKQAVEDAGNNPIFLKLLEKIILDPERFKKLLEKVEVGEEEEVKAEFKEIKKAVEEAGNDPIKLKELEEKLGSWLEHHHHHH	yes	yes
**C_4HR1_4r**	4	MMKKELYEFYFMPPLKQIEFLEELVNNPEKFKEFFKRLKEEPGAMELFLRNLYLMHPMVQIYFLELLVENPELFKLFFEYLEECPGAMEQFLLNLYLLHPMVQIEFLKLLVENPELFRLFFEYLKRCPGALELFKEIIELLDPIIQKYLKKLLEENPELKALVKEVEEEGSWLEHHHHHH	yes	yes

**C_5HR1_4r**	5	MSAKEIQDILERAEEVVEKGSIKDFLEVLELVKNCE<br>DEEVRNECIKKLAEAVLKMGDIICFLEVLKLVKECEN<br>EEVRNECIKILALAVVKMGDILCFLEVLELVKNCPNE<br>EVRNECIRLLAIAVLKMGSKTALAEVKKLVENCPNE<br>EIREECKKILALAEEEGSWLEHHHHHH	yes	yes
**C_5HR2_4r**	5	MVEELKRKLRQAKEDGDEELLERVKNEMLLLAVVD<br>PRVLVEVLNTAKELGDEEMYKKVKGIMLRLAVVDP<br>RVLVLVLELAEALGDEEMKEKVKNIMLLLAVVDPRV<br>LVLVLELAEELGDEEMKKEVEEILDKLAEVDPRVAVL<br>KEVAKKEGSWLEHHHHHH	yes	yes
**C_6HR1_4r**	6	MEEKIKELEEKVEELVKEALEKKDPAVLKKALVCVY<br>EMKKLGMPNEKLIELLKKLVEVLKKLALERVDPAVL<br>DLALVCVYEMKELGMPNEELIKLLKELVEVLRILALIN<br>VDPAVLDKALVCVYLMKELGMPNEELIKLLEELVEV<br>LRILALIRVDKRVLDKAEVCIEEMEELGMPEEKIKEL<br>REELKFVREILDKLGSWLEHHHHHH	yes	yes
**C_7HR1_4r**	7	MKEVKEKLKKKLEKCKATGDEQDYMDLMKECEKL<br>AKRGCIRGDLETVDTVLEFMLEVCKATGKPEFYQF<br>LMDTCEELARLGCILGNTKTVGLVLLKMLEVCEETG<br>DPEFYRKLMECCEELALLGCRLGNTVTVALVLFFML<br>EVCRATGDPEFFERLRRTCERLAALGKELGNEKIVA<br>LVEFFIEVVDRAGSWLEHHHHHH	yes	yes
**C_8HR1_4r**	8	MEEVLEELKKRLEEAKEKGDYEEIKKYLGSIAIVEN<br>NPEVVLKALEERYEIALLTGDFEGVRKYLGSIAIVKN<br>DPEVVLEALETRYLIALMEGDAEEIRKYLRSIAIVKG<br>DKEEEKKALKTLLLVALMEGDEEAVKEYKEKIKEVG<br>SWLEHHHHHH	yes	yes
**mpnn1**	2	MMRAREEAAVAADPRITRLVEAYRLLRRALGETDE<br>EVEAVAARLEANPIYRALMEAVLADPRILRLRYAREI<br>LRRALGETLSQEVEVFERLLKDPLFQALMRAILADP<br>EILRLVYAYEILRLALGETLEEVLECWEELRKDPRFQ<br>AWMRETLADPARLRRVFEELILQLARGETLAEIVAR<br>WKEEGSWLEHHHHHH	yes	yes
**mpnn2**	2	MEELKEKLKEAKETNDIELMKEILREILSDSEIIKEVL<br>KEGLLEKILEVALELAEKYNDIELMKLILETILSHSEII<br>KVVLEKGLLEKILEVALKLAKEKKDIYLMKLILETILSD<br>KRIIEKVLEKGLLEEILEVALELAEENEDPLLIKLILDLI<br>NSDERIKKVVEAKGLYEKILKVKEKLEKKYGSWLEH<br>HHHHH	yes	no
**mpnn3**	3	MVWEKIKKITLKKVKEEGLTEEELKEILKKIKESPVM<br>KLYRGAKYAGDDVTLDFIEETWKECIEEGKTFEELE<br>KRVEEWWNSPVNRLRRGARYAGDDVTLGFIKLTW<br>DKMKEEGKTLEELEEEVRRWQESPVFALLREALRD<br>GDNVRLGIIRLTVYECIVEGKSLEELEARIAEWRESP<br>VFALLDAALAAGDRATLGFIRATLYEAVVRGESLEEL<br>ERRVAAAAGSWLEHHHHHH	yes	yes
**mpnn4**	3	MSKSEKVKNTLEEVKEVMKEGKPEKAYELLKELAD<br>EISSDLEFVEFVEETDKENISEYVELTLELVEEFIKAG<br>EPEKAARLLELLASLISSSLNFVLFVEYTKKENIEKLV	yes	no

		EQTLELVDIFMEEGKPLLAARLLELLASLISTELQFKIFVEYTKKENIQKYVRKTLELVRIFMEEGKPLLAAELLELIESLISTEEQRKLLEEYTLPENLKEKKELKEELEKIFKEGSWLEHHHHHH		
**mpnn5**	4	MFYDVMQWIRPASYHAAMAALQAKNPALAALHAMVEEAAKANPFFAVMLHIRPADYWLALAALMAENPELLALHKLVLDAAMKNRRFAVMLFIRPASYWLALYALMAENPELLALFKLVIEKAVSNPEFLRKLYETPSSVWLFKFAEMAENPELLAAYKEEIEALGSWLEHHHHHH	yes	no
**mpnn6**	4	MKEHLLELFDEIAFSNKARRYWFELYPEIYEYWKKLIKECSIEKGHLLLIFFEIAFSNLERIYWFLLYPEIYKKWLDLIYECSIDKGHLLLIFFEIAFSNLERIFWFLKYPEIVLKAFELIYECSIEKGHELLLFLEESFSNLKKIFKNLKNPEEFLKELEEIAEGSWLEHHHHHH	no	N/A
**mpnn7**	5	MFHEQLKEDALELGTNEKLKNAALEALDEIEDPEHRLLIIDALELGKTTTLKLAAIKALMEIEDLEHRTLIIDALELGKTTRLRLAAIEALMEIEDLEHRRLIIRALELGTTTTERLAAIKRLMEIEDLEERRAIIKELEGSWLEHHHHHH	no	N/A
**mpnn8**	5	MLEELLELIGEADLSGEKEAKEEVIRKLKEYVEEKKKEGVDPRDVLRDLIGLADISGLARAKVFVIKKLKEYVKEMREKGVDPWEVLEELIGLADLSGLALAKVFVIEKLEEYVKEMLEEGVDPDEVYARLVELAELSGLALAKVLVRRWEKELKELKEGSWLEHHHHHH	yes	no
**mpnn9**	6	MSEIDKIIEEYEKKAKEEGVSKKVIGGIIGWMRRILAAGRPEIAEIIREYGIKASKFGVSPTVTGGIIGWMEEILEAGRPEIAERIREYGLEASAFGVSPRVTGGIMDWMIEILEAGRPDLAEKIARLGLRASARGVSPEEVERIMEEMIREEGSWLEHHHHHH	no	N/A
**mpnn10**	8	MRKELTDLMLELCRAEDVEVQLACVDRFLEVTENLDDETRLTELEVLLCLAEDERVQLRCVEAFLEVTESLDVEERLAALKRLLRLCEDPRCQRMCVVAFLYVTEELDDETRLAALEELRELCEDPRIRKVCDVRAAYVERGSWLEHHHHHH	no	N/A
**rosetta1**	2	MEEEQLEEKRQEIEKALKKNNIEELIELILELIAKIREIAKTEEEQLEILRQLIEKALKKNNIILLILLILILIALILEIAKTEEEQLEILRQLIEKALKKNNIILLILLILILIALILEIAKTEEEQLEILRQLIEKAEKKNNRILLELLRLILEALQREIAKTGSWLEHHHHHH	no	N/A
**rosetta2**	2	MKEETEKRIREAKEEVEKRIKRTSDEKQQIEEVQQIIKELLLEAIAEGNKEILELVIRIAKELVEKLIKRISDEKLQIEVVQQIIKILLLFAIVVGNKEILELVIRIAKELVEKLIKRISDEKLQIEVVQQIIKILLLFAIVVGNKEILELVERIAKELVEKLQKRISDEKLQEEVEQQIRKIEQLFAEVVGNLEHHHHHH	yes	yes
**rosetta3**	3	MSDELRELQRENNIEKAWELFRQNNKEEAEKILEEIYQIIRQGSEWLRLRILQEINLIDAAWELFRQNNKEFA	no	N/A

		KTILEIIRKIIEQGSEWLRLRILEQINLIDAAWQLFRENNKEFAKIILEIIREIIEKGSEWLRLRILKVINLQDAAEELKRQNNEEFAKIIEEILQQIIEKGSEELREKIKKESGSWLEHHHHHH		
**rosetta4**	3	MDEQRAVWRELEKRREELKKVLQERDIEQVVRVIQELLRRFNLDEQRAVWIILEVLRELLKWVLQEYDIEQVVRVIQTLLRTFNLDEQRAVWIILEVLRELLKWVLQYYDIEQVVRVIQTLLRTFNLDEQRAVWIILEVLRELLKWVLQYYDIEQVVRVIQHLLRSFNLDEQRAKEIIQEVLRELEKWVRQYEDGSWLEHHHHHH	no	N/A
**rosetta5**	4	MSEELREKIERDGDEDLREIIERAEKYEKRGNDWAVRQIEKLVEKILKLLEQIRRQGDEDLEEIIKRAIKYVKRGNYWAVEEILELVKKILELKLAIEAQGDEDLKEIIQRAIEYVKQGNSEAVREILKLVEKILQLKLAIEAQGDEELKKEIQKAIEYVKQGNSEAVKRILKEVEKTLREKLKKQASGSWLEHHHHHH	yes	no
**rosetta6**	4	MDELALRRELRDPEEHEKIREDTSRVLQQLEEAVQARDKRALRILLRDPLIHELIREDTSIVLIVLEIAVQARDVEALRILLRDPLIHELIREDTSIVEIVLEIAVQARDVLALRILLRDPLIHELIREDTSIVHIVLEIARQARDVLALRILRRDPLIHELEREDTGSWLEHHHHHH	yes	no
**rosetta7**	5	MDEEVKEVLEKLKDPEEFRRELEEIQKDSAAIIRVIKRWEKIFEVSKEEFVKVFLEVAKTPELFALILEVIRKNSAAIILVIHILENIFKVNKEEFVKVILEVLKTPYLFALLLEVIRKNSAAIHLVIRILENIFKVNKEEFVKVILEVLKTPYLWALLEEVNKKNDQAQKLARHIEENIEEVNKEAFEEVRKKVGSWLEHHHHHH	yes	no
**rosetta8**	5	MNQEVERLEEELEKQGIDEQQLRRIRKLLQRLAALGNQEVVRLILELVEEGIDEQQLRRIVKLLLELAALGNQEVVRLILELVQEGIDEQQLRRIVKLLRRLAALGNQEVVRLILVLVRDGIDEQQLRRSVKKLEEQAAHGNQEEVREELVKEISGSWLEHHHHHH	yes	no
**rosetta9**	6	MDSEKEHSRLRKDRERTSEQEIKEVLERILWEAVAERDSELLHSILRLIREITSEQEIKEVLERILQLAVALRDSELLHSILRLIREITSEQEIKEVLERILEDAVALRDSELLYSILILIWLITSEQEIKEVLERIREQAEALRDSELEHSIKILKKLITSGSWLEHHHHHH	no	N/A
**rosetta10**	8	MNQEAEIRKARETLRRIQKTWERGNQEEAIERLLELLIRLIVGGNQEALIEVAEILLELIQKIWERGNQEEAIEILLELLIVLIVGGNQEALIRVAQILLELIQKIWERGNQEEAIEILLELLEVLSVGGNQEALIIVALILLLLIQKIWERGNQEEAHEILEELEEVLREGGNQQAQRIVALIQQLLEQKIRERGNQGSWLEHHHHHH	no	N/A

Supplementary Table 5: Data collection and refinement statistics (molecular replacement).

	C_2HR1_4r (PDB: 8EOV)	C_3HR3_4r (PDB: 8EOZ)	C_4HR1_4r (PDB: 8EOX)	C_2HR4_8r (PDB: 8ERW)
**Data collection**				
Space group	*P*3_2*21*	*R3:H*	*P*2_1	*P*$2_12_12_1$
Cell dimensions				
a, *b*, *c* (Å)	49.14, 49.14, 111.21	68.22, 68.22, 139.03	62.75, 80.36, 66.70	55.31, 55.38, 145.13
α, β, γ (°)	90, 90, 120	90, 90, 120	90, 112.75, 90	90, 90, 90
Resolution (Å)	42.56 - 1.59 (1.64 -1.59)*	46.35 - 3.00 (3.10 - 3.00)*	53.79 - 3.3 (3.41 - 3.30)*	37.79 - 2.88 (2.98 - 2.88)*
R_{merge}	0.012 (1.038)	0.040 (0.689)	0.035 (0.700)	0.115 (0.632)
$I / \sigma I$	33.39 (0.68)	10.95 (4.09)	7.22 (0.90)	7.87 (1.39)
Completeness (%)	99.76 (99.67)	98.49 (99.90)	97.04 (98.69)	95.62 (86.48)
Redundancy	12.6 (13.1)	8.2 (6.7)	4.2 (4.3)	4.4 (3.5)
**Refinement**				
Resolution (Å)	42.56 - 1.59 (1.64 -1.59)*	46.35 - 3.00 (3.10 - 3.00)*	53.79 - 3.3 (3.41 - 3.30)	37.79 - 2.88 (2.98 - 2.88)
No. reflections	21592 (2113)	4527 (328)	9035 (903)	10135 (876)
R_{work} / R_{free}	0.2023/ 0.2244	0.2343/ 0.2902	0.2947/ 0.3516)	0.2611/ 0.2862
No. atoms				
Protein	1254	1874	5619	3157
Ligand/ion	25	0	0	0
Water	42	0	0	9
B-factors				
Protein	45.23	36.21	135.76	79.93
Ligand/ion	76.41	0	0	0
Water	52.73	0	0	71.98
R.m.s. deviations				
Bond lengths (Å)	0.006	0.002	0.002	0.002
Bond angles (°)	0.620	0.430	0.450	0.380

*Single Crystal used for each data collection. *Values in parentheses are for the highest-resolution shell.

Supplementary Table 6. CryoEM Data Collection Statistics for Bounded Designs

	C_5HR2_4r	C_3HR3_8r	C_4HR1_4r	C_6HR1_4r	C_6HR1_8r
Microscope	Glacios	Glacios	Glacios	Glacios	Glacios
Voltage (kV)	200	200	200	200	200
Detector	Gatan K3 Summit	Gatan K3 Summit	Gatan K3 Summit	Gatan K3 Summit	Gatan K3 Summit
Recording mode	Counting	Counting	Counting	Counting	Counting
Magnification	45,000x	45,000x	45,000x	45,000x	45,000x
Movie micrograph pixel size (Å)	0.4425	0.4425	0.4425	0.4425	0.4425
Dose rate (e^{-}/Å^2/s)	10	10	10	10	10
No. of frames per movie micrograph	99	99	99	99	99
Frame exposure time (ms)	0.0505	0.0505	0.0505	0.0505	0.0505
Movie micrograph exposure time (s)	5.0	5.0	5.0	5.0	5.0
Total dose (e^{-}/Å^2)	50	50	50	50	50
Under focus range (μm)	1.0 - 2.0	1.0 - 2.0	1.0 - 2.0	1.0 - 2.0	0.7-1.8
Number of movie micrographs	306	1810	1146	694	1468

Supplementary Table 7. CryoEM Data Collection Statistics for Unbounded Designs.

	C_3HR3_9r_shift4	C_4HR1_8r_shift5
Microscope	Glacios	Glacios
Voltage (kV)	200	200
Detector	Gatan K3 Summit	Gatan K3 Summit
Recording mode	Counting	Counting
Magnification	45,000x	45,000x
Movie micrograph pixel size (Å)	0.4425	0.4425
Dose rate (e^-/Å^2/s)	10	10
No. of frames per movie micrograph	99	99
Frame exposure time (ms)	0.0505	0.0505
Movie micrograph exposure time (s)	5.0	5.0
Total dose (e^-/Å^2)	50	50
Under focus range (μm)	0.7 - 1.8	0.7 - 1.8
Number of movie micrographs	916	656
