# Supplementary figures and images for: Precisely patterned nanofibres made from extendable protein multiplexes (part 1 of 2)
Source: Nat Chem. 2023 Sep 4;15(12):1664–71. doi: 10.1038/s41557-023-01314-x (PMC10695826; doi:10.1038/s41557-023-01314-x)

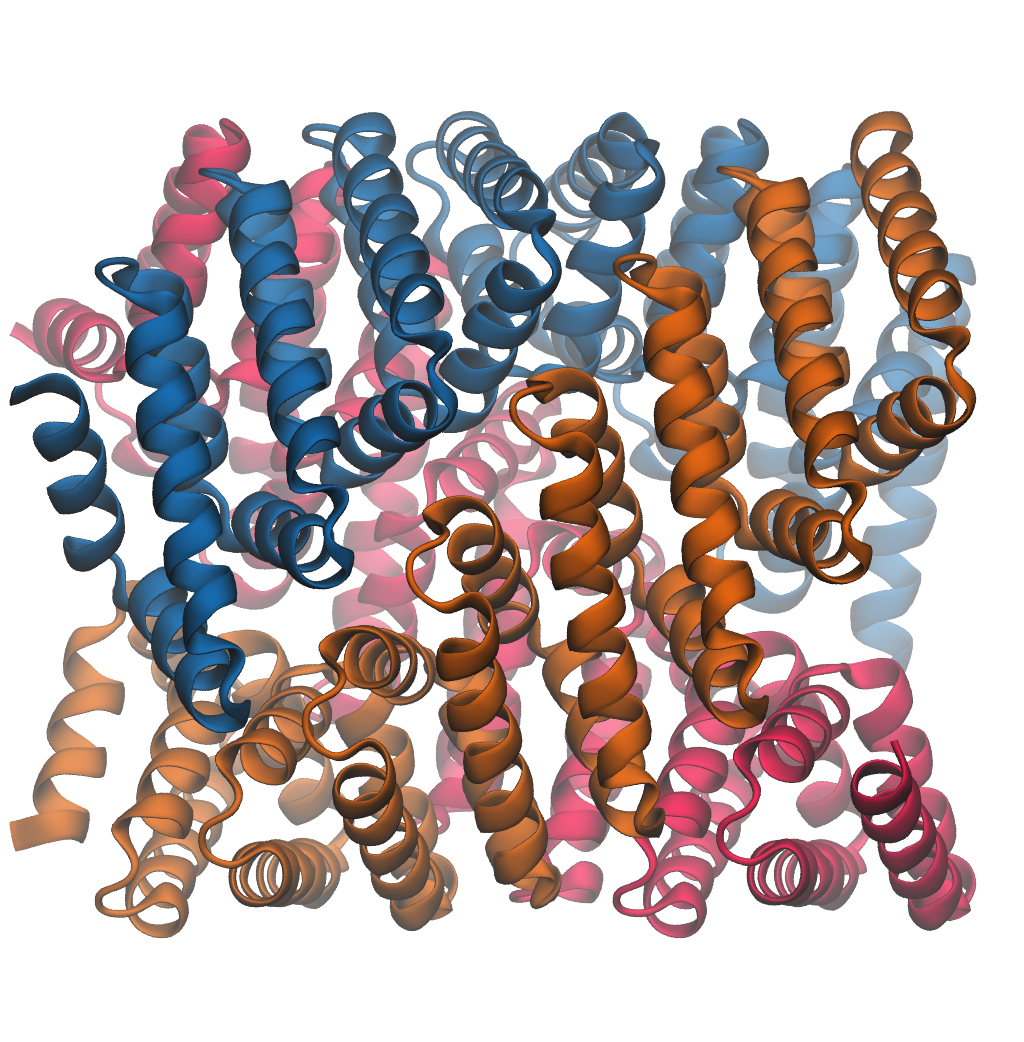

Supplement: Supplementary file 2 — Protein model images. [file 41557_2023_1314_MOESM2_ESM.zip › Figure1/8rO.jpg]

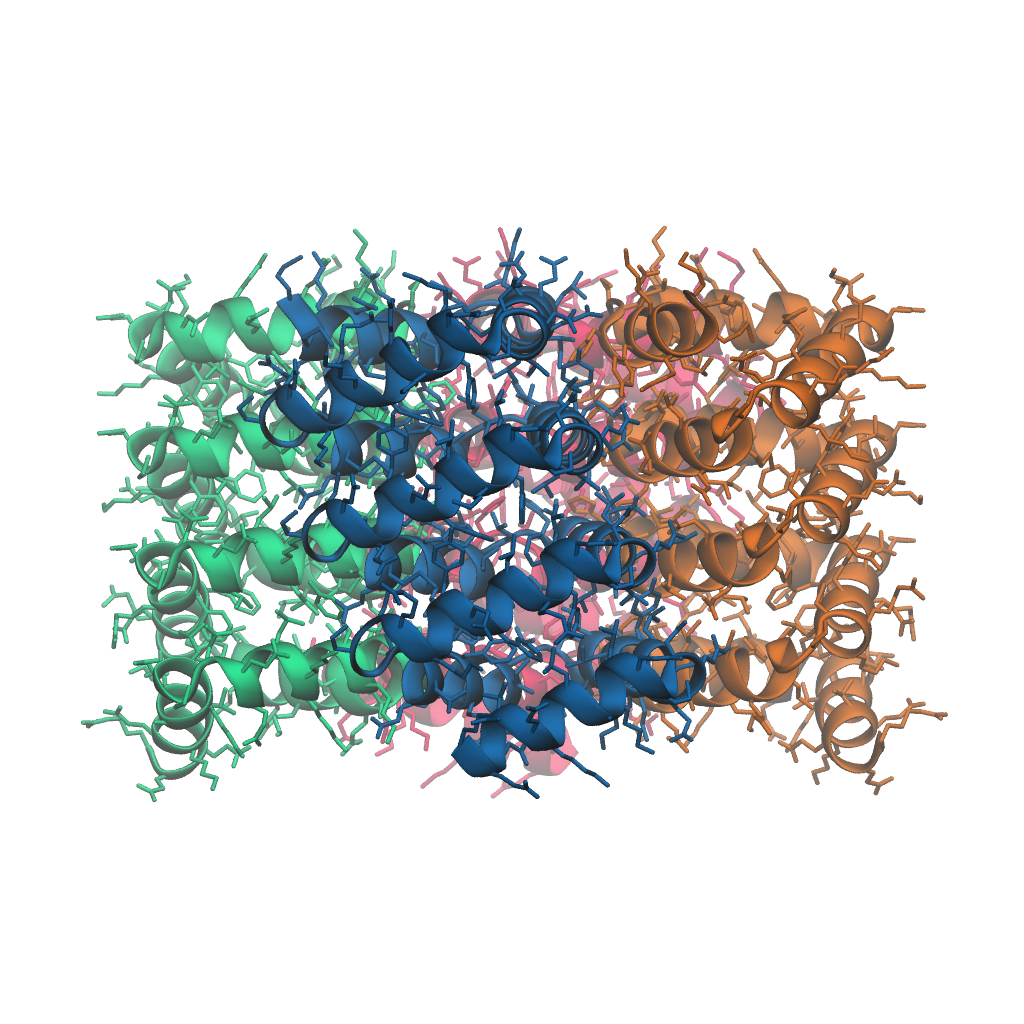

Supplement: Supplementary file 2 — Protein model images. [file 41557_2023_1314_MOESM2_ESM.zip › Figure1/3o22_SC.jpg]

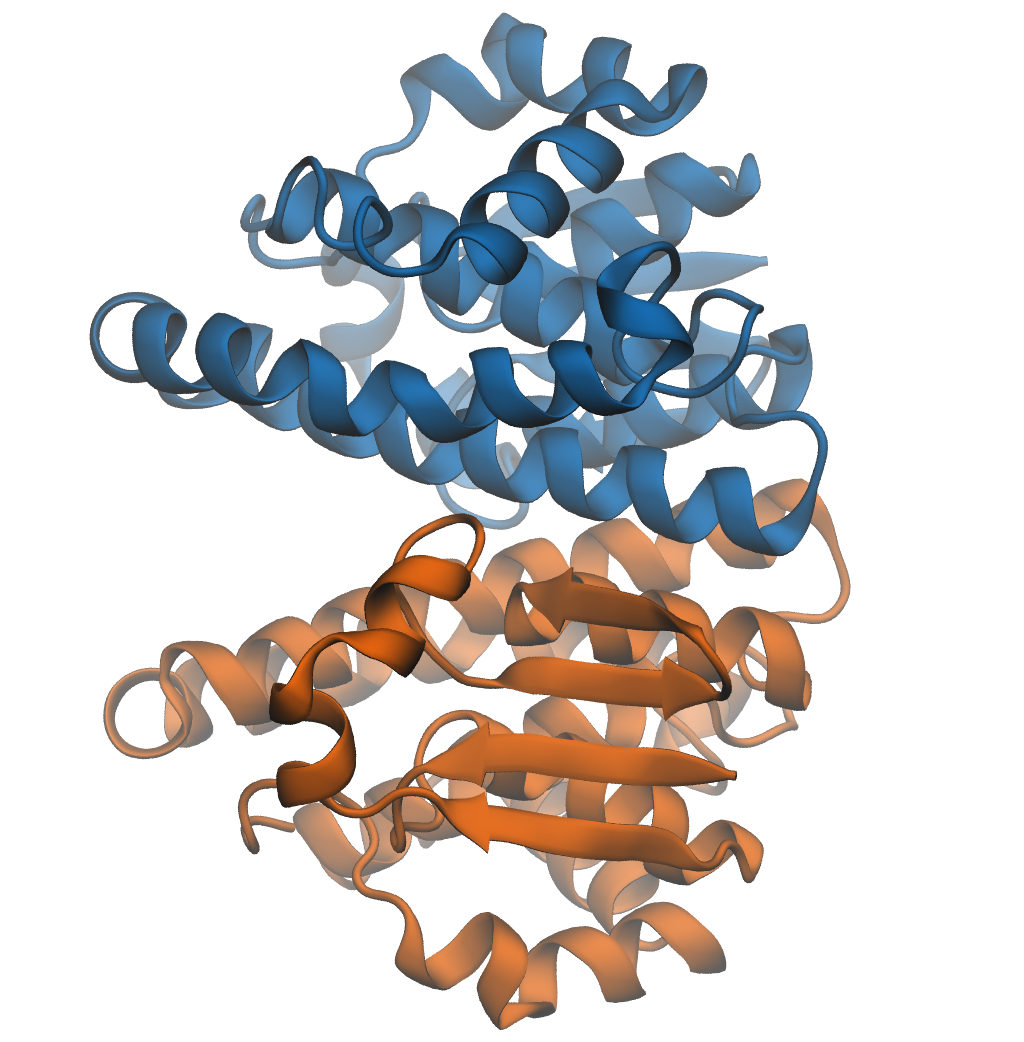

Supplement: Supplementary file 2 — Protein model images. [file 41557_2023_1314_MOESM2_ESM.zip › Figure1/A10GS.jpg]

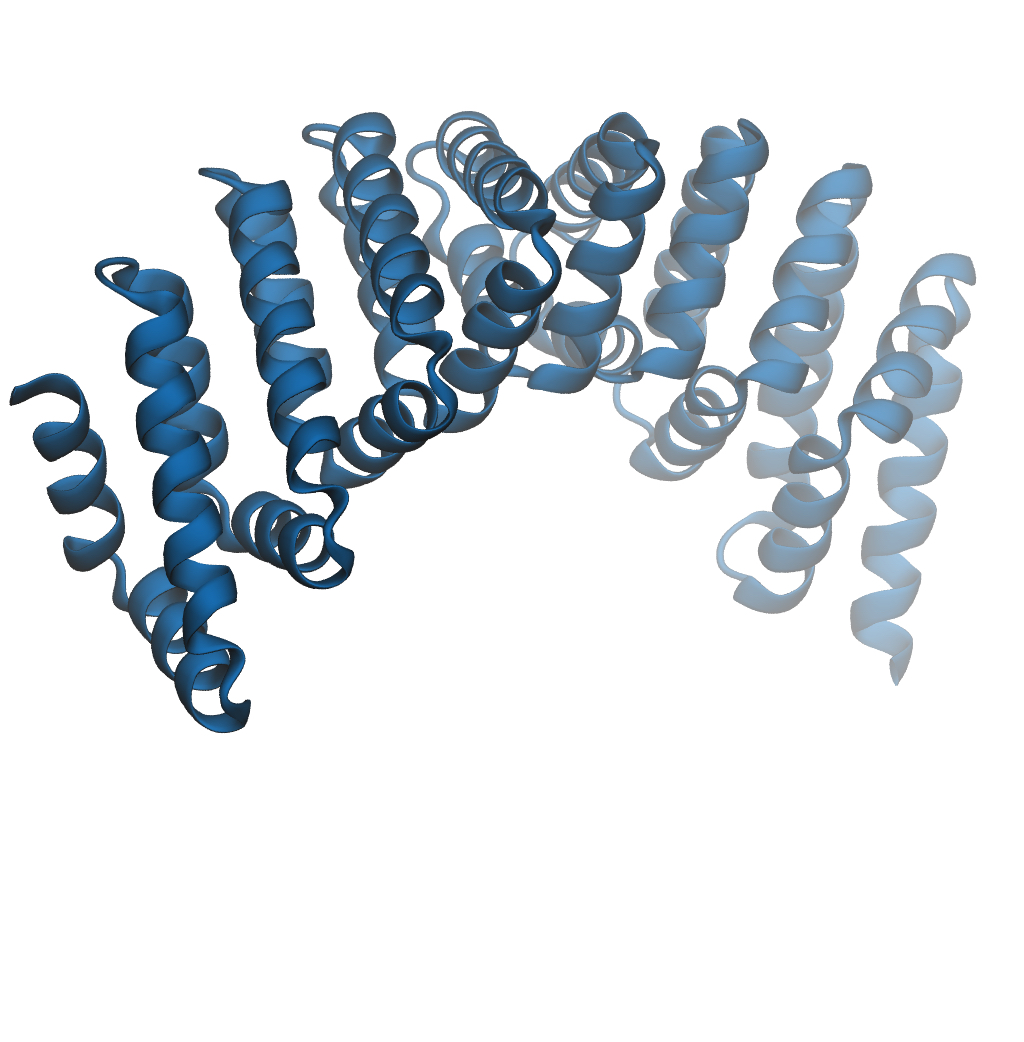

Supplement: Supplementary file 2 — Protein model images. [file 41557_2023_1314_MOESM2_ESM.zip › Figure1/8rM.jpg]

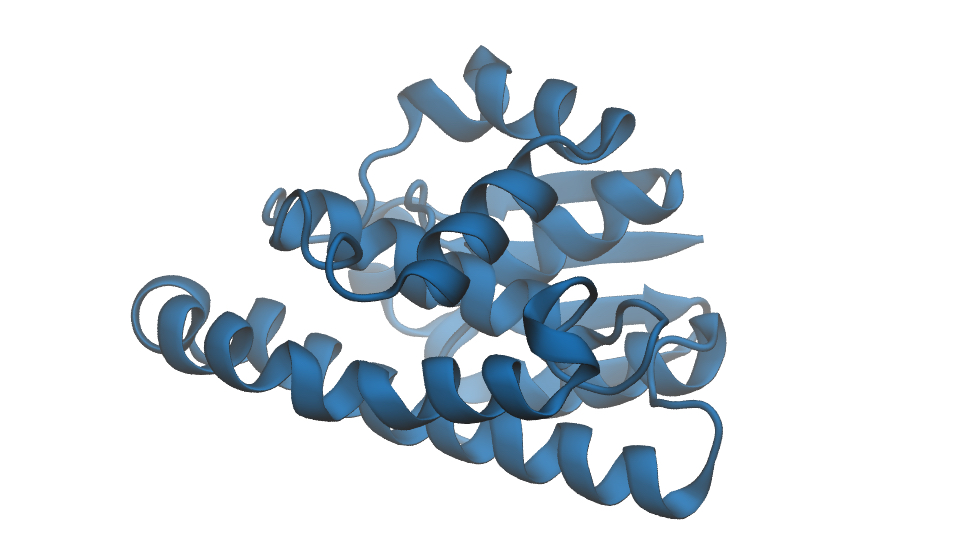

Supplement: Supplementary file 2 — Protein model images. [file 41557_2023_1314_MOESM2_ESM.zip › Figure1/10gs_monomer.jpg]

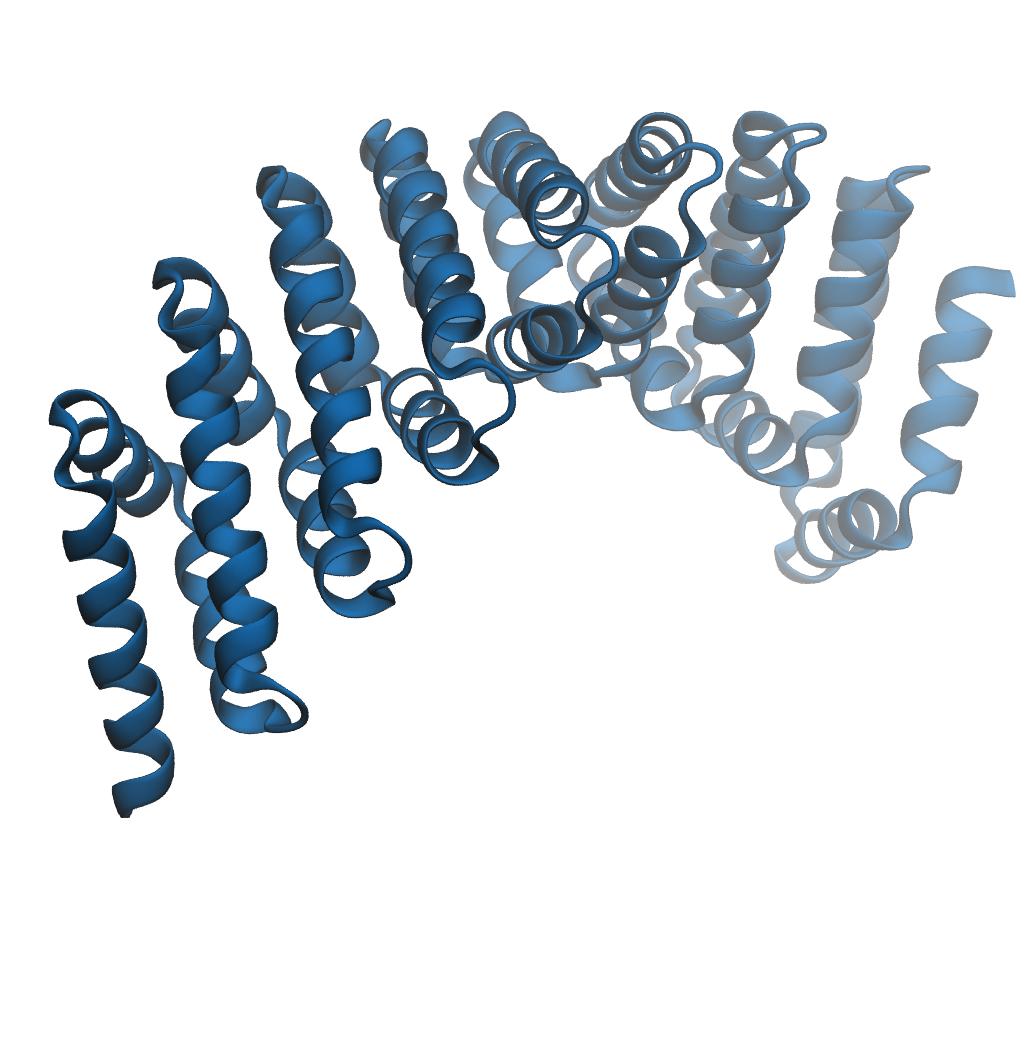

Supplement: Supplementary file 2 — Protein model images. [file 41557_2023_1314_MOESM2_ESM.zip › Figure1/8rMb.jpg]

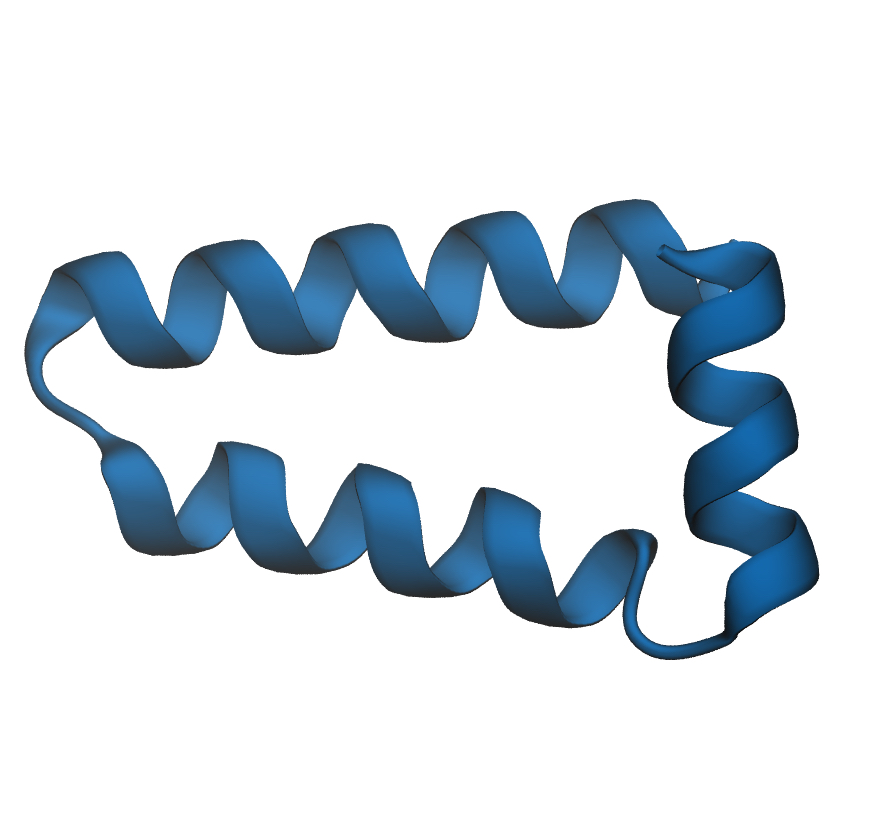

Supplement: Supplementary file 2 — Protein model images. [file 41557_2023_1314_MOESM2_ESM.zip › Figure1/3H.jpg]

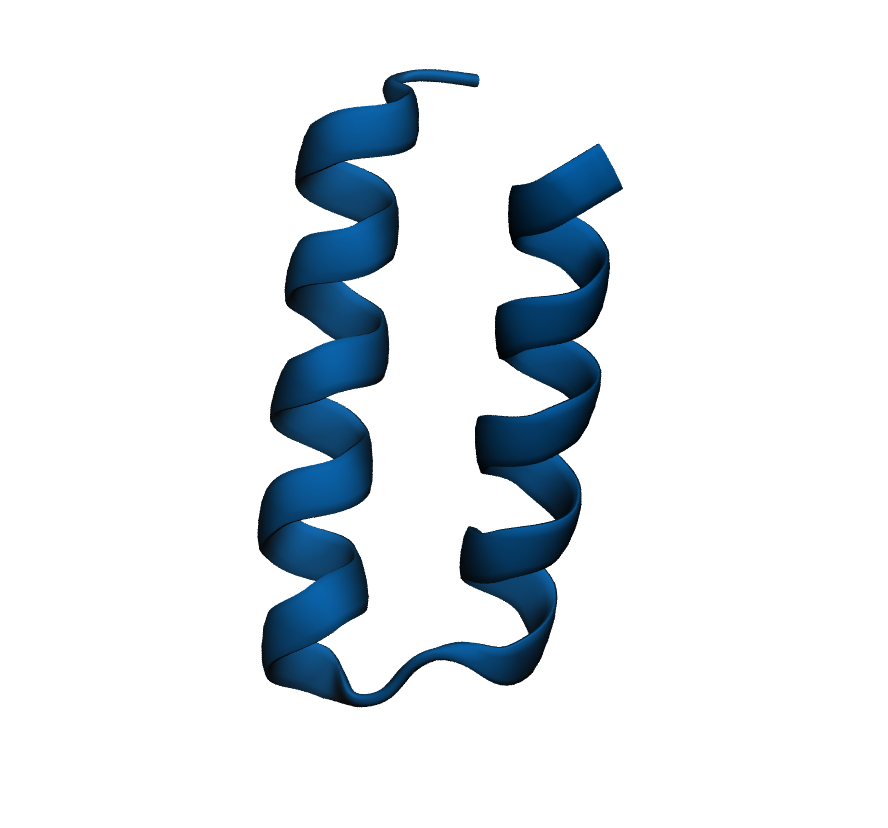

Supplement: Supplementary file 2 — Protein model images. [file 41557_2023_1314_MOESM2_ESM.zip › Figure1/2H.jpg]

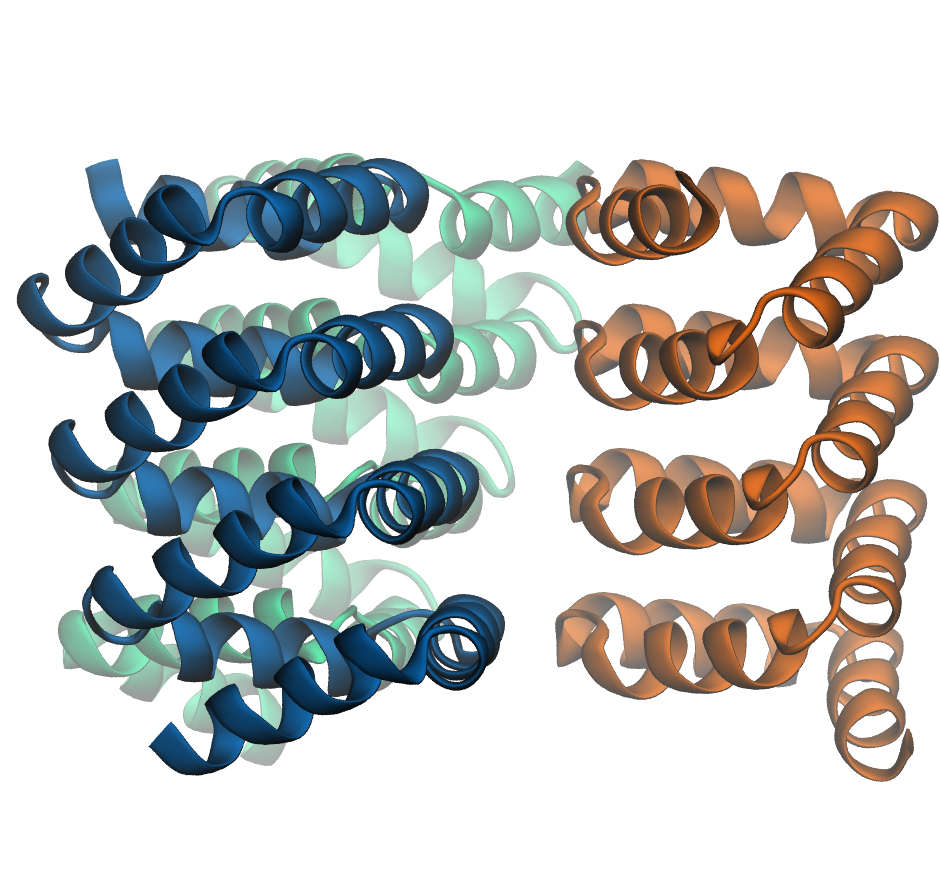

Supplement: Supplementary file 2 — Protein model images. [file 41557_2023_1314_MOESM2_ESM.zip › Figure1/3o22_C3.jpg]

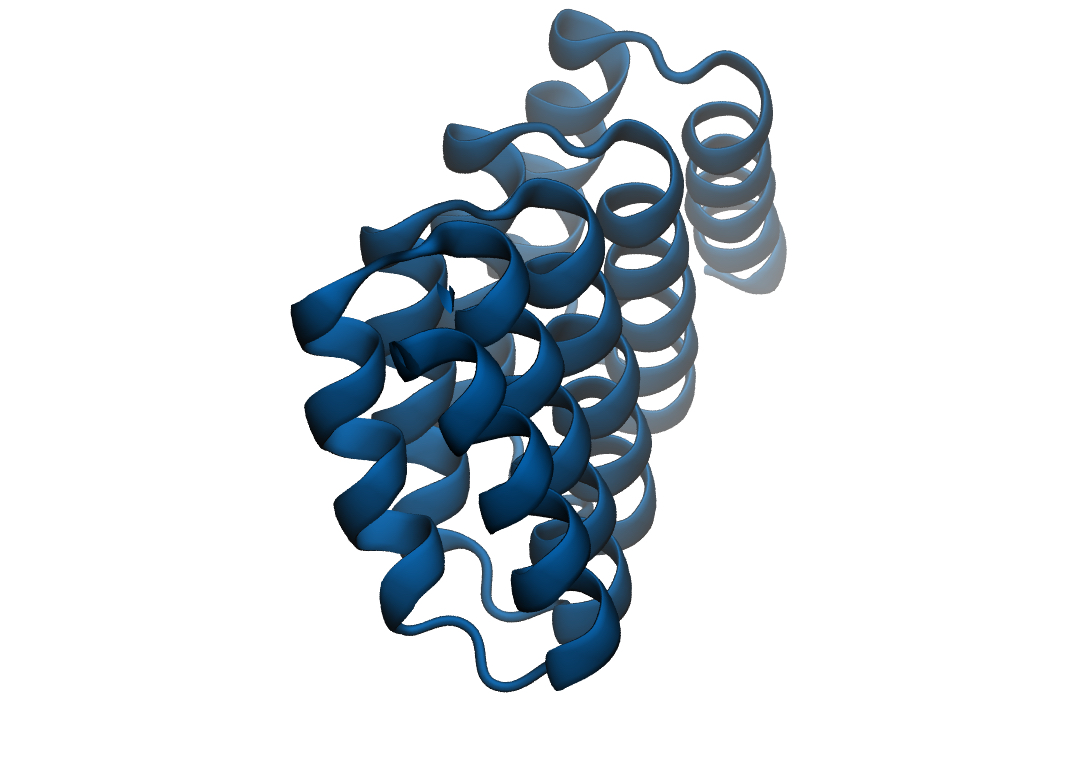

Supplement: Supplementary file 2 — Protein model images. [file 41557_2023_1314_MOESM2_ESM.zip › Figure1/chrG.jpg]

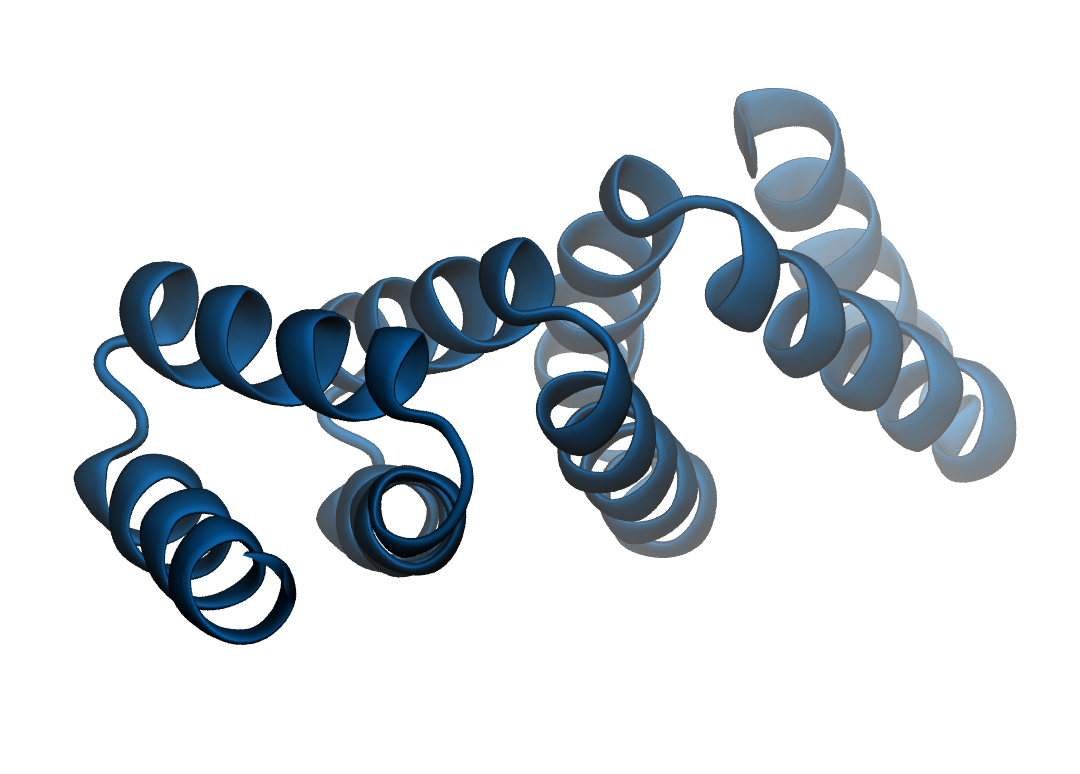

Supplement: Supplementary file 2 — Protein model images. [file 41557_2023_1314_MOESM2_ESM.zip › Figure1/chrF.jpg]

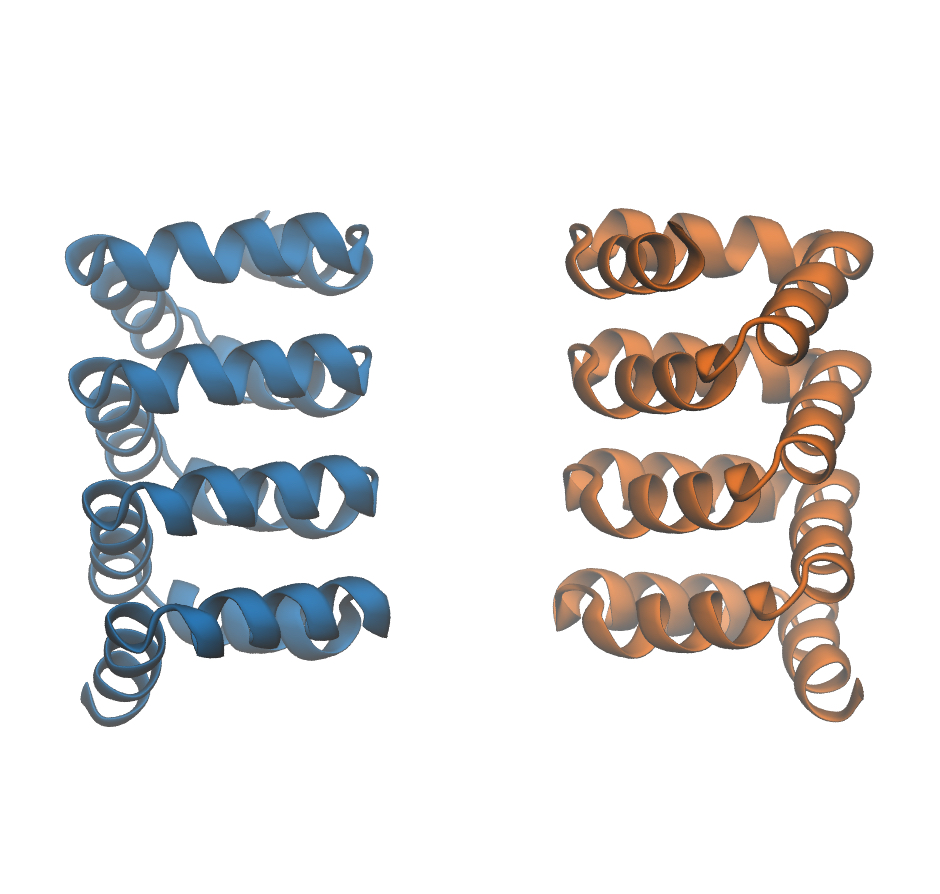

Supplement: Supplementary file 2 — Protein model images. [file 41557_2023_1314_MOESM2_ESM.zip › Figure1/3o22_C2.jpg]

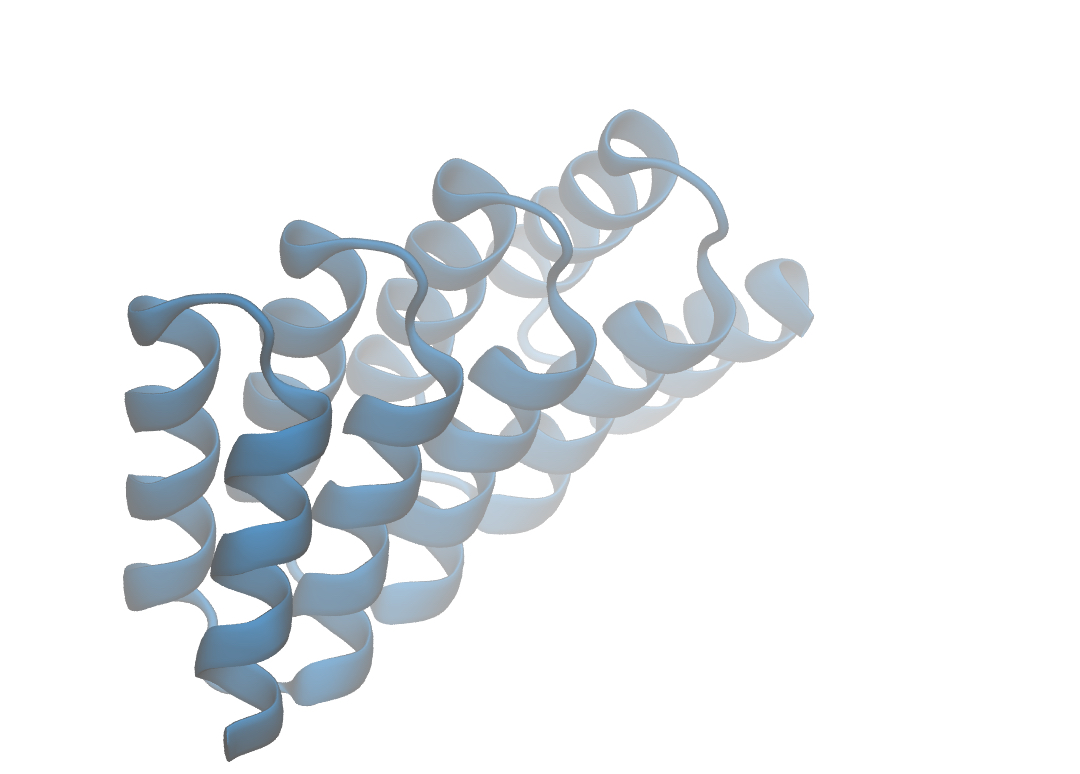

Supplement: Supplementary file 2 — Protein model images. [file 41557_2023_1314_MOESM2_ESM.zip › Figure1/chrD.jpg]

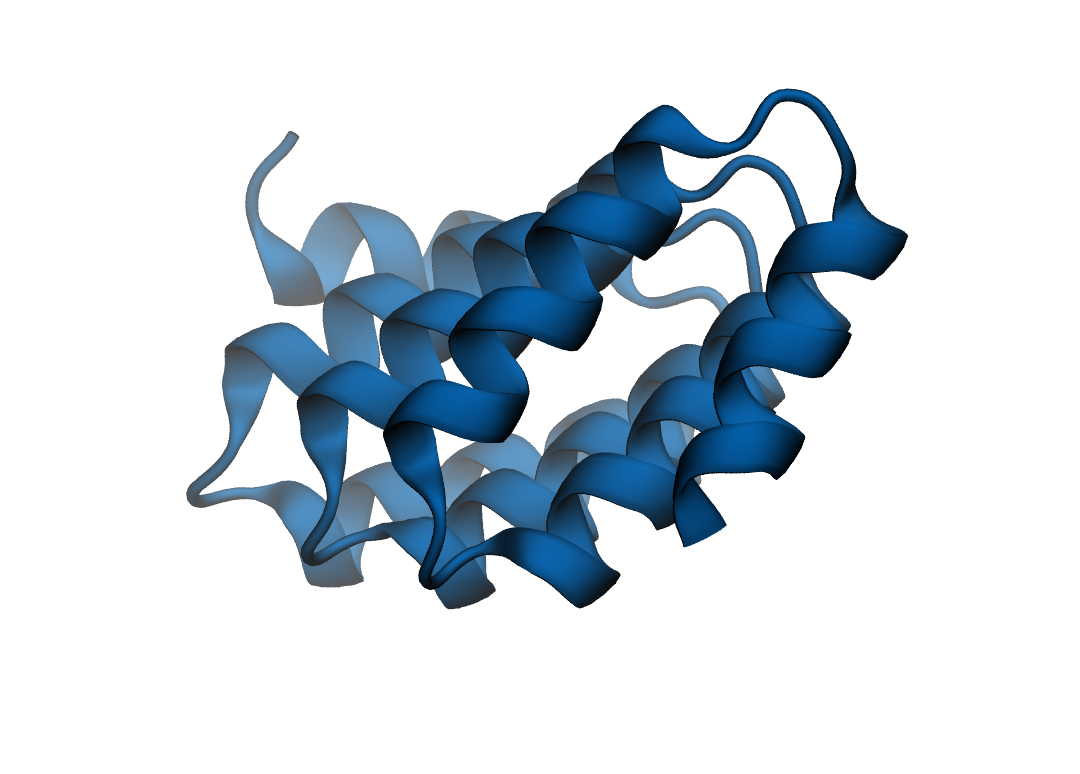

Supplement: Supplementary file 2 — Protein model images. [file 41557_2023_1314_MOESM2_ESM.zip › Figure1/chrE.jpg]

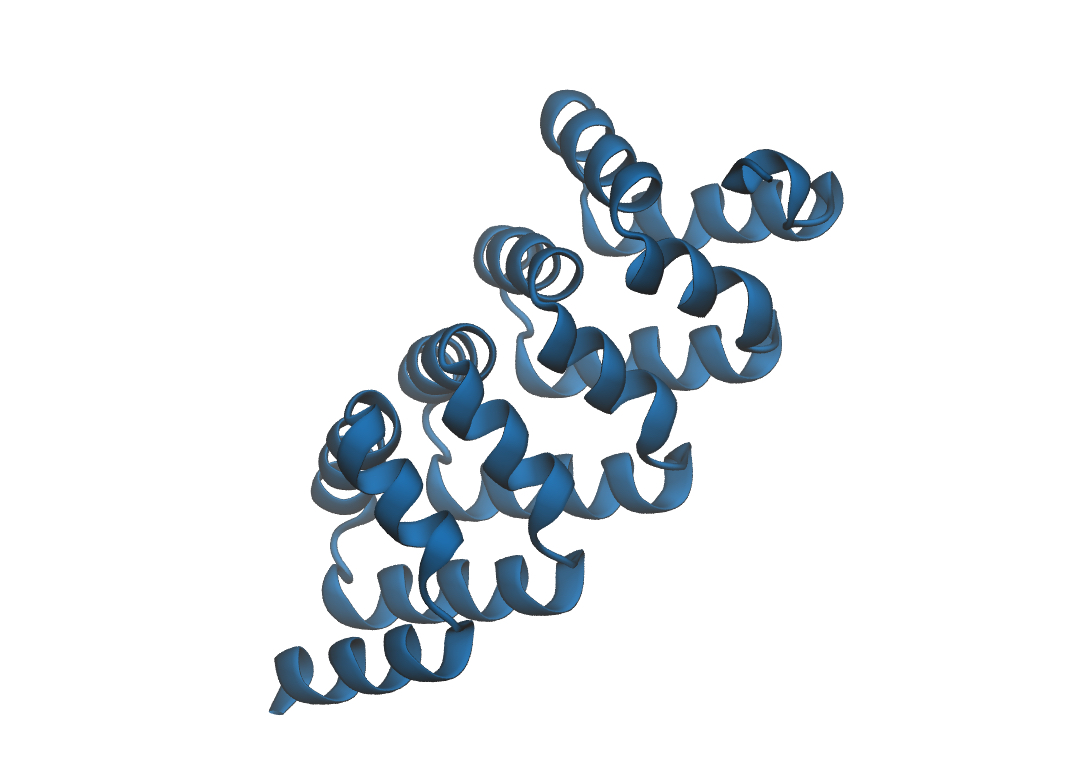

Supplement: Supplementary file 2 — Protein model images. [file 41557_2023_1314_MOESM2_ESM.zip › Figure1/chrA.jpg]

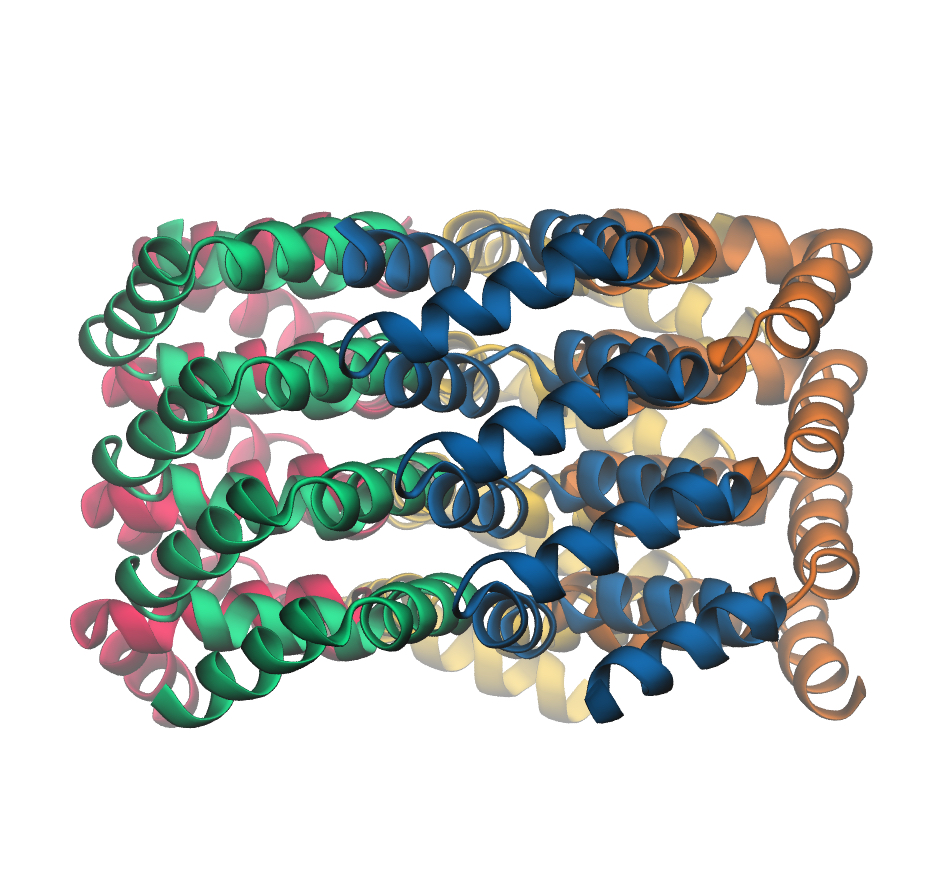

Supplement: Supplementary file 2 — Protein model images. [file 41557_2023_1314_MOESM2_ESM.zip › Figure1/3o22_C5.jpg]

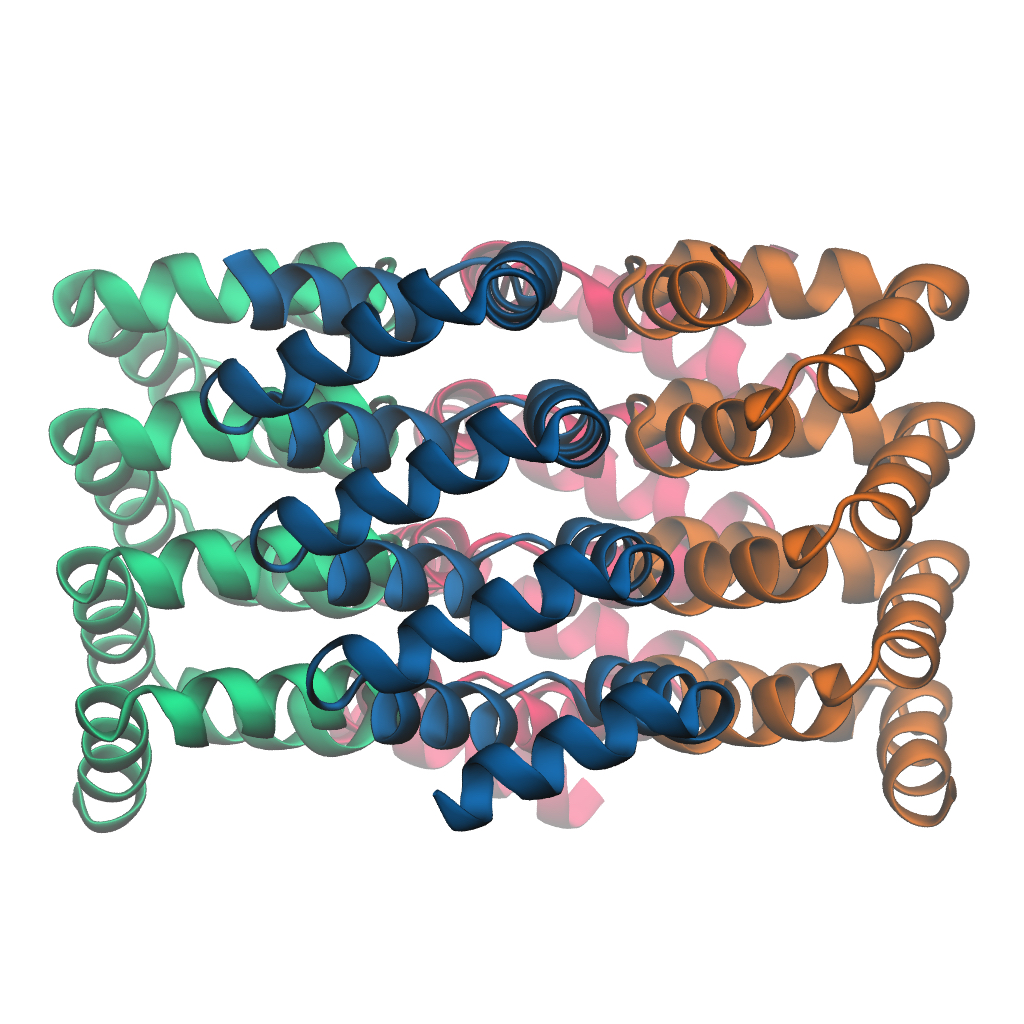

Supplement: Supplementary file 2 — Protein model images. [file 41557_2023_1314_MOESM2_ESM.zip › Figure1/3o22_C4.jpg]

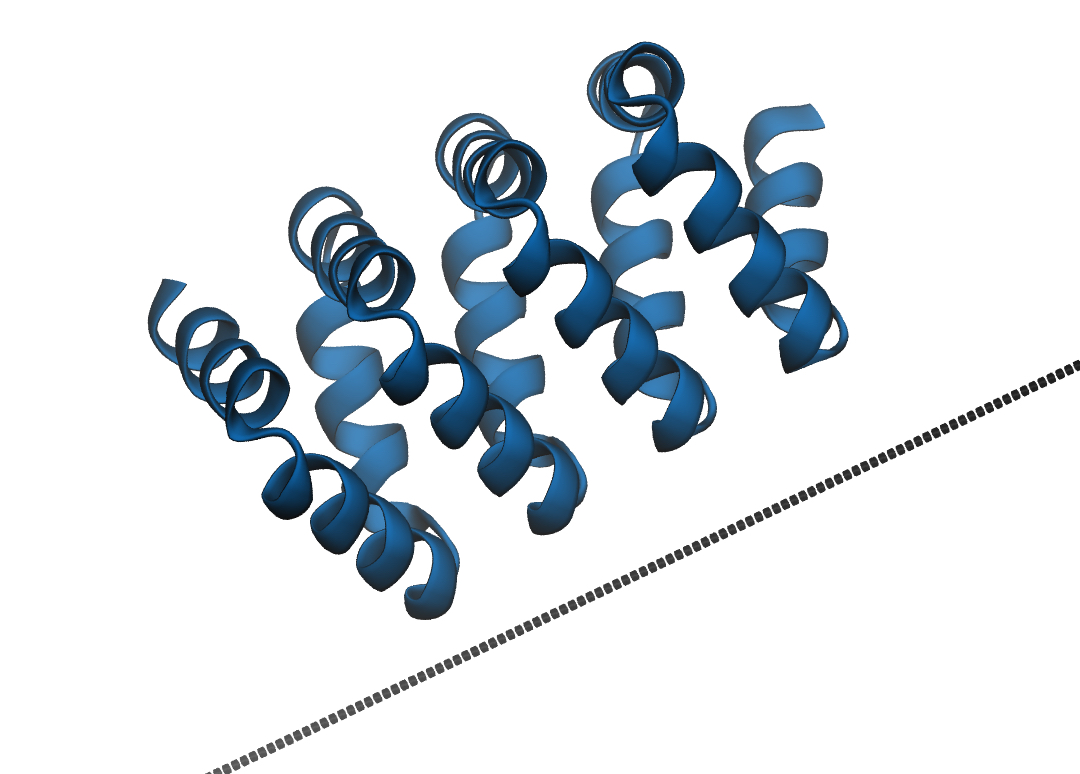

Supplement: Supplementary file 2 — Protein model images. [file 41557_2023_1314_MOESM2_ESM.zip › Figure1/3o22_helical.jpg]

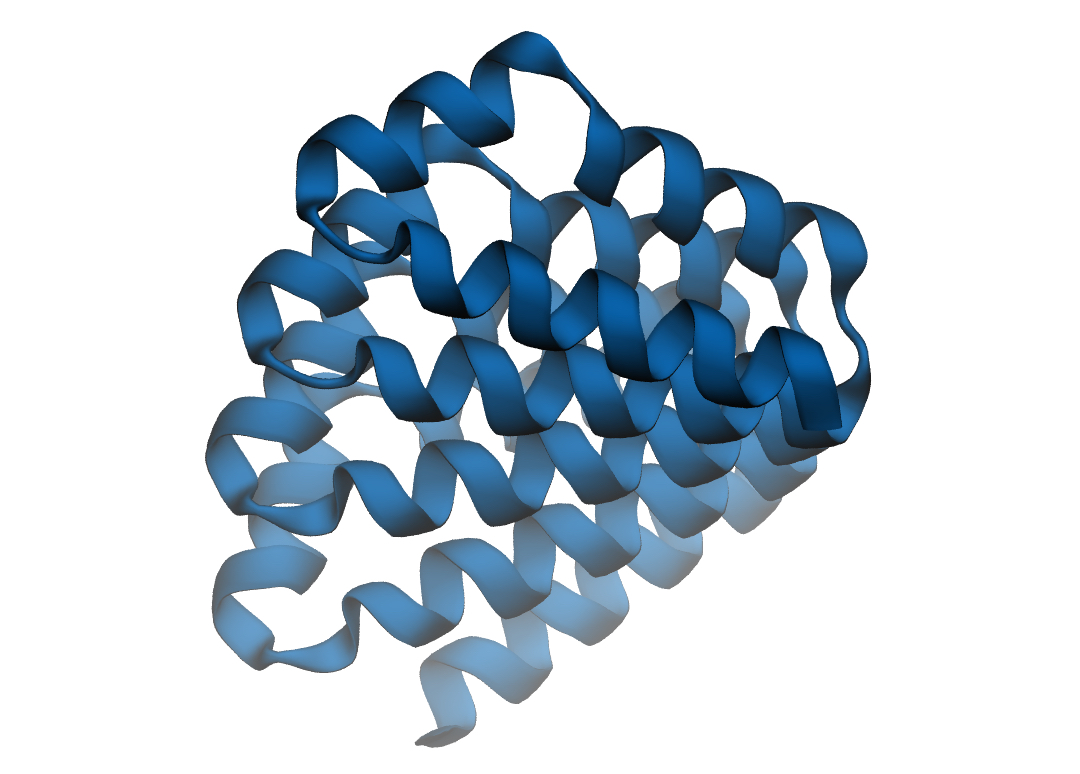

Supplement: Supplementary file 2 — Protein model images. [file 41557_2023_1314_MOESM2_ESM.zip › Figure1/chrB.jpg]

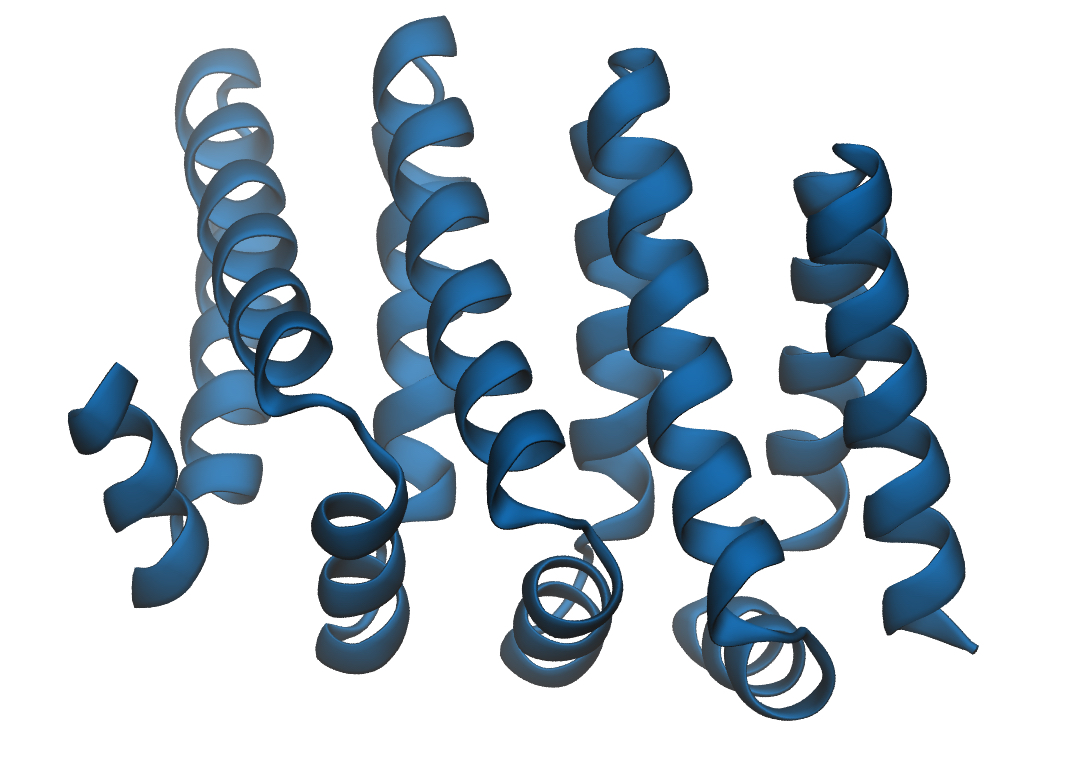

Supplement: Supplementary file 2 — Protein model images. [file 41557_2023_1314_MOESM2_ESM.zip › Figure1/chrC.jpg]

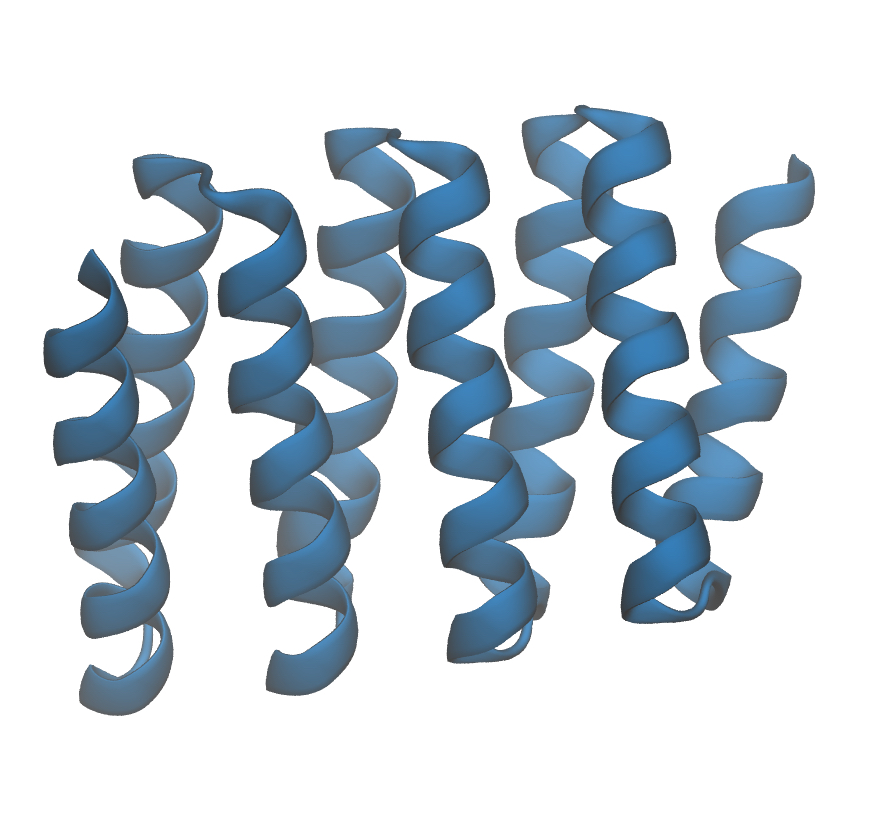

Supplement: Supplementary file 2 — Protein model images. [file 41557_2023_1314_MOESM2_ESM.zip › Figure1/repEx.jpg]

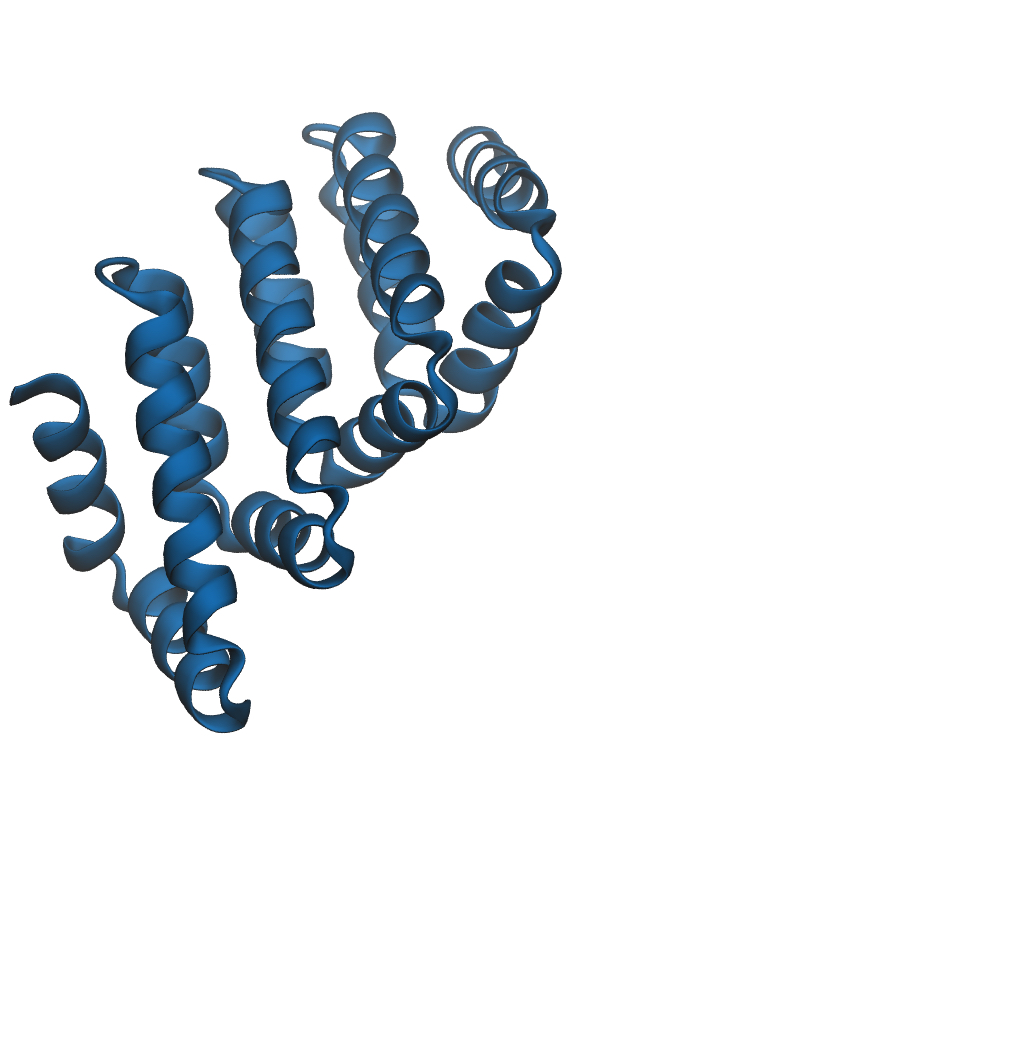

Supplement: Supplementary file 2 — Protein model images. [file 41557_2023_1314_MOESM2_ESM.zip › Figure1/4rM.jpg]

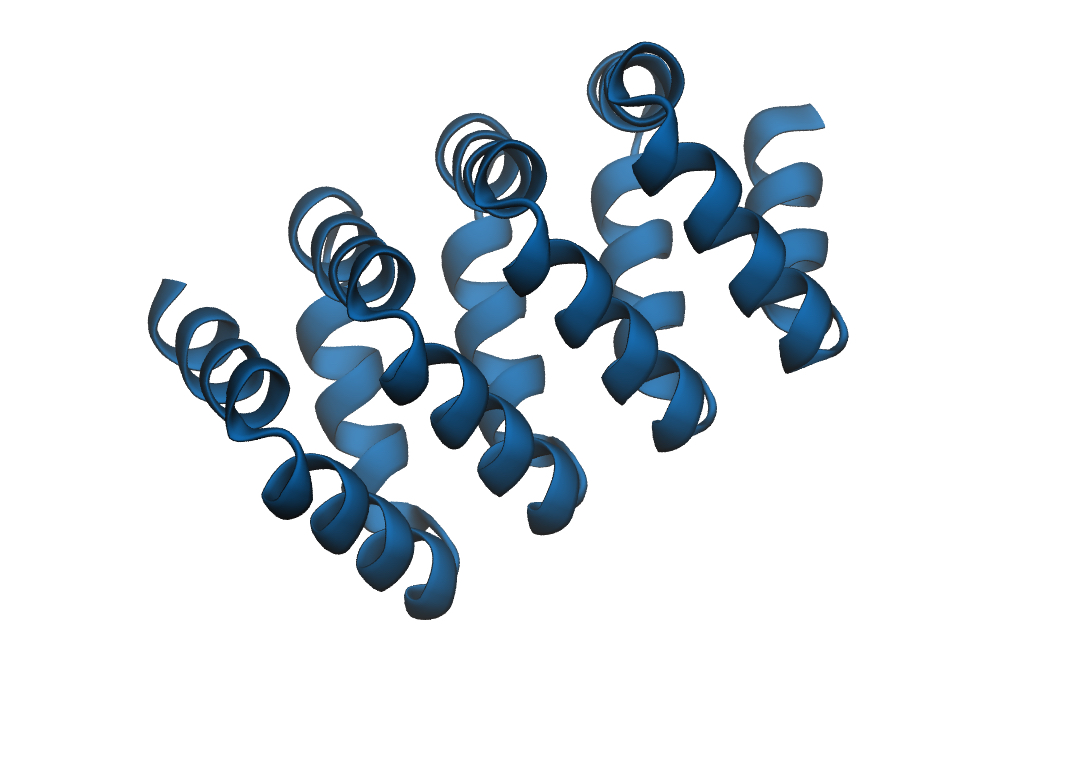

Supplement: Supplementary file 2 — Protein model images. [file 41557_2023_1314_MOESM2_ESM.zip › Figure1/3o22_mono.jpg]

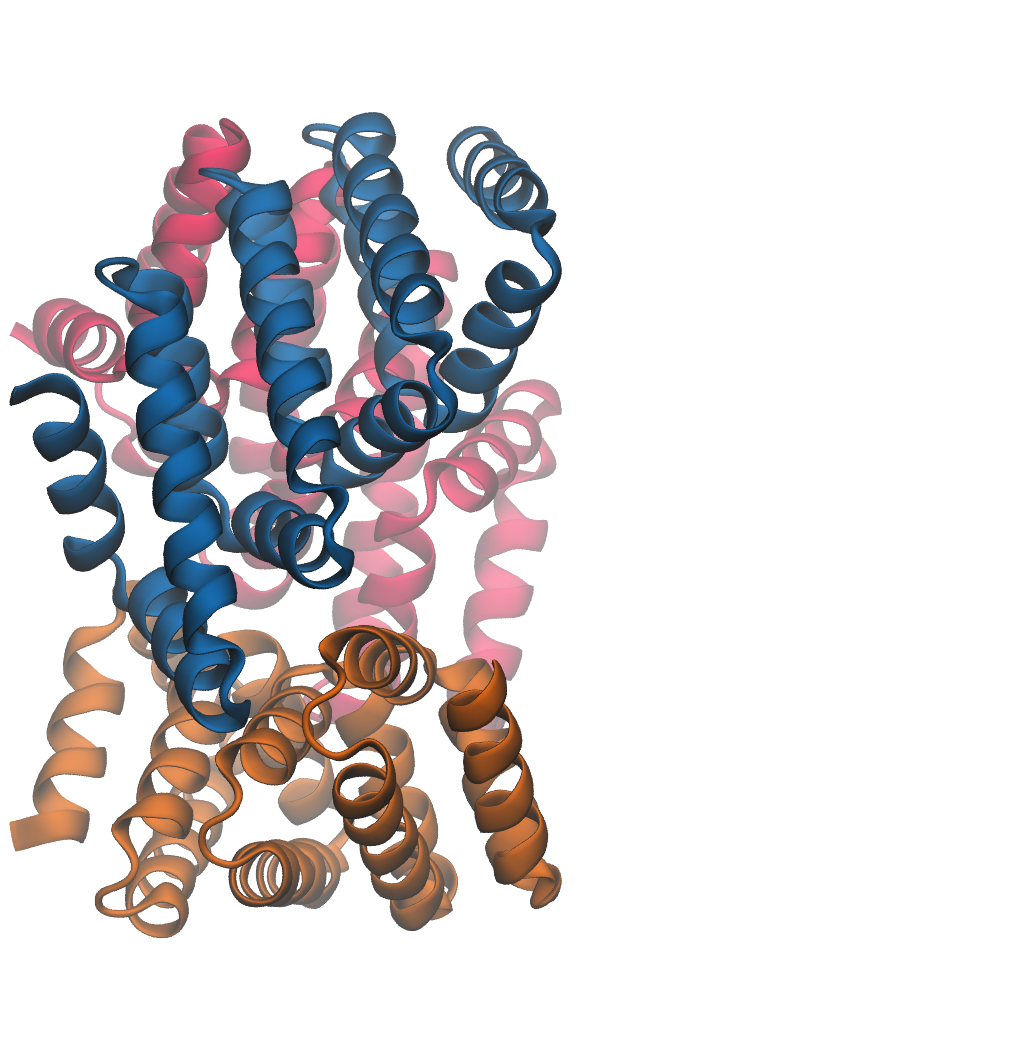

Supplement: Supplementary file 2 — Protein model images. [file 41557_2023_1314_MOESM2_ESM.zip › Figure1/4rO.jpg]

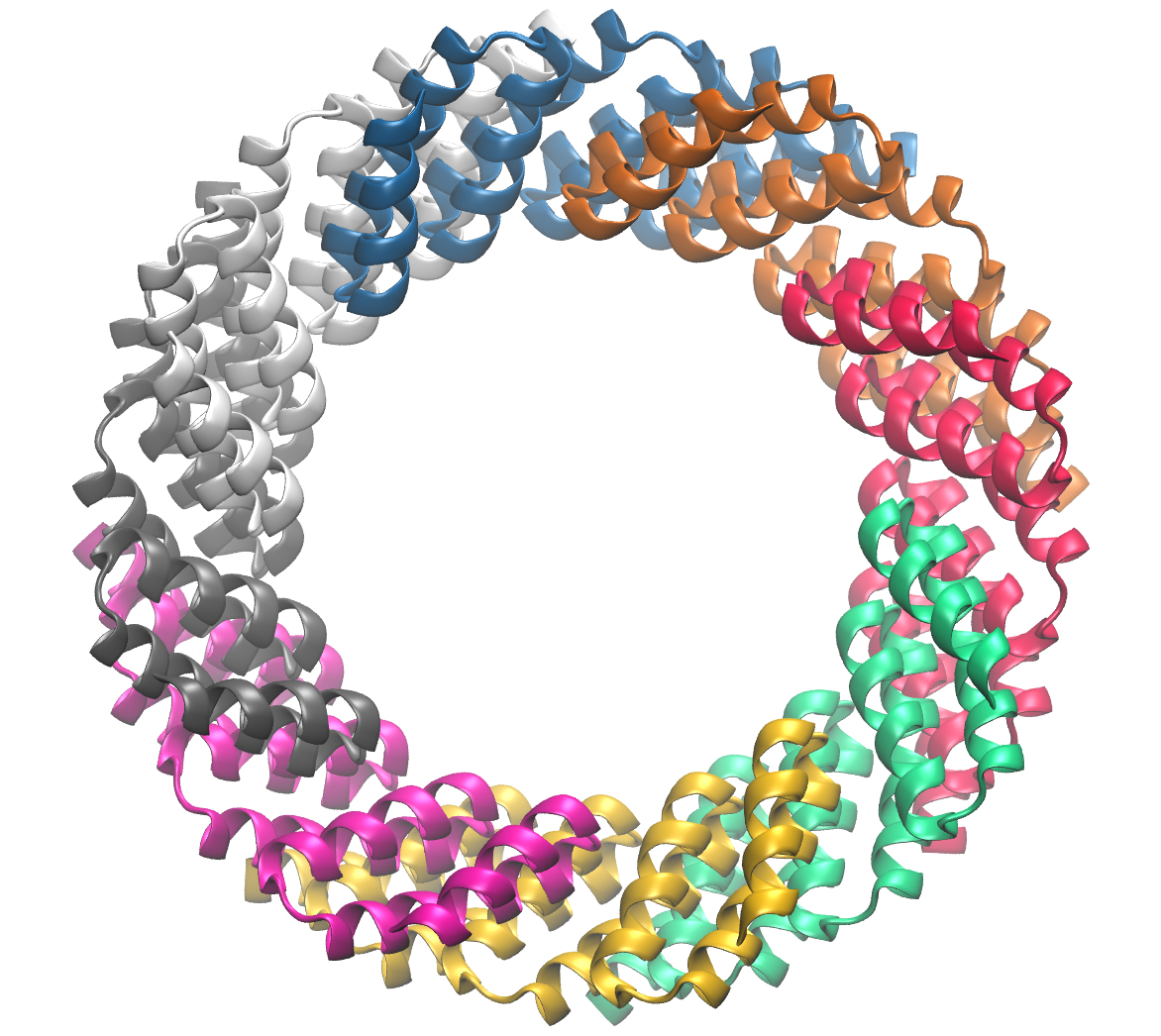

Supplement: Supplementary file 3 — Protein model images, SEC source data and SAXS source data. [file 41557_2023_1314_MOESM3_ESM.zip › Figure2/2o63.jpg]

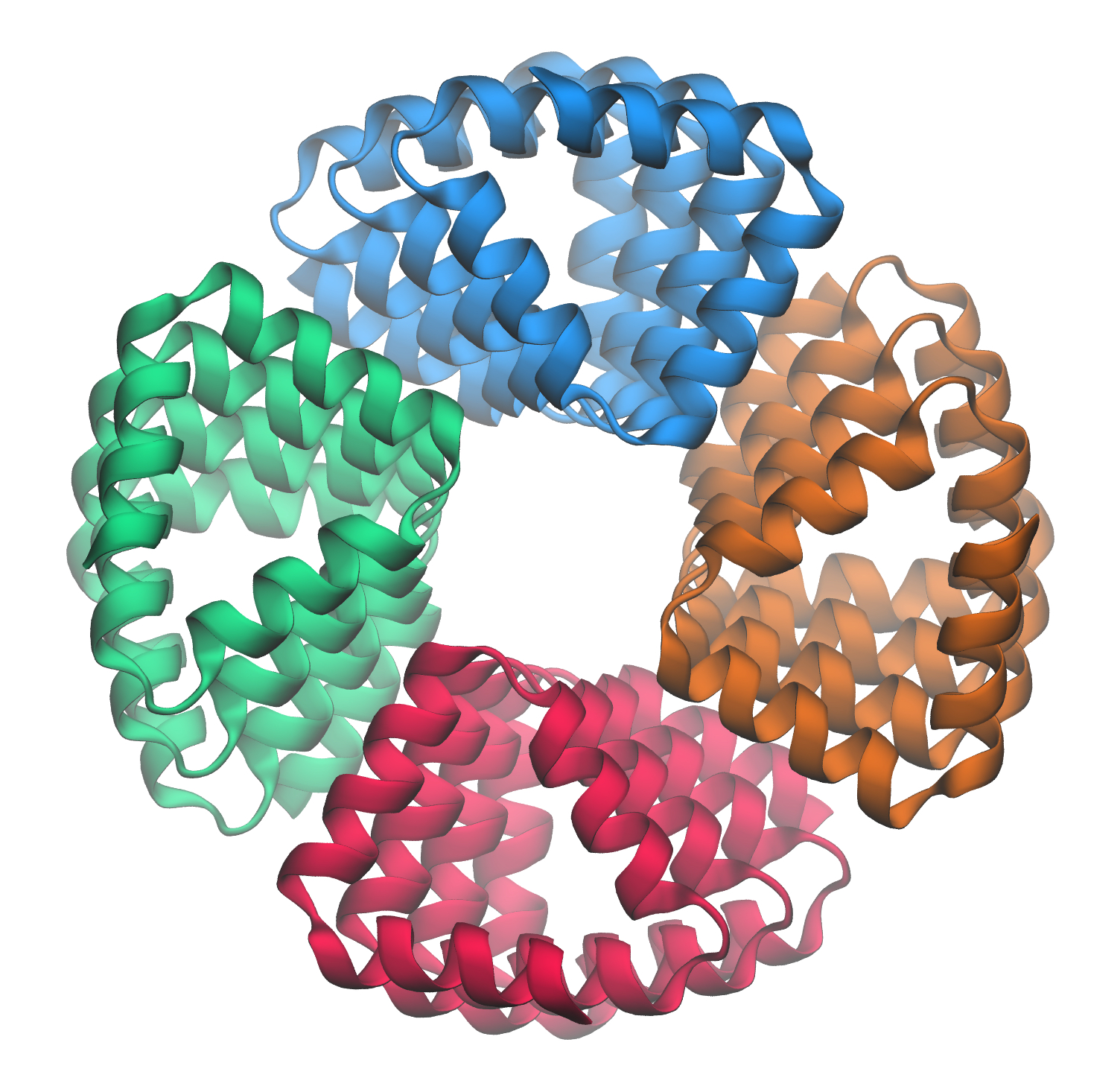

Supplement: Supplementary file 3 — Protein model images, SEC source data and SAXS source data. [file 41557_2023_1314_MOESM3_ESM.zip › Figure2/3o22.jpg]

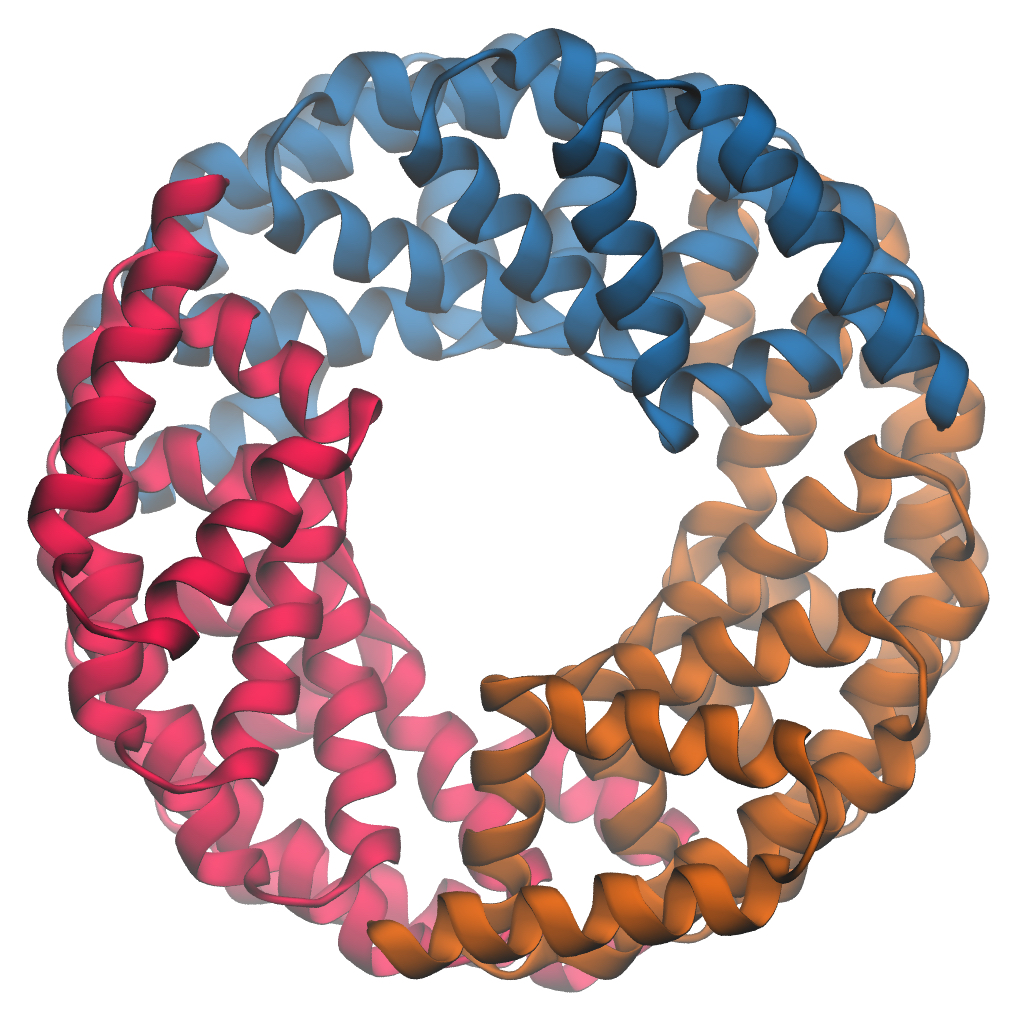

Supplement: Supplementary file 3 — Protein model images, SEC source data and SAXS source data. [file 41557_2023_1314_MOESM3_ESM.zip › Figure2/3o52.jpg]

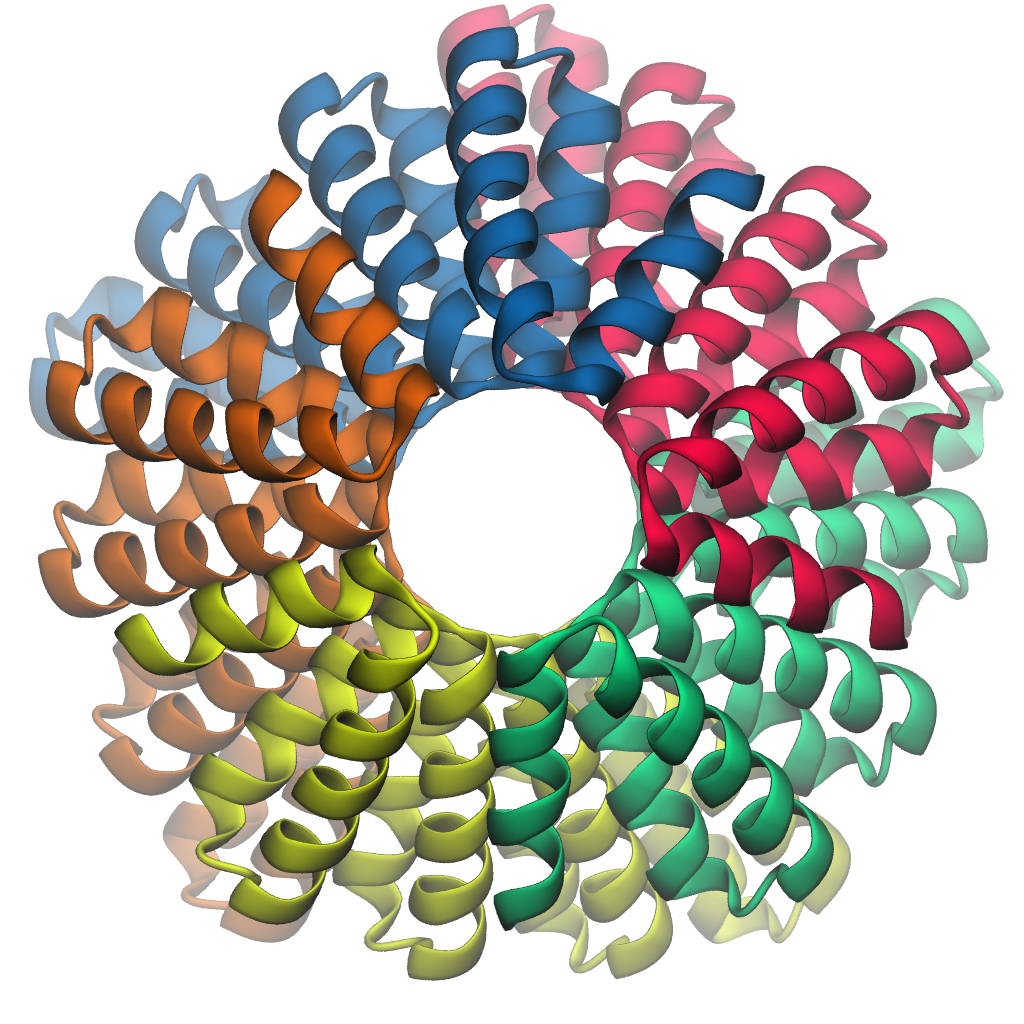

Supplement: Supplementary file 3 — Protein model images, SEC source data and SAXS source data. [file 41557_2023_1314_MOESM3_ESM.zip › Figure2/2o31.jpg]

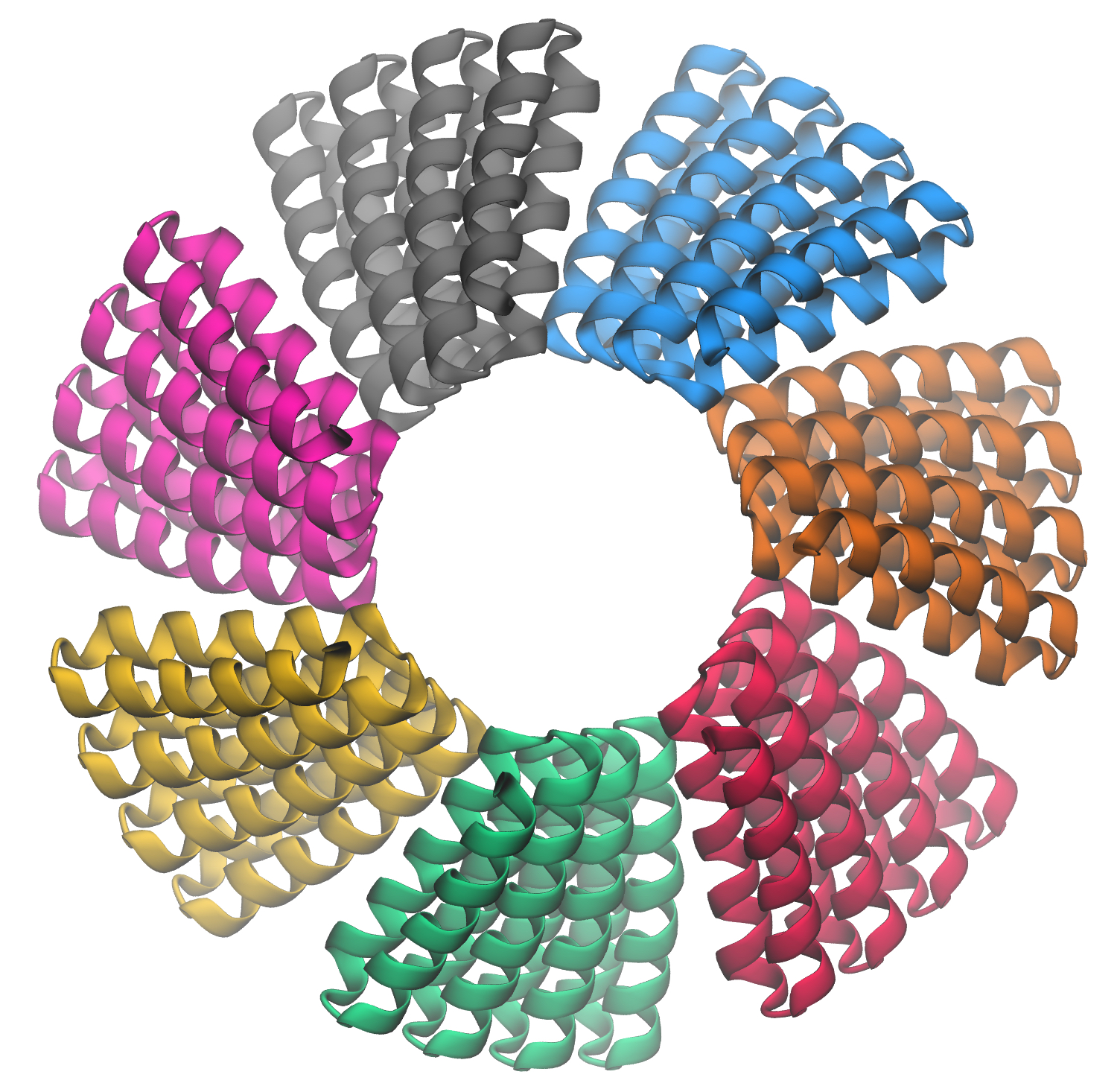

Supplement: Supplementary file 3 — Protein model images, SEC source data and SAXS source data. [file 41557_2023_1314_MOESM3_ESM.zip › Figure2/2o43.jpg]

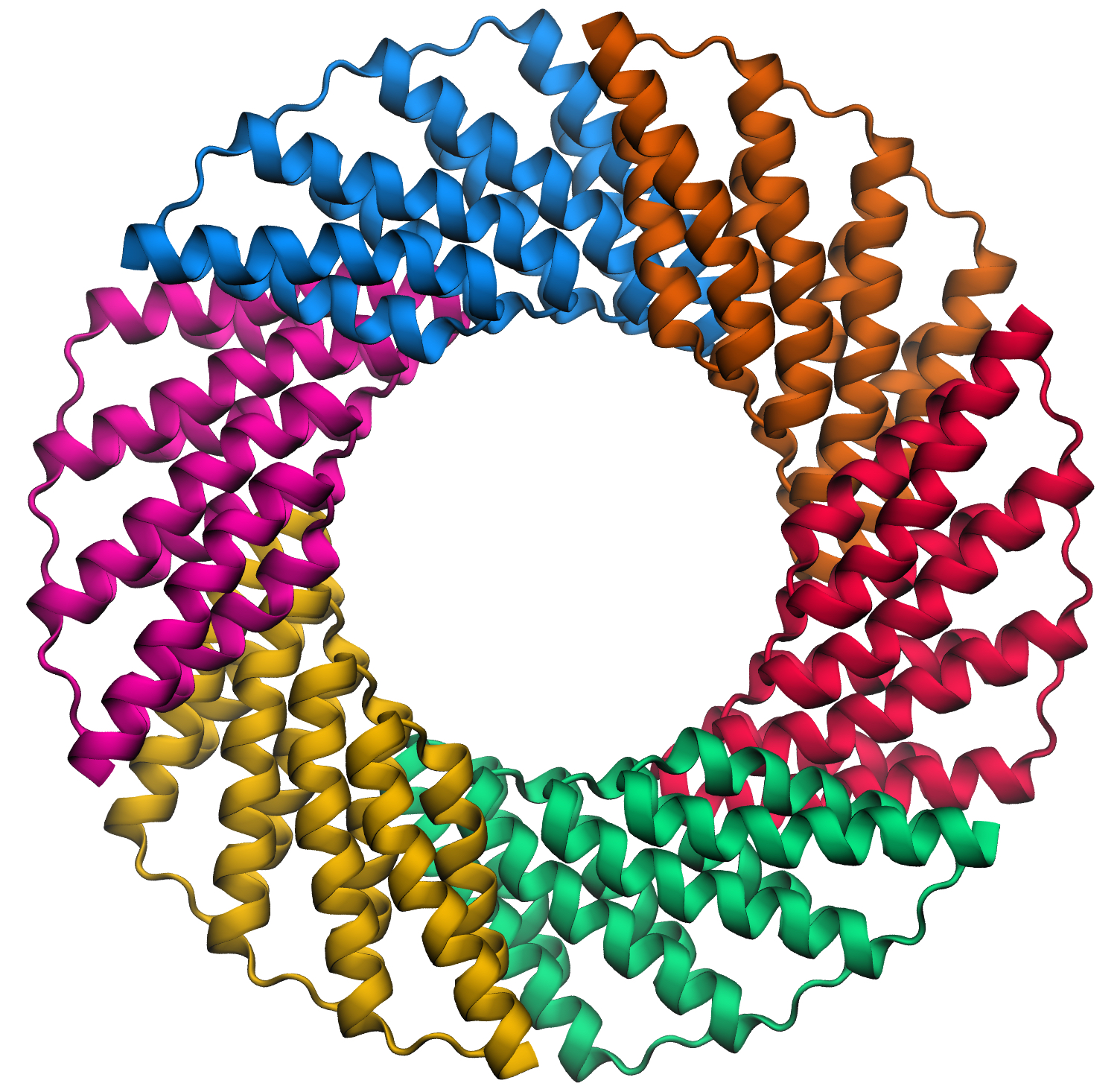

Supplement: Supplementary file 3 — Protein model images, SEC source data and SAXS source data. [file 41557_2023_1314_MOESM3_ESM.zip › Figure2/2o44.jpg]

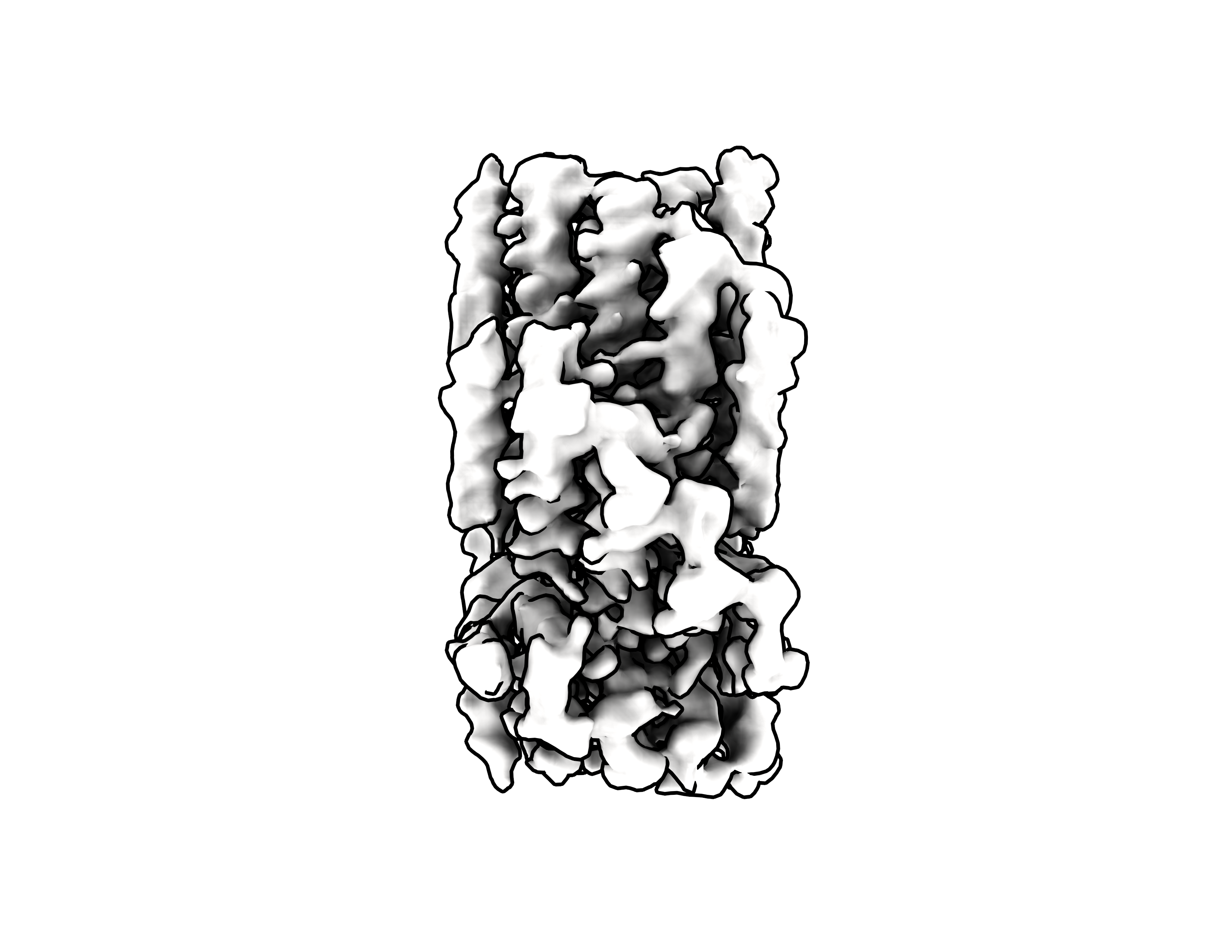

Supplement: Supplementary file 4 — Protein model images, crystal structure images and cryoEM map images. [file 41557_2023_1314_MOESM4_ESM.zip › Figure3/2o44_long.tiff]

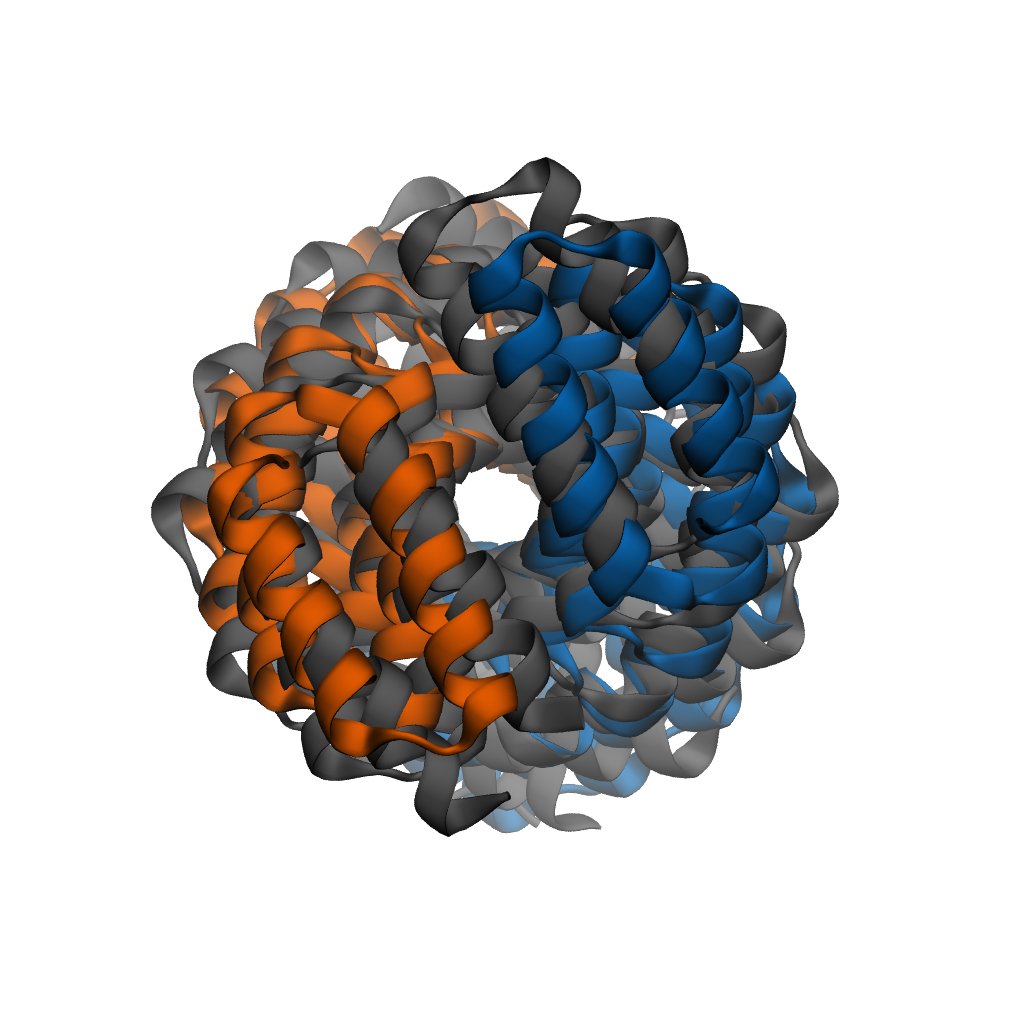

Supplement: Supplementary file 4 — Protein model images, crystal structure images and cryoEM map images. [file 41557_2023_1314_MOESM4_ESM.zip › Figure3/x17_overlay.jpg]

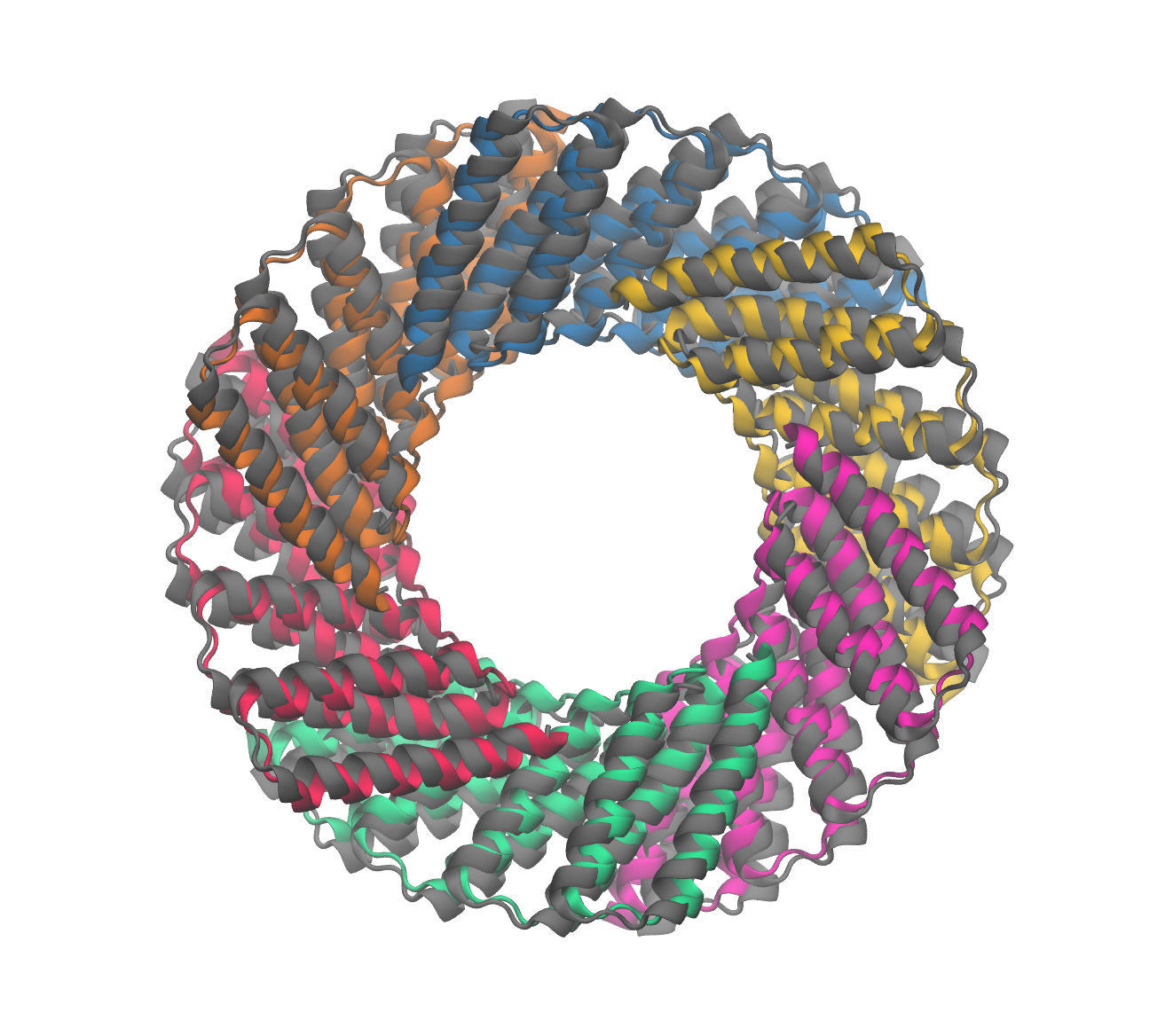

Supplement: Supplementary file 4 — Protein model images, crystal structure images and cryoEM map images. [file 41557_2023_1314_MOESM4_ESM.zip › Figure3/2o44_cryo_top.jpg]

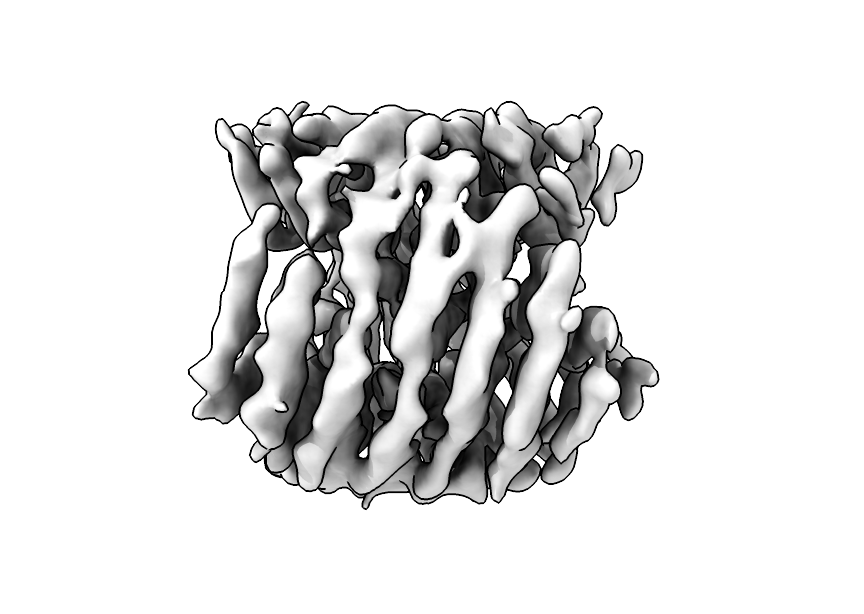

Supplement: Supplementary file 4 — Protein model images, crystal structure images and cryoEM map images. [file 41557_2023_1314_MOESM4_ESM.zip › Figure3/8r11_map_side.tif]

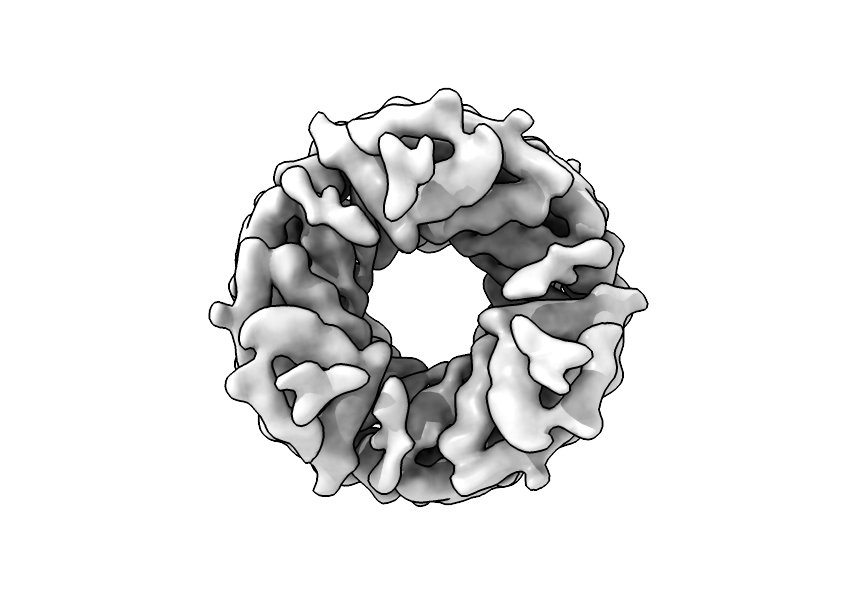

Supplement: Supplementary file 4 — Protein model images, crystal structure images and cryoEM map images. [file 41557_2023_1314_MOESM4_ESM.zip › Figure3/8r11_map_top.tif]

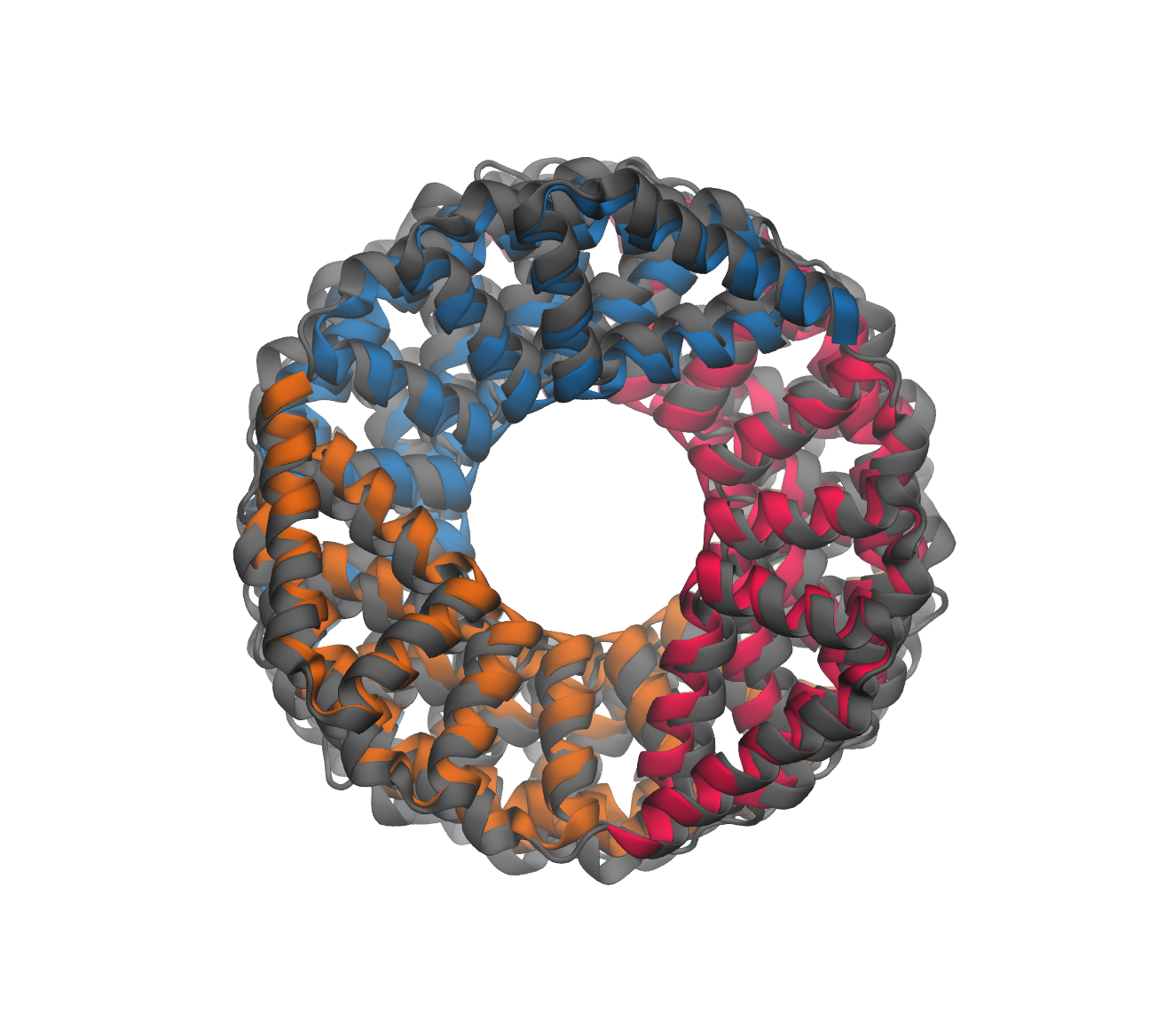

Supplement: Supplementary file 4 — Protein model images, crystal structure images and cryoEM map images. [file 41557_2023_1314_MOESM4_ESM.zip › Figure3/8r11_cryo_top.jpg]

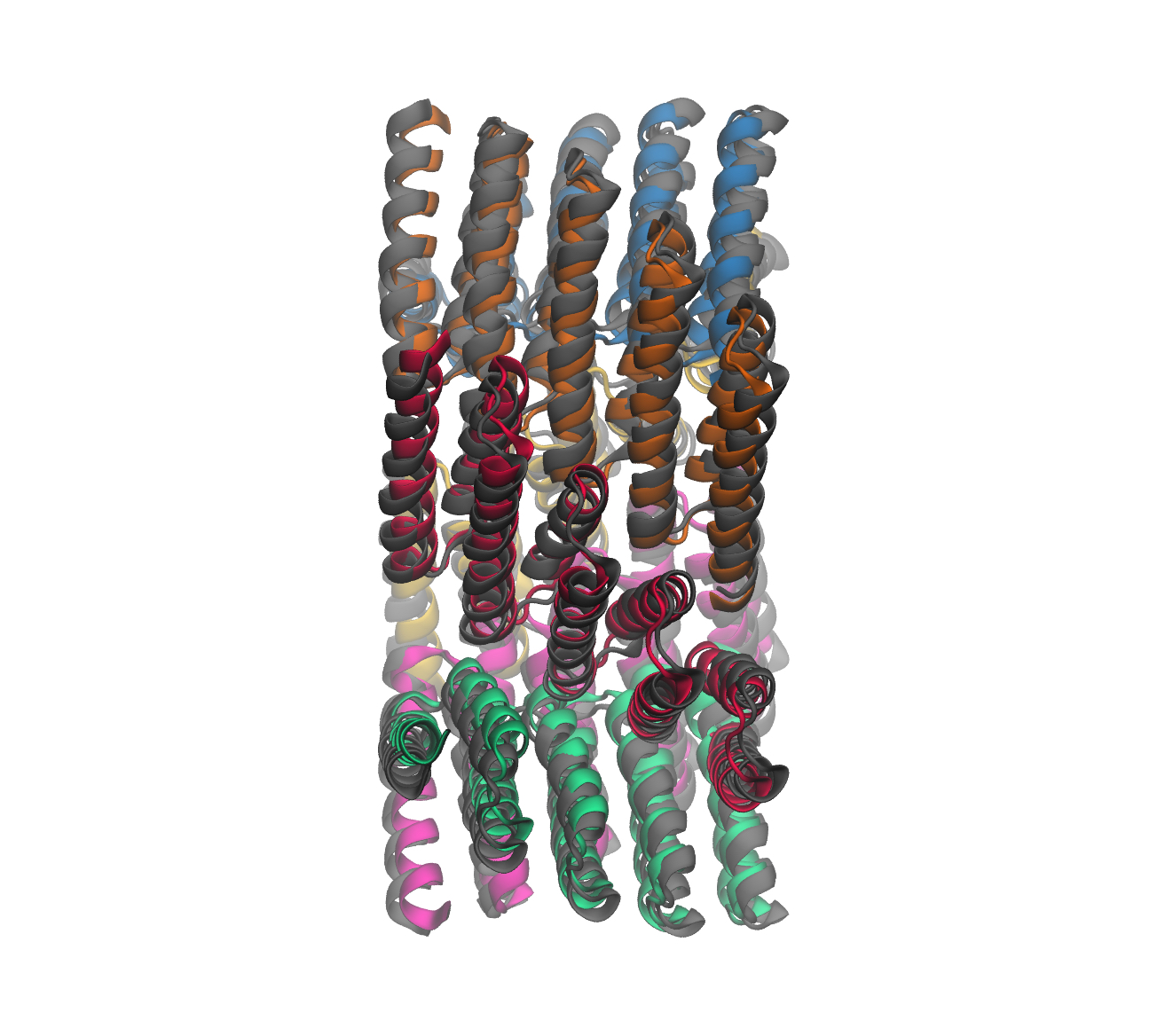

Supplement: Supplementary file 4 — Protein model images, crystal structure images and cryoEM map images. [file 41557_2023_1314_MOESM4_ESM.zip › Figure3/2o44_cryo_side.jpg]

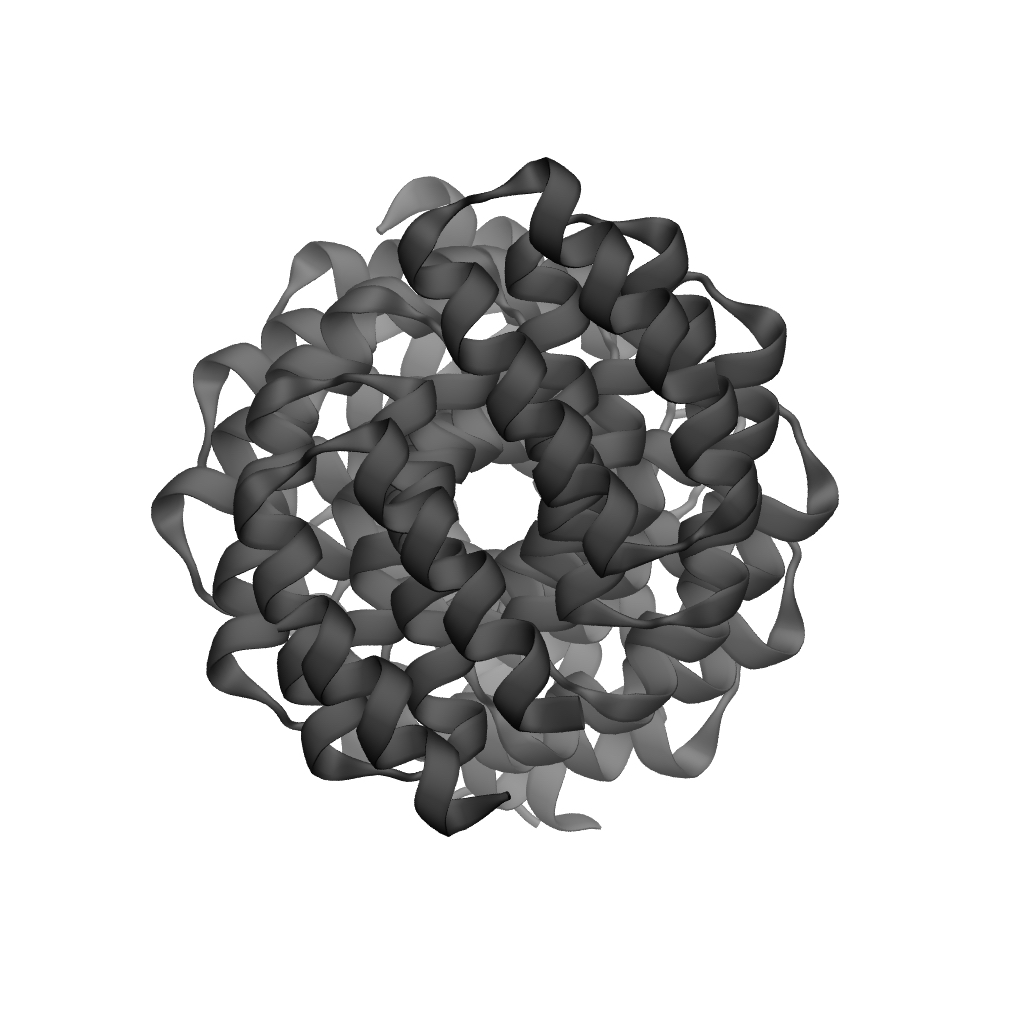

Supplement: Supplementary file 4 — Protein model images, crystal structure images and cryoEM map images. [file 41557_2023_1314_MOESM4_ESM.zip › Figure3/x17_xtal.jpg]

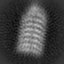

Supplement: Supplementary file 4 — Protein model images, crystal structure images and cryoEM map images. [file 41557_2023_1314_MOESM4_ESM.zip › Figure3/8r20_cryo_top.jpg]

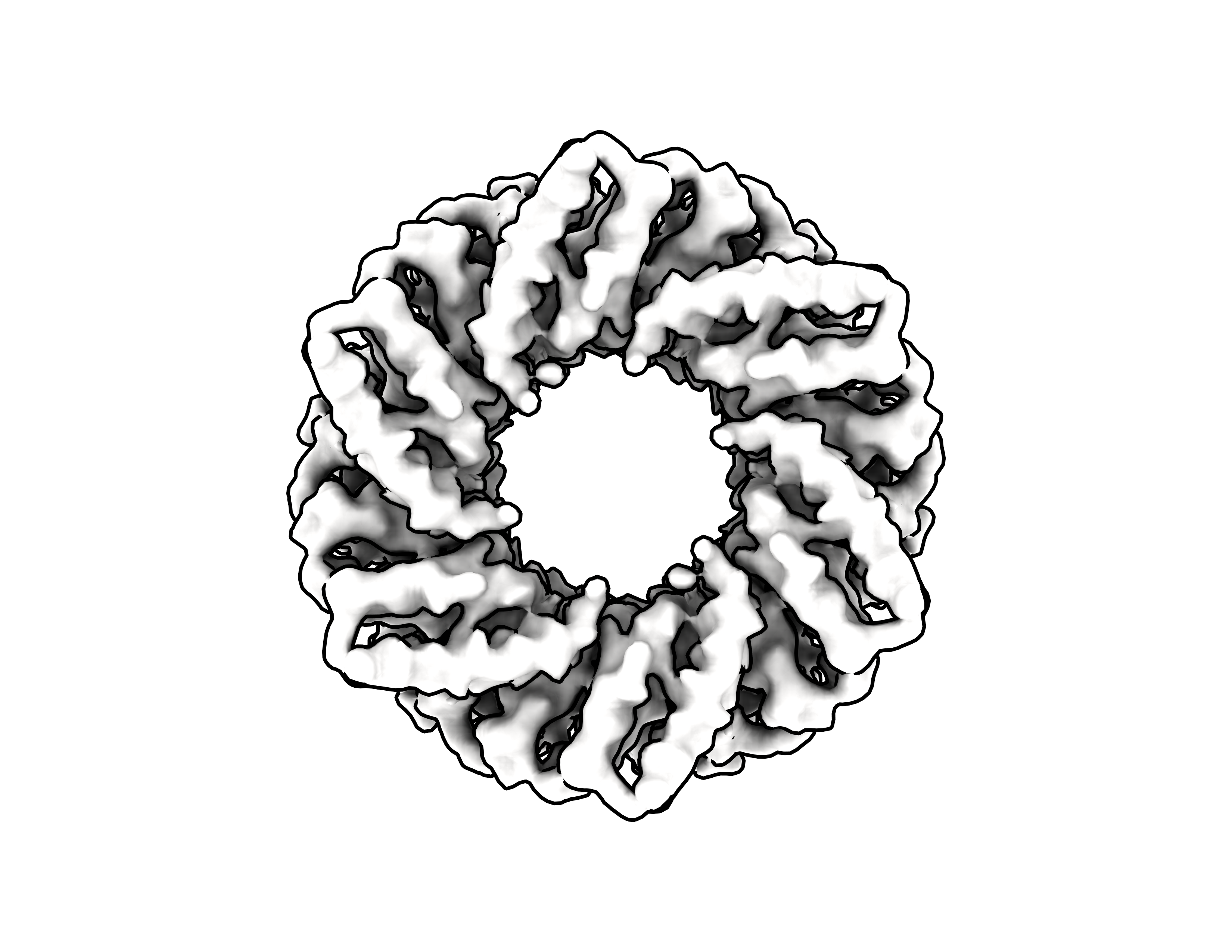

Supplement: Supplementary file 4 — Protein model images, crystal structure images and cryoEM map images. [file 41557_2023_1314_MOESM4_ESM.zip › Figure3/2o44_axis.tiff]

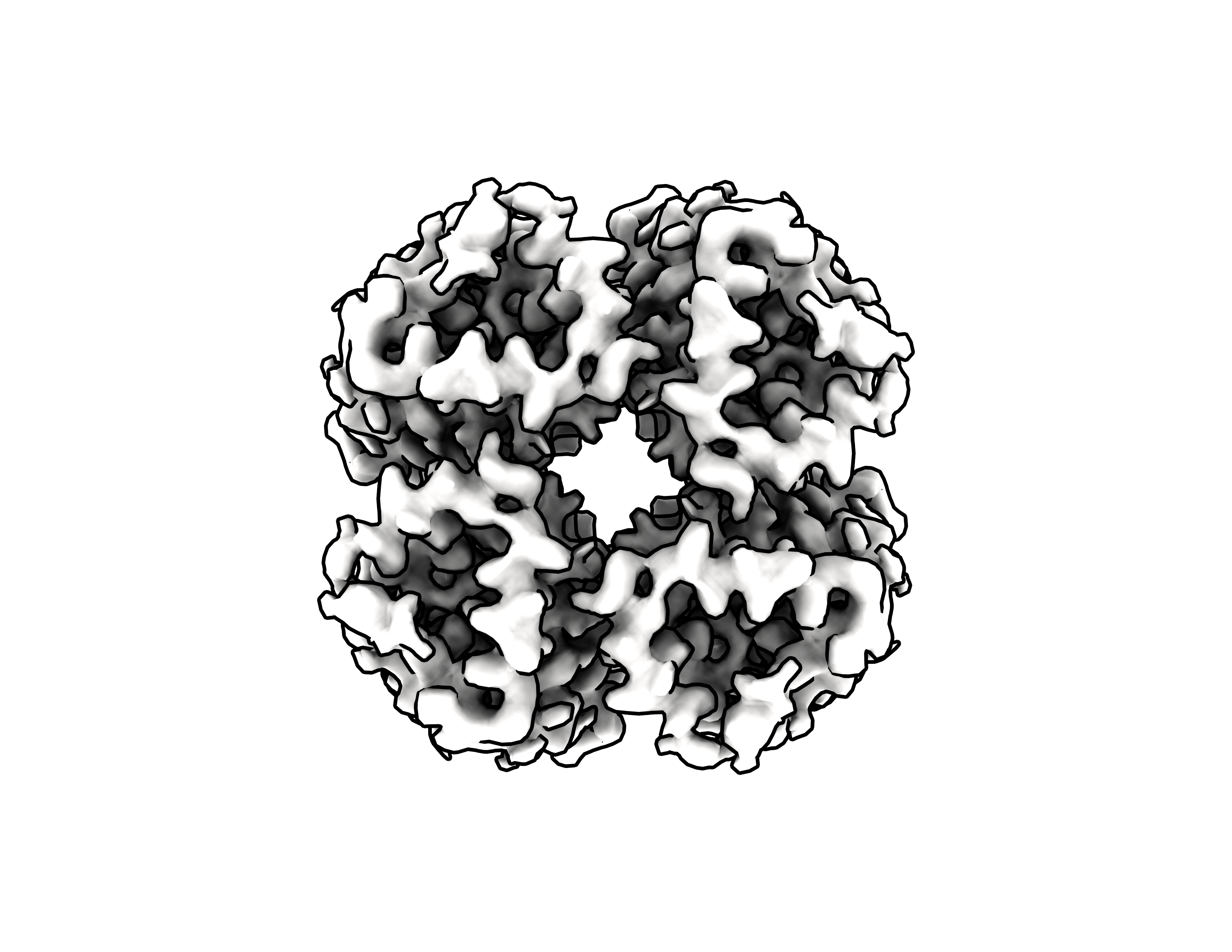

Supplement: Supplementary file 4 — Protein model images, crystal structure images and cryoEM map images. [file 41557_2023_1314_MOESM4_ESM.zip › Figure3/3o22_acix.tiff]

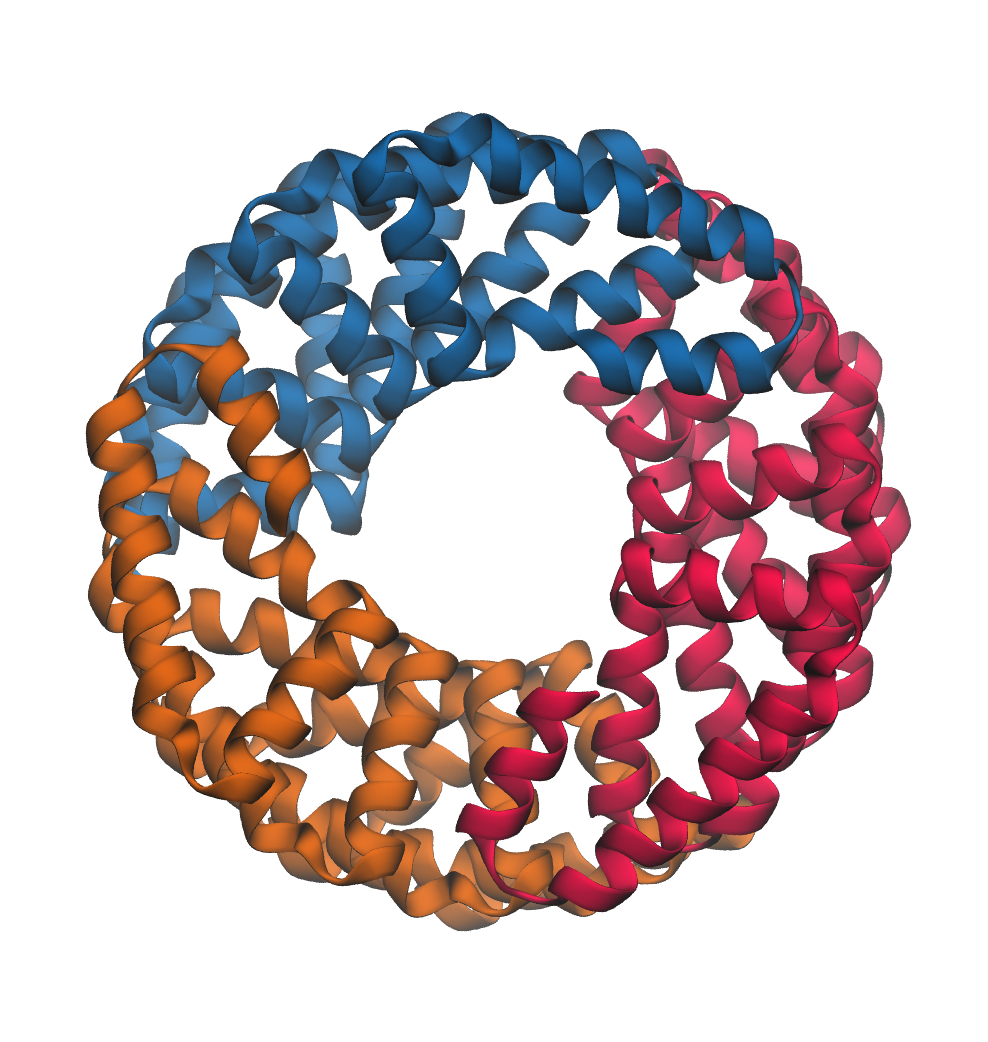

Supplement: Supplementary file 4 — Protein model images, crystal structure images and cryoEM map images. [file 41557_2023_1314_MOESM4_ESM.zip › Figure3/3o52_model.jpg]

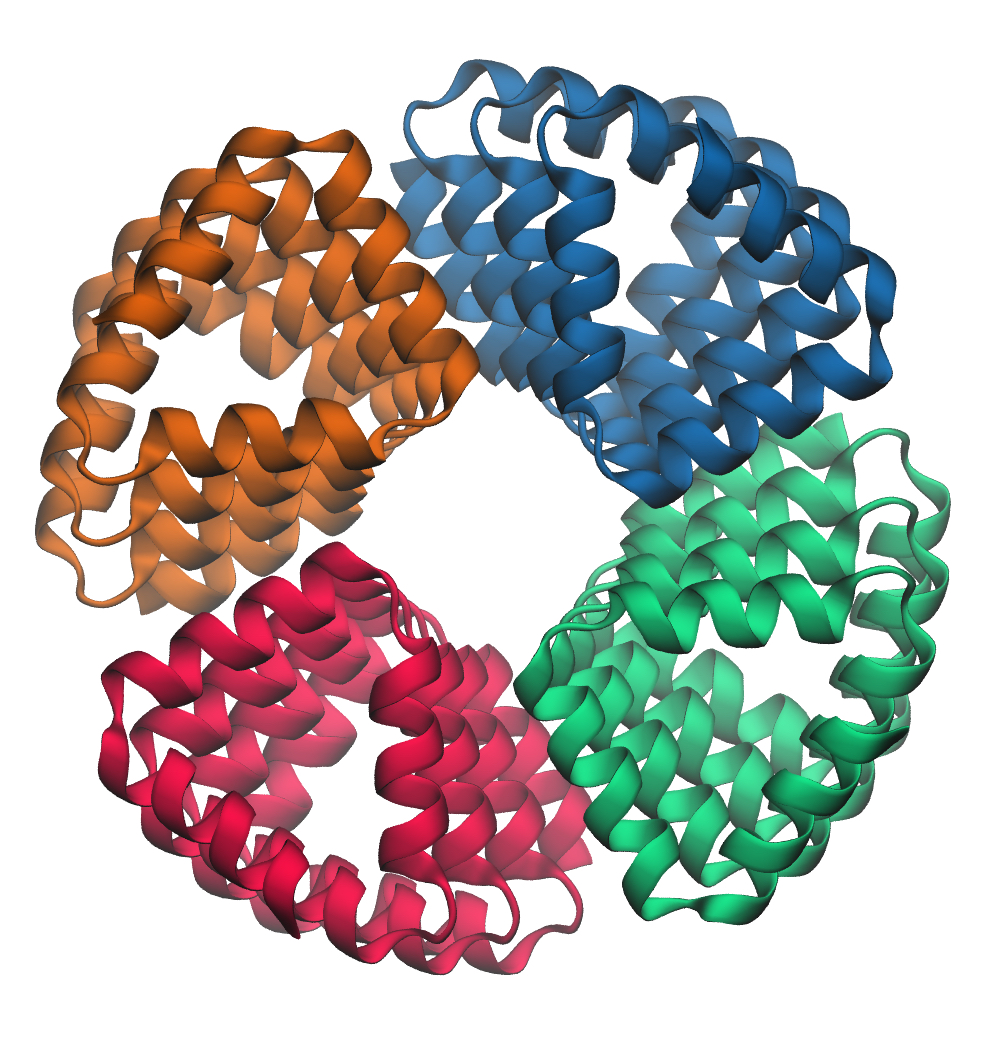

Supplement: Supplementary file 4 — Protein model images, crystal structure images and cryoEM map images. [file 41557_2023_1314_MOESM4_ESM.zip › Figure3/3o22_model.jpg]

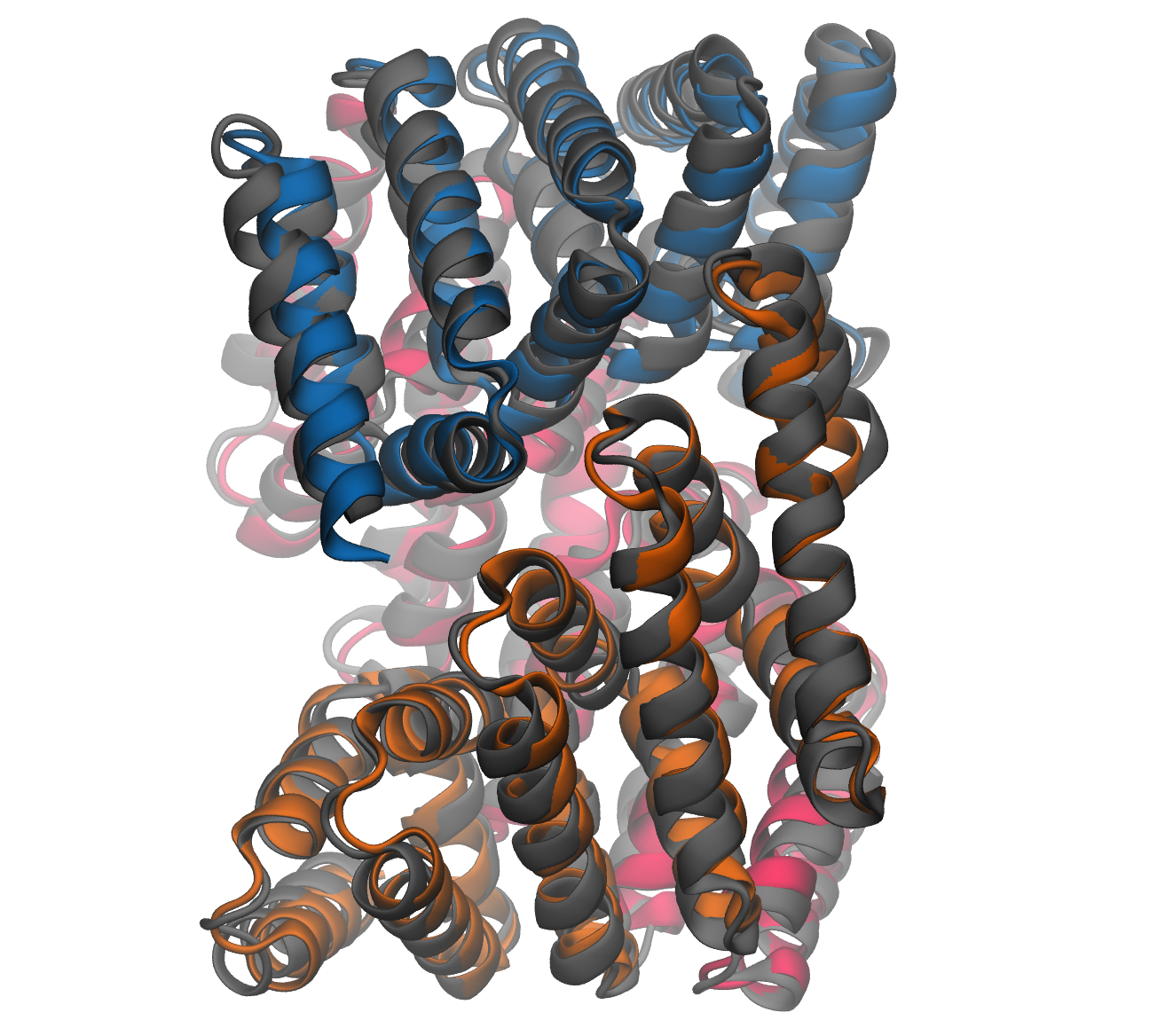

Supplement: Supplementary file 4 — Protein model images, crystal structure images and cryoEM map images. [file 41557_2023_1314_MOESM4_ESM.zip › Figure3/3o52_xtal_side.jpg]

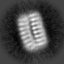

Supplement: Supplementary file 4 — Protein model images, crystal structure images and cryoEM map images. [file 41557_2023_1314_MOESM4_ESM.zip › Figure3/8r43_cryo_side.jpg]

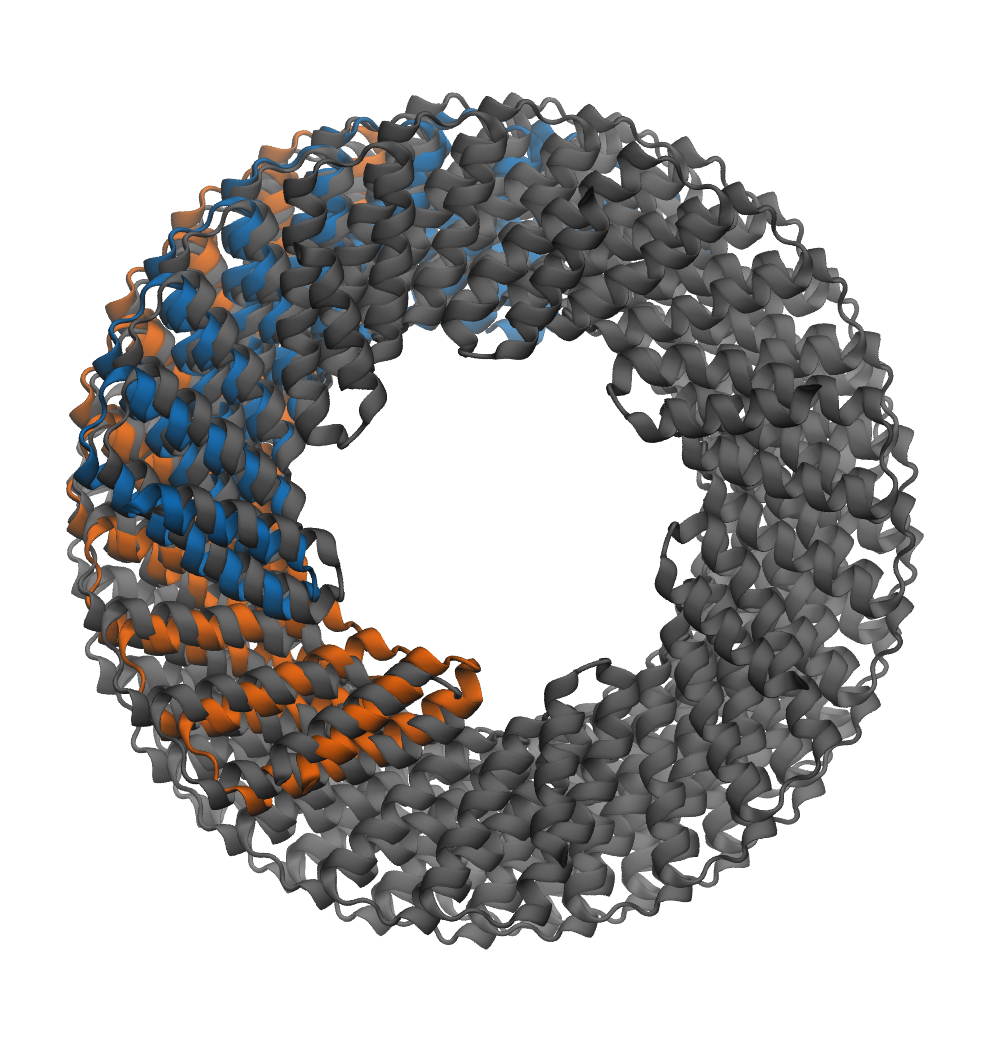

Supplement: Supplementary file 4 — Protein model images, crystal structure images and cryoEM map images. [file 41557_2023_1314_MOESM4_ESM.zip › Figure3/8r10_overlay.jpg]

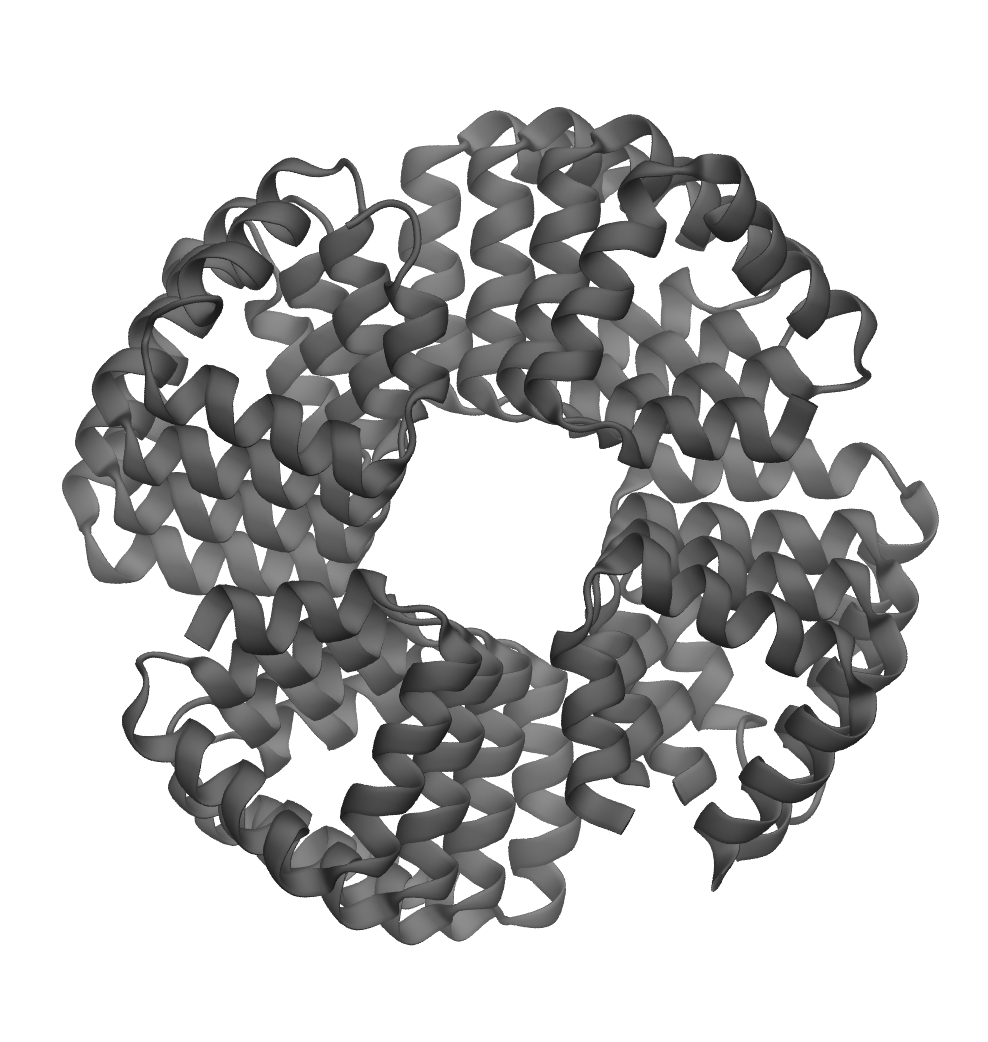

Supplement: Supplementary file 4 — Protein model images, crystal structure images and cryoEM map images. [file 41557_2023_1314_MOESM4_ESM.zip › Figure3/3o22_xtal.jpg]

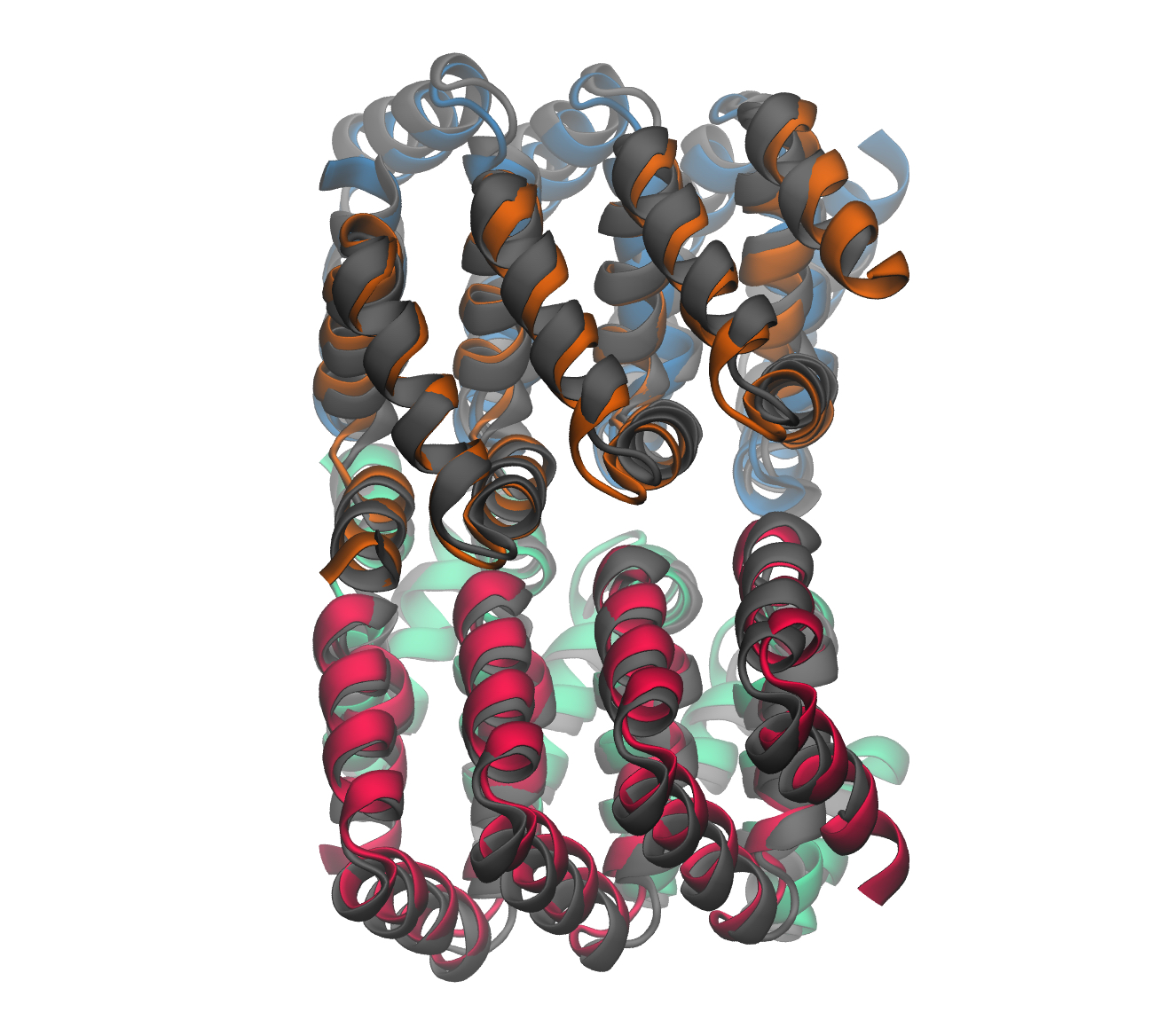

Supplement: Supplementary file 4 — Protein model images, crystal structure images and cryoEM map images. [file 41557_2023_1314_MOESM4_ESM.zip › Figure3/3o22_cryo_side.jpg]

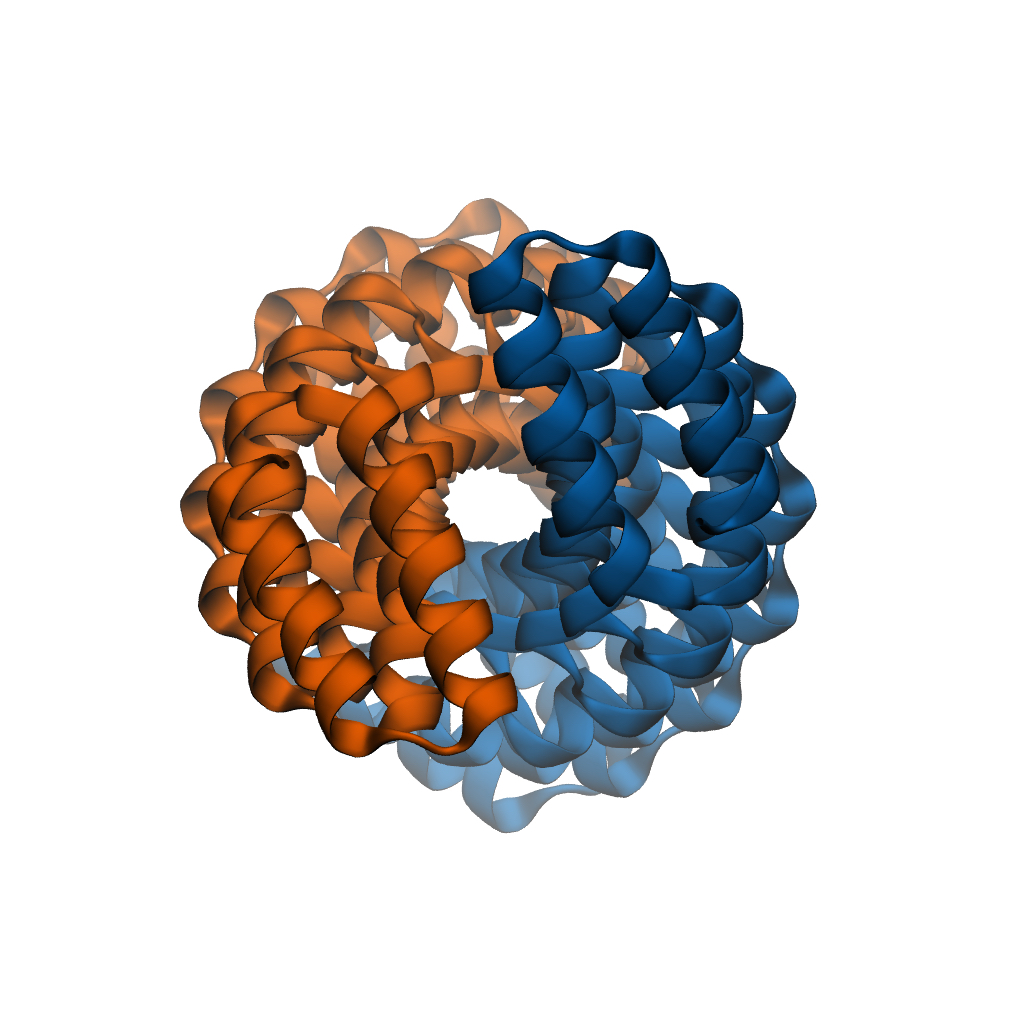

Supplement: Supplementary file 4 — Protein model images, crystal structure images and cryoEM map images. [file 41557_2023_1314_MOESM4_ESM.zip › Figure3/x17_model.jpg]

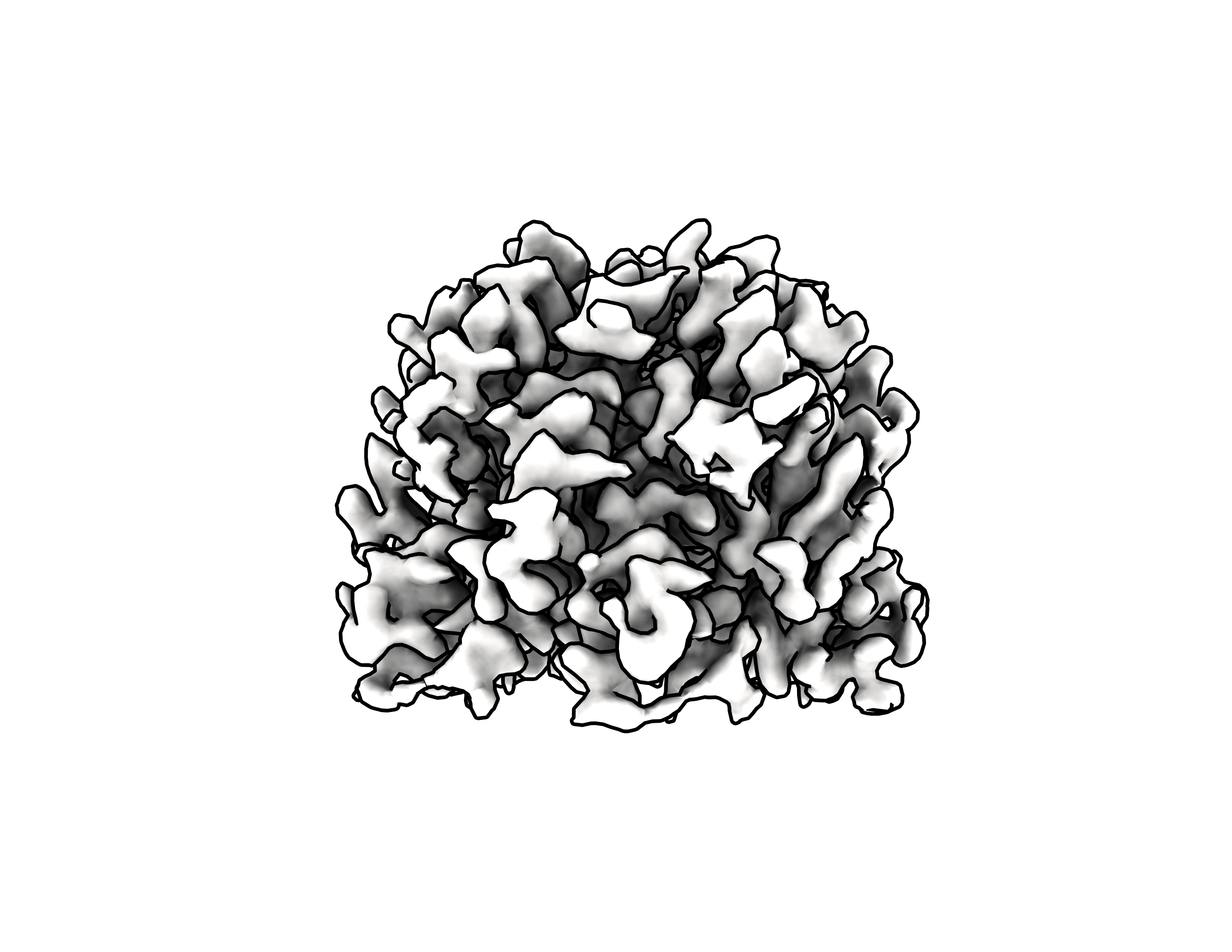

Supplement: Supplementary file 4 — Protein model images, crystal structure images and cryoEM map images. [file 41557_2023_1314_MOESM4_ESM.zip › Figure3/2o31_side.tiff]

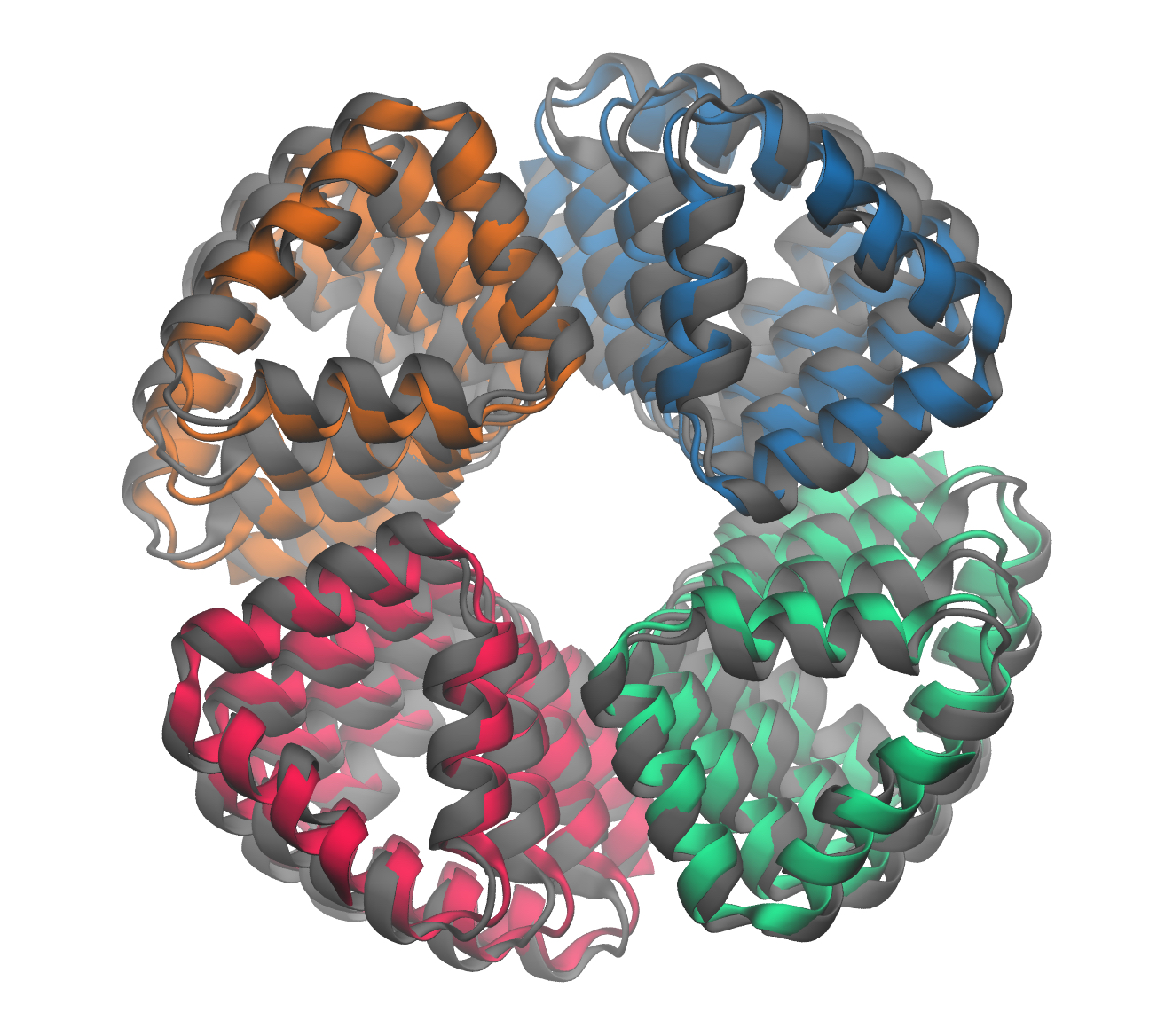

Supplement: Supplementary file 4 — Protein model images, crystal structure images and cryoEM map images. [file 41557_2023_1314_MOESM4_ESM.zip › Figure3/3o22_cryo_top.jpg]

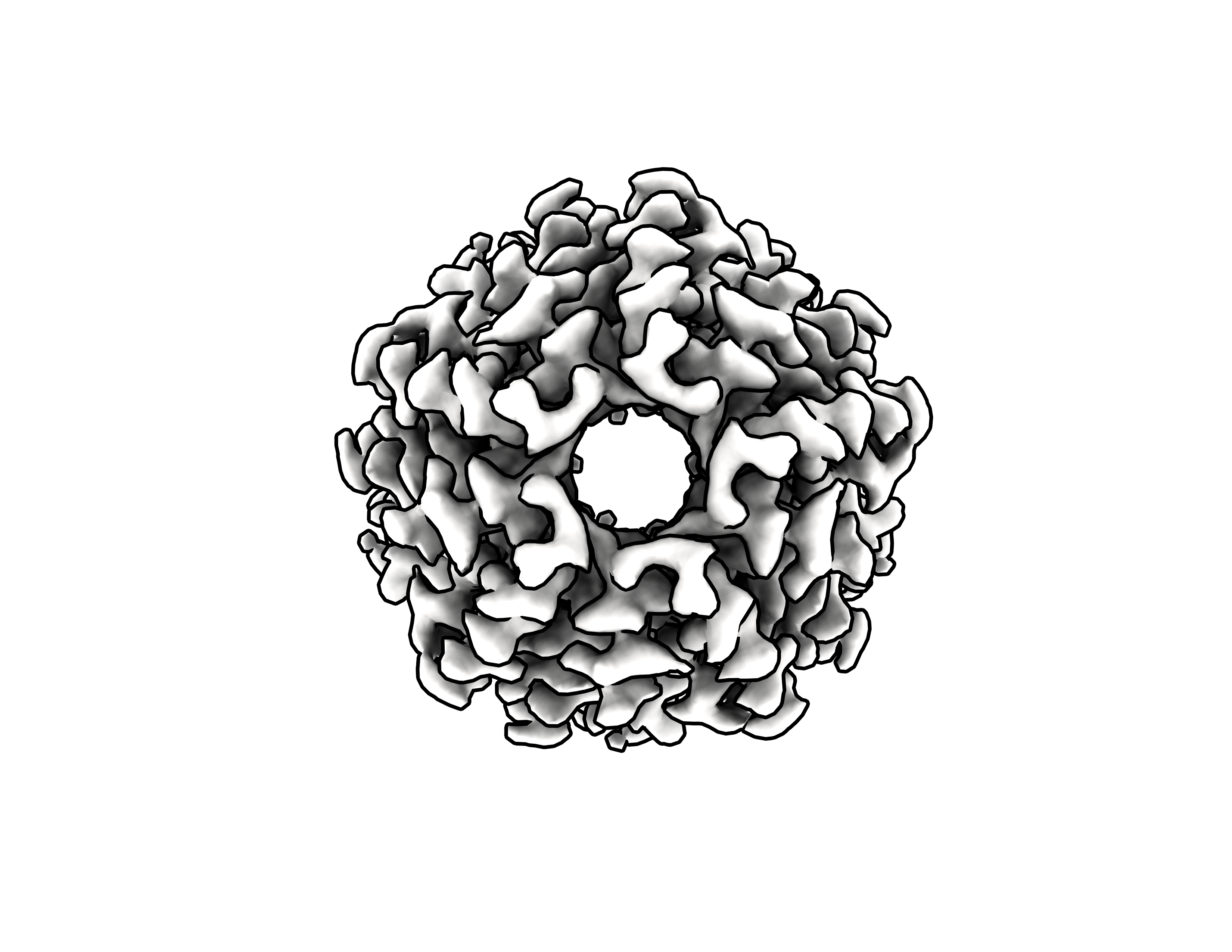

Supplement: Supplementary file 4 — Protein model images, crystal structure images and cryoEM map images. [file 41557_2023_1314_MOESM4_ESM.zip › Figure3/2o31_axis.tiff]

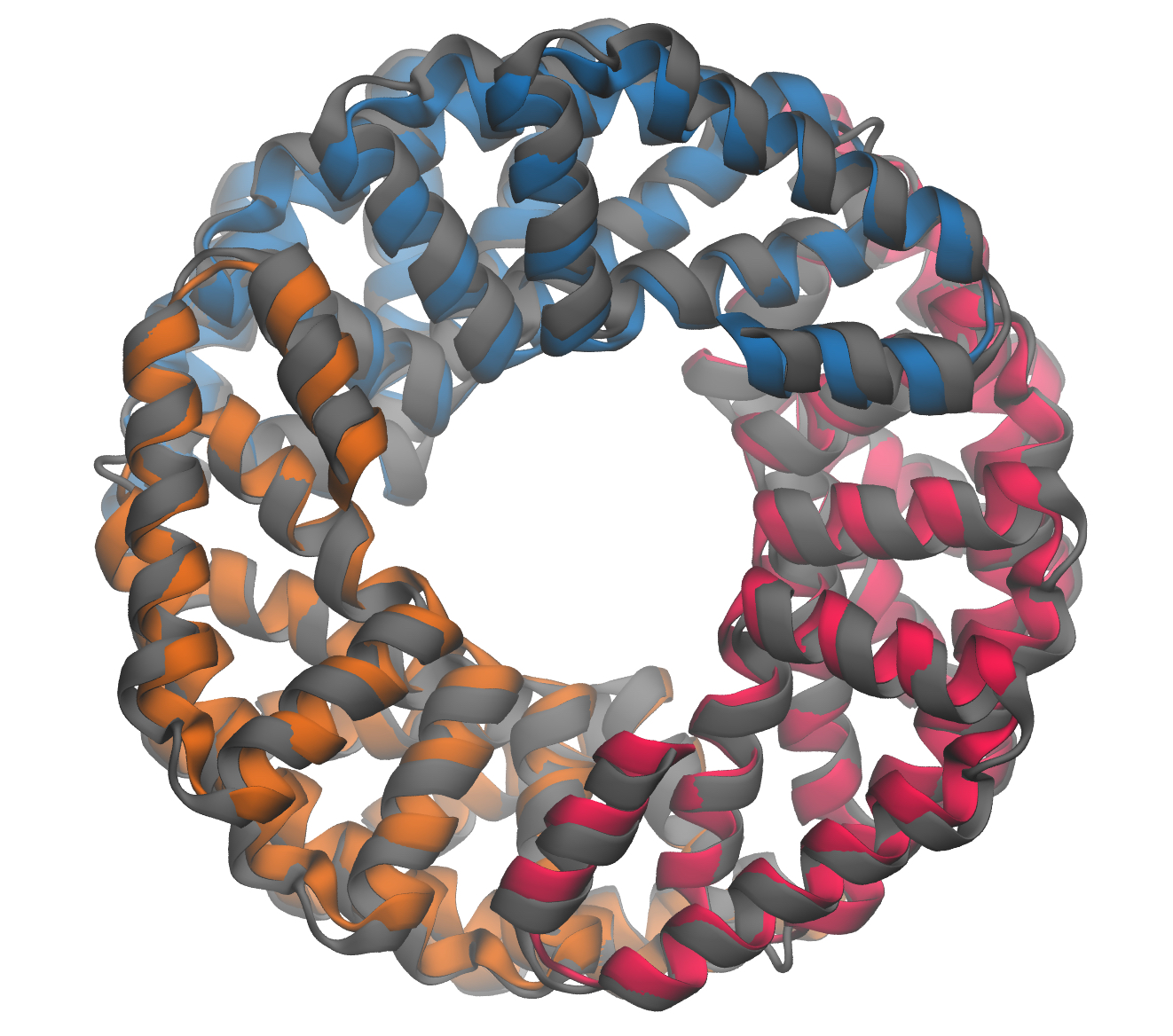

Supplement: Supplementary file 4 — Protein model images, crystal structure images and cryoEM map images. [file 41557_2023_1314_MOESM4_ESM.zip › Figure3/3o52_xtal_top.jpg]

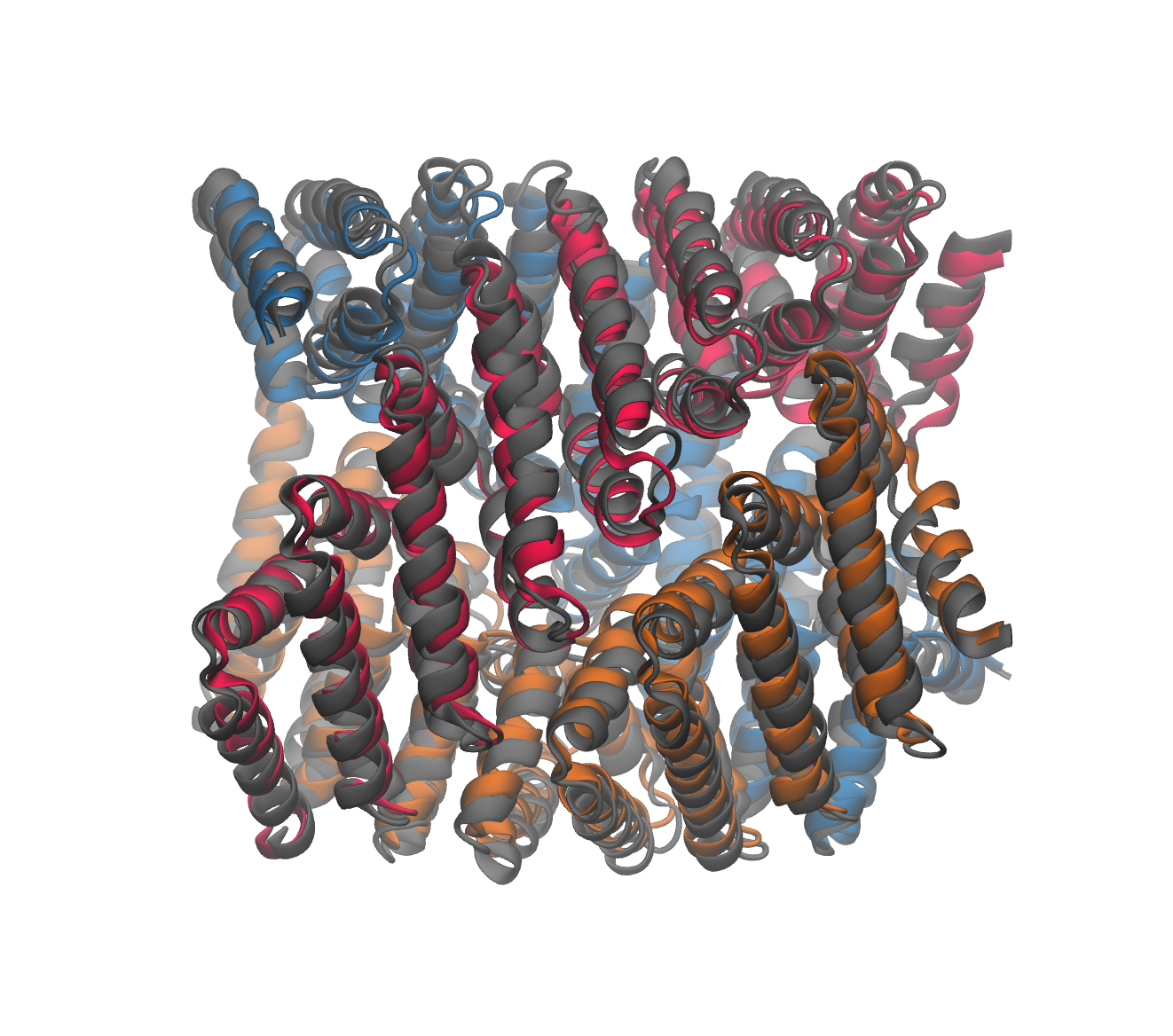

Supplement: Supplementary file 4 — Protein model images, crystal structure images and cryoEM map images. [file 41557_2023_1314_MOESM4_ESM.zip › Figure3/8r11_cryo_side.jpg]

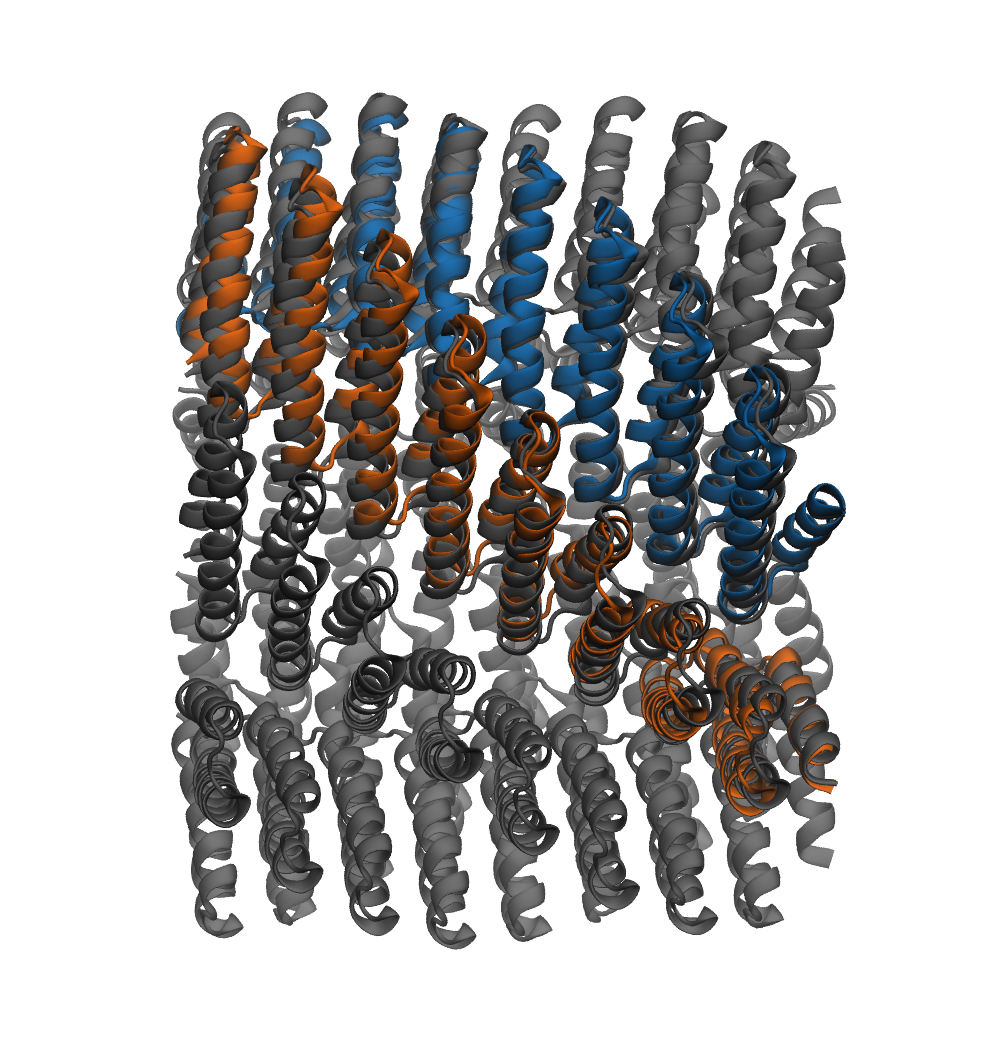

Supplement: Supplementary file 4 — Protein model images, crystal structure images and cryoEM map images. [file 41557_2023_1314_MOESM4_ESM.zip › Figure3/8r10_overlay_side.jpg]

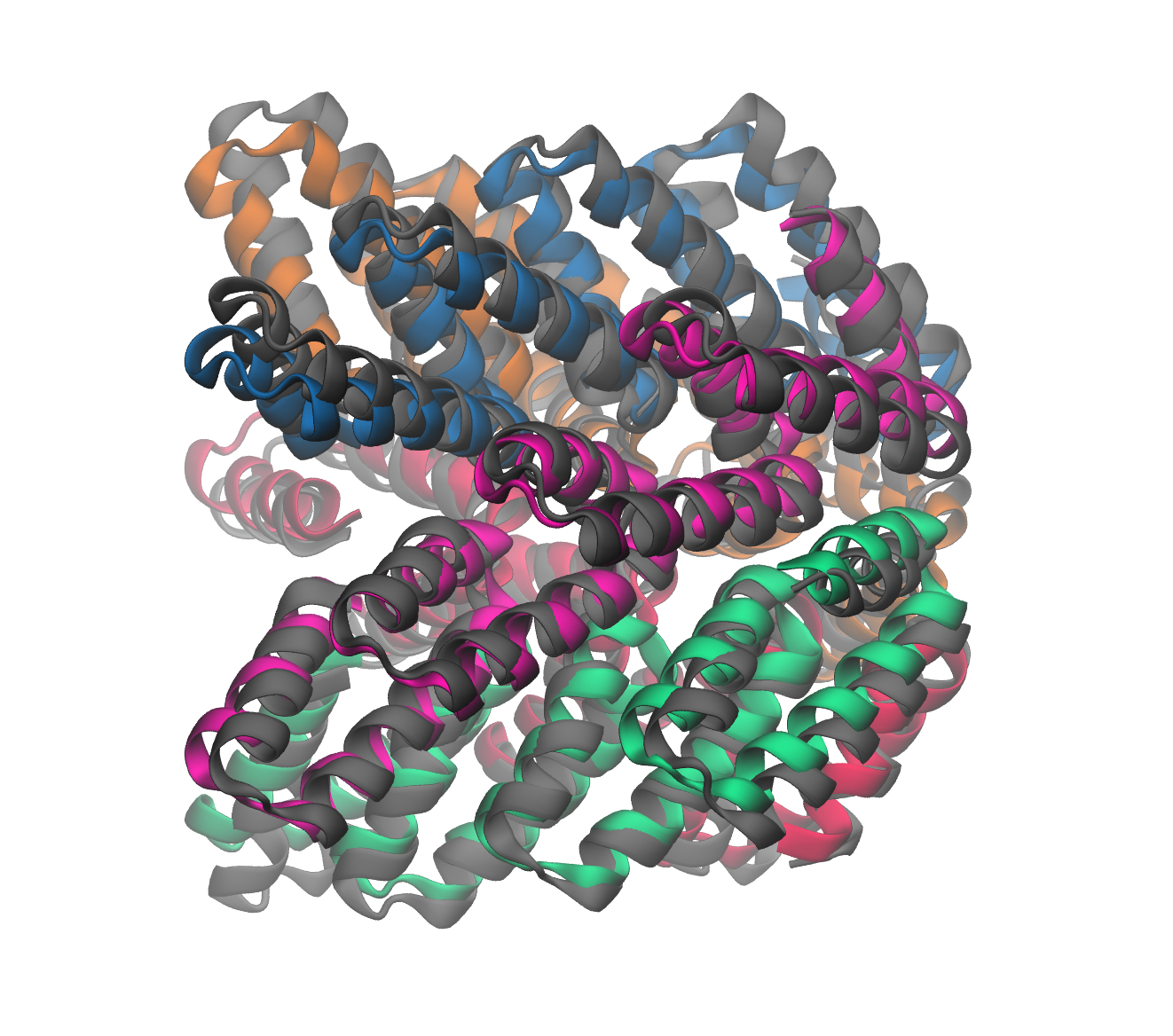

Supplement: Supplementary file 4 — Protein model images, crystal structure images and cryoEM map images. [file 41557_2023_1314_MOESM4_ESM.zip › Figure3/2o31_cryo_side.jpg]

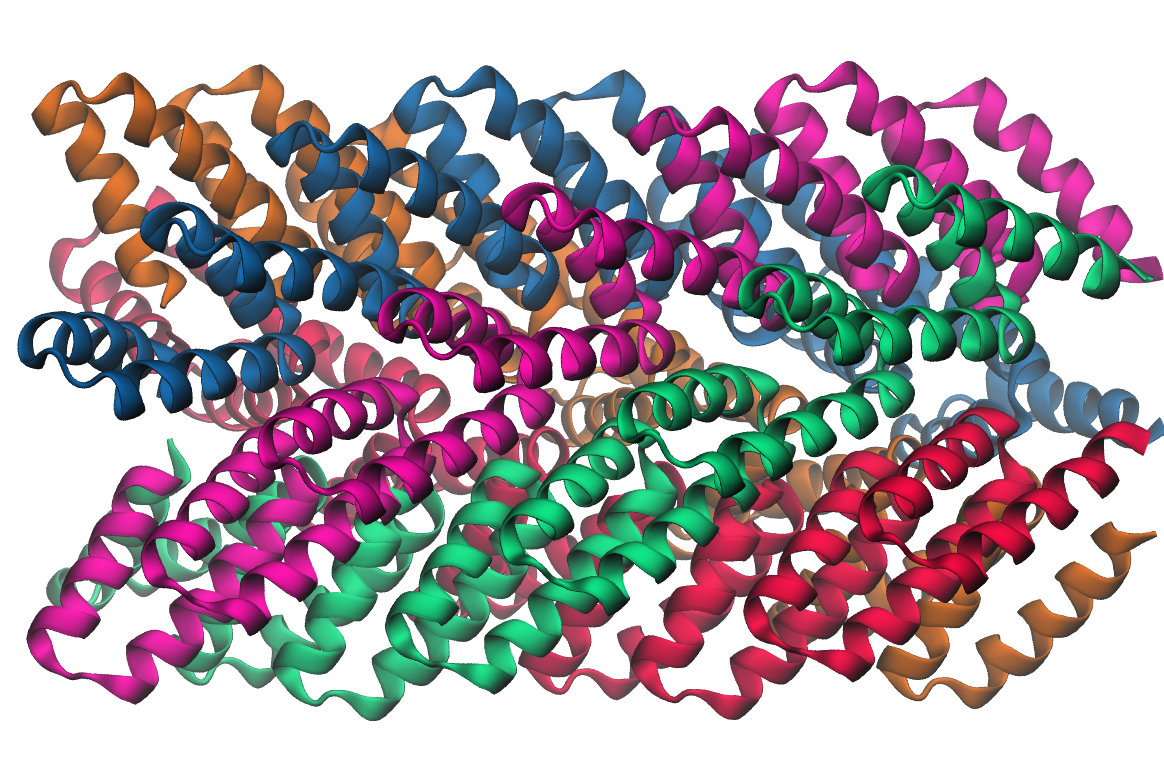

Supplement: Supplementary file 4 — Protein model images, crystal structure images and cryoEM map images. [file 41557_2023_1314_MOESM4_ESM.zip › Figure3/8r20_model_side.jpg]

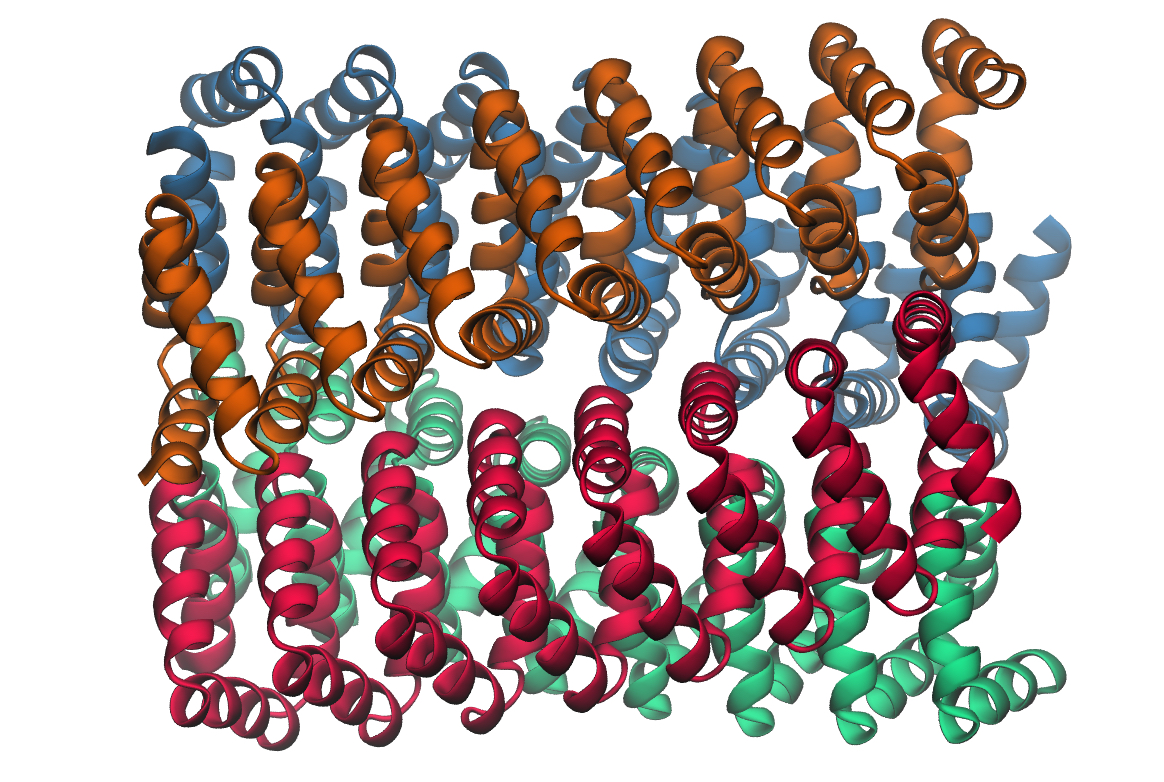

Supplement: Supplementary file 4 — Protein model images, crystal structure images and cryoEM map images. [file 41557_2023_1314_MOESM4_ESM.zip › Figure3/8r43_model_side.jpg]

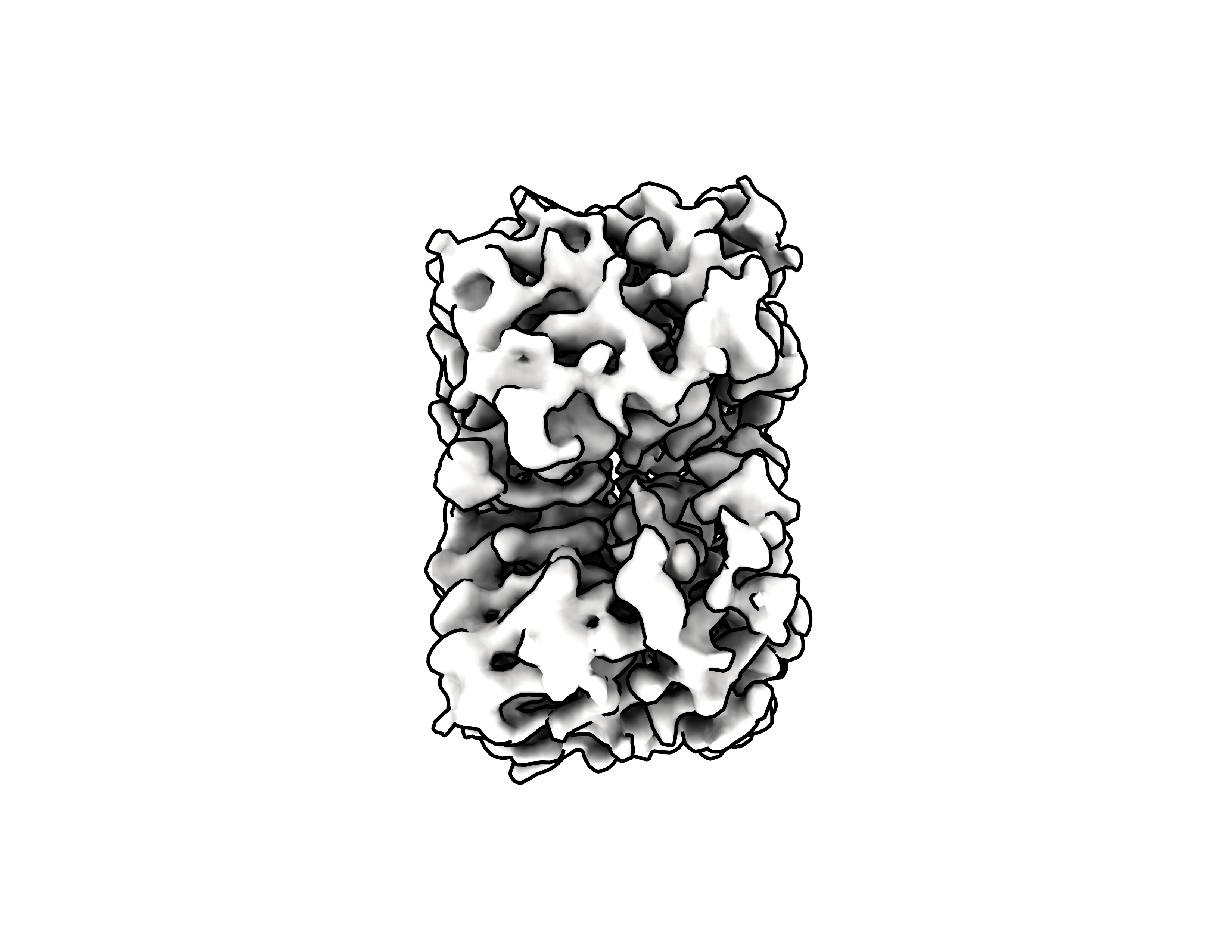

Supplement: Supplementary file 4 — Protein model images, crystal structure images and cryoEM map images. [file 41557_2023_1314_MOESM4_ESM.zip › Figure3/3o22_long.tiff]

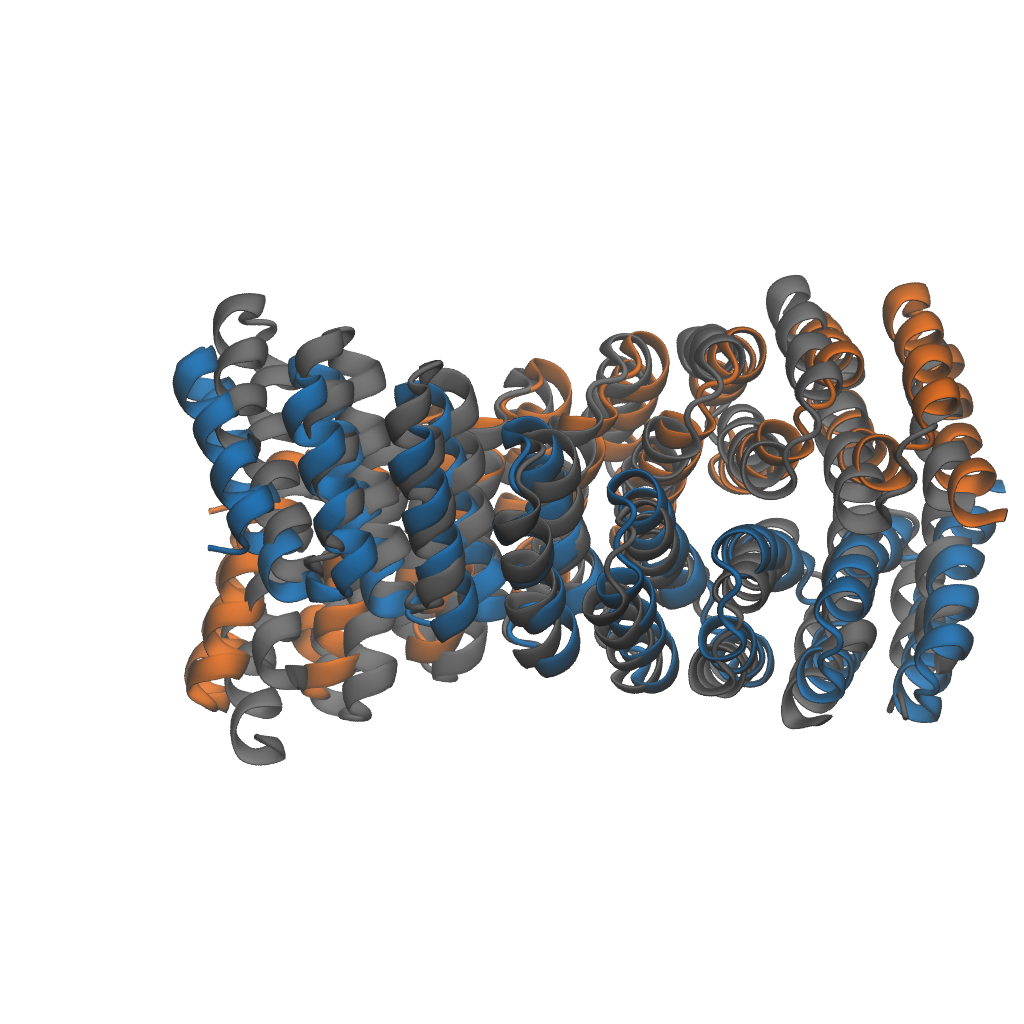

Supplement: Supplementary file 4 — Protein model images, crystal structure images and cryoEM map images. [file 41557_2023_1314_MOESM4_ESM.zip › Figure3/x17_overlay_side.jpg]

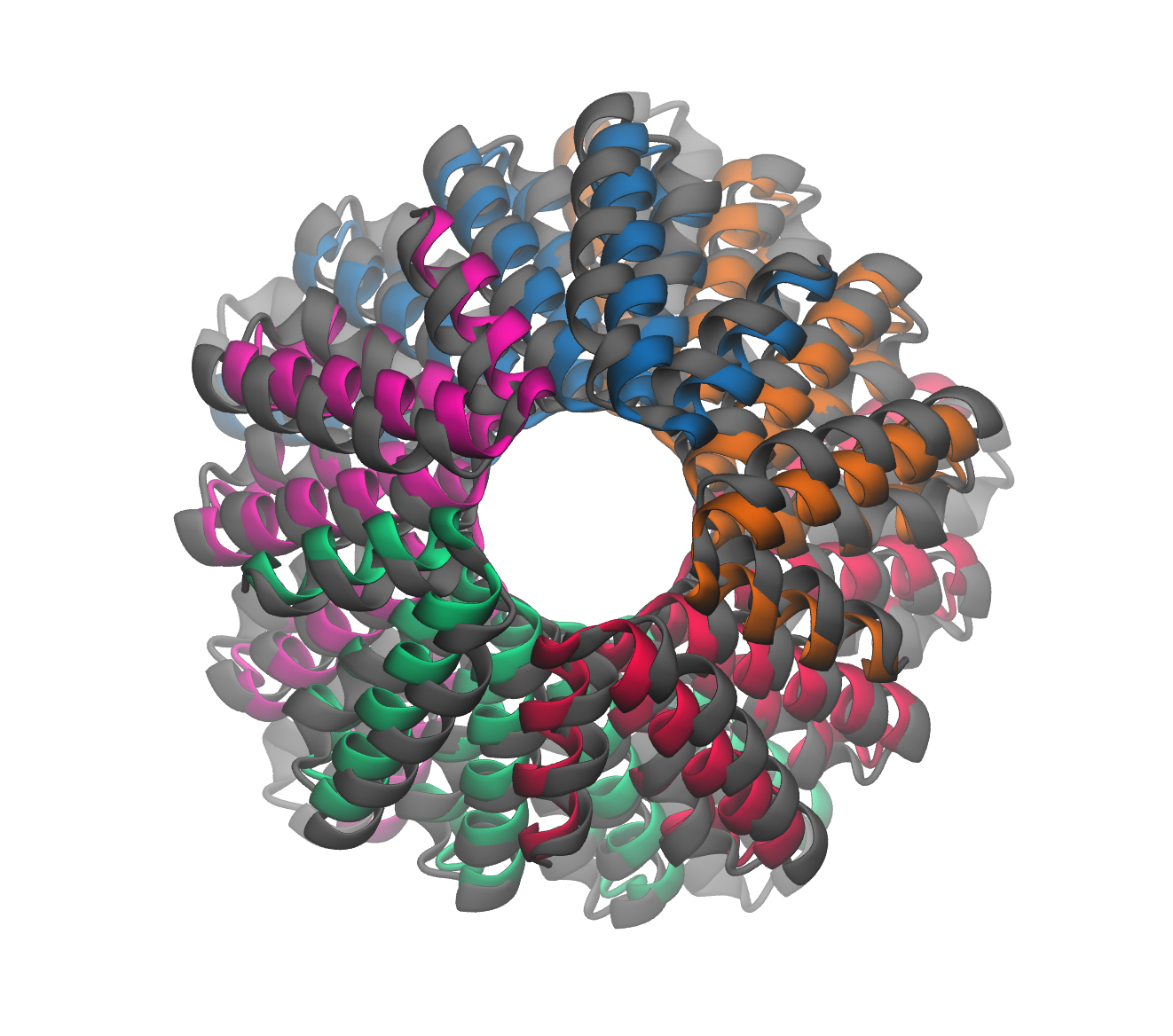

Supplement: Supplementary file 4 — Protein model images, crystal structure images and cryoEM map images. [file 41557_2023_1314_MOESM4_ESM.zip › Figure3/2o31_cryo_top.jpg]

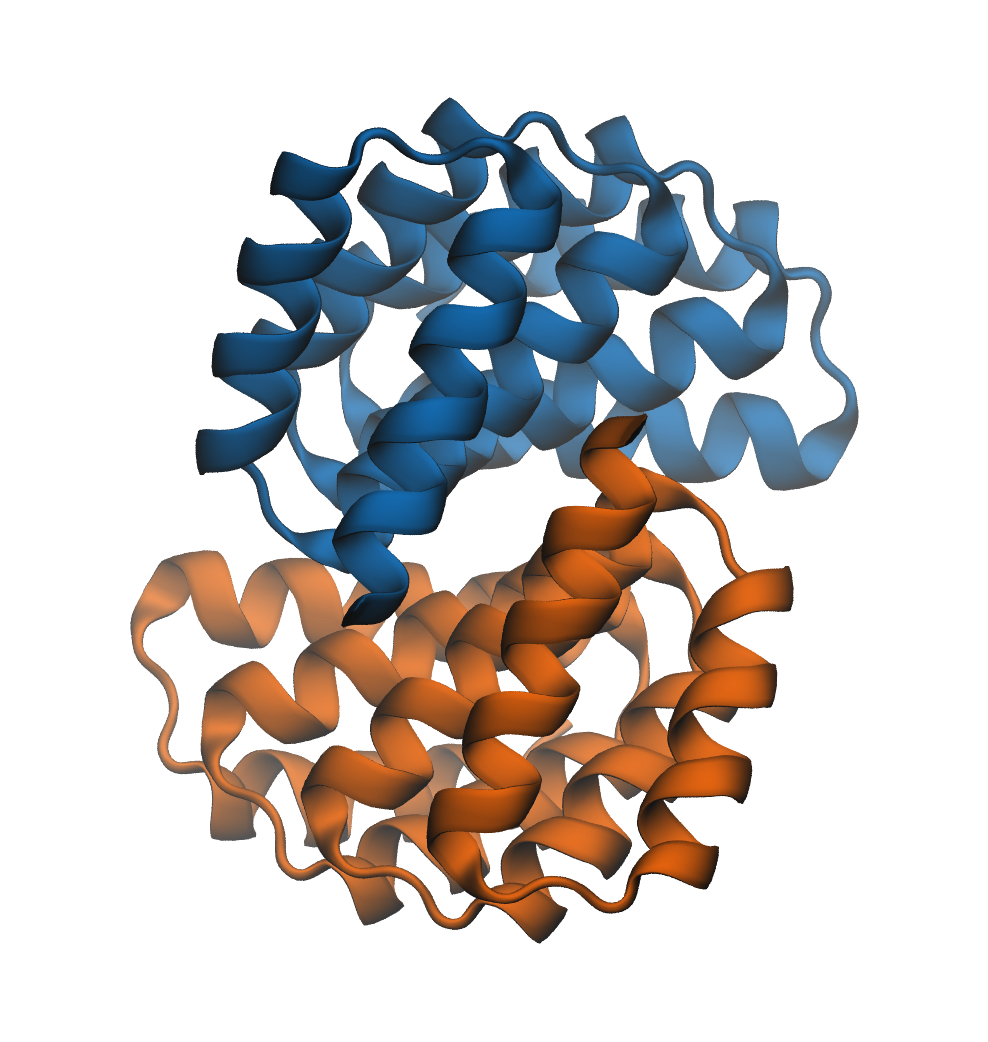

Supplement: Supplementary file 4 — Protein model images, crystal structure images and cryoEM map images. [file 41557_2023_1314_MOESM4_ESM.zip › Figure3/2d7_model.jpg]

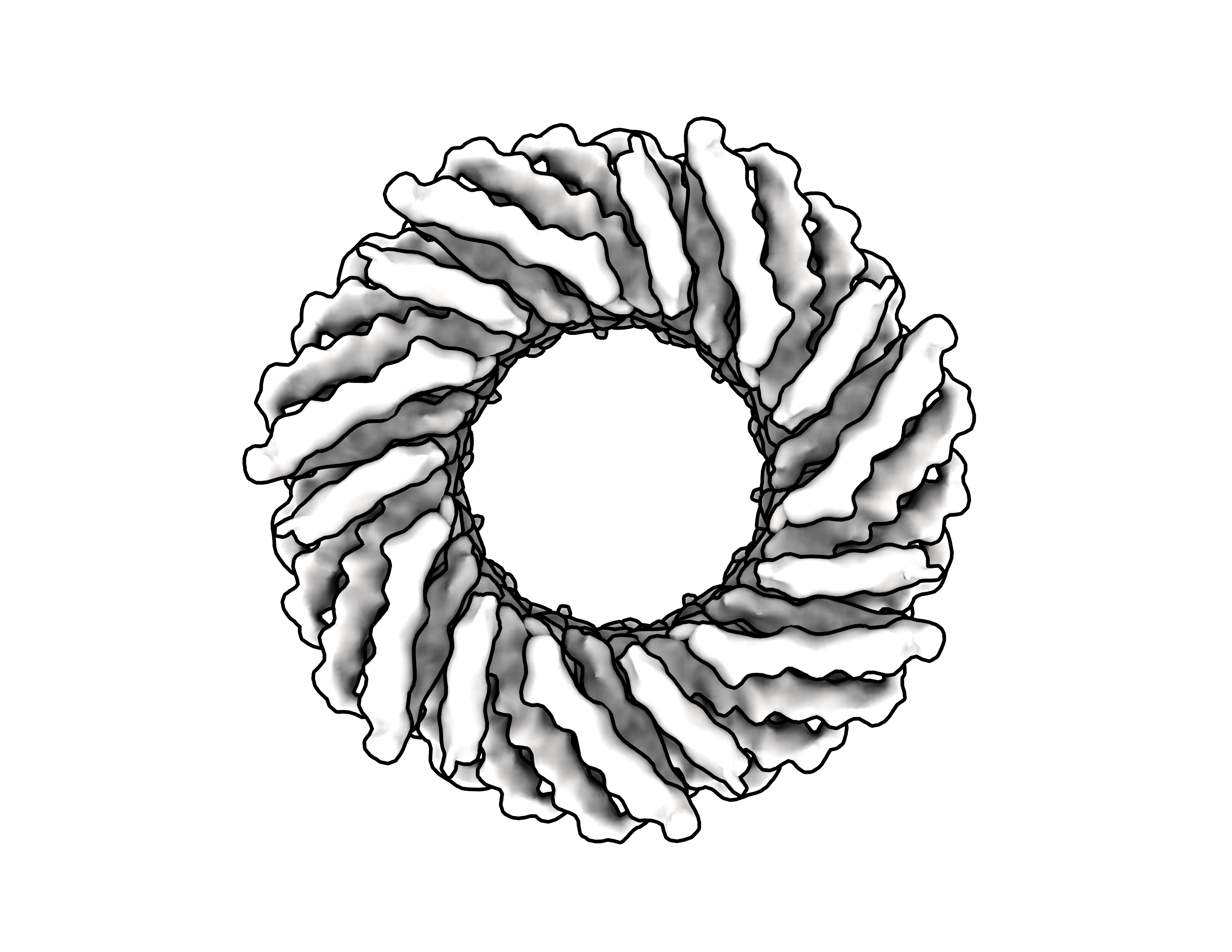

Supplement: Supplementary file 4 — Protein model images, crystal structure images and cryoEM map images. [file 41557_2023_1314_MOESM4_ESM.zip › Figure3/8r10_axis.jpg]

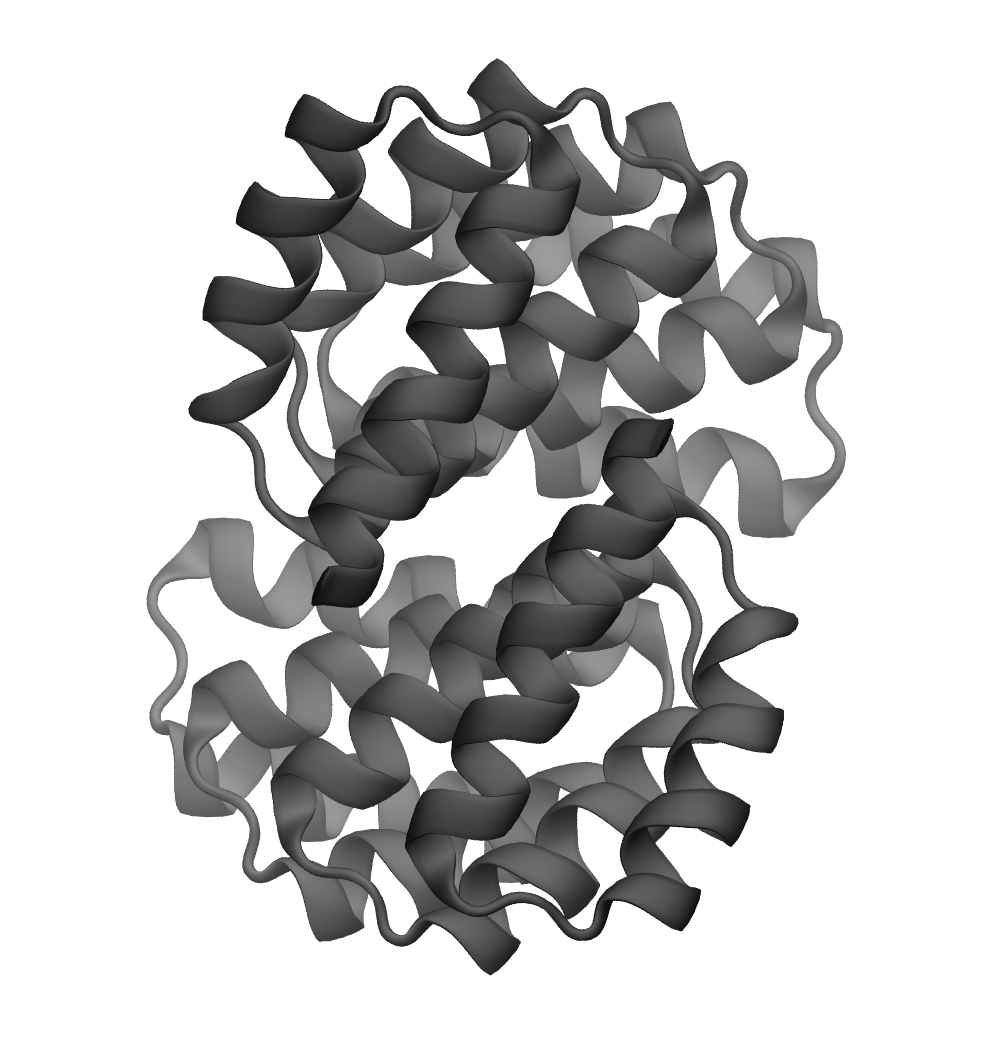

Supplement: Supplementary file 4 — Protein model images, crystal structure images and cryoEM map images. [file 41557_2023_1314_MOESM4_ESM.zip › Figure3/2d7_xtal.jpg]

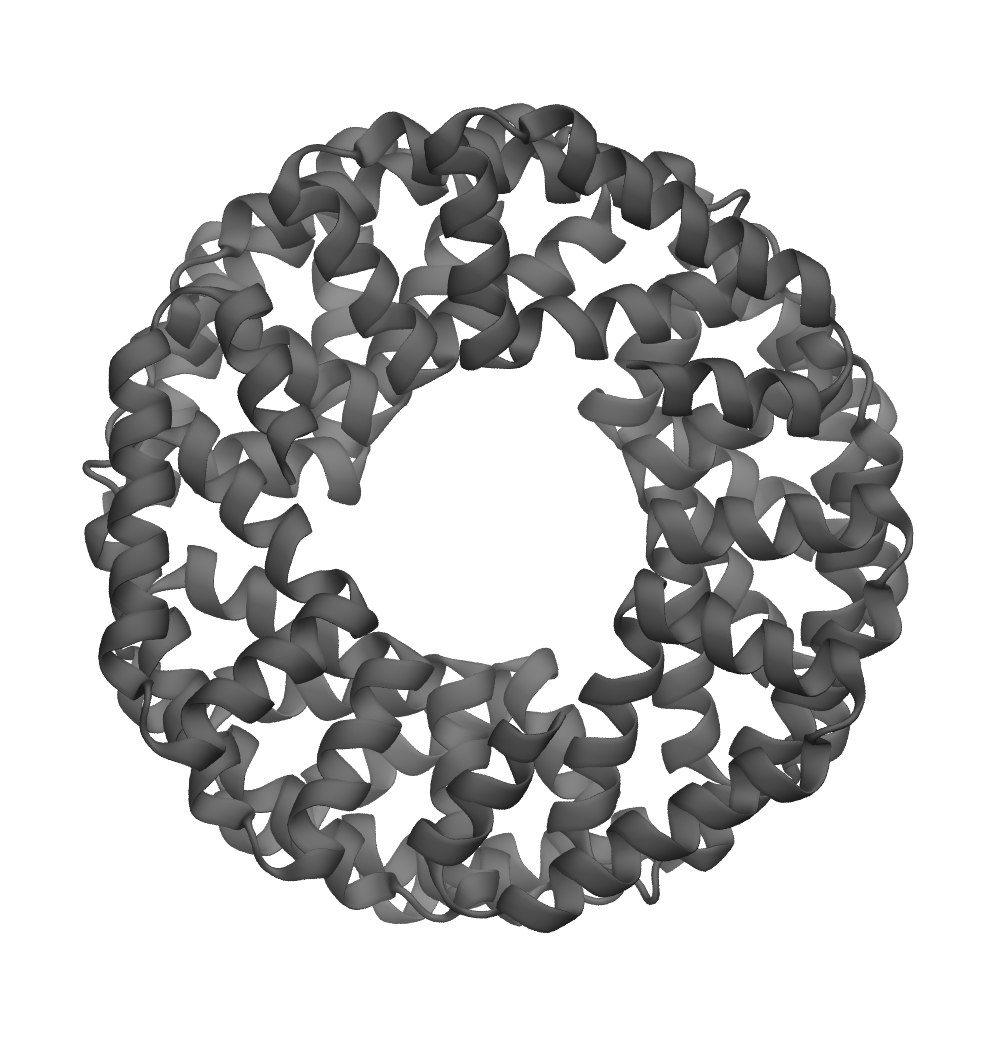

Supplement: Supplementary file 4 — Protein model images, crystal structure images and cryoEM map images. [file 41557_2023_1314_MOESM4_ESM.zip › Figure3/3o52_xtal.jpg]

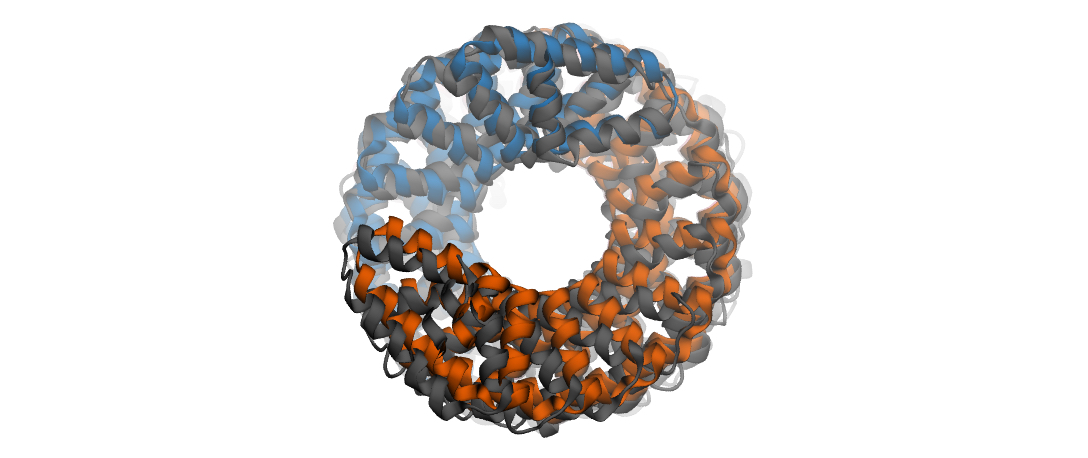

Supplement: Supplementary file 5 — Schematic image, nsEM images, cryoEM map images and protein model images. [file 41557_2023_1314_MOESM5_ESM.zip › Figure4/log44_cryo_top.jpg]

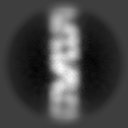

Supplement: Supplementary file 5 — Schematic image, nsEM images, cryoEM map images and protein model images. [file 41557_2023_1314_MOESM5_ESM.zip › Figure4/cryosparc_P220_J199_020_class_averages_3.jpg]

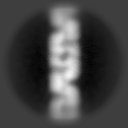

Supplement: Supplementary file 5 — Schematic image, nsEM images, cryoEM map images and protein model images. [file 41557_2023_1314_MOESM5_ESM.zip › Figure4/cryosparc_P220_J199_020_class_averages_2.jpg]

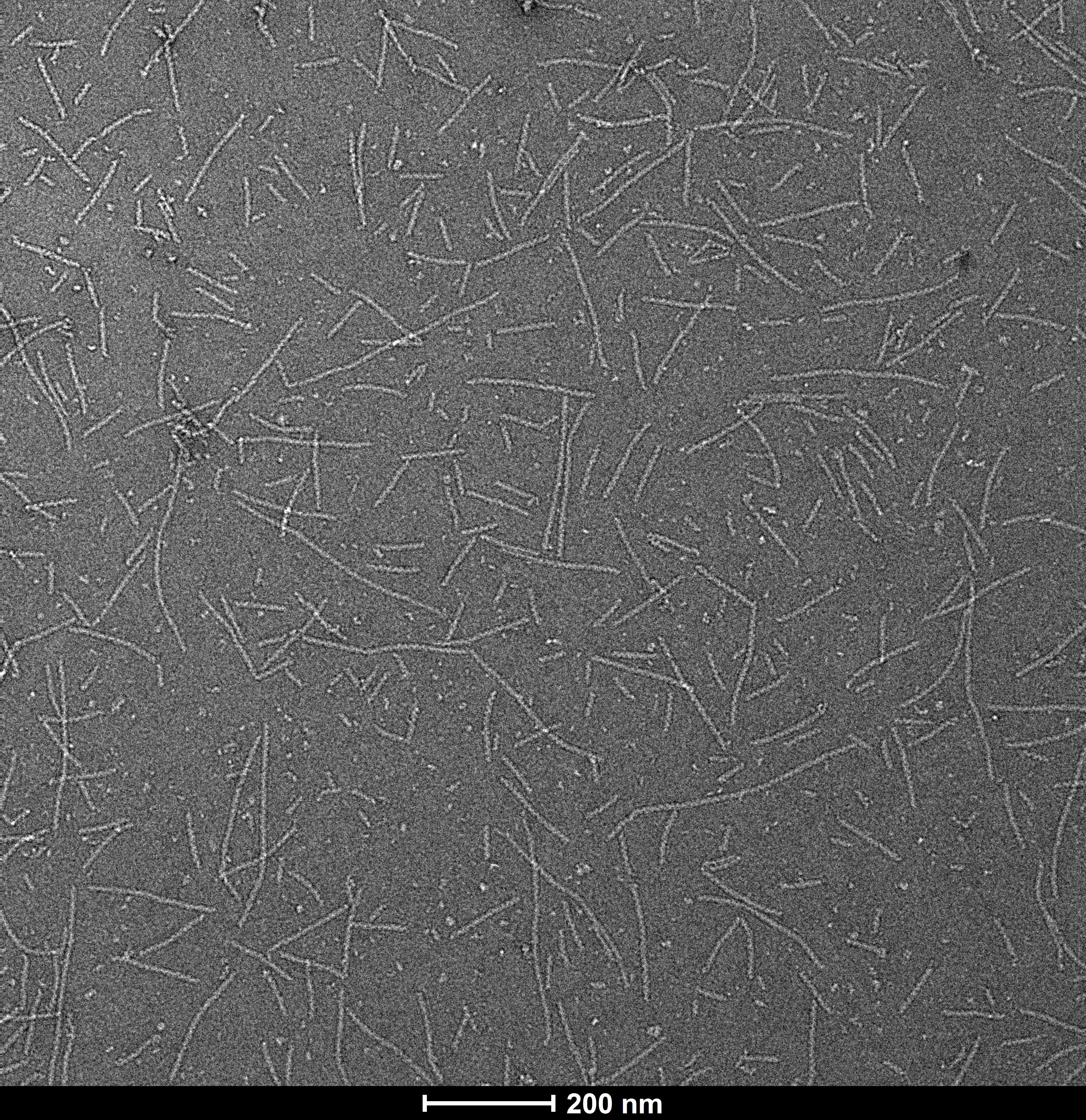

Supplement: Supplementary file 5 — Schematic image, nsEM images, cryoEM map images and protein model images. [file 41557_2023_1314_MOESM5_ESM.zip › Figure4/WB8_4S_log44_0p02mgml_bEx_36k.jpg]

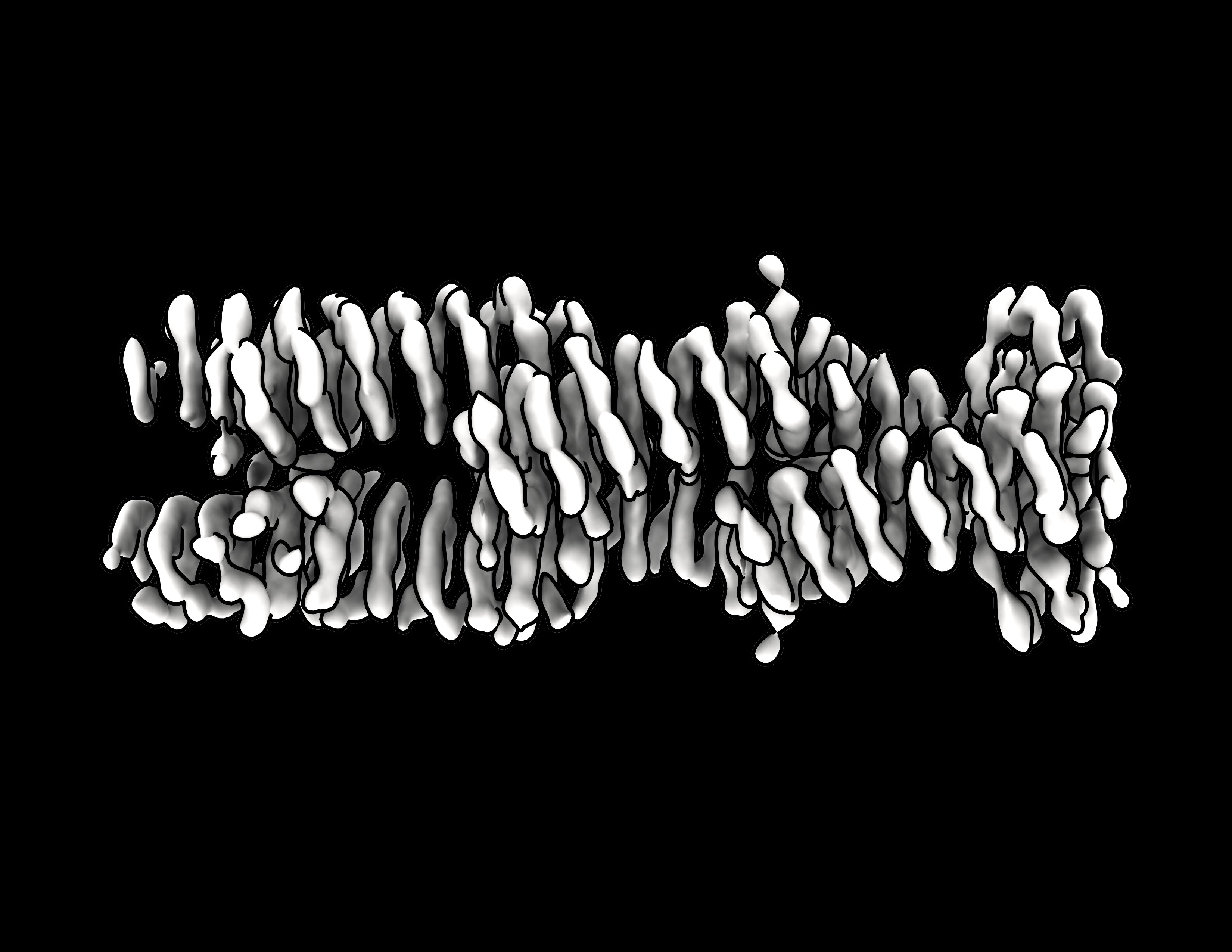

Supplement: Supplementary file 5 — Schematic image, nsEM images, cryoEM map images and protein model images. [file 41557_2023_1314_MOESM5_ESM.zip › Figure4/log41_long.jpg]

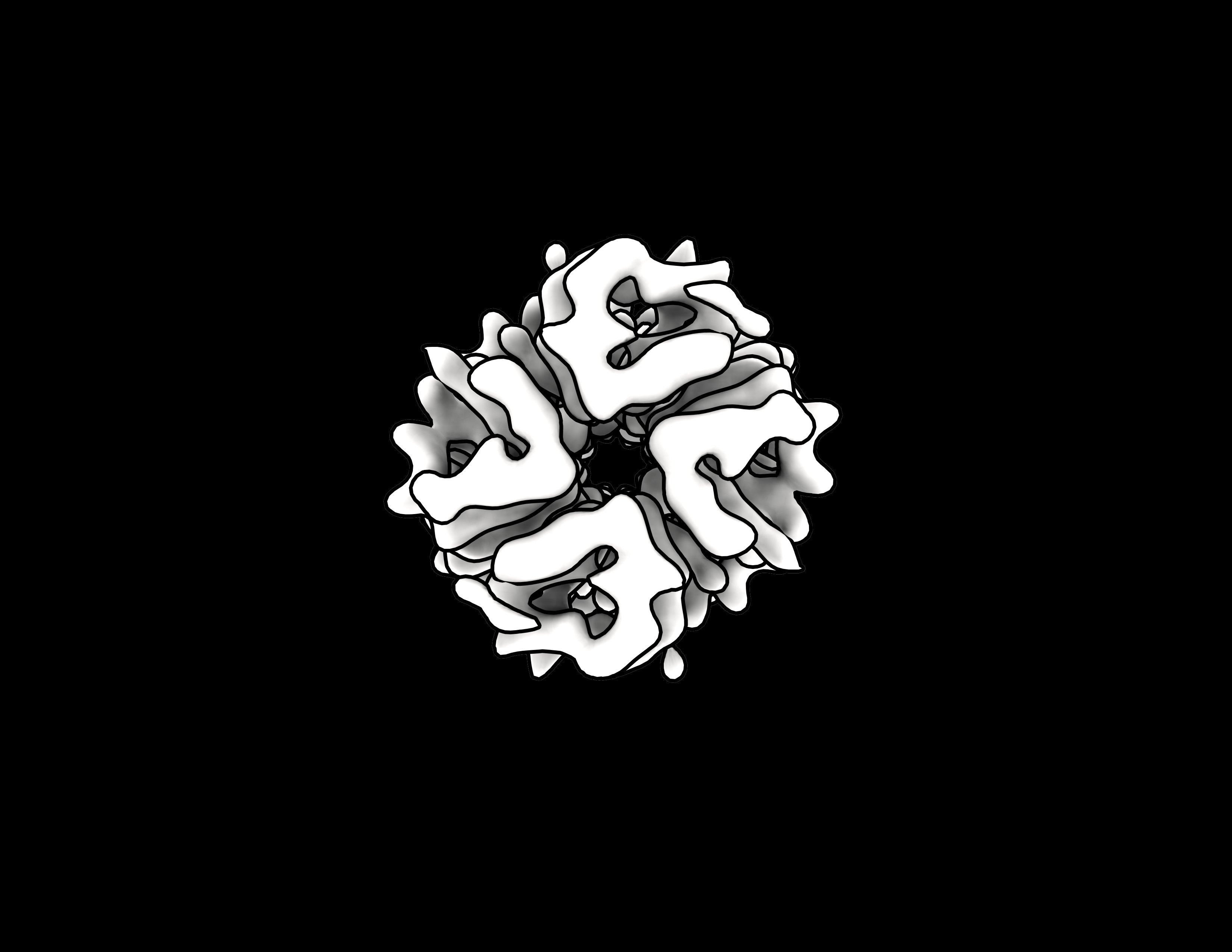

Supplement: Supplementary file 5 — Schematic image, nsEM images, cryoEM map images and protein model images. [file 41557_2023_1314_MOESM5_ESM.zip › Figure4/log41_axis.jpg]

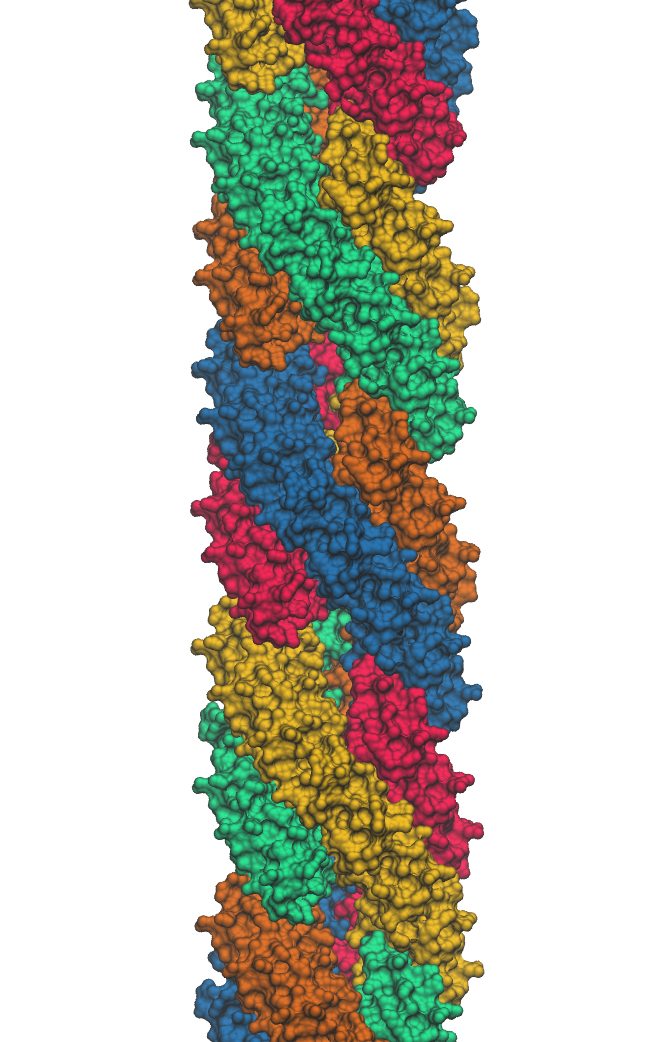

Supplement: Supplementary file 5 — Schematic image, nsEM images, cryoEM map images and protein model images. [file 41557_2023_1314_MOESM5_ESM.zip › Figure4/cap_log107.jpg]

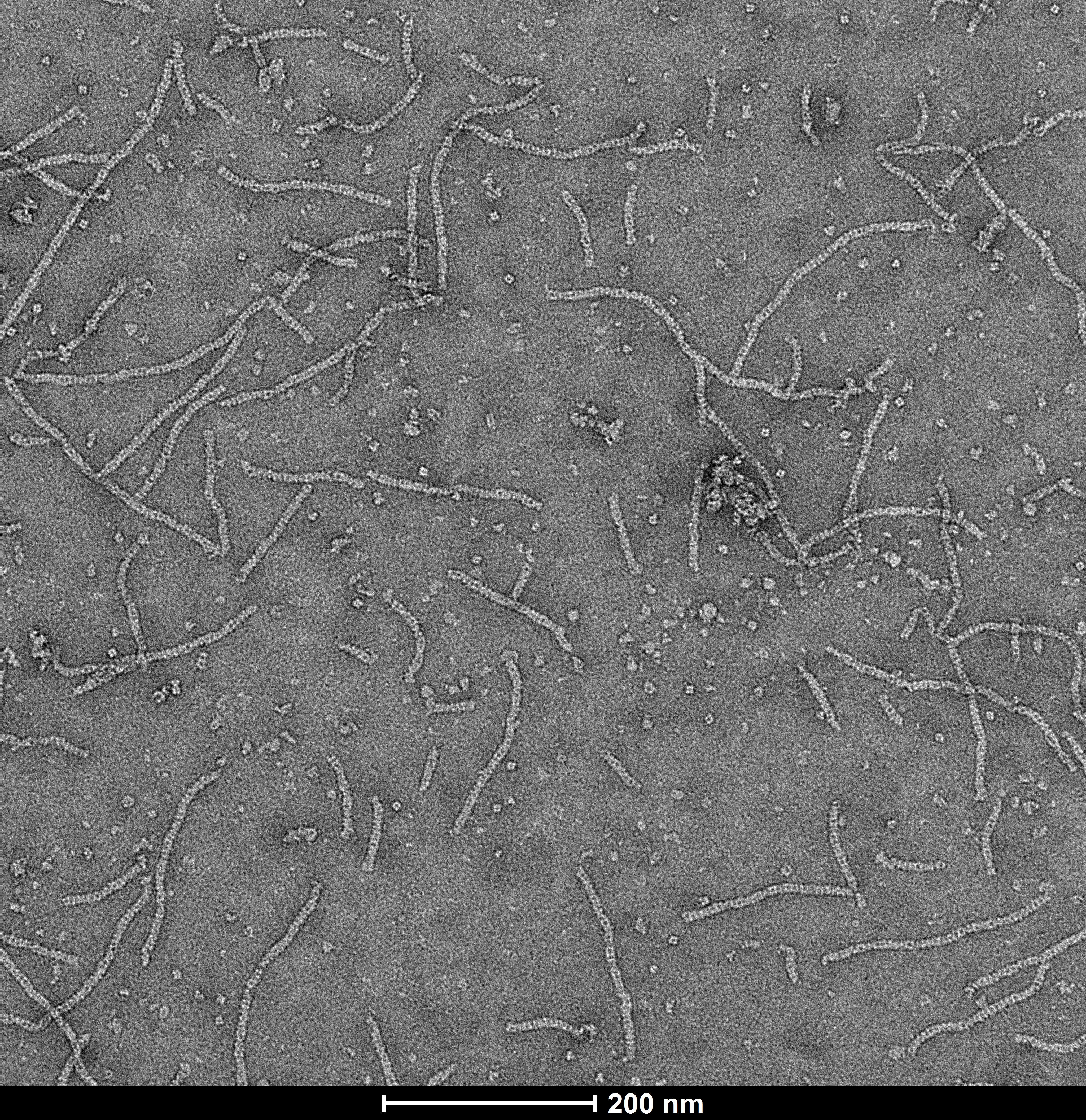

Supplement: Supplementary file 5 — Schematic image, nsEM images, cryoEM map images and protein model images. [file 41557_2023_1314_MOESM5_ESM.zip › Figure4/WB8_5R_log41C_0p02mgml_bEx_36k.jpg]

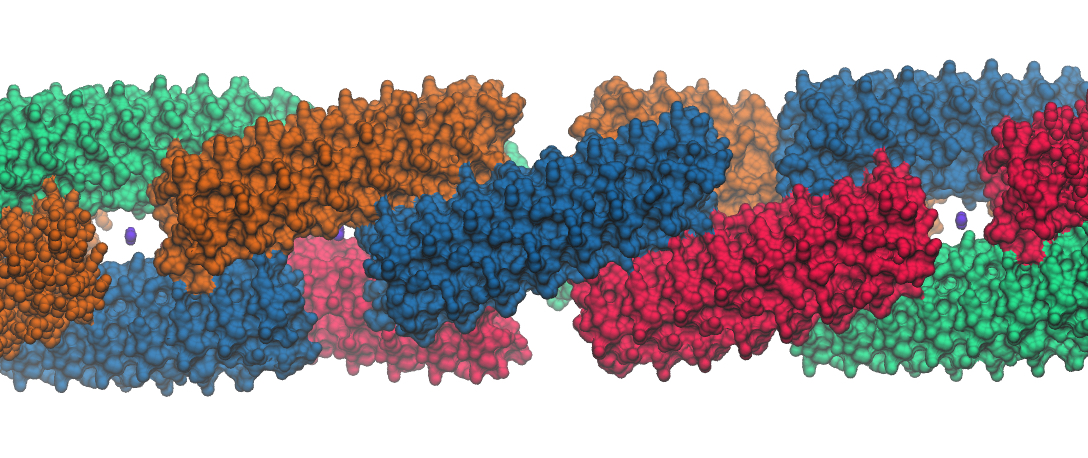

Supplement: Supplementary file 5 — Schematic image, nsEM images, cryoEM map images and protein model images. [file 41557_2023_1314_MOESM5_ESM.zip › Figure4/log41_surf.jpg]

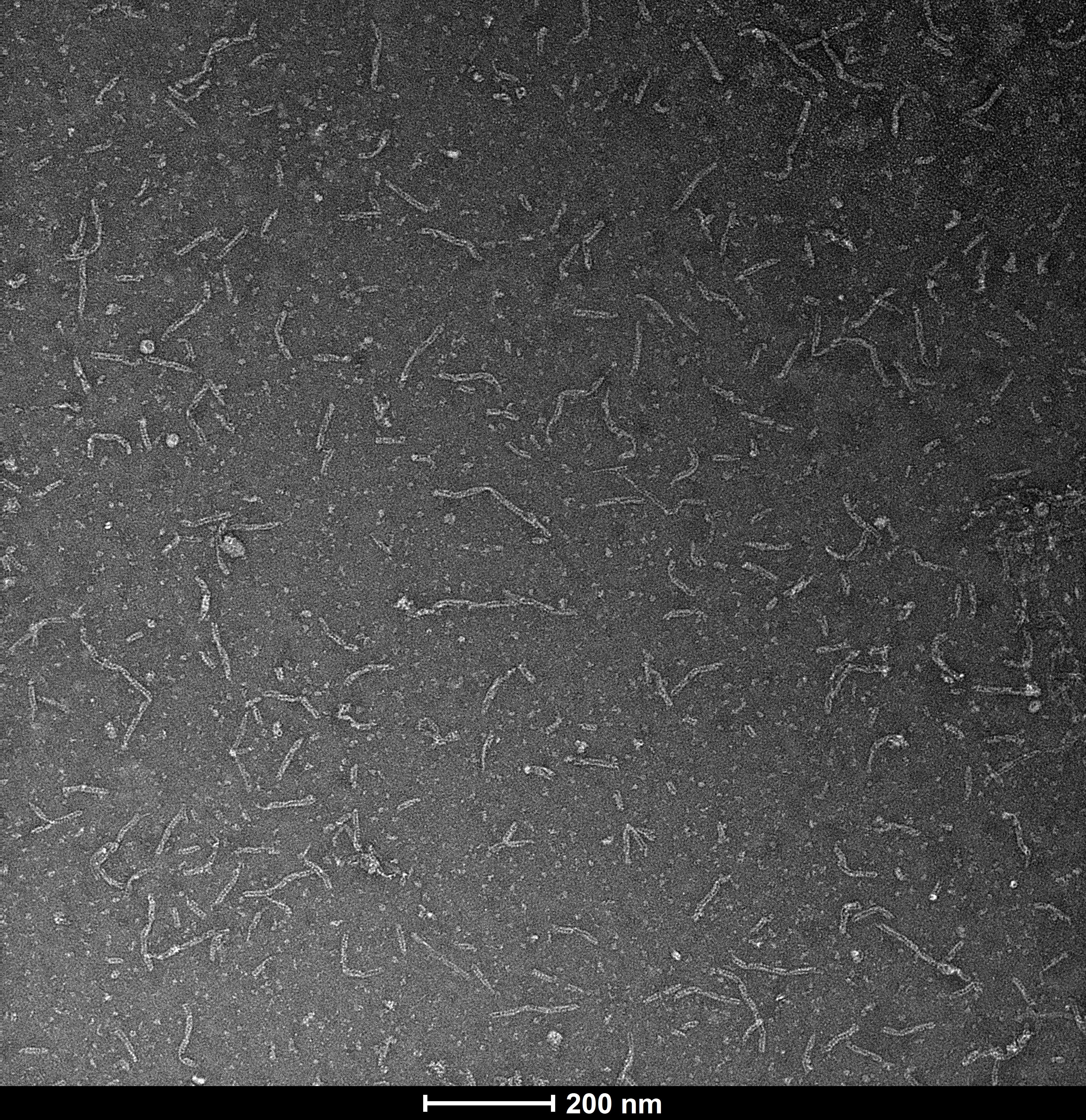

Supplement: Supplementary file 5 — Schematic image, nsEM images, cryoEM map images and protein model images. [file 41557_2023_1314_MOESM5_ESM.zip › Figure4/WB8_6O_log47_bEx_0p06mgml_36k.jpg]

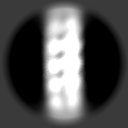

Supplement: Supplementary file 5 — Schematic image, nsEM images, cryoEM map images and protein model images. [file 41557_2023_1314_MOESM5_ESM.zip › Figure4/cryosparc_P240_J7_020_class_averages_1.jpg]

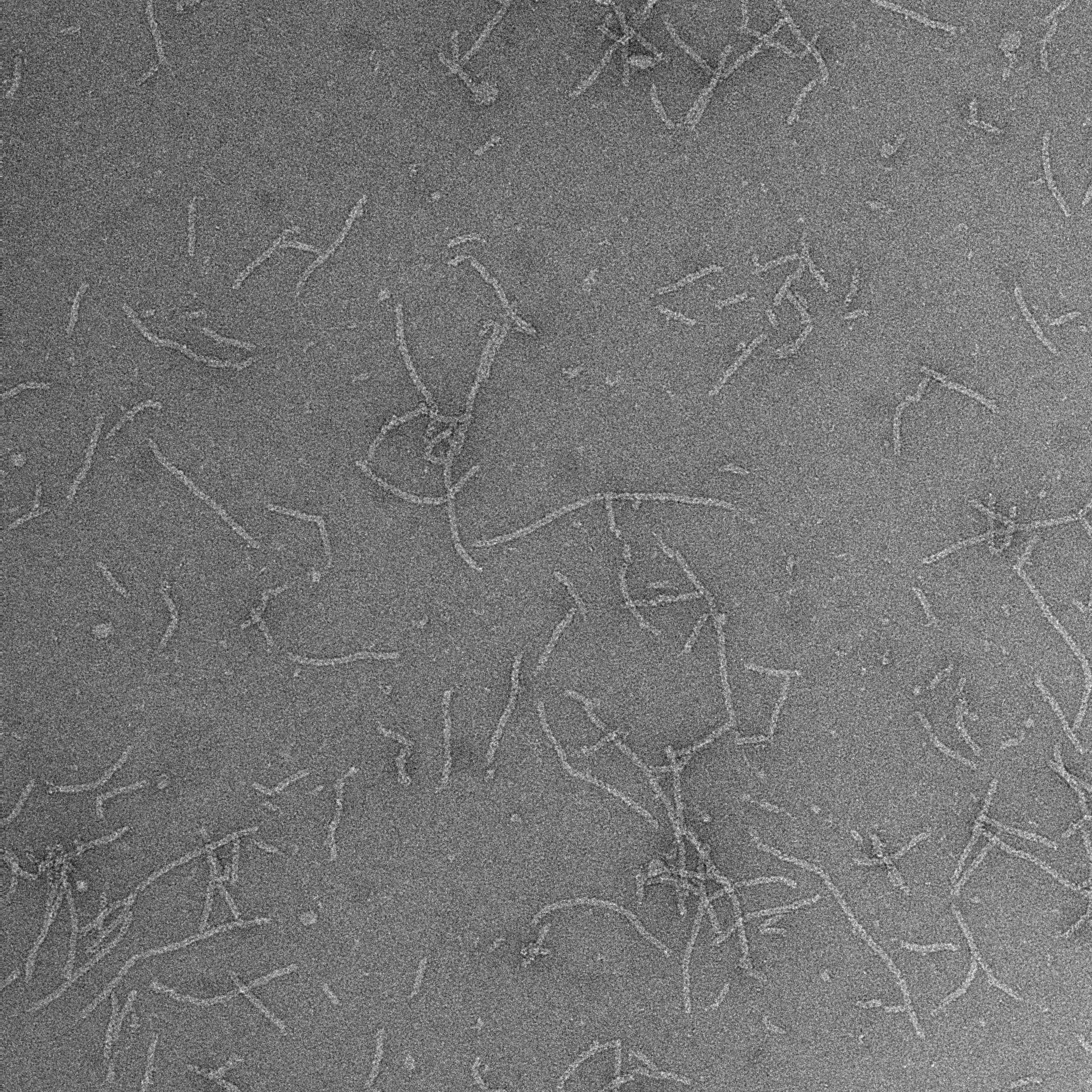

Supplement: Supplementary file 5 — Schematic image, nsEM images, cryoEM map images and protein model images. [file 41557_2023_1314_MOESM5_ESM.zip › Figure4/FoilHole_24634064_Data_24632561_24632563_20220824_135740.jpg]

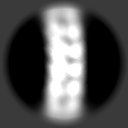

Supplement: Supplementary file 5 — Schematic image, nsEM images, cryoEM map images and protein model images. [file 41557_2023_1314_MOESM5_ESM.zip › Figure4/cryosparc_P240_J7_020_class_averages_2.jpg]

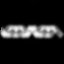

Supplement: Supplementary file 5 — Schematic image, nsEM images, cryoEM map images and protein model images. [file 41557_2023_1314_MOESM5_ESM.zip › Figure4/cryosparc_P219_J196_templates (1).jpg]

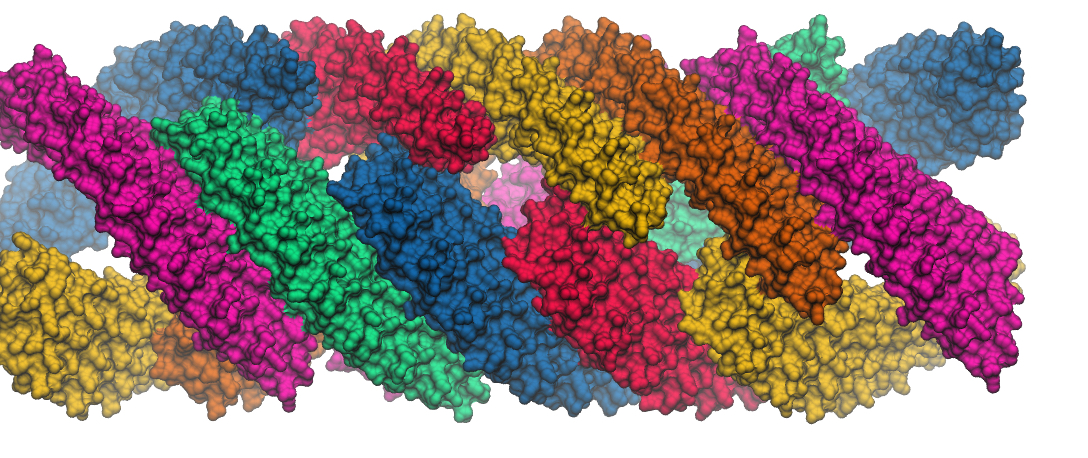

Supplement: Supplementary file 5 — Schematic image, nsEM images, cryoEM map images and protein model images. [file 41557_2023_1314_MOESM5_ESM.zip › Figure4/log47_surf.jpg]

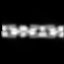

Supplement: Supplementary file 5 — Schematic image, nsEM images, cryoEM map images and protein model images. [file 41557_2023_1314_MOESM5_ESM.zip › Figure4/cryosparc_P219_J198_templates.jpg]

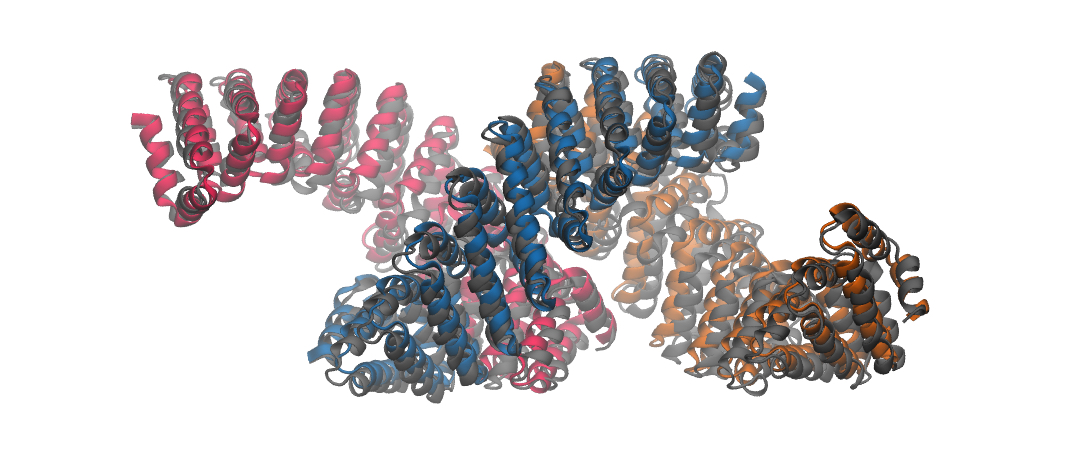

Supplement: Supplementary file 5 — Schematic image, nsEM images, cryoEM map images and protein model images. [file 41557_2023_1314_MOESM5_ESM.zip › Figure4/log44_cryo.jpg]

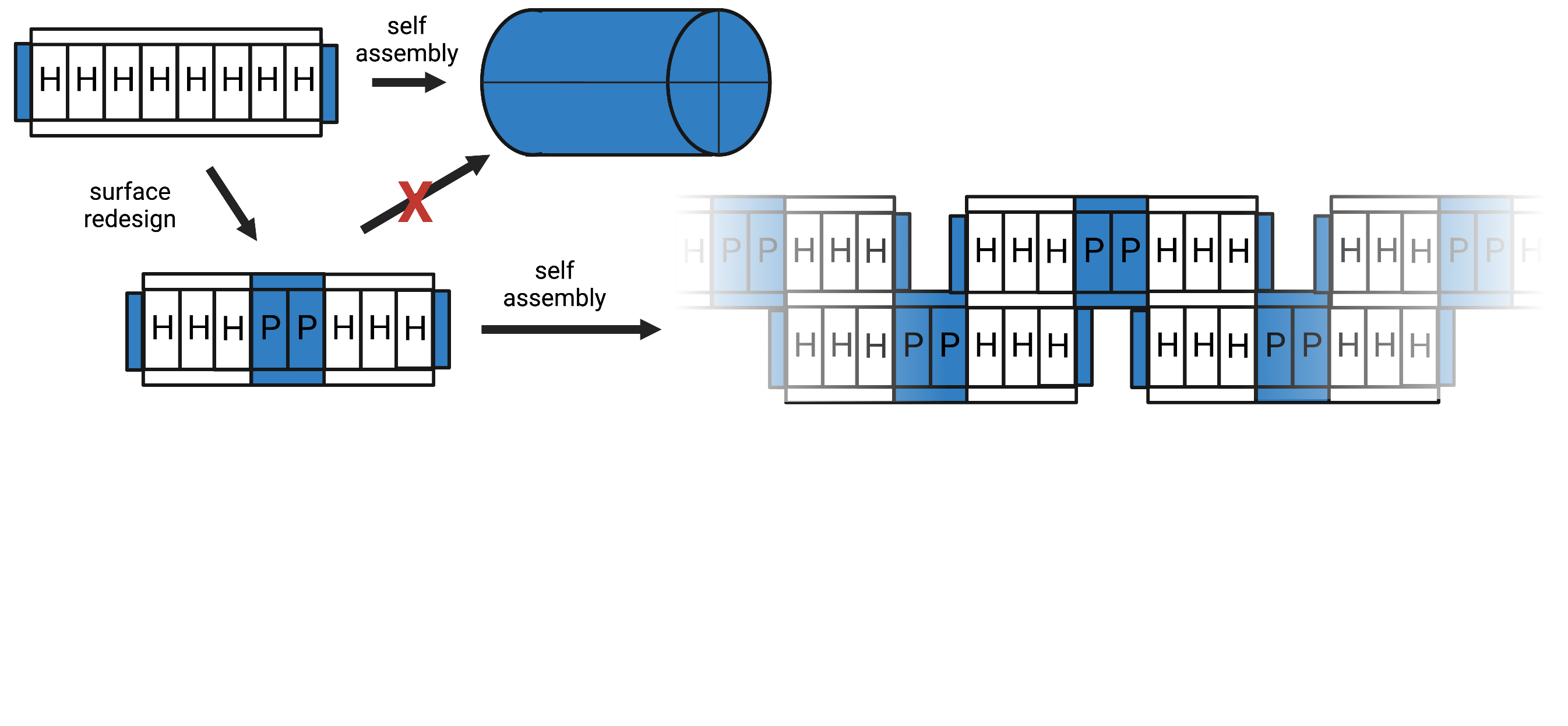

Supplement: Supplementary file 5 — Schematic image, nsEM images, cryoEM map images and protein model images. [file 41557_2023_1314_MOESM5_ESM.zip › Figure4/Fiber_schematic.tiff]

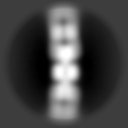

Supplement: Supplementary file 5 — Schematic image, nsEM images, cryoEM map images and protein model images. [file 41557_2023_1314_MOESM5_ESM.zip › Figure4/cryosparc_P221_J128_020_class_averages_2.jpg]

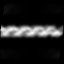

Supplement: Supplementary file 5 — Schematic image, nsEM images, cryoEM map images and protein model images. [file 41557_2023_1314_MOESM5_ESM.zip › Figure4/cryosparc_P219_J200_templates (1).jpg]

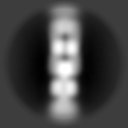

Supplement: Supplementary file 5 — Schematic image, nsEM images, cryoEM map images and protein model images. [file 41557_2023_1314_MOESM5_ESM.zip › Figure4/cryosparc_P221_J128_020_class_averages_1.jpg]

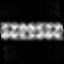

Supplement: Supplementary file 5 — Schematic image, nsEM images, cryoEM map images and protein model images. [file 41557_2023_1314_MOESM5_ESM.zip › Figure4/cryosparc_P219_J202_templates.jpg]

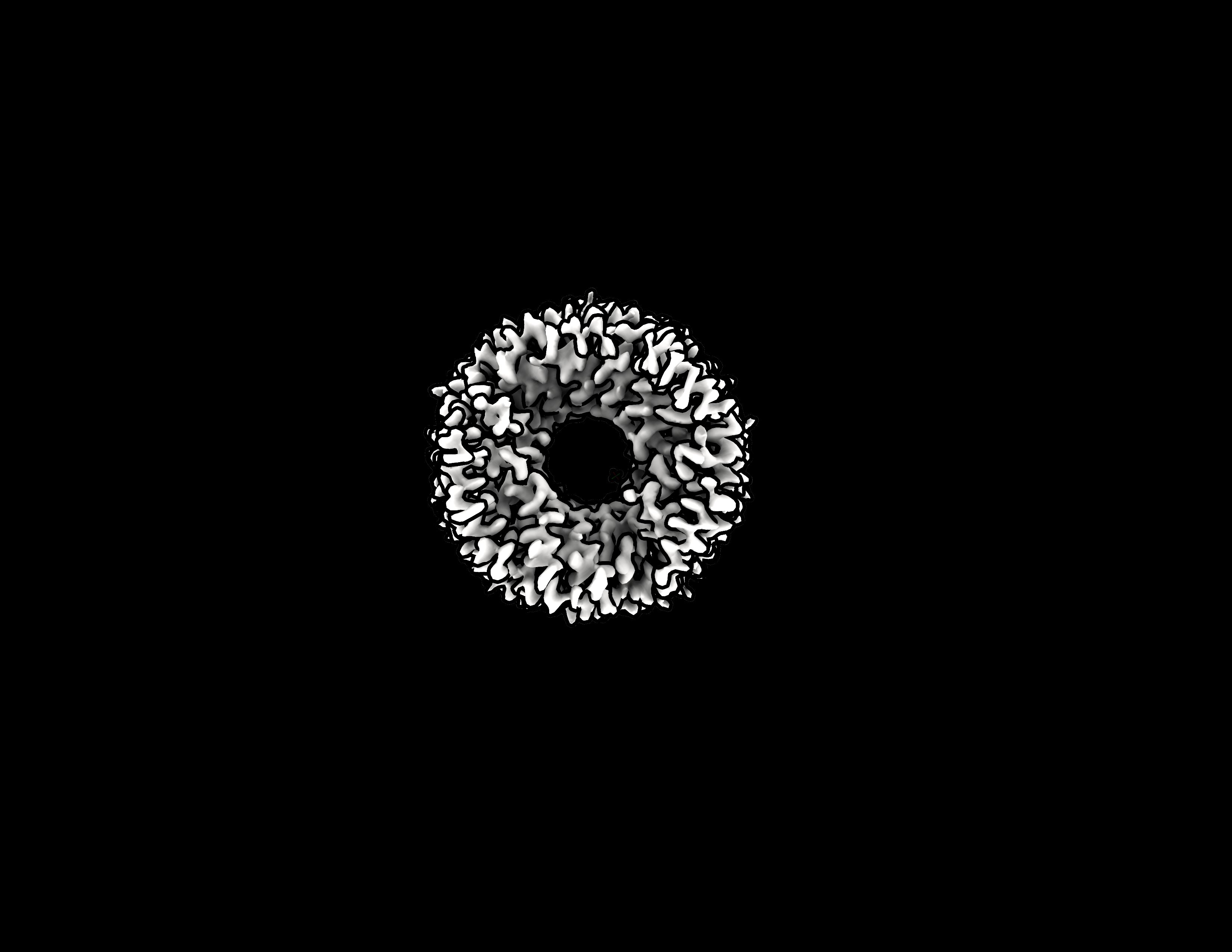

Supplement: Supplementary file 5 — Schematic image, nsEM images, cryoEM map images and protein model images. [file 41557_2023_1314_MOESM5_ESM.zip › Figure4/log44_axis.jpg]

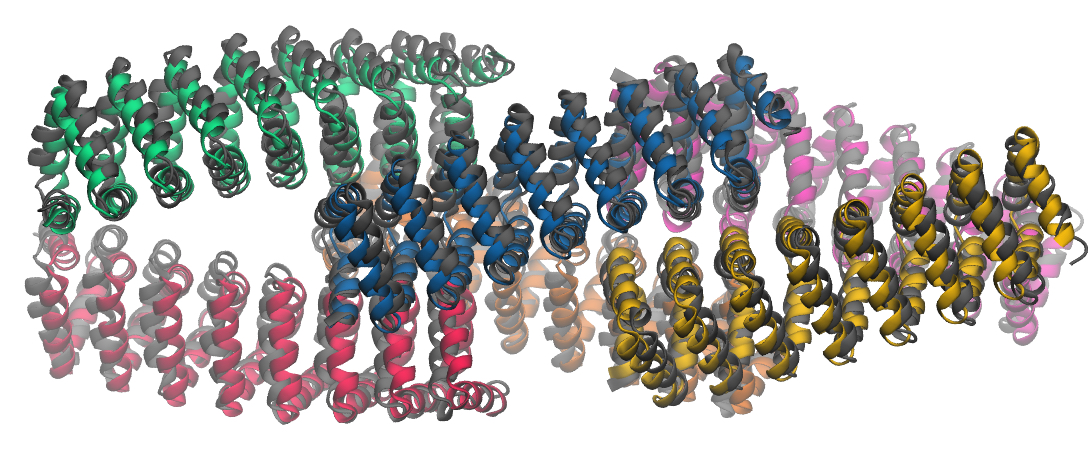

Supplement: Supplementary file 5 — Schematic image, nsEM images, cryoEM map images and protein model images. [file 41557_2023_1314_MOESM5_ESM.zip › Figure4/log41_cryo_side.jpg]

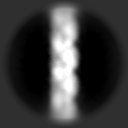

Supplement: Supplementary file 5 — Schematic image, nsEM images, cryoEM map images and protein model images. [file 41557_2023_1314_MOESM5_ESM.zip › Figure4/cryosparc_P232_J13_020_class_averages_1.jpg]

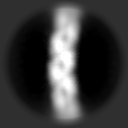

Supplement: Supplementary file 5 — Schematic image, nsEM images, cryoEM map images and protein model images. [file 41557_2023_1314_MOESM5_ESM.zip › Figure4/cryosparc_P232_J13_020_class_averages_3.jpg]

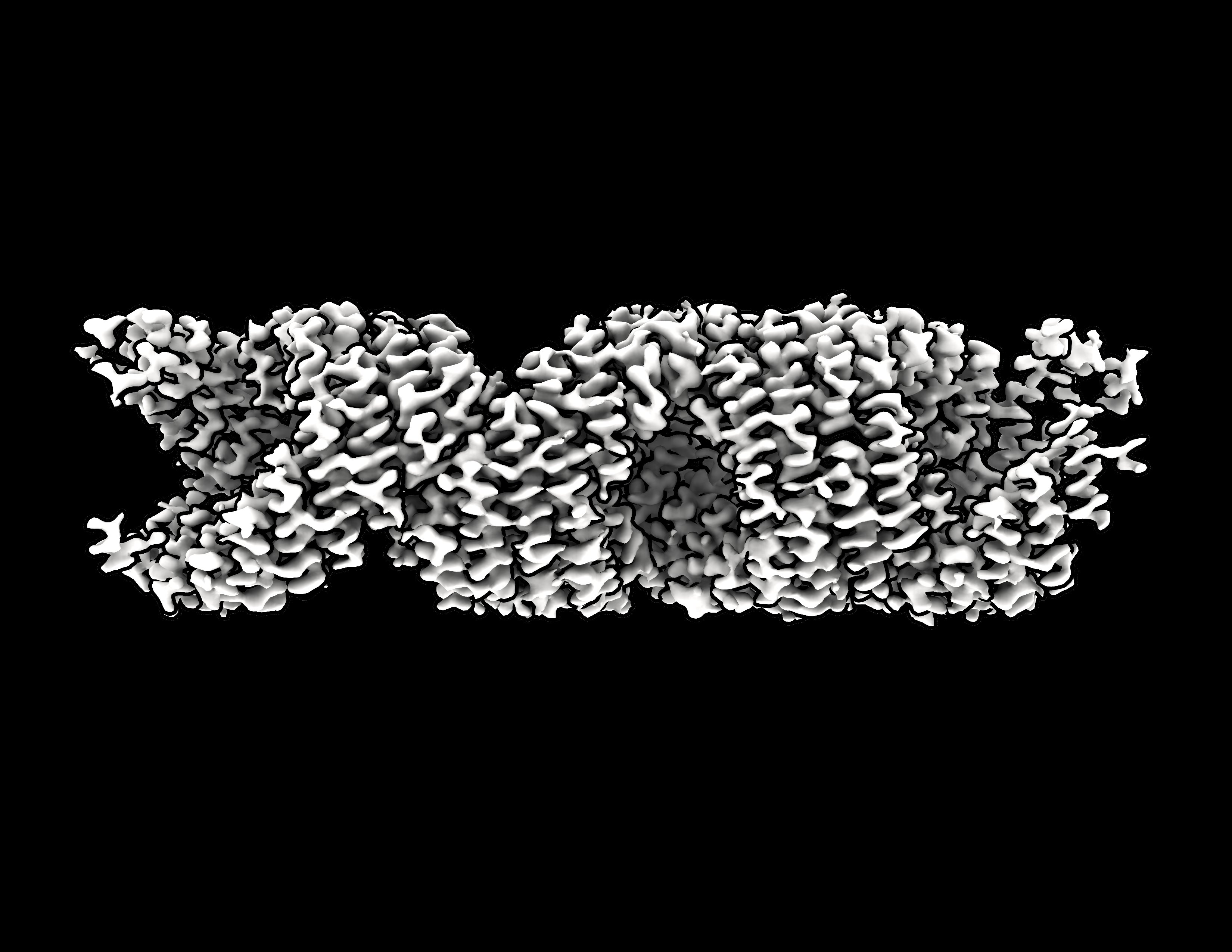

Supplement: Supplementary file 5 — Schematic image, nsEM images, cryoEM map images and protein model images. [file 41557_2023_1314_MOESM5_ESM.zip › Figure4/log44_long.jpg]

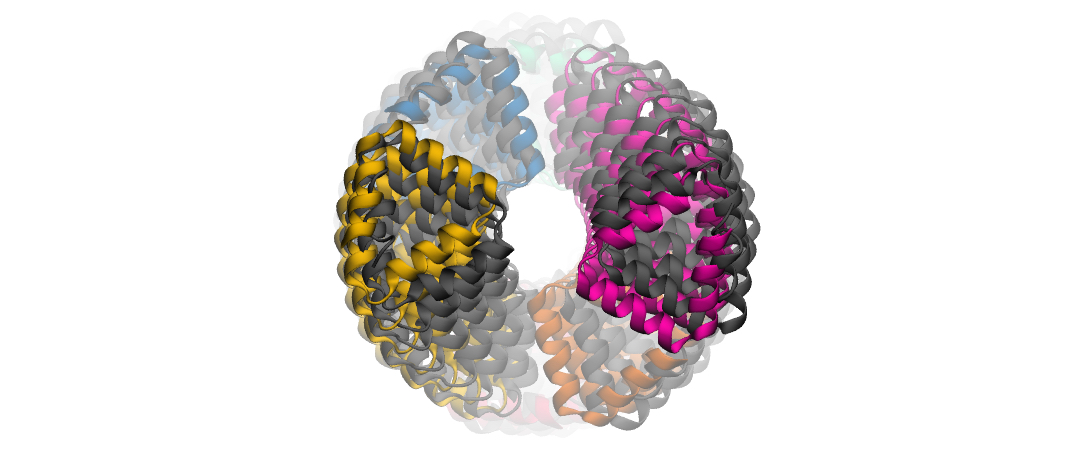

Supplement: Supplementary file 5 — Schematic image, nsEM images, cryoEM map images and protein model images. [file 41557_2023_1314_MOESM5_ESM.zip › Figure4/log41_cryo_top.jpg]

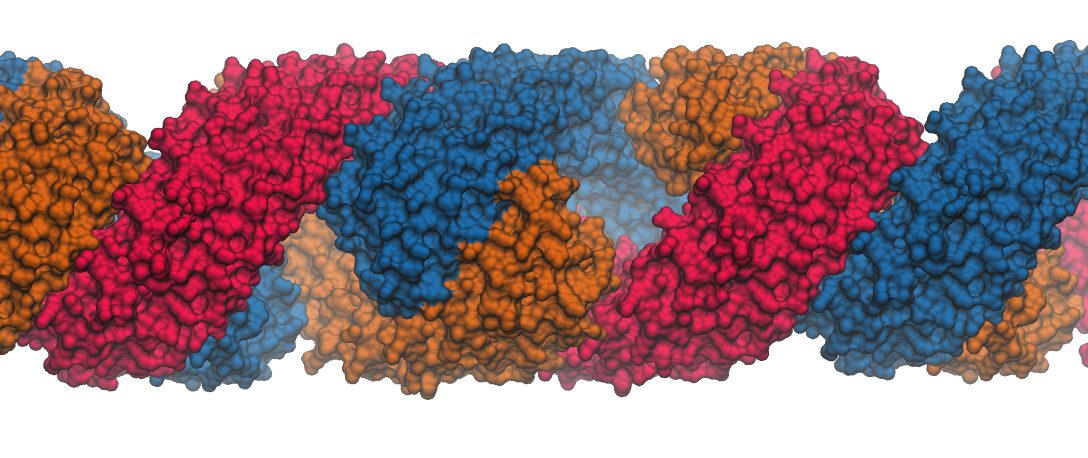

Supplement: Supplementary file 5 — Schematic image, nsEM images, cryoEM map images and protein model images. [file 41557_2023_1314_MOESM5_ESM.zip › Figure4/log44_surf.jpg]

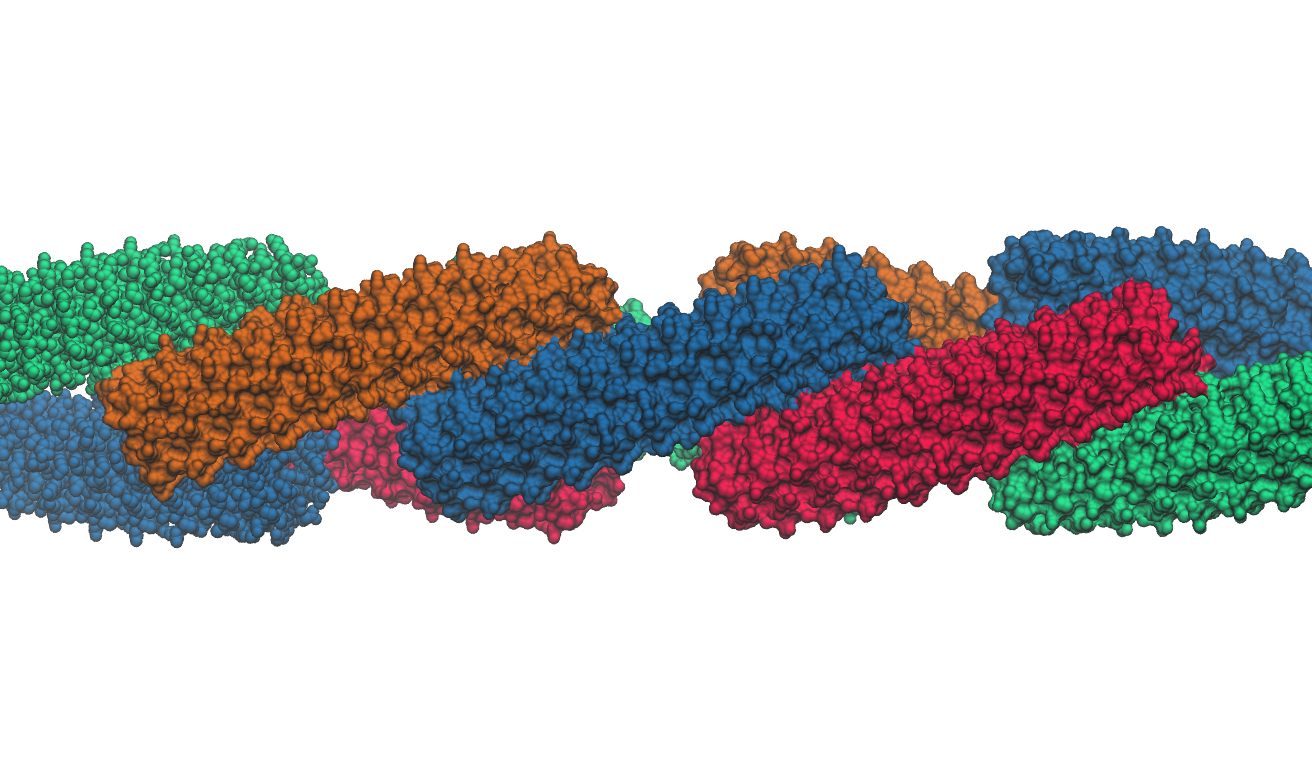

Supplement: Supplementary file 6 — Schematic image, nsEM images and persistence length source data. [file 41557_2023_1314_MOESM6_ESM.zip › Figure5/log167.jpg]

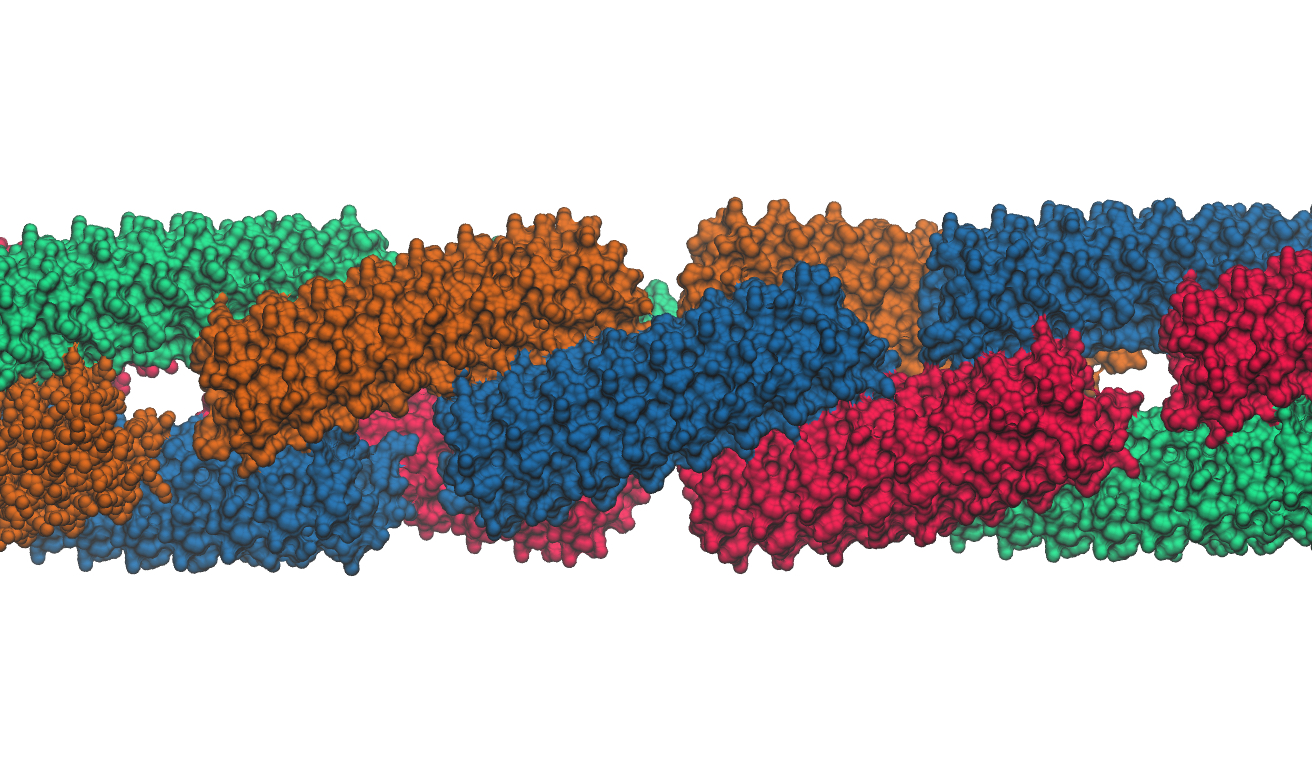

Supplement: Supplementary file 6 — Schematic image, nsEM images and persistence length source data. [file 41557_2023_1314_MOESM6_ESM.zip › Figure5/log166.jpg]

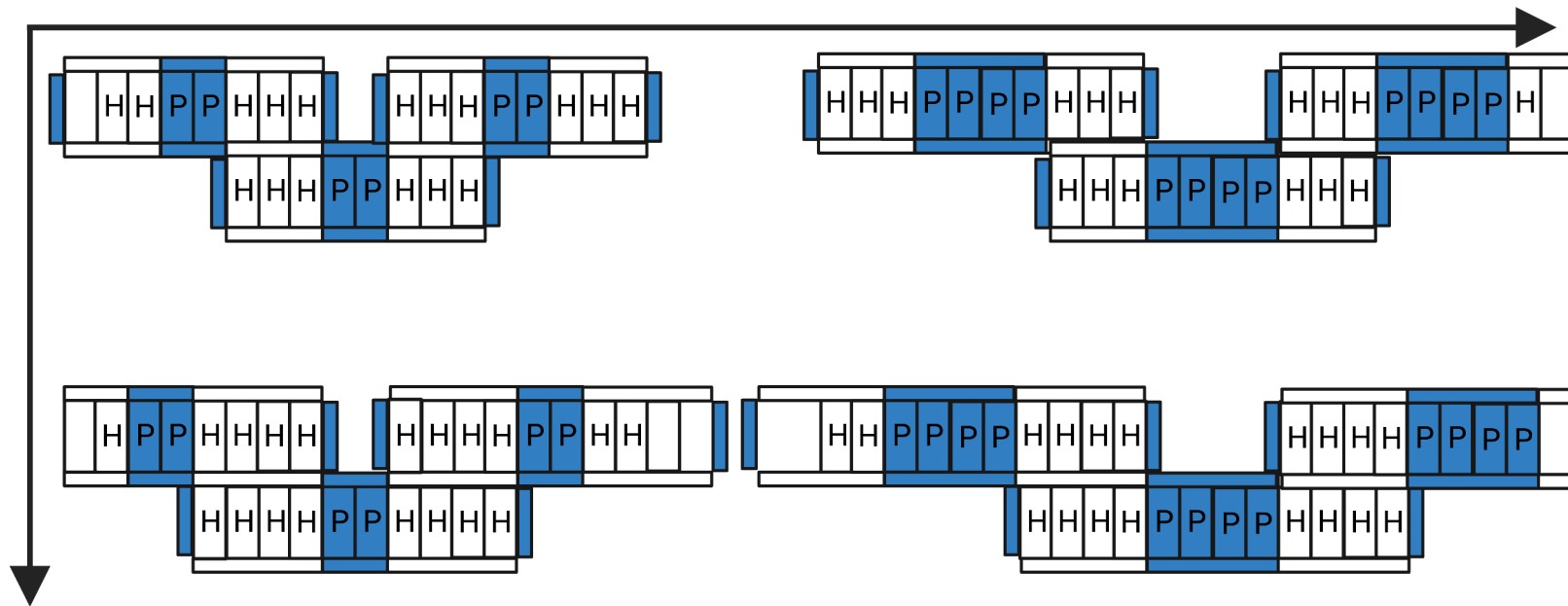

Supplement: Supplementary file 6 — Schematic image, nsEM images and persistence length source data. [file 41557_2023_1314_MOESM6_ESM.zip › Figure5/FiberSchematic.pdf]

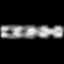

Supplement: Supplementary file 6 — Schematic image, nsEM images and persistence length source data. [file 41557_2023_1314_MOESM6_ESM.zip › Figure5/cryosparc_P219_cryosparc_P219_J208_templates.jpg]

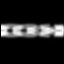

Supplement: Supplementary file 6 — Schematic image, nsEM images and persistence length source data. [file 41557_2023_1314_MOESM6_ESM.zip › Figure5/cryosparc_P219_J238_templates (1).jpg]

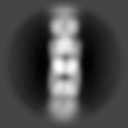

Supplement: Supplementary file 6 — Schematic image, nsEM images and persistence length source data. [file 41557_2023_1314_MOESM6_ESM.zip › Figure5/cryosparc_P236_J11_020_class_averages_1.jpg]
