# Supplementary figures and images for: Precisely patterned nanofibres made from extendable protein multiplexes (part 2 of 2)
Source: Nat Chem. 2023 Sep 4;15(12):1664–71. doi: 10.1038/s41557-023-01314-x (PMC10695826; doi:10.1038/s41557-023-01314-x)

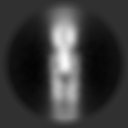

Supplement: Supplementary file 6 — Schematic image, nsEM images and persistence length source data. [file 41557_2023_1314_MOESM6_ESM.zip › Figure5/cryosparc_P239_J32_020_class_averages_2.jpg]

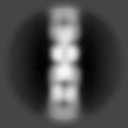

Supplement: Supplementary file 6 — Schematic image, nsEM images and persistence length source data. [file 41557_2023_1314_MOESM6_ESM.zip › Figure5/cryosparc_P236_J11_020_class_averages_2.jpg]

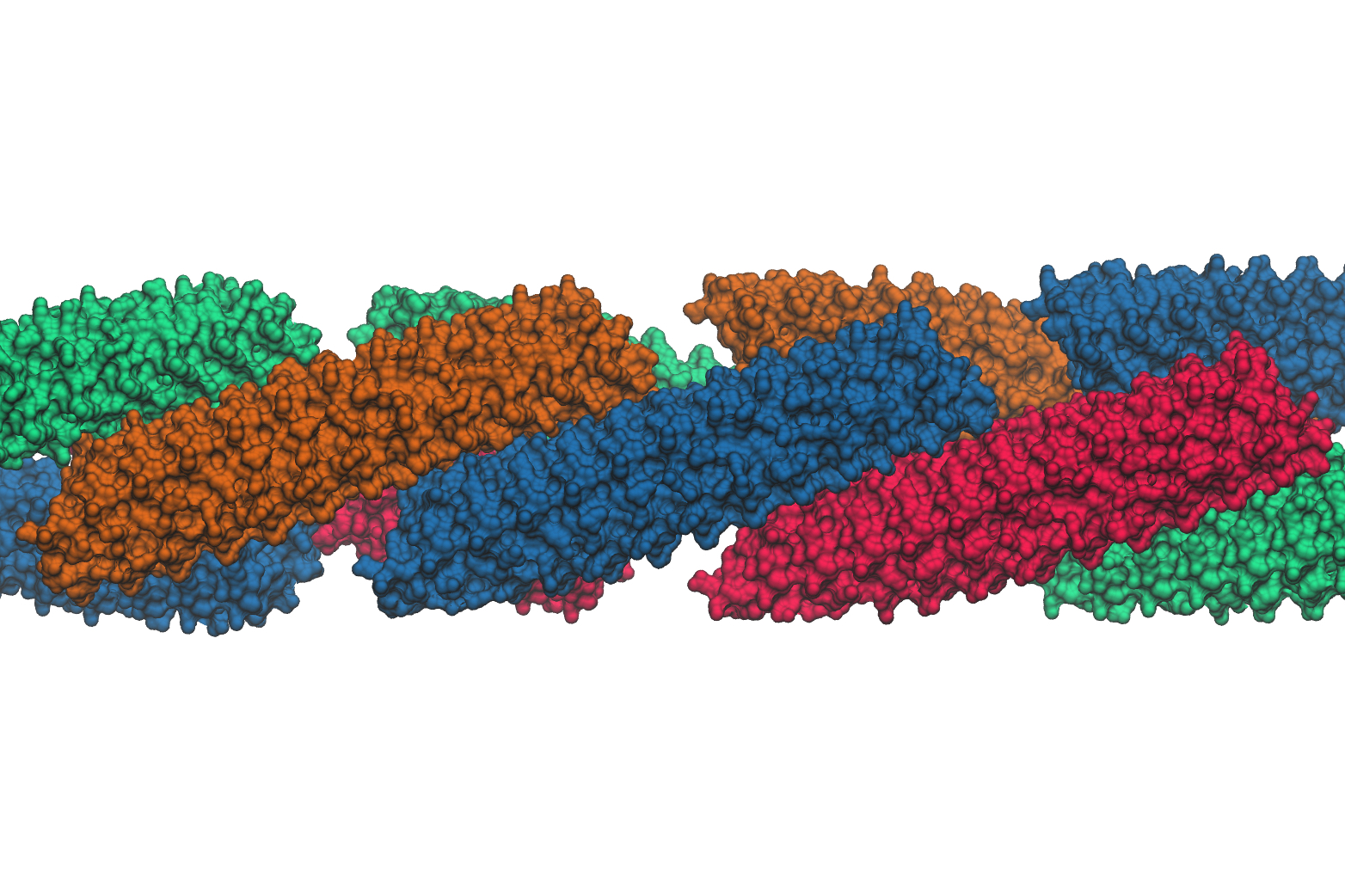

Supplement: Supplementary file 6 — Schematic image, nsEM images and persistence length source data. [file 41557_2023_1314_MOESM6_ESM.zip › Figure5/cap_log156.jpg]

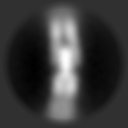

Supplement: Supplementary file 6 — Schematic image, nsEM images and persistence length source data. [file 41557_2023_1314_MOESM6_ESM.zip › Figure5/cryosparc_P239_J32_020_class_averages_1.jpg]

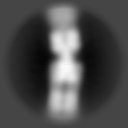

Supplement: Supplementary file 6 — Schematic image, nsEM images and persistence length source data. [file 41557_2023_1314_MOESM6_ESM.zip › Figure5/cryosparc_P246_J6_020_class_averages_2.jpg]

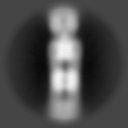

Supplement: Supplementary file 6 — Schematic image, nsEM images and persistence length source data. [file 41557_2023_1314_MOESM6_ESM.zip › Figure5/cryosparc_P246_J6_020_class_averages.jpg]

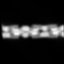

Supplement: Supplementary file 6 — Schematic image, nsEM images and persistence length source data. [file 41557_2023_1314_MOESM6_ESM.zip › Figure5/cryosparc_P219_cryosparc_P219_J204_templates.jpg]

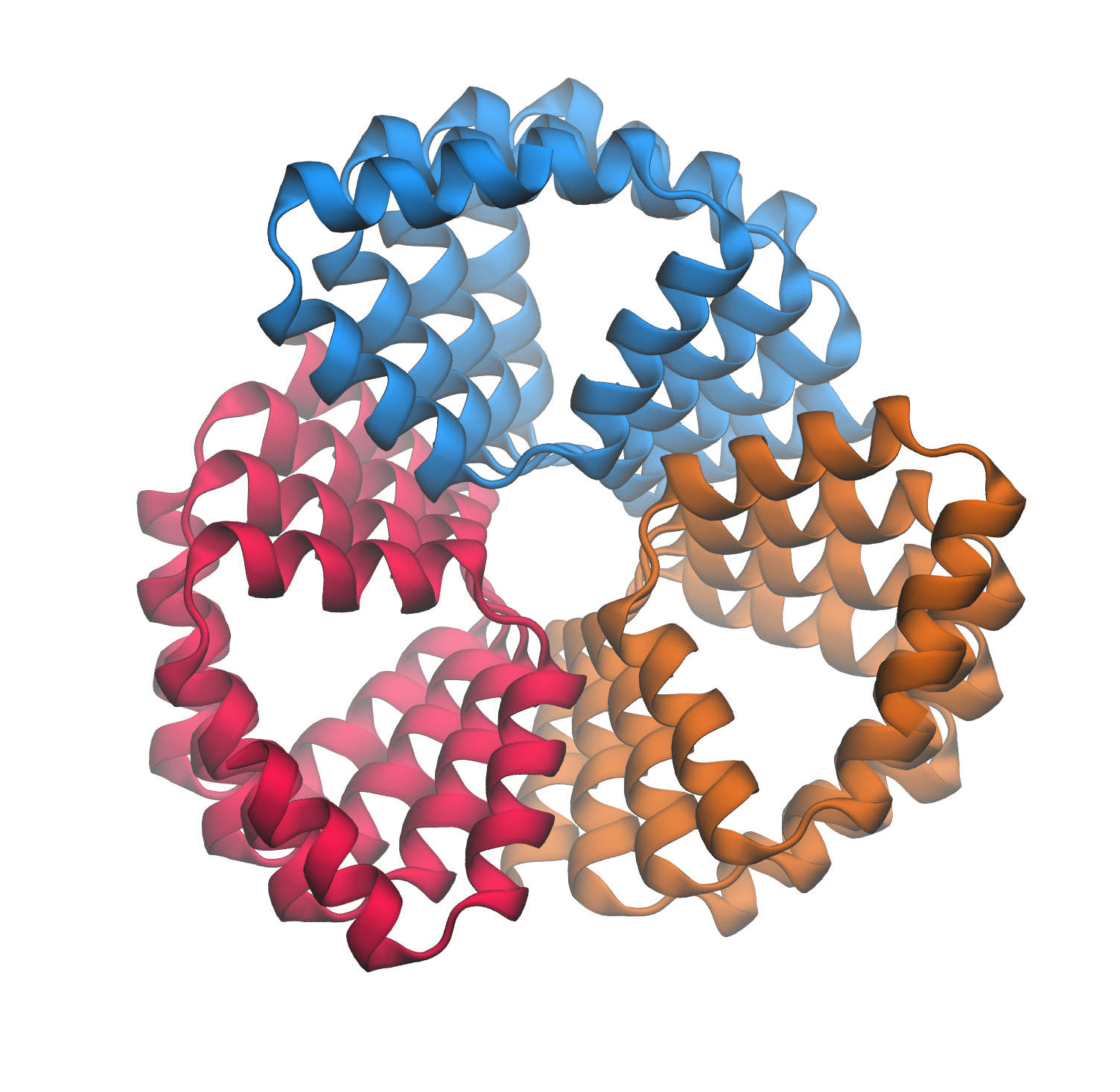

Supplement: Supplementary file 7 — Protein images, SEC source data and SAXS source data. [file 41557_2023_1314_MOESM7_ESM.zip › ExtendedDataFigure1/3o27.jpg]

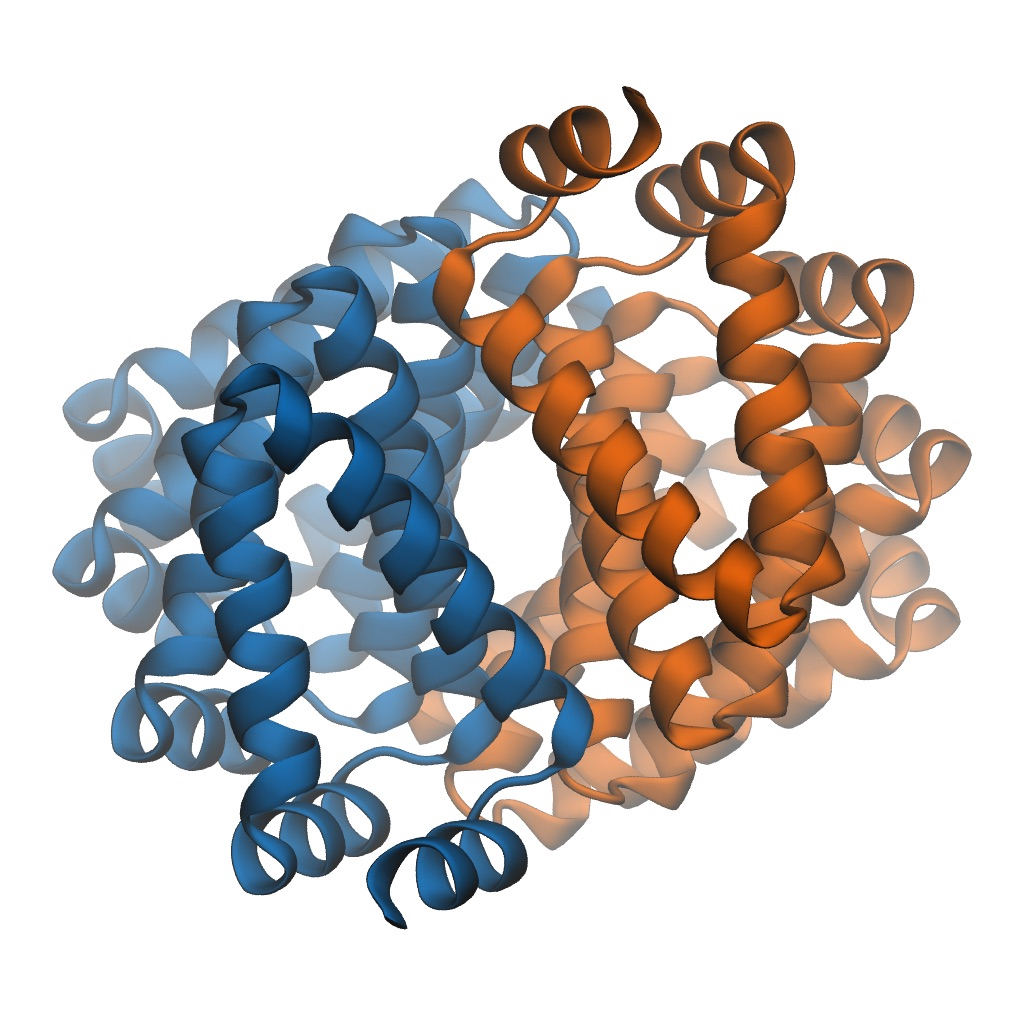

Supplement: Supplementary file 7 — Protein images, SEC source data and SAXS source data. [file 41557_2023_1314_MOESM7_ESM.zip › ExtendedDataFigure1/3d11.jpg]

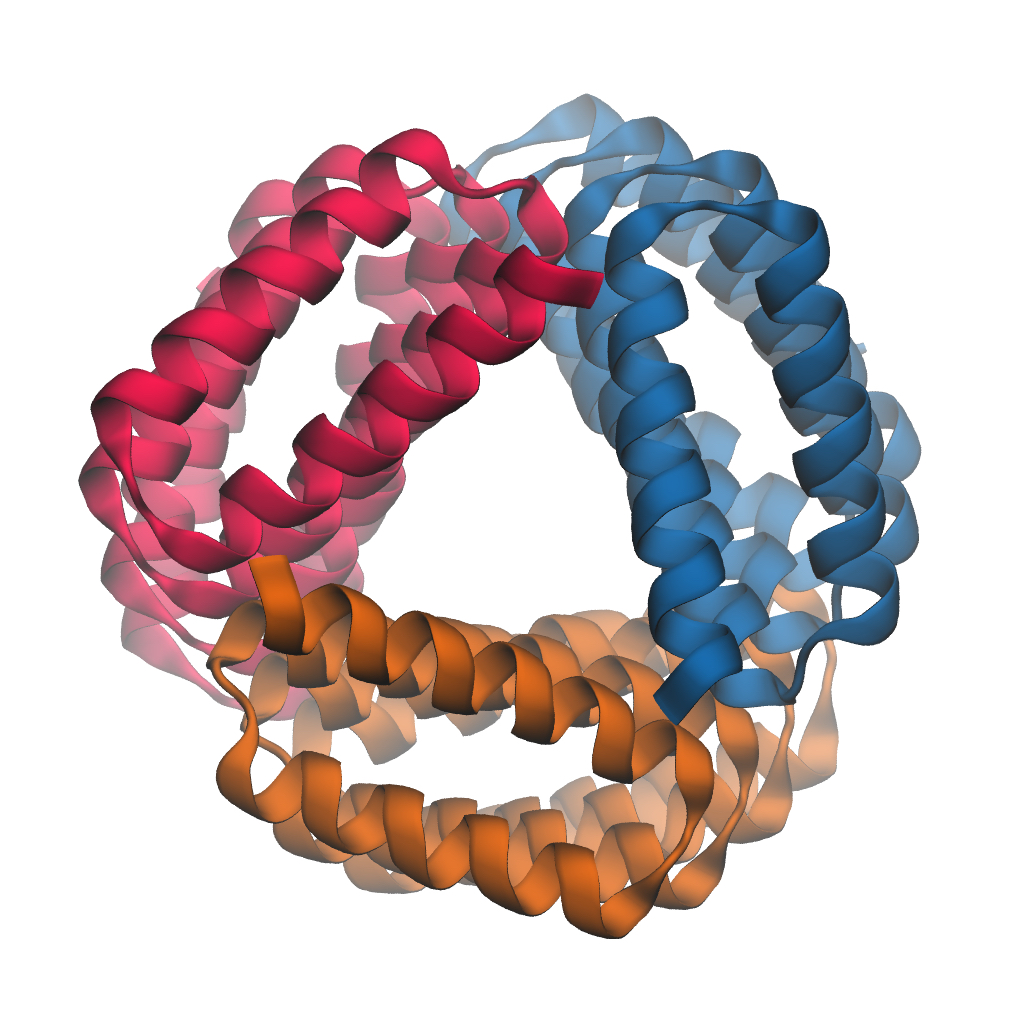

Supplement: Supplementary file 7 — Protein images, SEC source data and SAXS source data. [file 41557_2023_1314_MOESM7_ESM.zip › ExtendedDataFigure1/3o76.jpg]

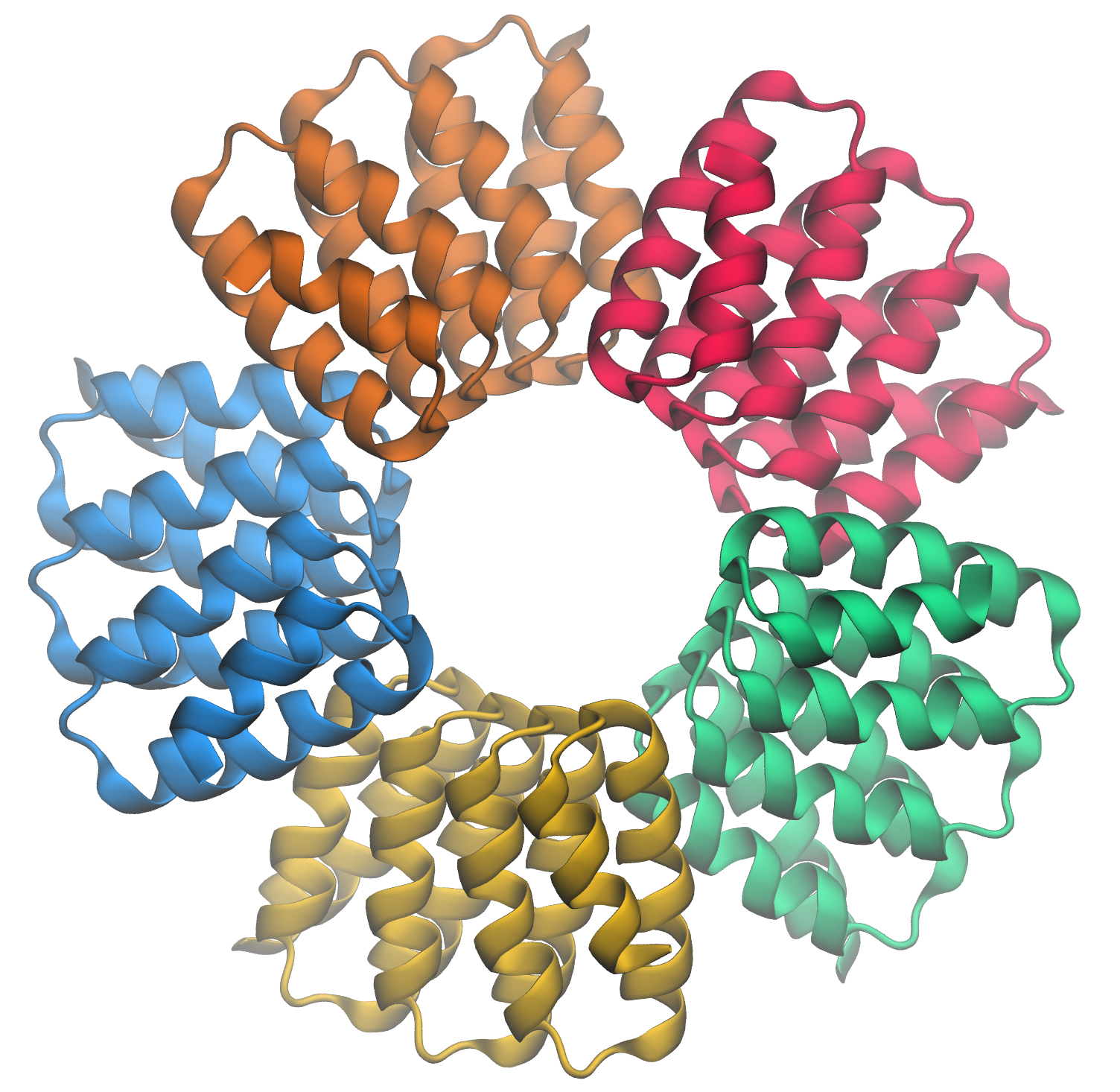

Supplement: Supplementary file 7 — Protein images, SEC source data and SAXS source data. [file 41557_2023_1314_MOESM7_ESM.zip › ExtendedDataFigure1/2o35.jpg]

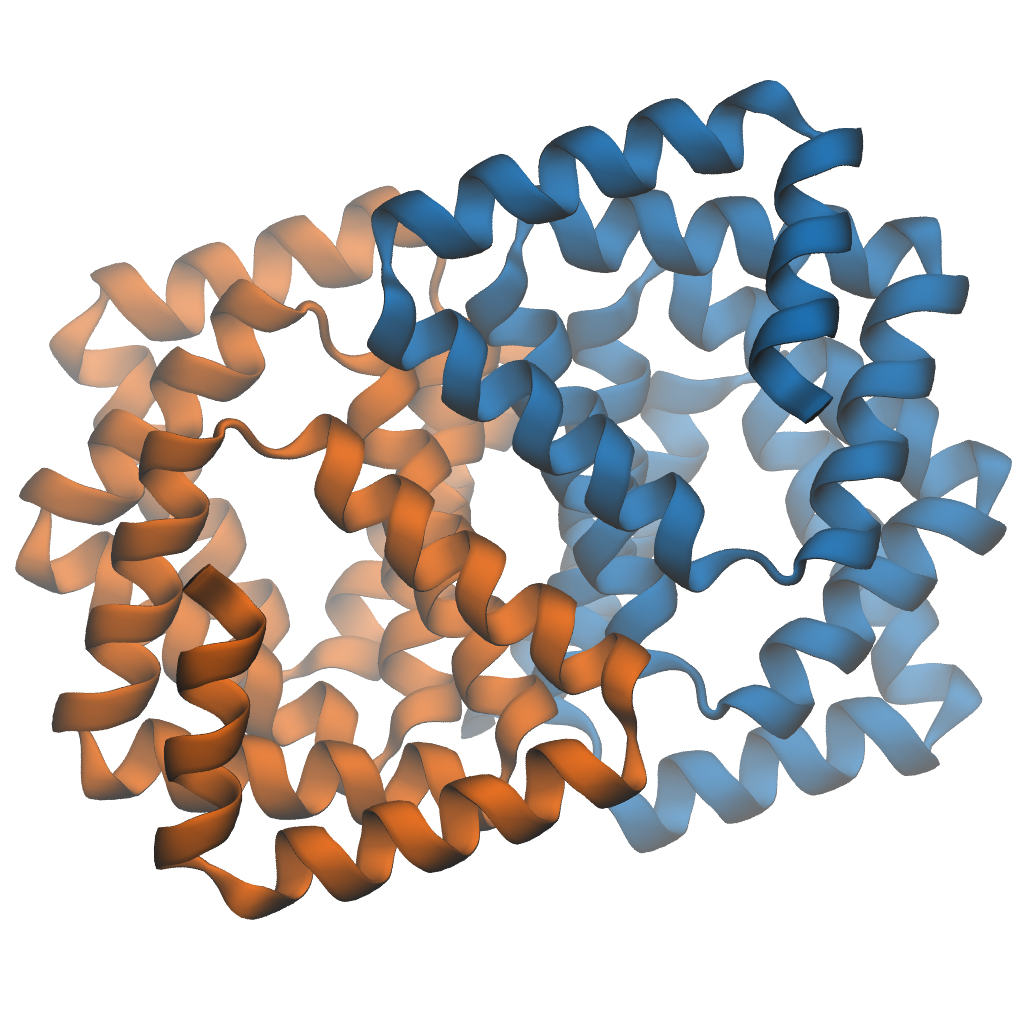

Supplement: Supplementary file 7 — Protein images, SEC source data and SAXS source data. [file 41557_2023_1314_MOESM7_ESM.zip › ExtendedDataFigure1/3d19.jpg]

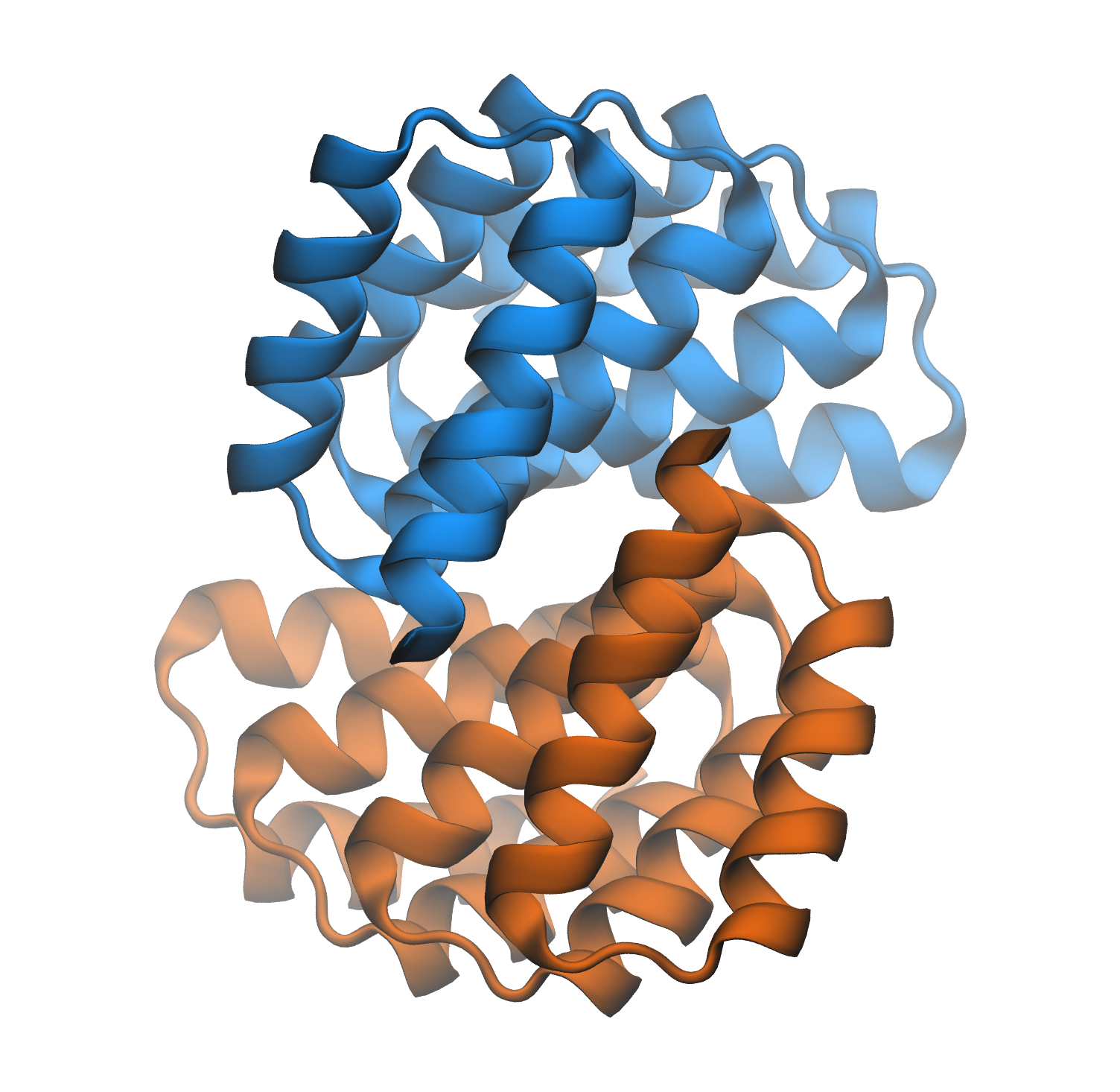

Supplement: Supplementary file 7 — Protein images, SEC source data and SAXS source data. [file 41557_2023_1314_MOESM7_ESM.zip › ExtendedDataFigure1/2d7.jpg]

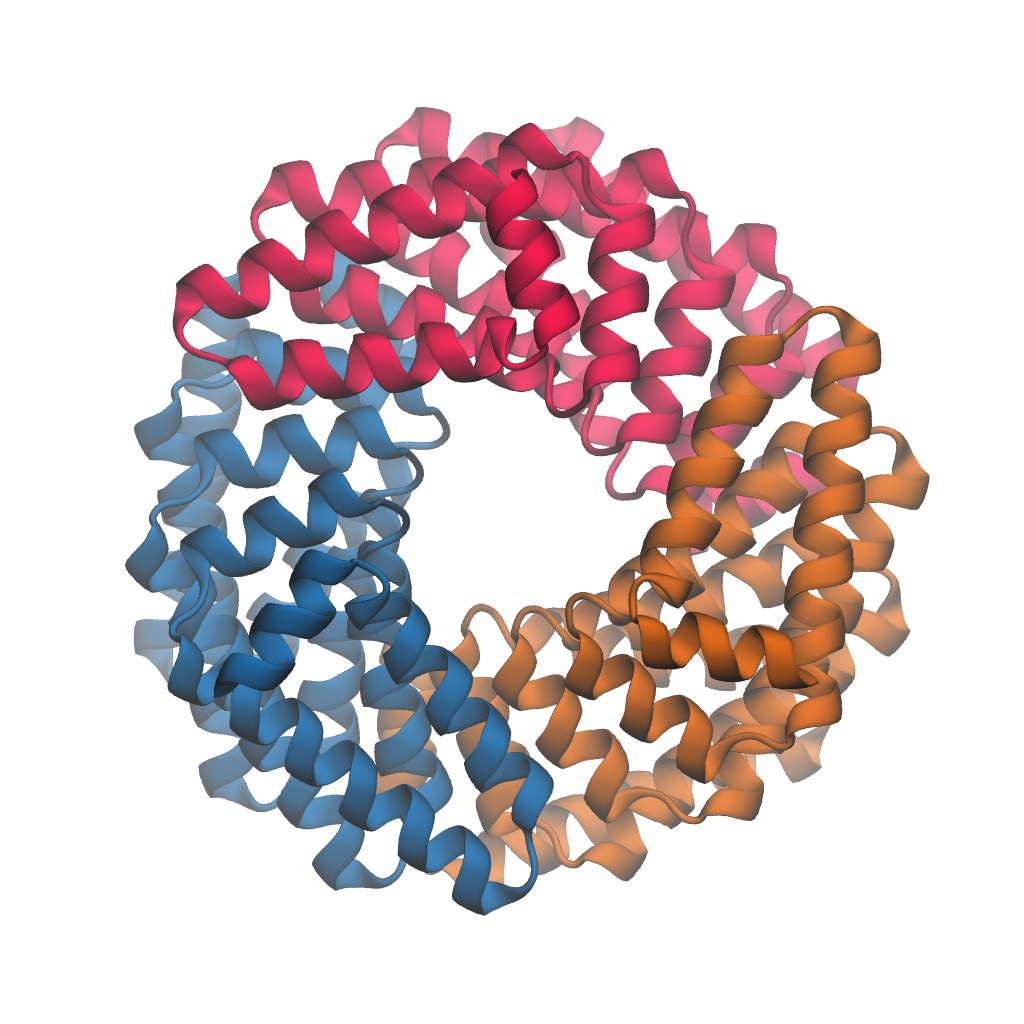

Supplement: Supplementary file 8 — Protein images, SEC source data and circular dichroism source data. [file 41557_2023_1314_MOESM8_ESM.zip › ExtendedDataFigure2/3o35.jpg]

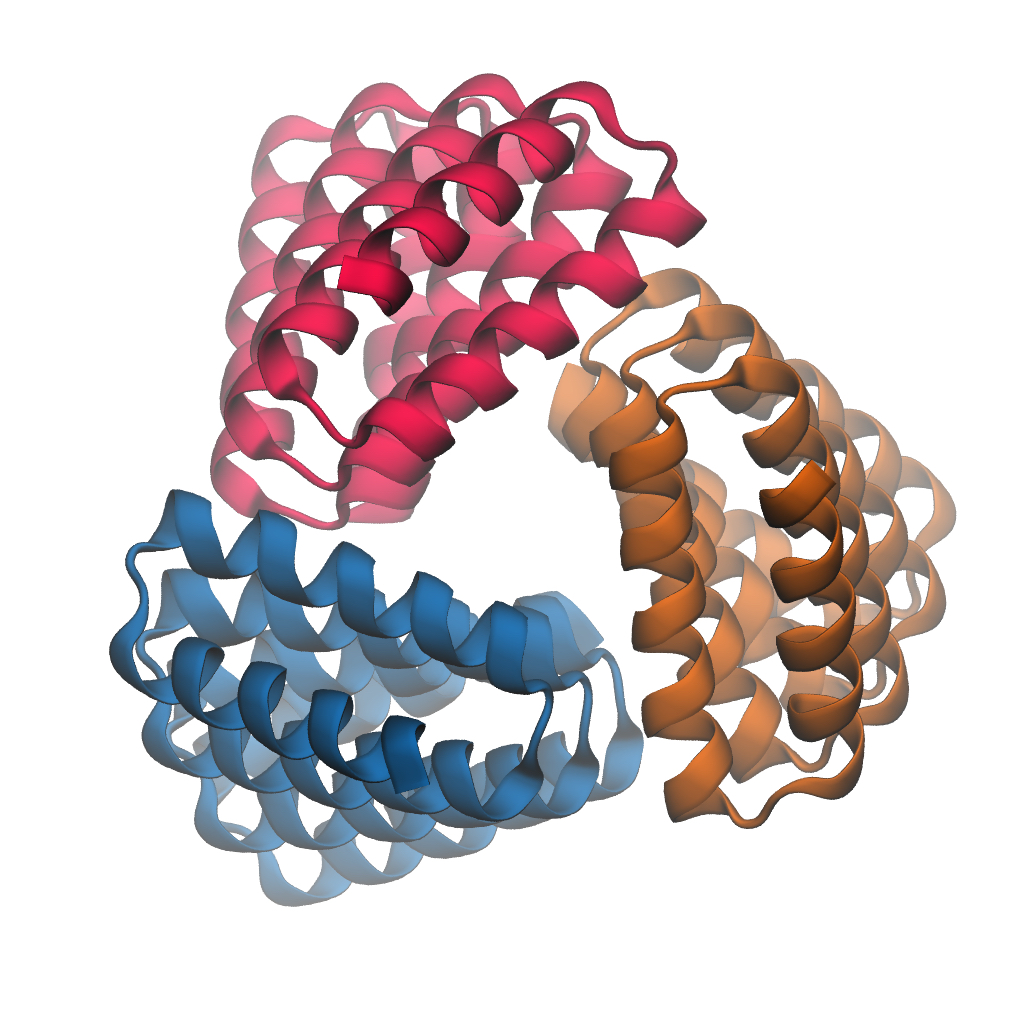

Supplement: Supplementary file 8 — Protein images, SEC source data and circular dichroism source data. [file 41557_2023_1314_MOESM8_ESM.zip › ExtendedDataFigure2/3o43.jpg]

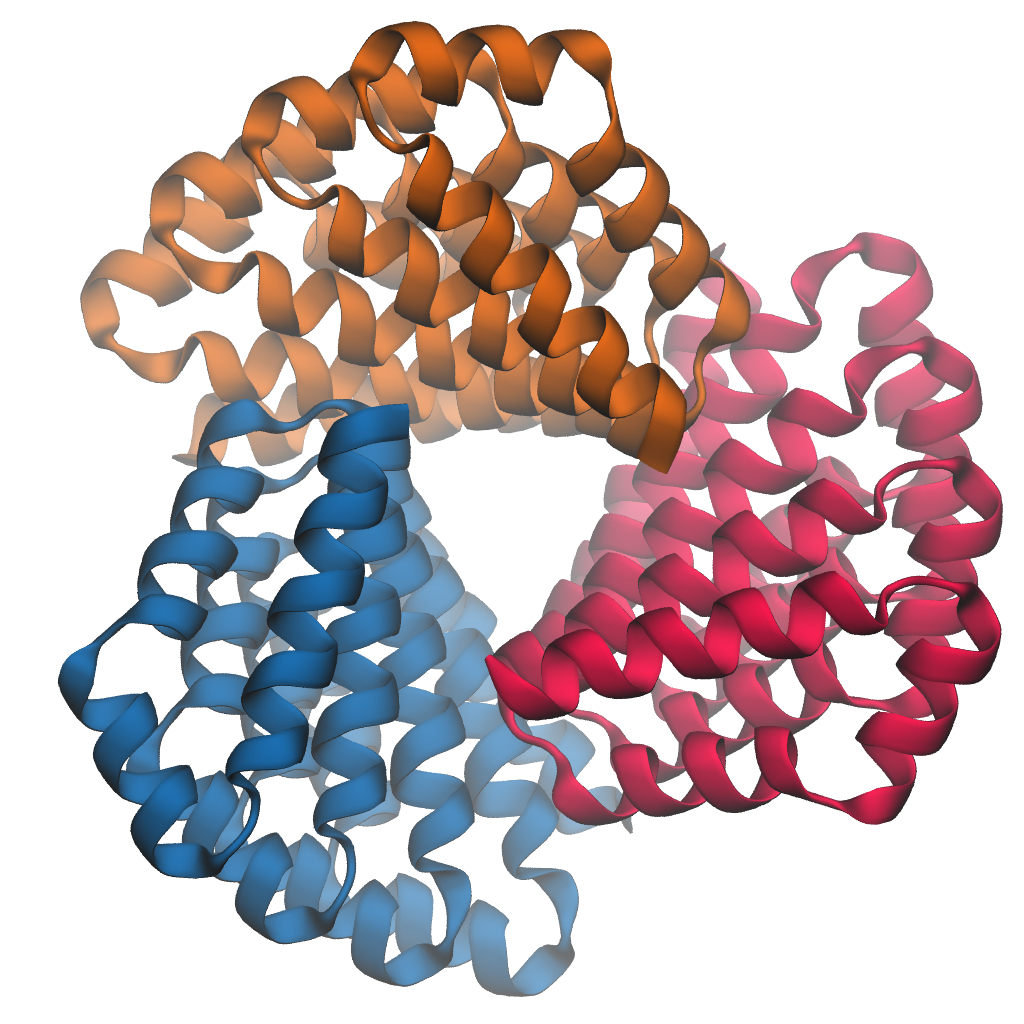

Supplement: Supplementary file 8 — Protein images, SEC source data and circular dichroism source data. [file 41557_2023_1314_MOESM8_ESM.zip › ExtendedDataFigure2/3o49.jpg]

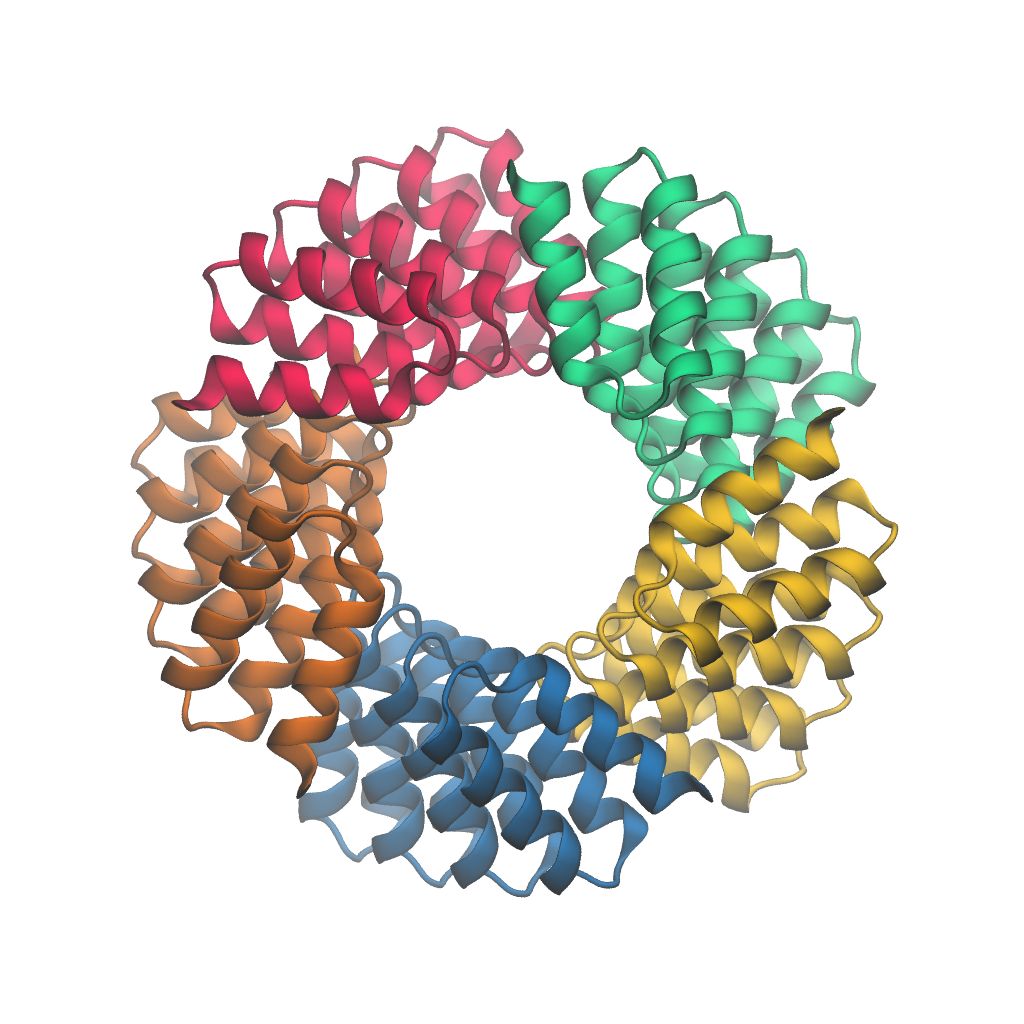

Supplement: Supplementary file 8 — Protein images, SEC source data and circular dichroism source data. [file 41557_2023_1314_MOESM8_ESM.zip › ExtendedDataFigure2/2o25.jpg]

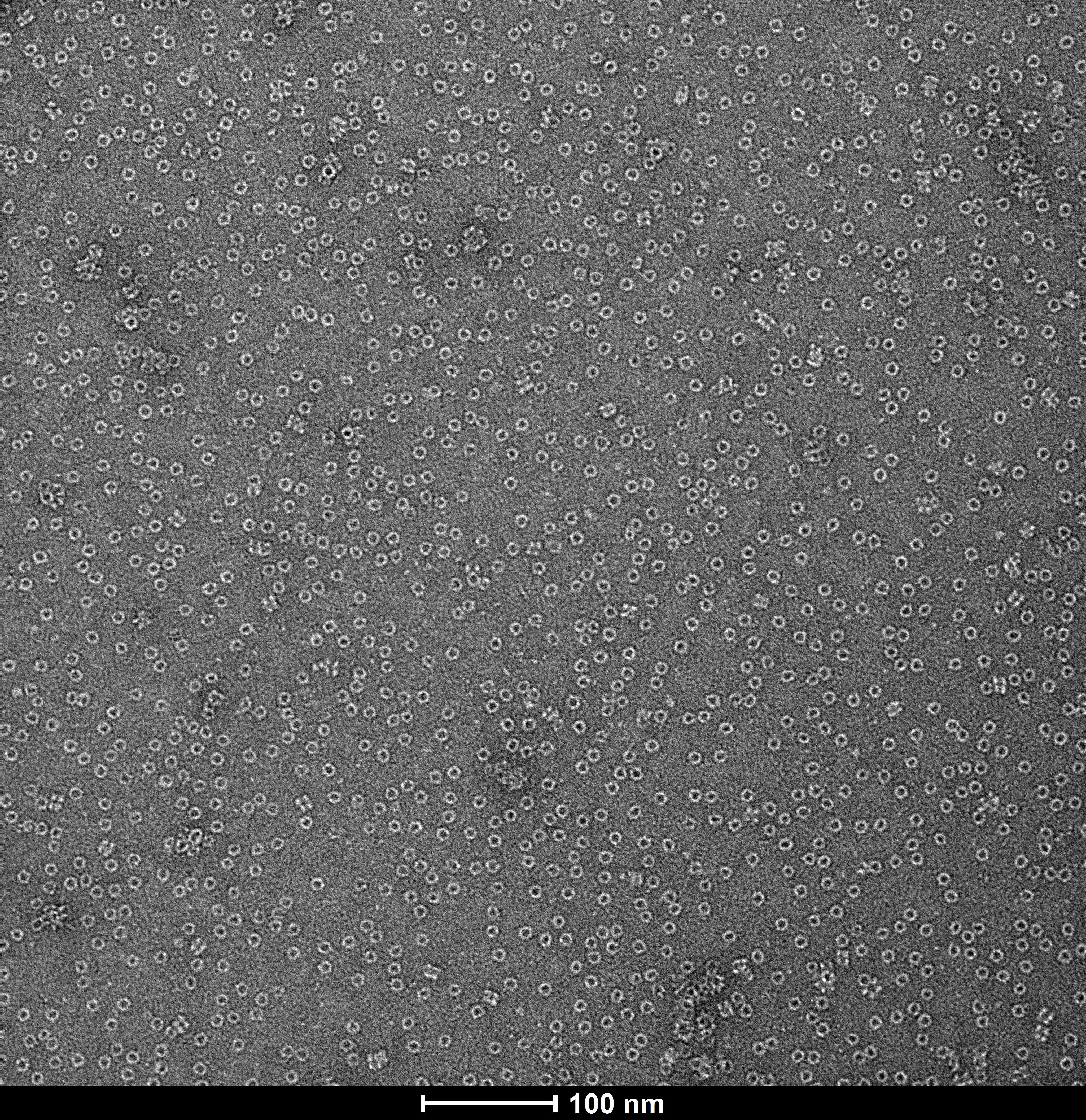

Supplement: Supplementary file 9 — The nsEM images. [file 41557_2023_1314_MOESM9_ESM.zip › ExtendedDataFigure3/WB5_7T_2o63_0p01mgml_73k.jpg]

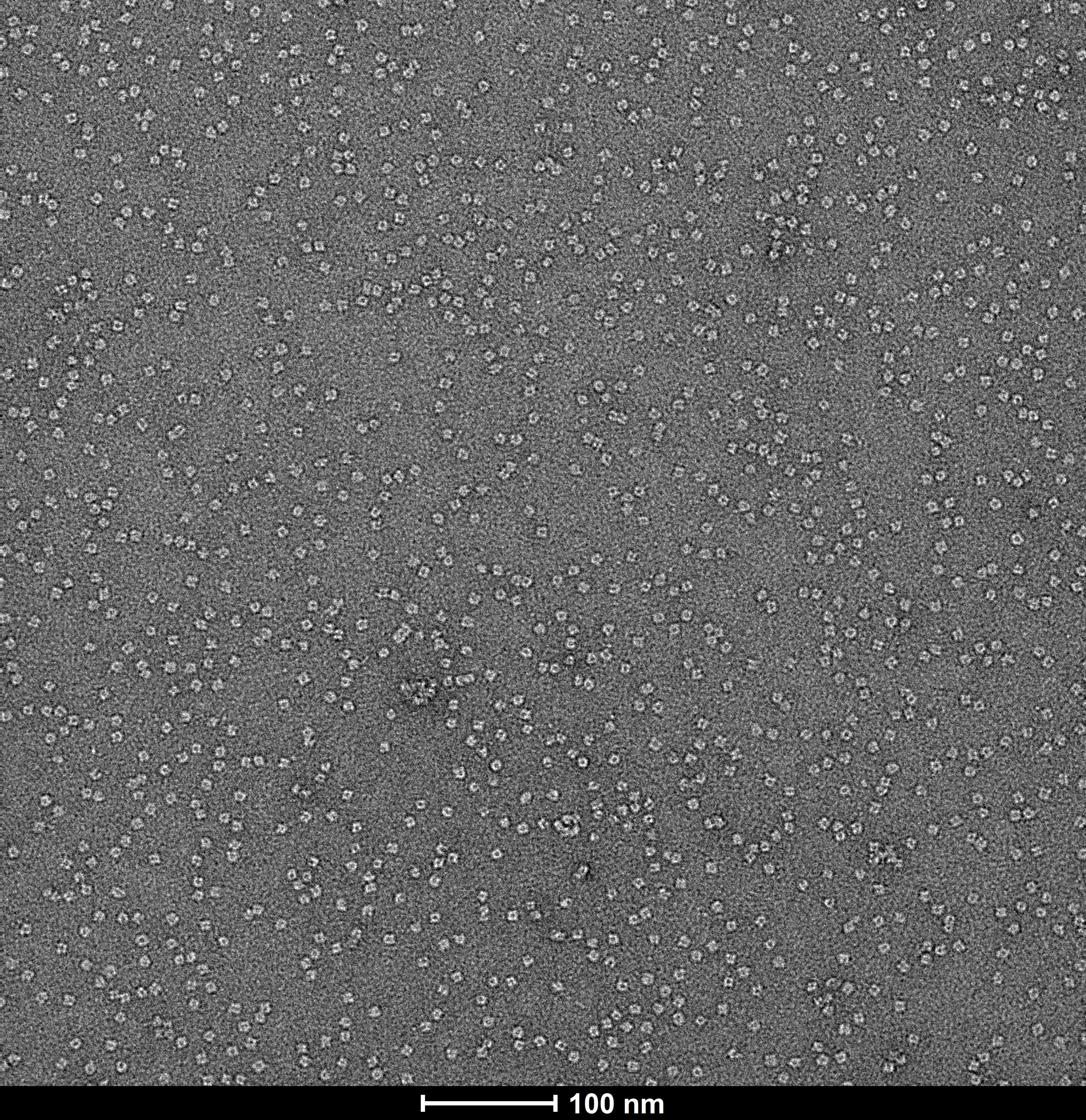

Supplement: Supplementary file 9 — The nsEM images. [file 41557_2023_1314_MOESM9_ESM.zip › ExtendedDataFigure3/WB4_10F_3o22_1_200_73k.jpg]

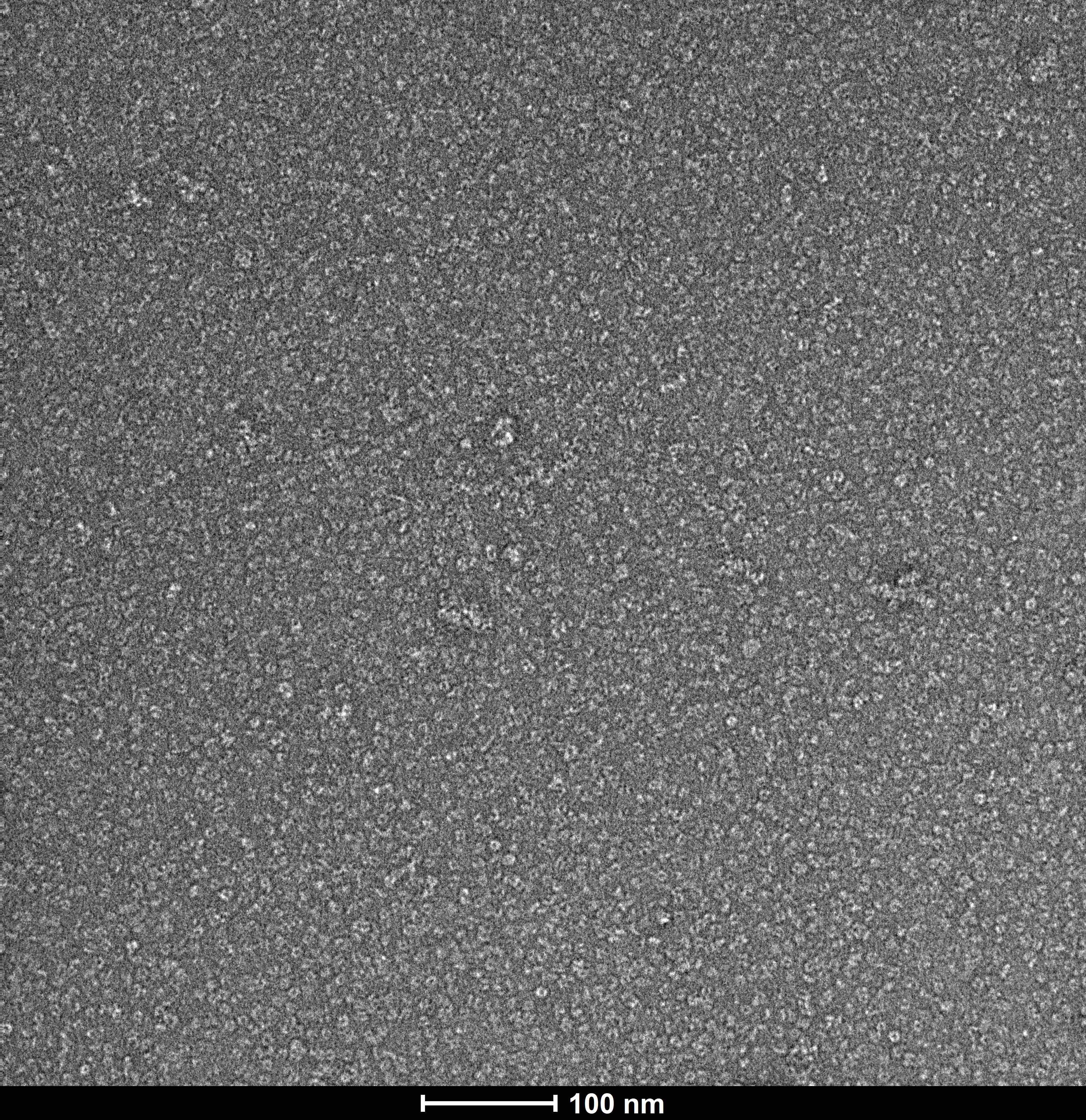

Supplement: Supplementary file 9 — The nsEM images. [file 41557_2023_1314_MOESM9_ESM.zip › ExtendedDataFigure3/WB4_8Q_2o35_0p08_mg_ml_36k.jpg]

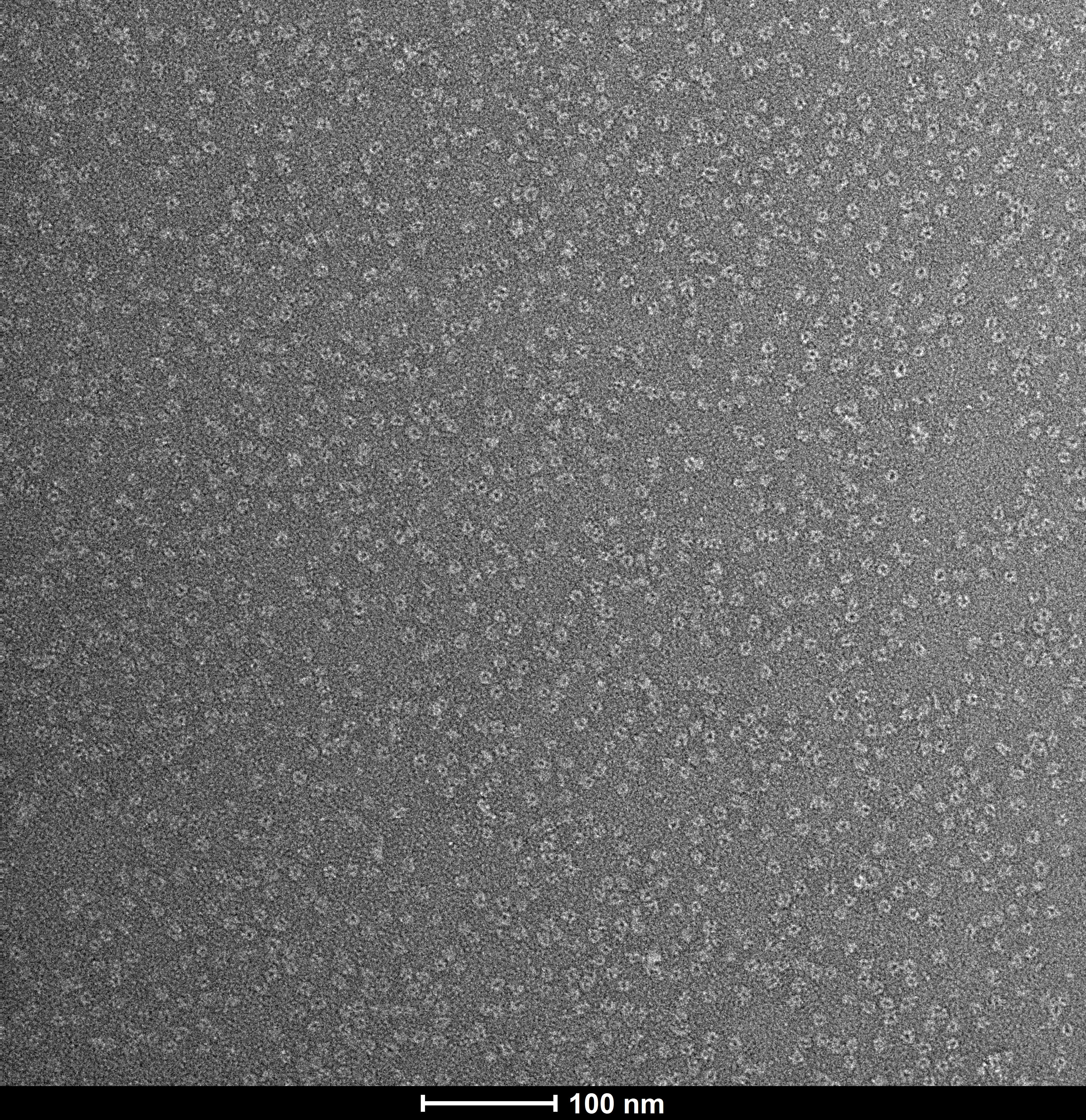

Supplement: Supplementary file 9 — The nsEM images. [file 41557_2023_1314_MOESM9_ESM.zip › ExtendedDataFigure3/WB4_10O_2o43_1_20_73k.jpg]

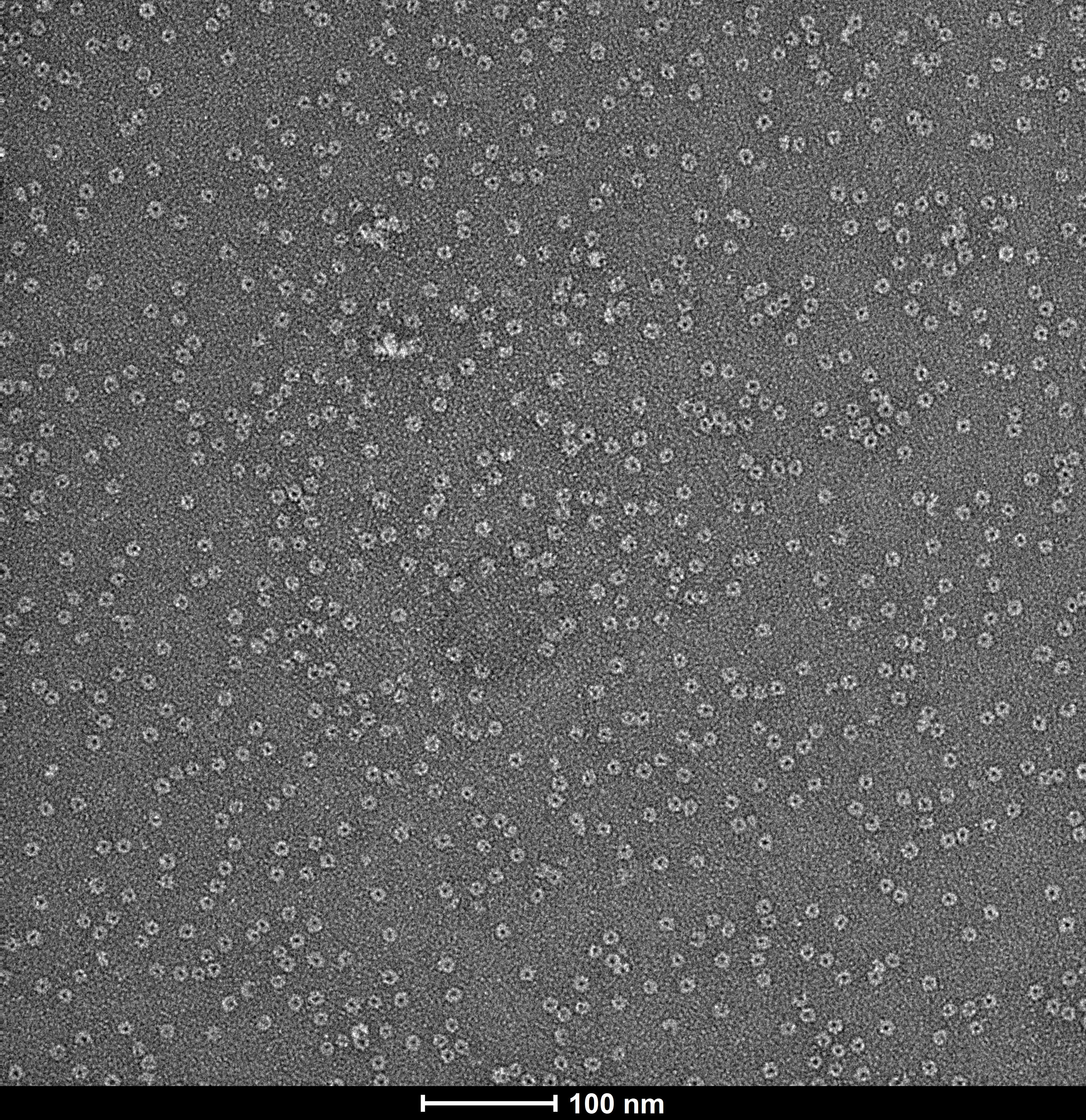

Supplement: Supplementary file 9 — The nsEM images. [file 41557_2023_1314_MOESM9_ESM.zip › ExtendedDataFigure3/WB4_10Q_2o44_1_20_73k.jpg]

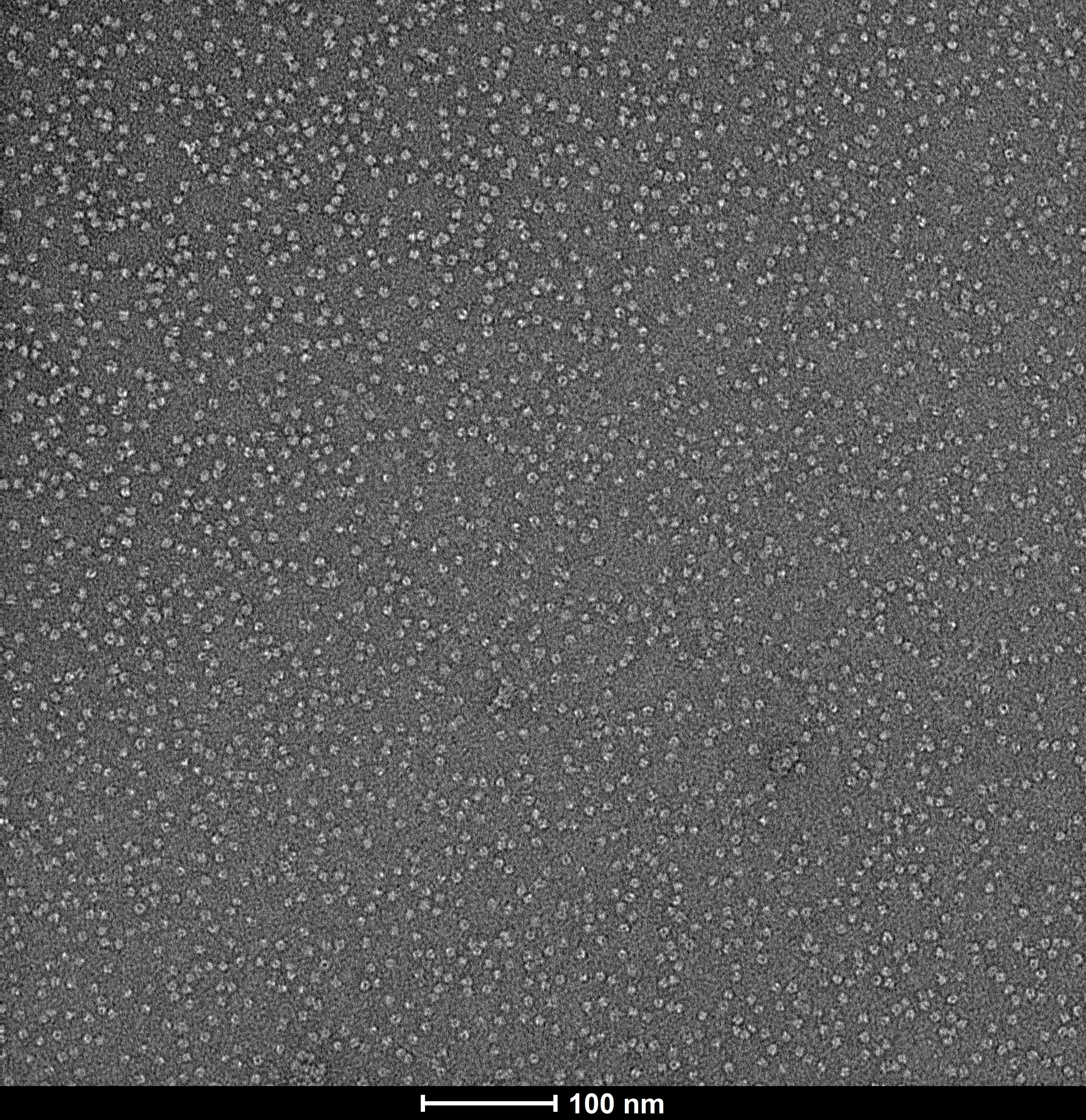

Supplement: Supplementary file 9 — The nsEM images. [file 41557_2023_1314_MOESM9_ESM.zip › ExtendedDataFigure3/WB4_10B_2o31_1_200_73k.jpg]

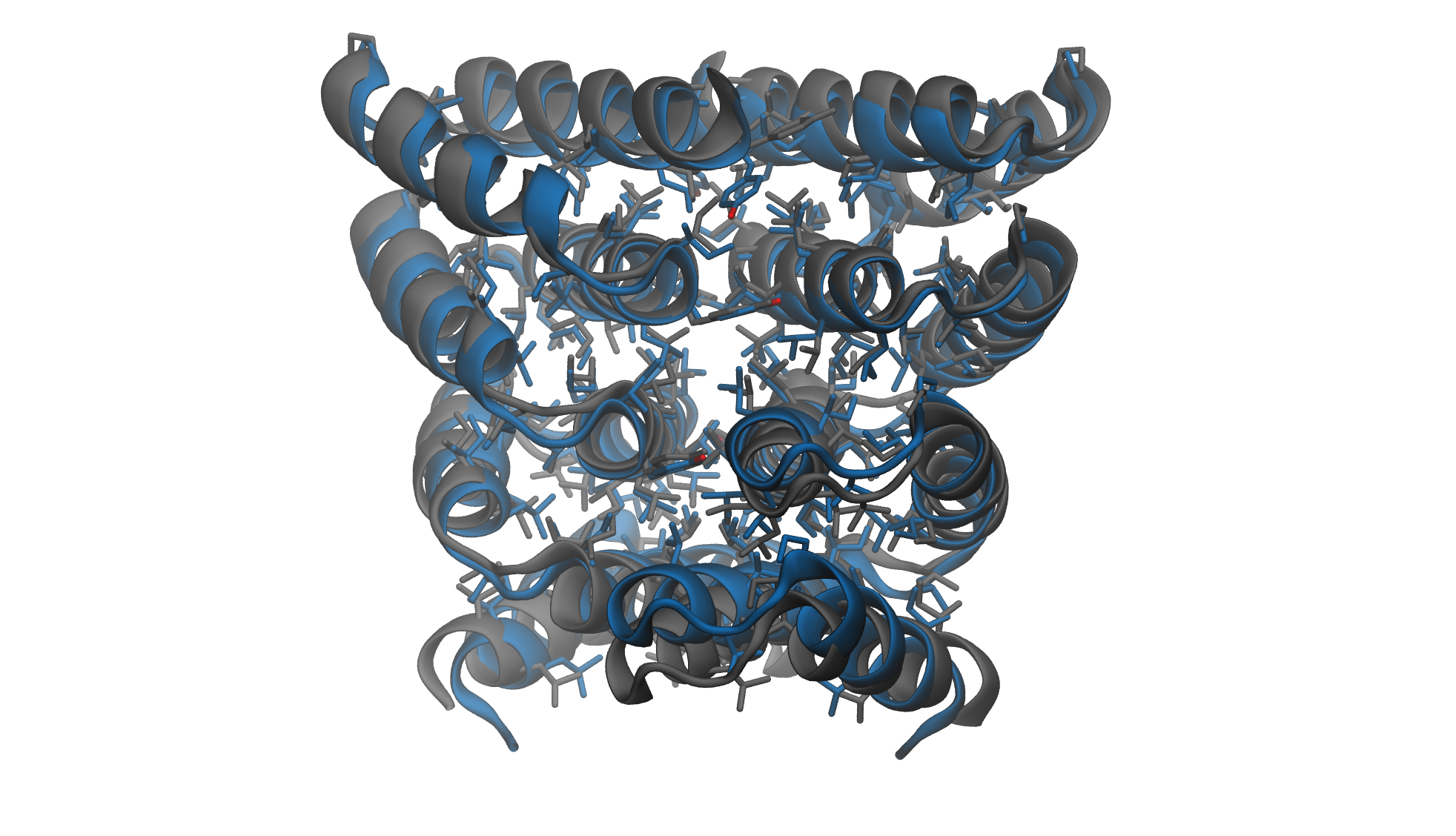

Supplement: Supplementary file 10 — Protein images. [file 41557_2023_1314_MOESM10_ESM.zip › ExtendedDataFigure4/2d7_supp.jpg]

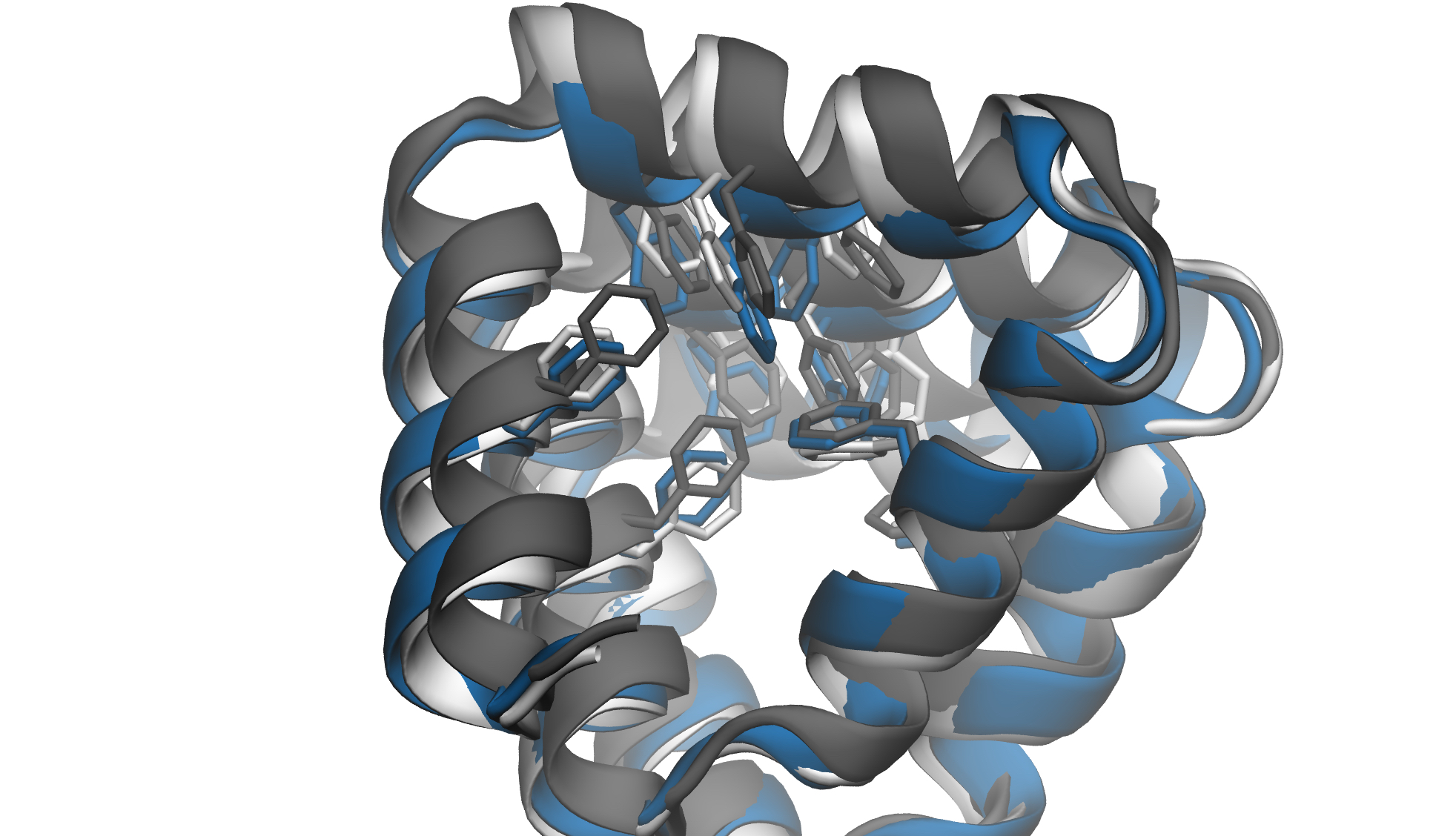

Supplement: Supplementary file 10 — Protein images. [file 41557_2023_1314_MOESM10_ESM.zip › ExtendedDataFigure4/3o22_supp.jpg]

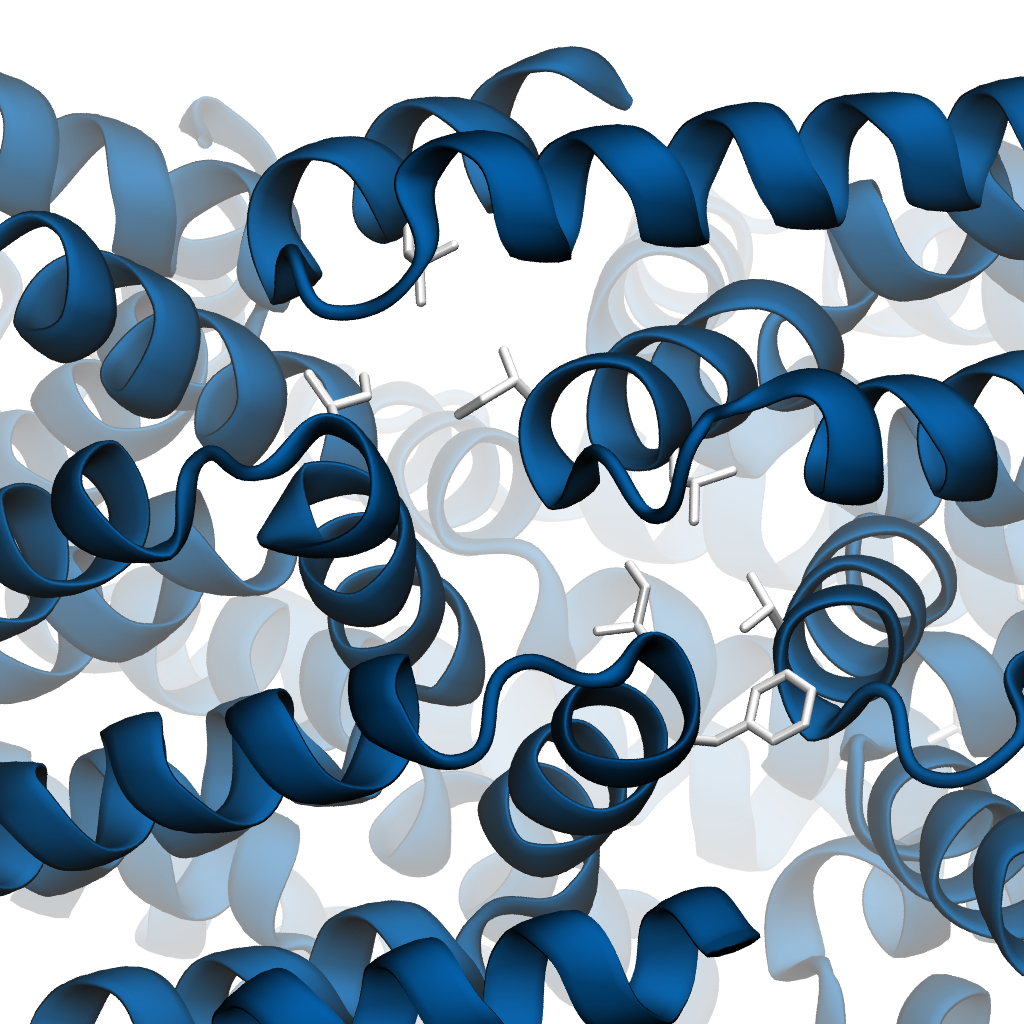

Supplement: Supplementary file 10 — Protein images. [file 41557_2023_1314_MOESM10_ESM.zip › ExtendedDataFigure4/3o52_SC.jpg]

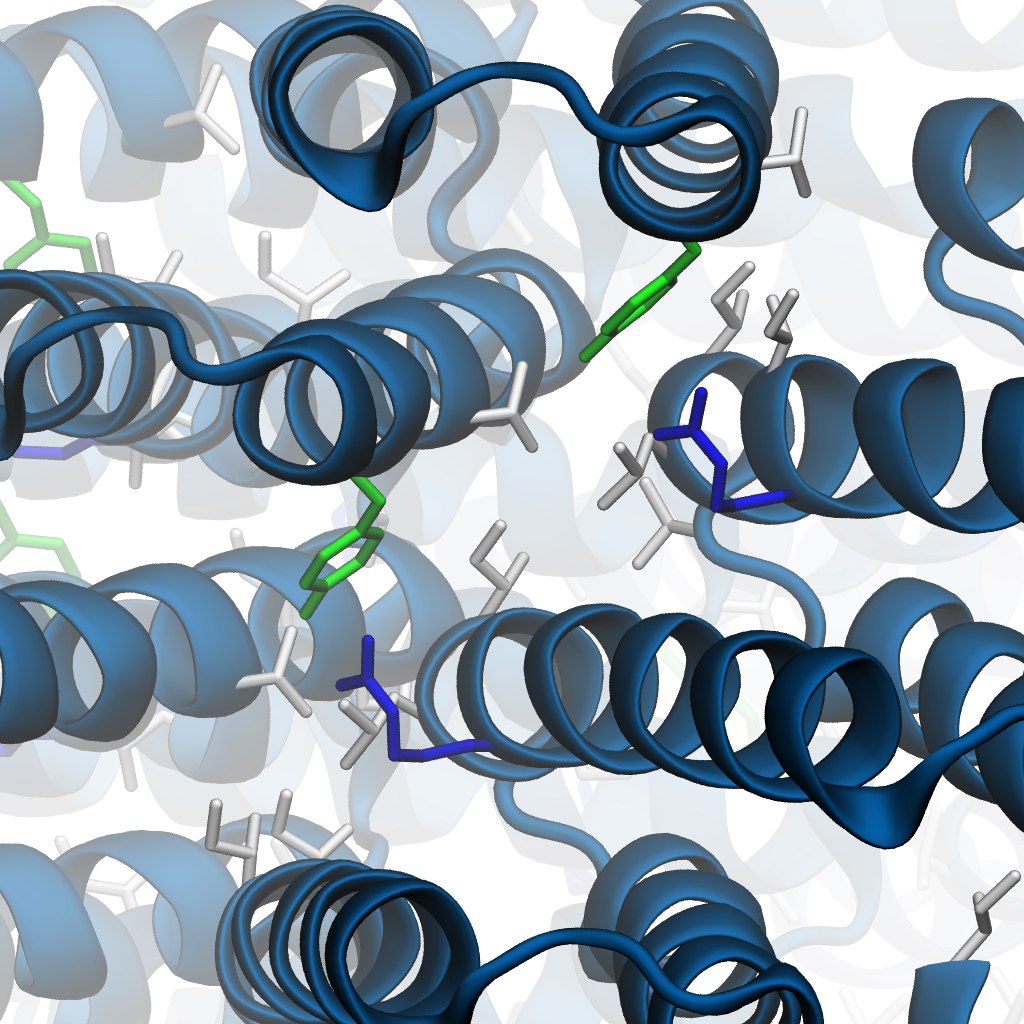

Supplement: Supplementary file 10 — Protein images. [file 41557_2023_1314_MOESM10_ESM.zip › ExtendedDataFigure4/2o44_SC.jpg]

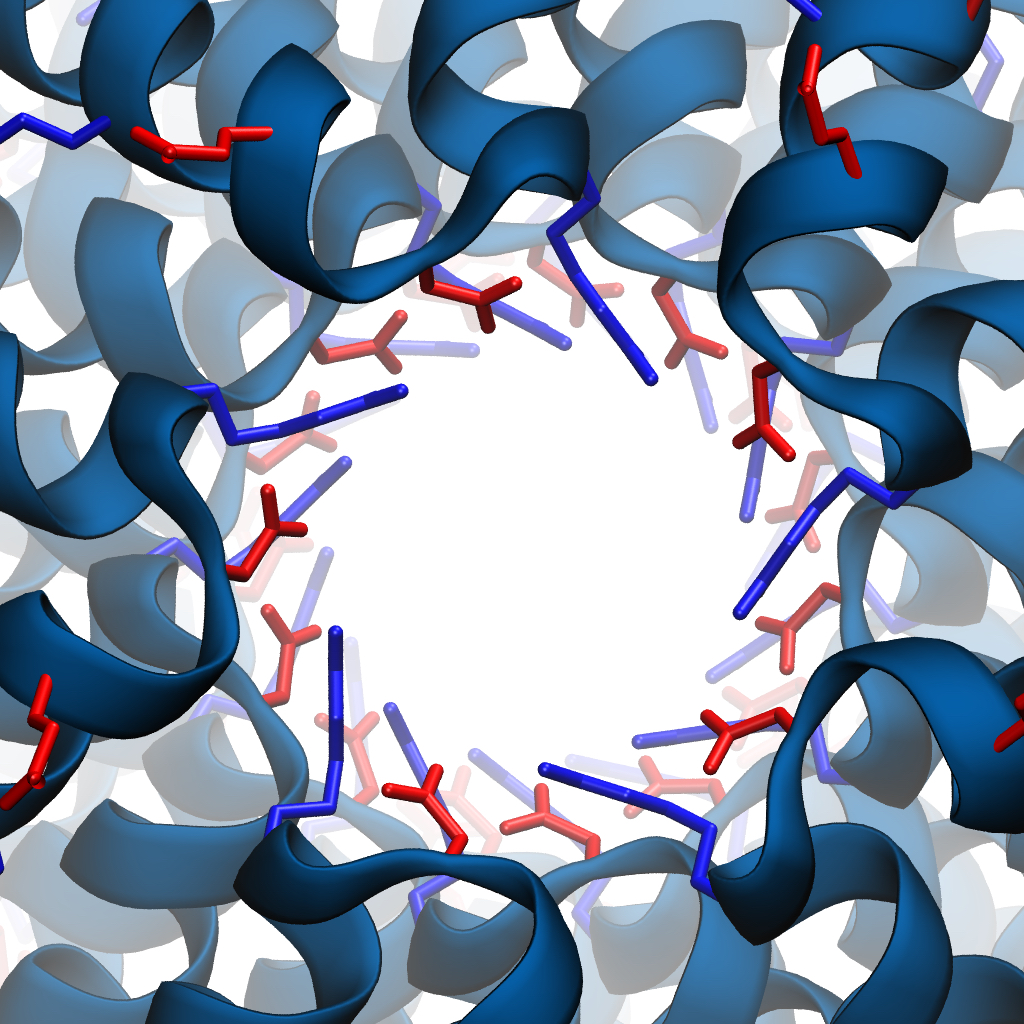

Supplement: Supplementary file 10 — Protein images. [file 41557_2023_1314_MOESM10_ESM.zip › ExtendedDataFigure4/2o31_SC.jpg]

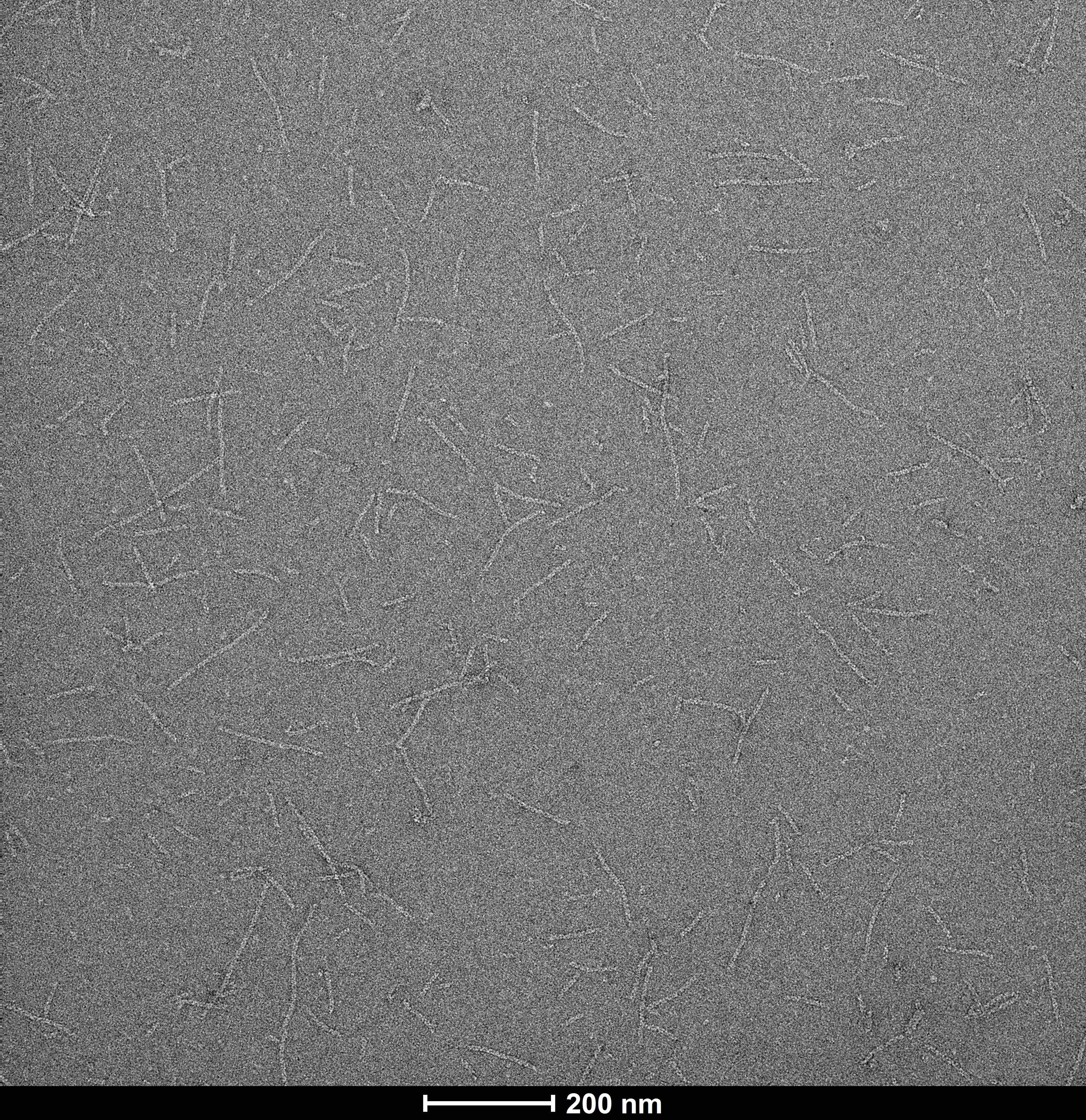

Supplement: Supplementary file 11 — The nsEM images. [file 41557_2023_1314_MOESM11_ESM.zip › ExtendedDataFigure5/WB14_1C_L44_4C_T7d_1_25_36k.jpg]

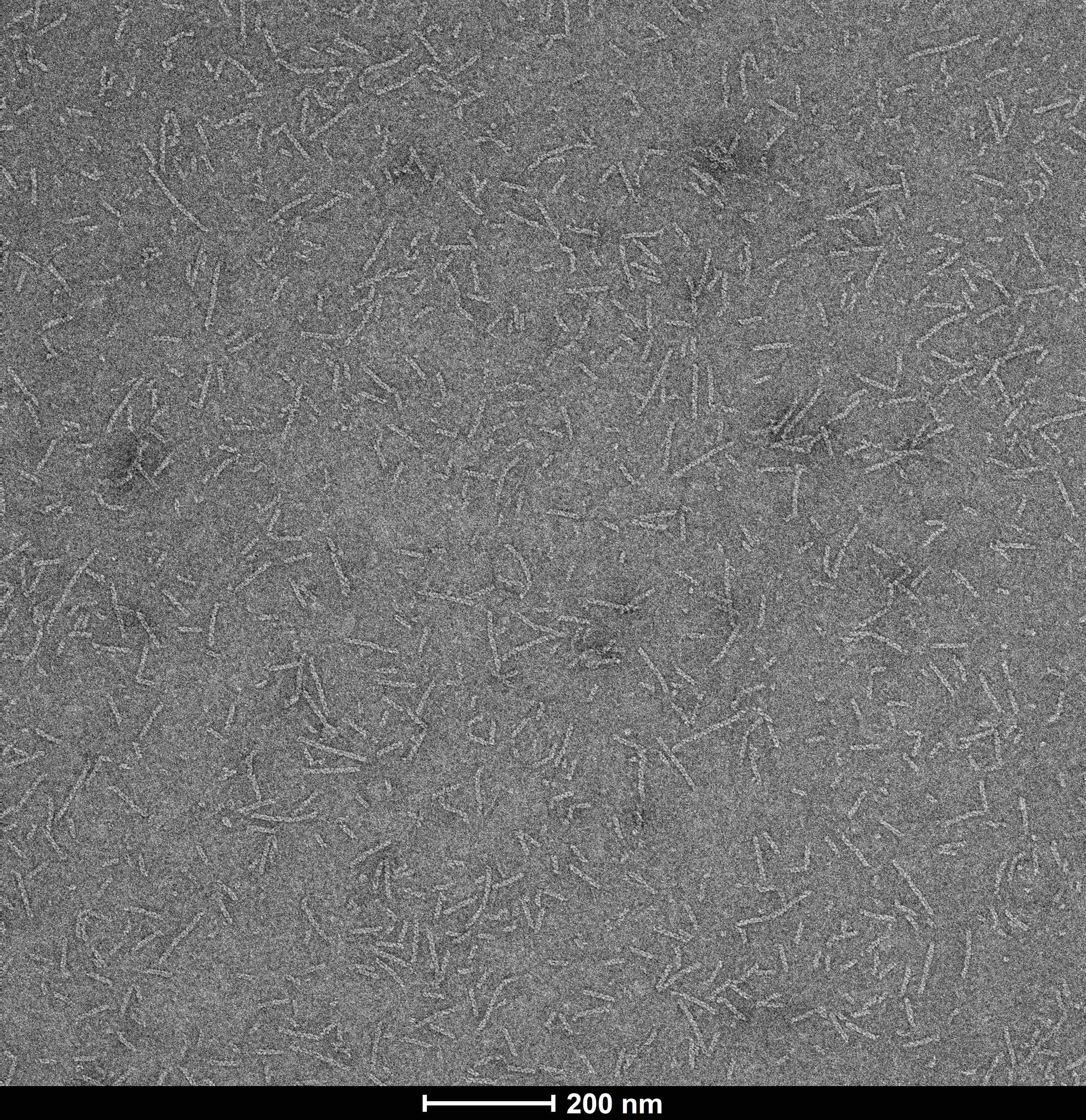

Supplement: Supplementary file 11 — The nsEM images. [file 41557_2023_1314_MOESM11_ESM.zip › ExtendedDataFigure5/WB13_5R_L44_37C_T0_1_25_36k.jpg]

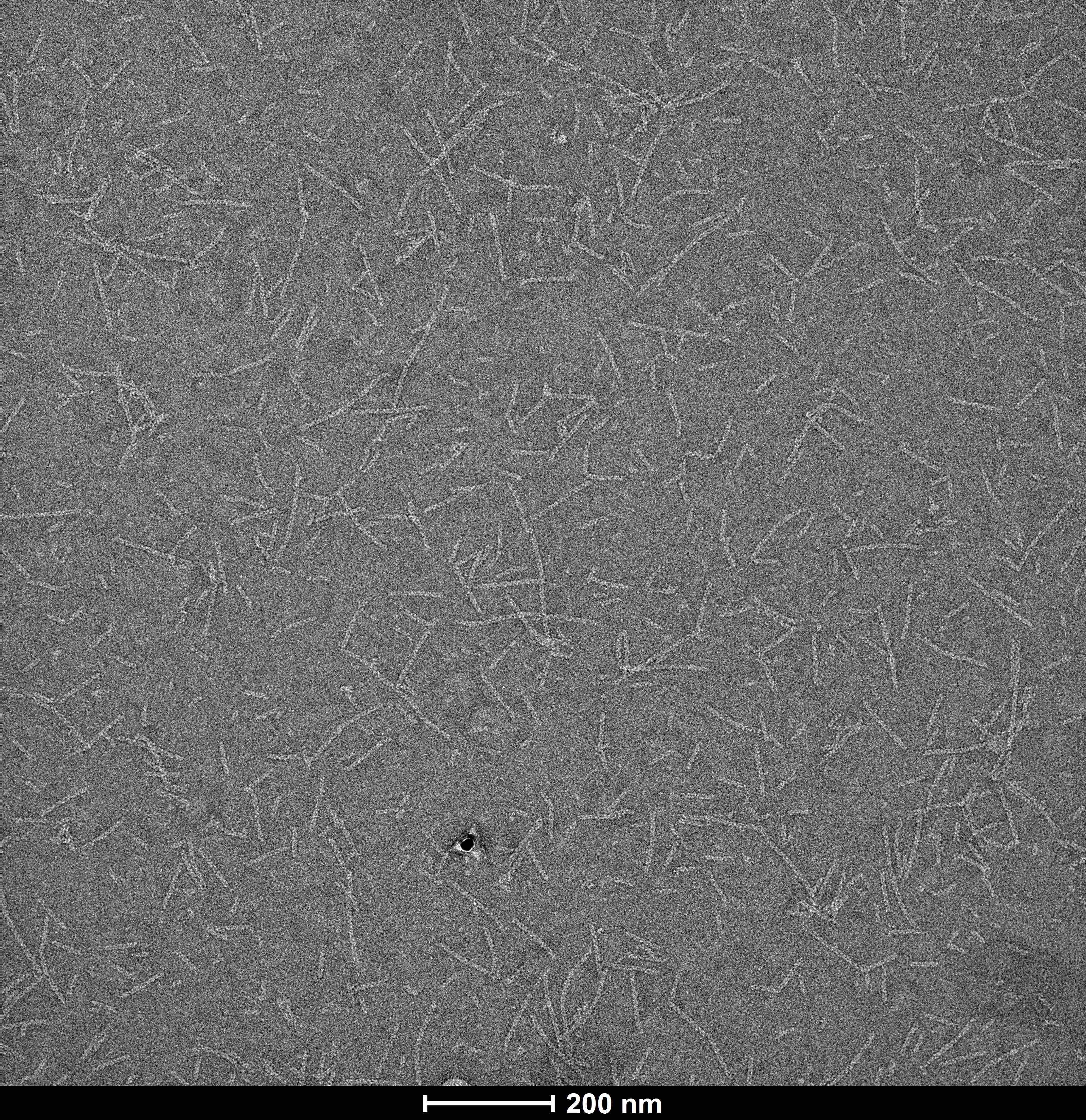

Supplement: Supplementary file 11 — The nsEM images. [file 41557_2023_1314_MOESM11_ESM.zip › ExtendedDataFigure5/WB13_9R_L44_37C_T24_1_25_36k.jpg]

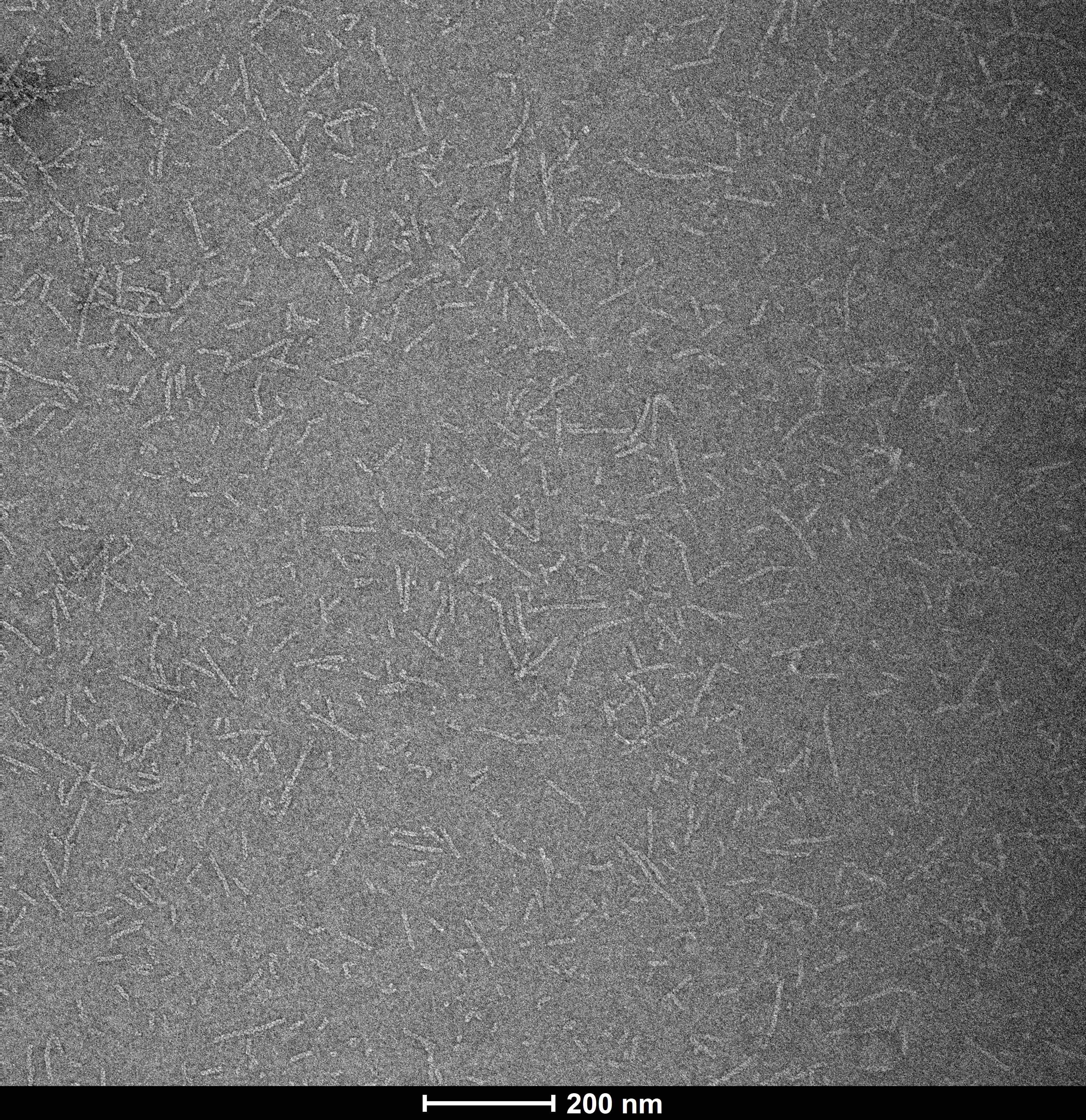

Supplement: Supplementary file 11 — The nsEM images. [file 41557_2023_1314_MOESM11_ESM.zip › ExtendedDataFigure5/WB13_5R_L44_22C_T0_1_25_36k.jpg]

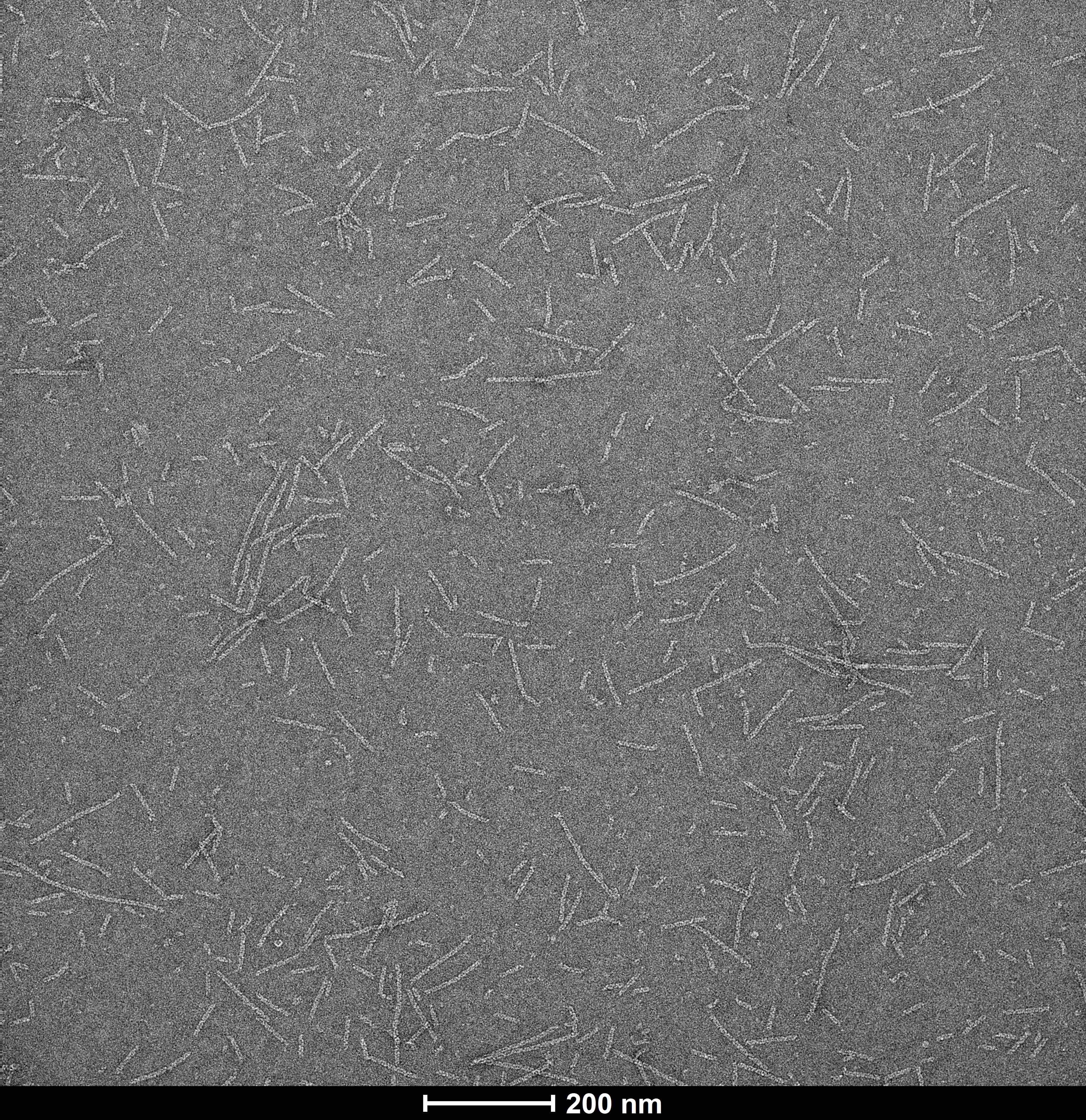

Supplement: Supplementary file 11 — The nsEM images. [file 41557_2023_1314_MOESM11_ESM.zip › ExtendedDataFigure5/WB13_8S_L44_4C_T24_1_25_36k.jpg]

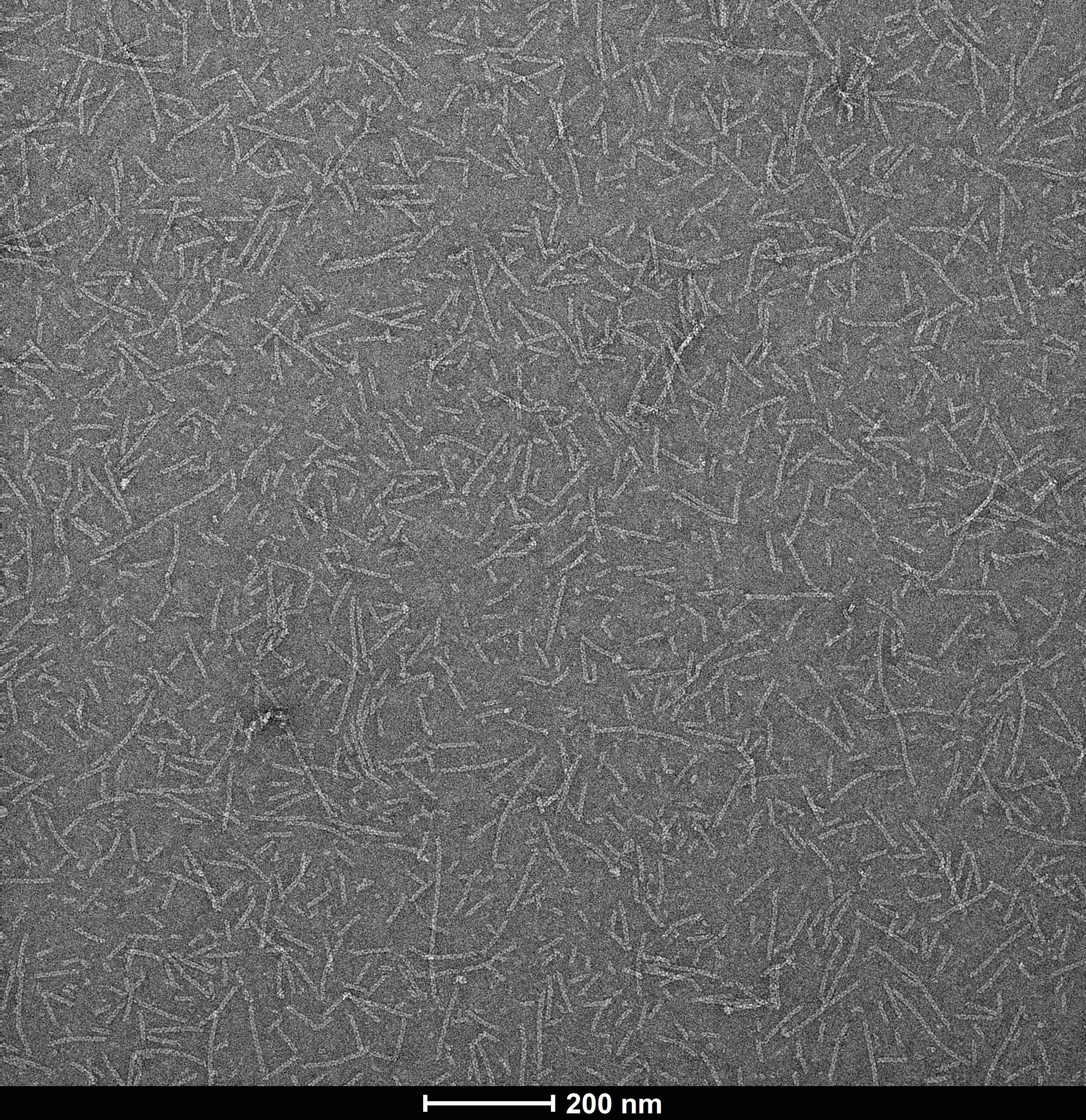

Supplement: Supplementary file 11 — The nsEM images. [file 41557_2023_1314_MOESM11_ESM.zip › ExtendedDataFigure5/WB13_9N_L44_22C_T24_1_25_36k.jpg]

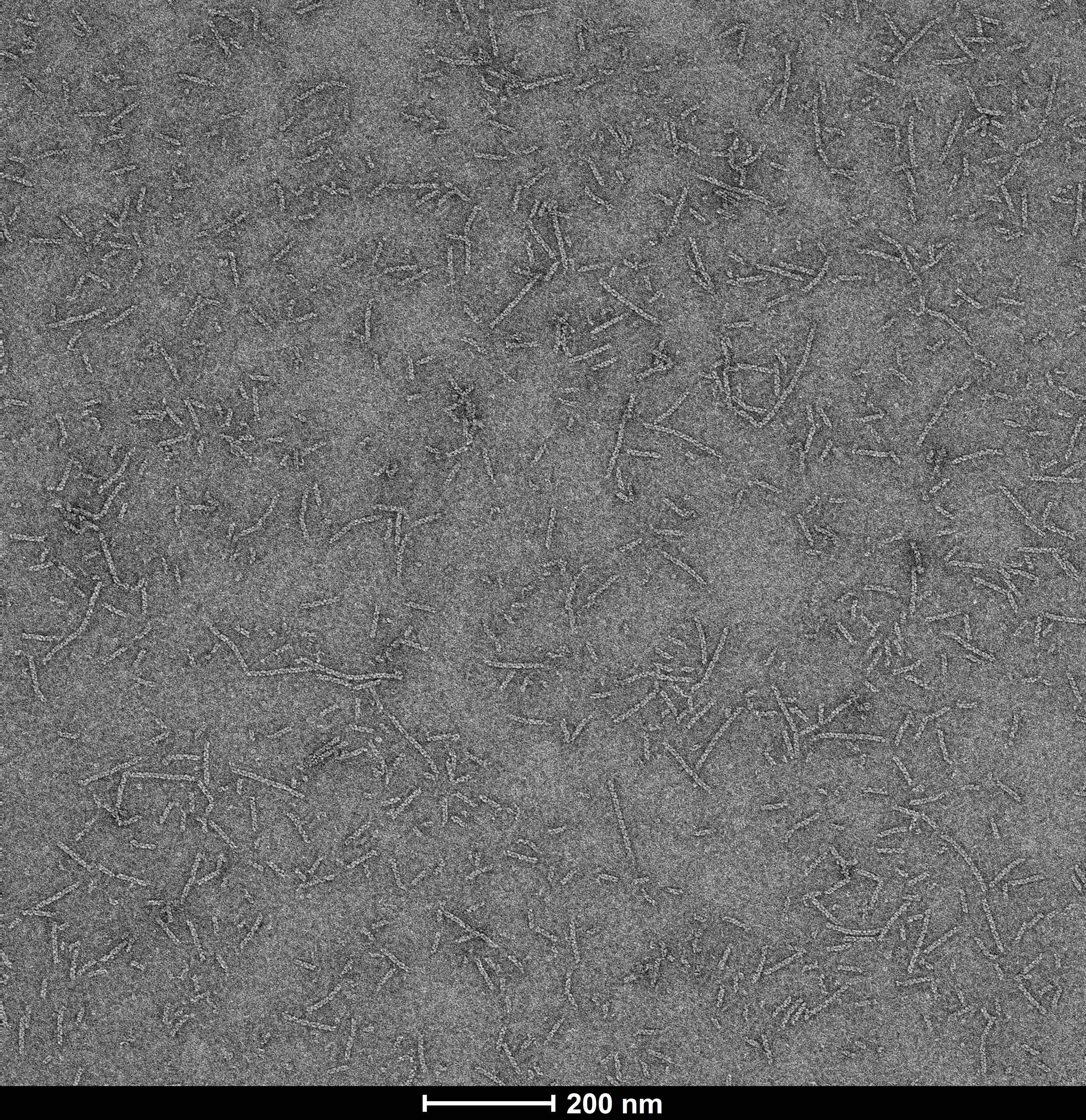

Supplement: Supplementary file 11 — The nsEM images. [file 41557_2023_1314_MOESM11_ESM.zip › ExtendedDataFigure5/WB13_5R_L44_4C_T0_1_25_36k.jpg]

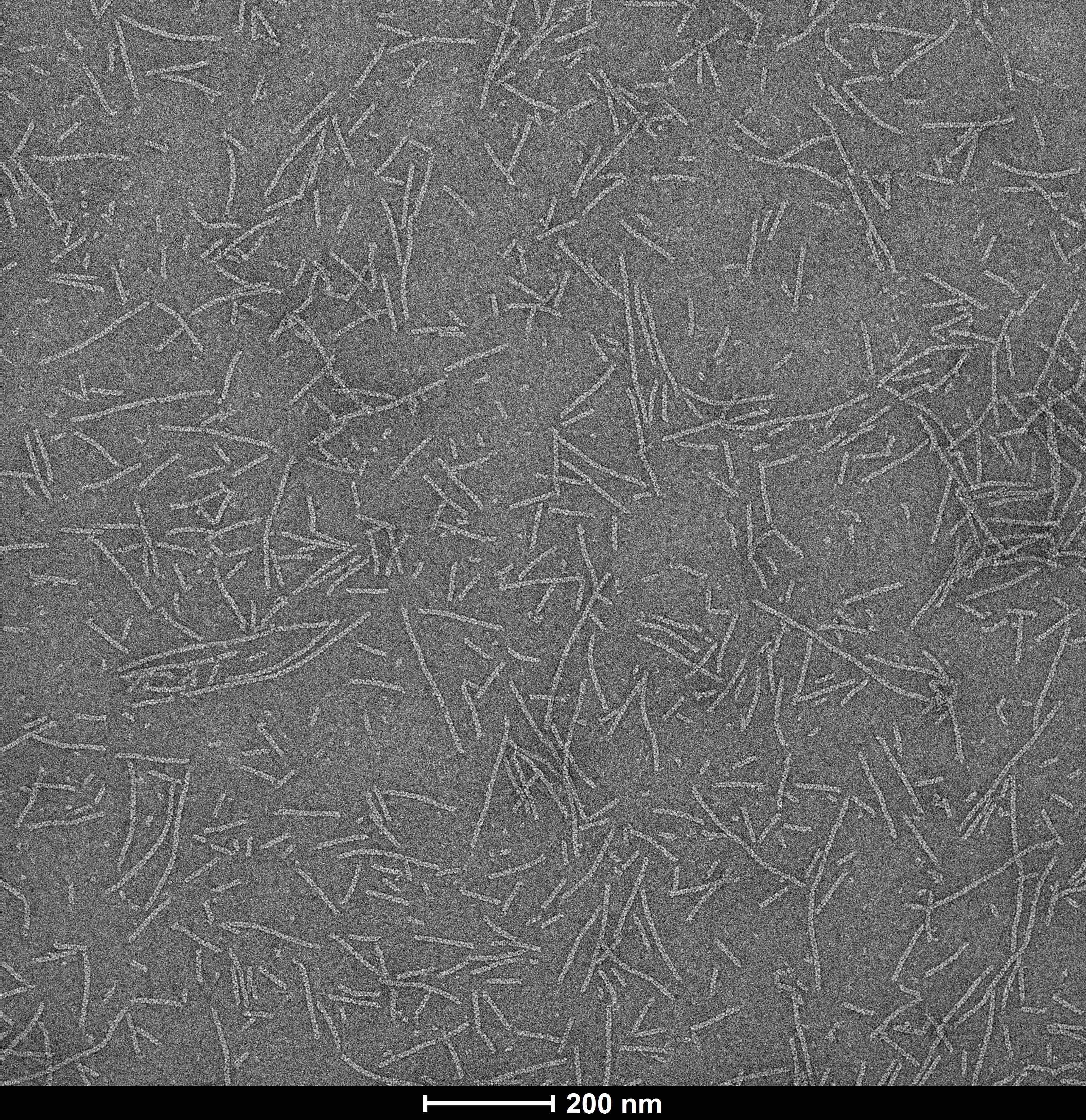

Supplement: Supplementary file 11 — The nsEM images. [file 41557_2023_1314_MOESM11_ESM.zip › ExtendedDataFigure5/WB14_1G_L44_22C_T7d_1_25_36k.jpg]

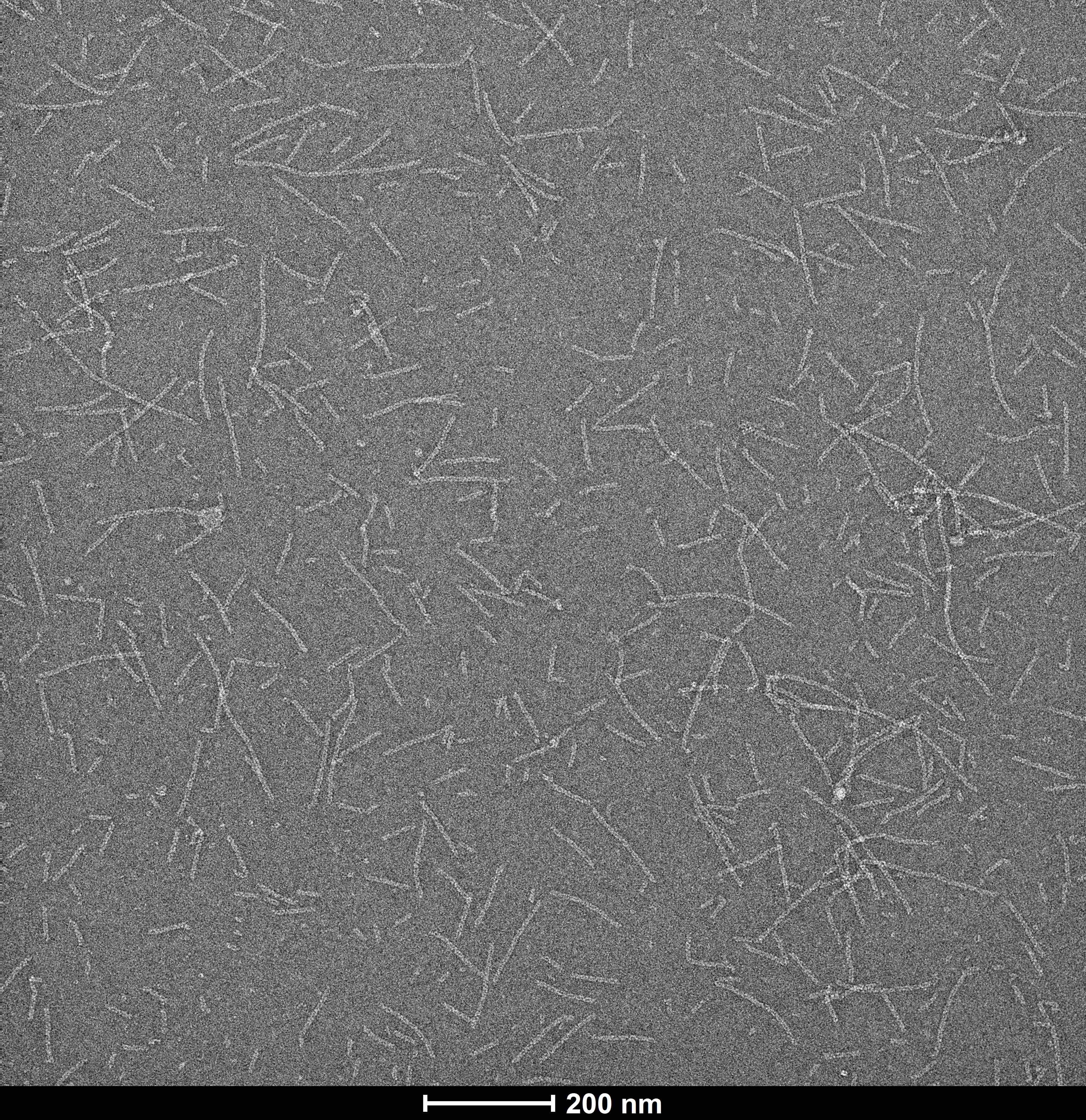

Supplement: Supplementary file 11 — The nsEM images. [file 41557_2023_1314_MOESM11_ESM.zip › ExtendedDataFigure5/WB14_2B_L44_37C_T7d_1_25_36k.jpg]

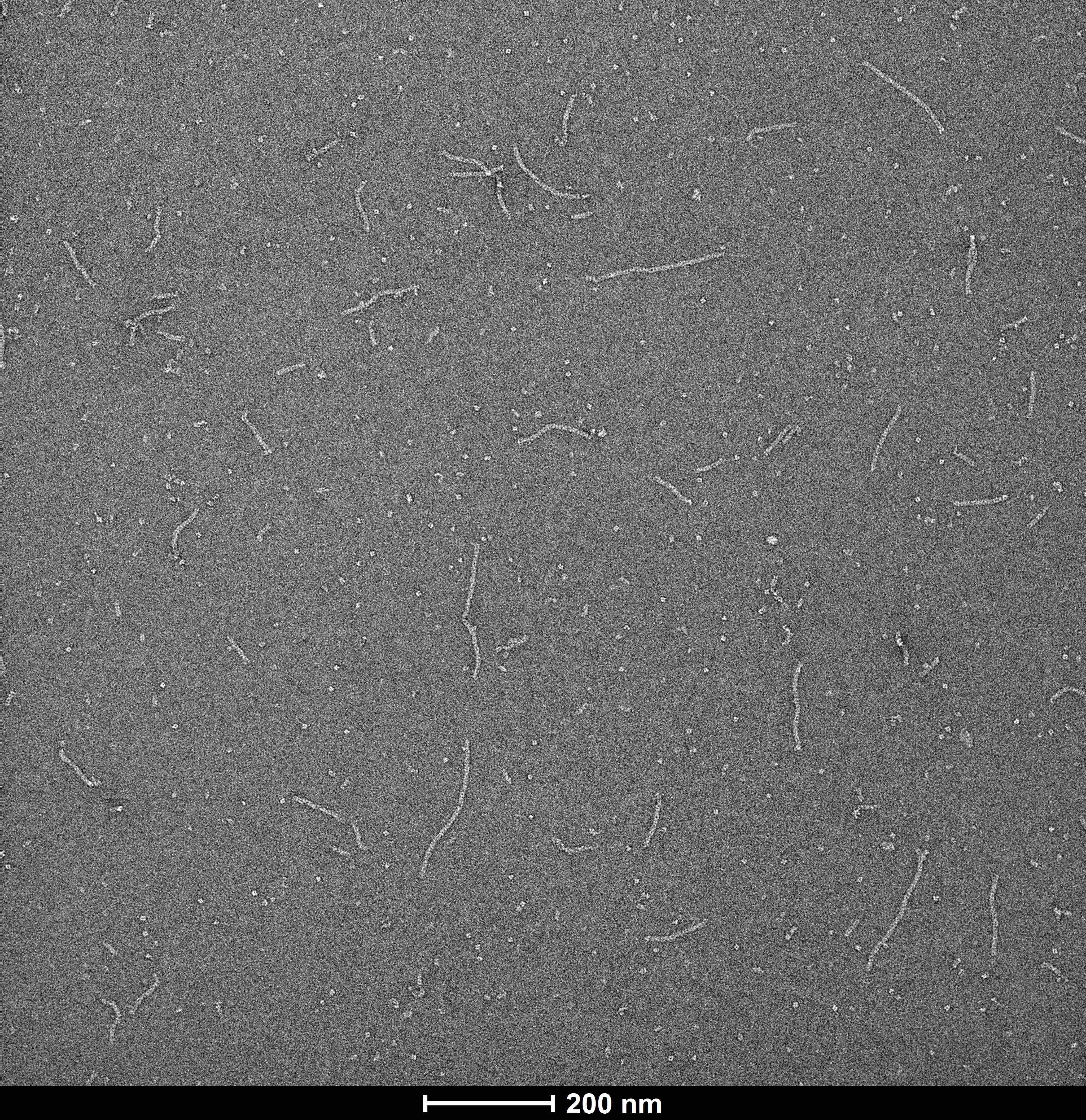

Supplement: Supplementary file 12 — The nsEM images. [file 41557_2023_1314_MOESM12_ESM.zip › ExtendedDataFigure6/WB14_1E_L41_22C_T7d_1_25_36k.jpg]

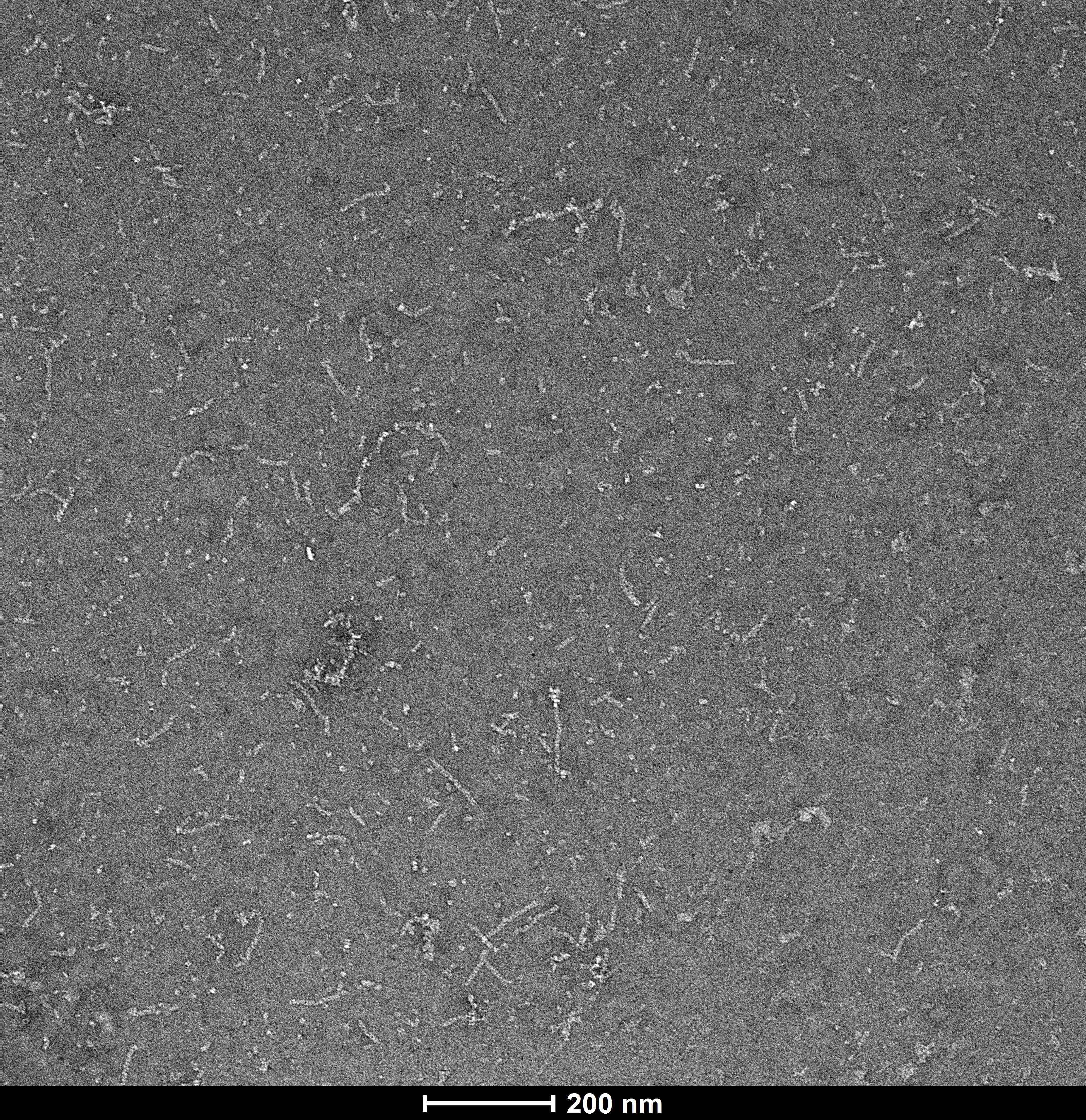

Supplement: Supplementary file 12 — The nsEM images. [file 41557_2023_1314_MOESM12_ESM.zip › ExtendedDataFigure6/WB13_5P_L41_22C_T0_1_25_36k.jpg]

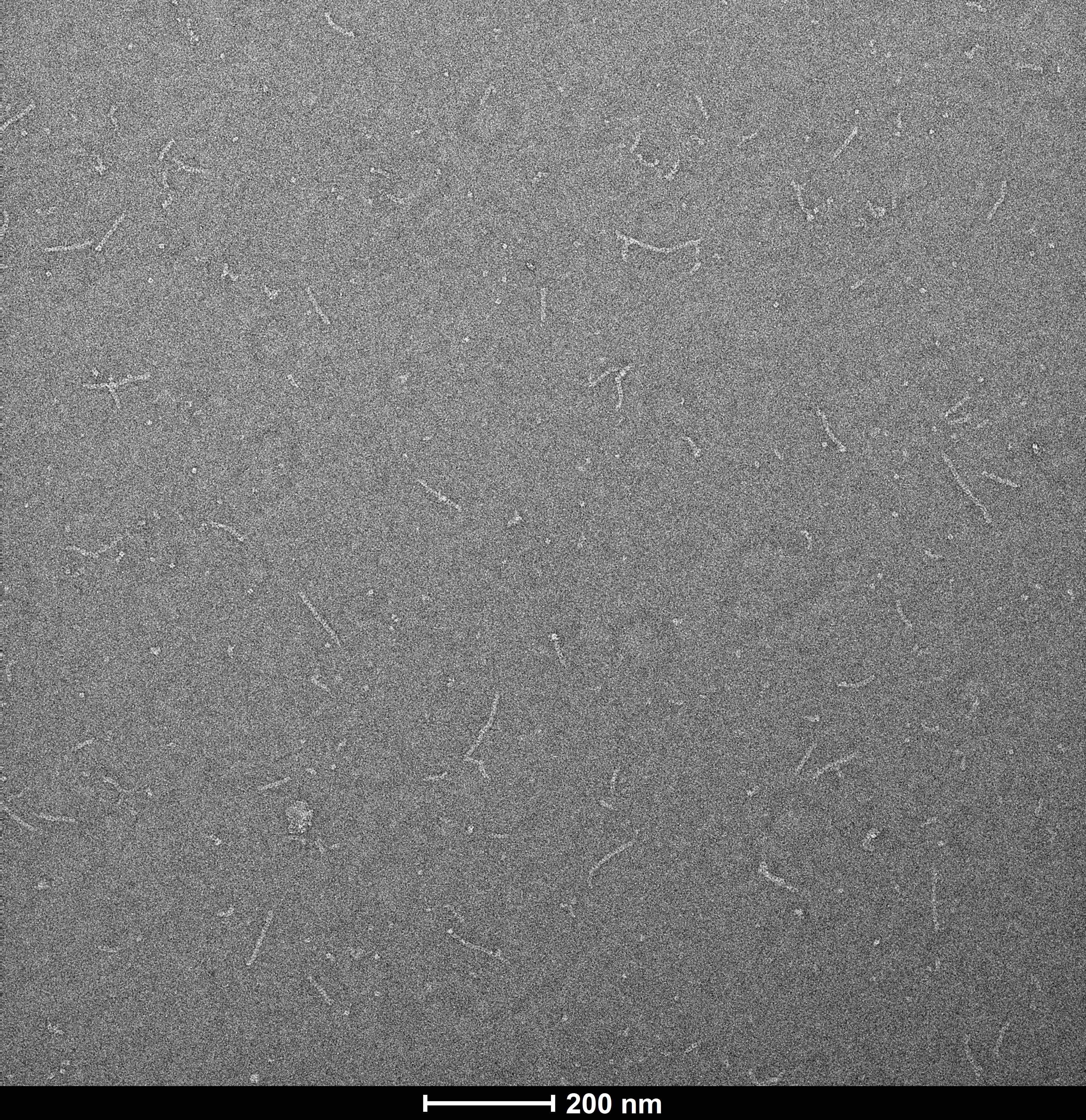

Supplement: Supplementary file 12 — The nsEM images. [file 41557_2023_1314_MOESM12_ESM.zip › ExtendedDataFigure6/WB13_8Q_L41_4C_T24_1_25_36k.jpg]

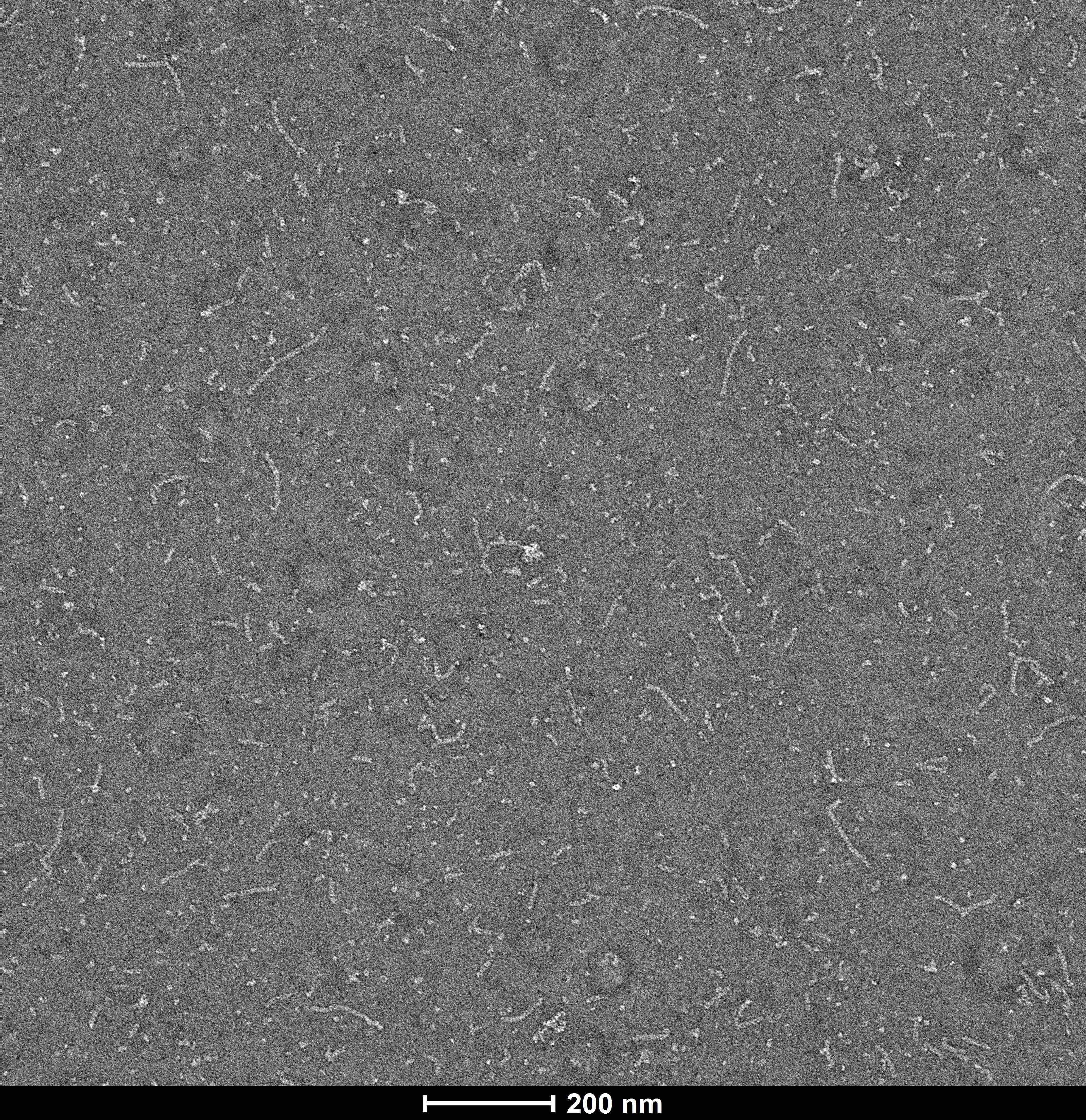

Supplement: Supplementary file 12 — The nsEM images. [file 41557_2023_1314_MOESM12_ESM.zip › ExtendedDataFigure6/WB13_5P_L41_4C_T0_1_25_36k.jpg]

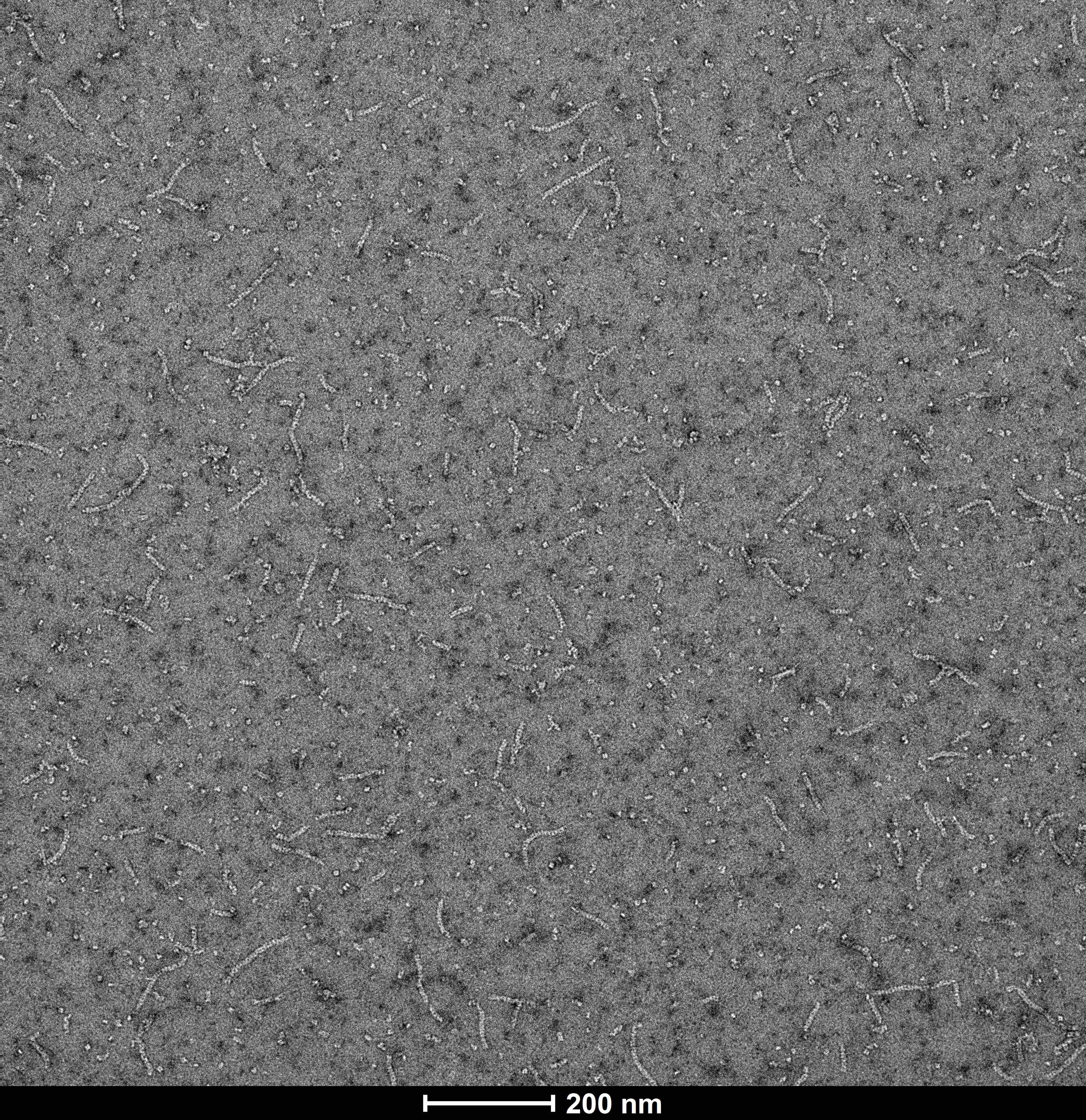

Supplement: Supplementary file 12 — The nsEM images. [file 41557_2023_1314_MOESM12_ESM.zip › ExtendedDataFigure6/WB13_9P_L41_37C_T24_1_25_36k.jpg]

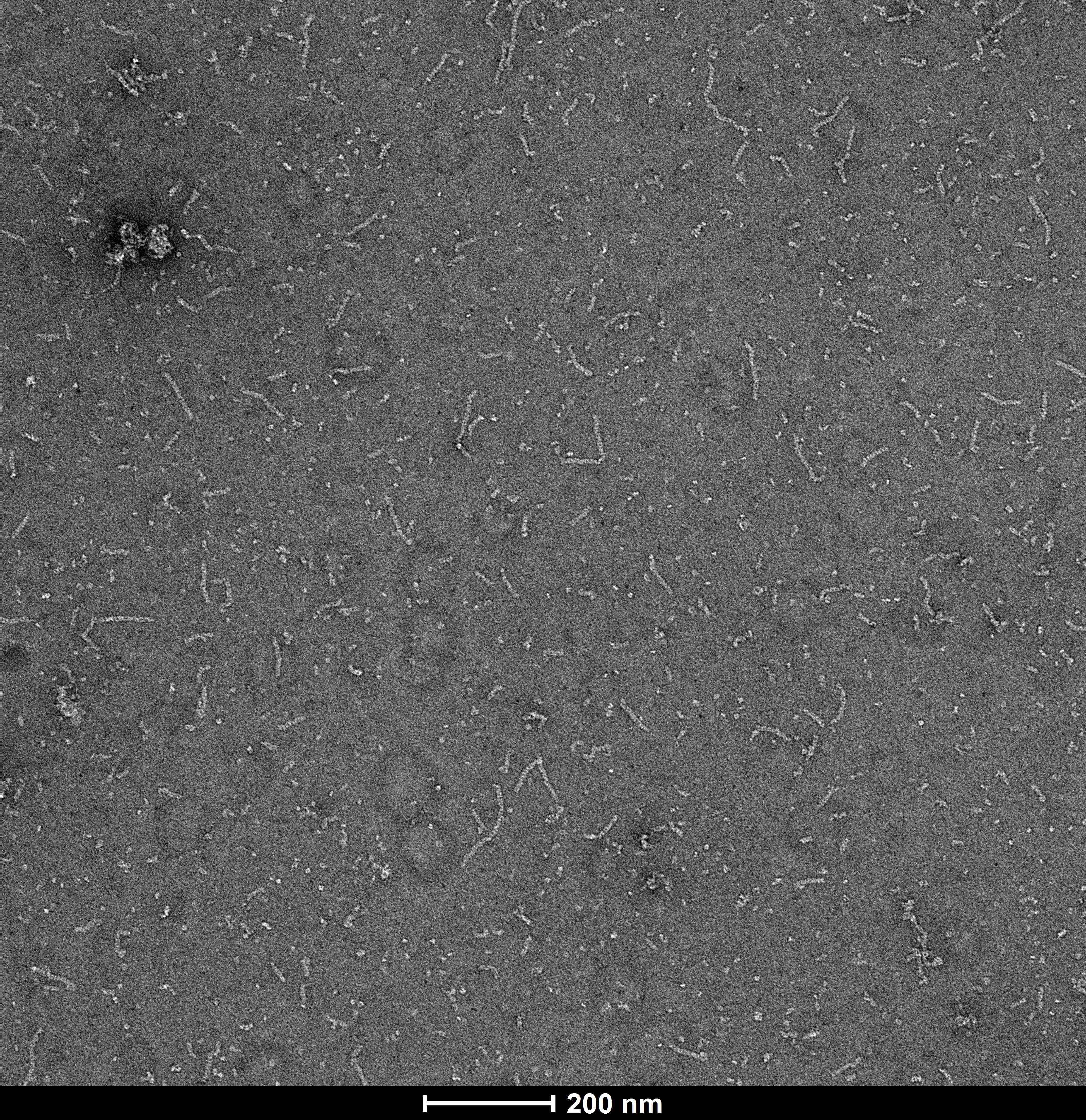

Supplement: Supplementary file 12 — The nsEM images. [file 41557_2023_1314_MOESM12_ESM.zip › ExtendedDataFigure6/WB13_5P_L41_37C_T0_1_25_36k.jpg]

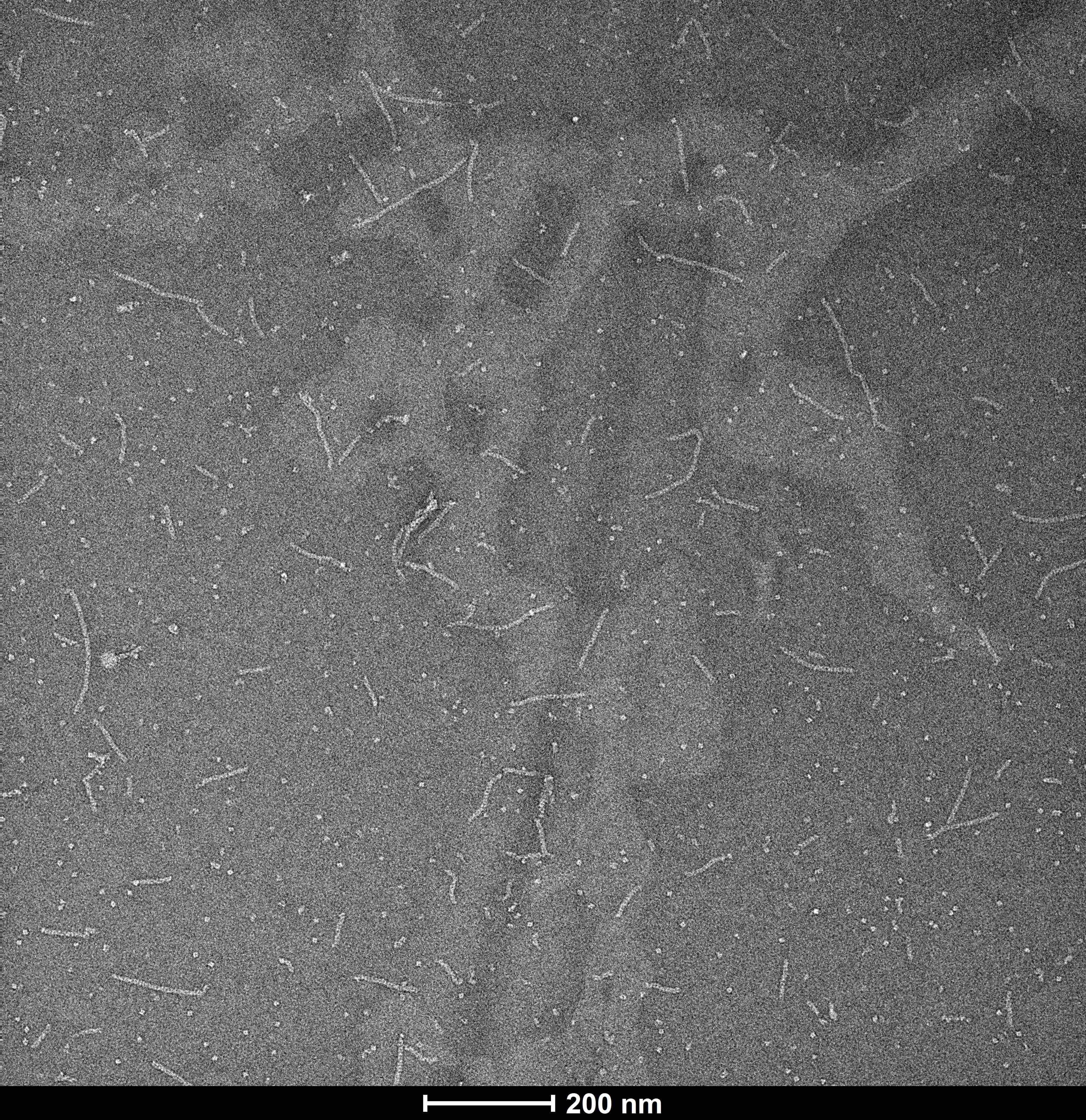

Supplement: Supplementary file 12 — The nsEM images. [file 41557_2023_1314_MOESM12_ESM.zip › ExtendedDataFigure6/WB14_1I_L41_37C_T7d_1_25_36k.jpg]

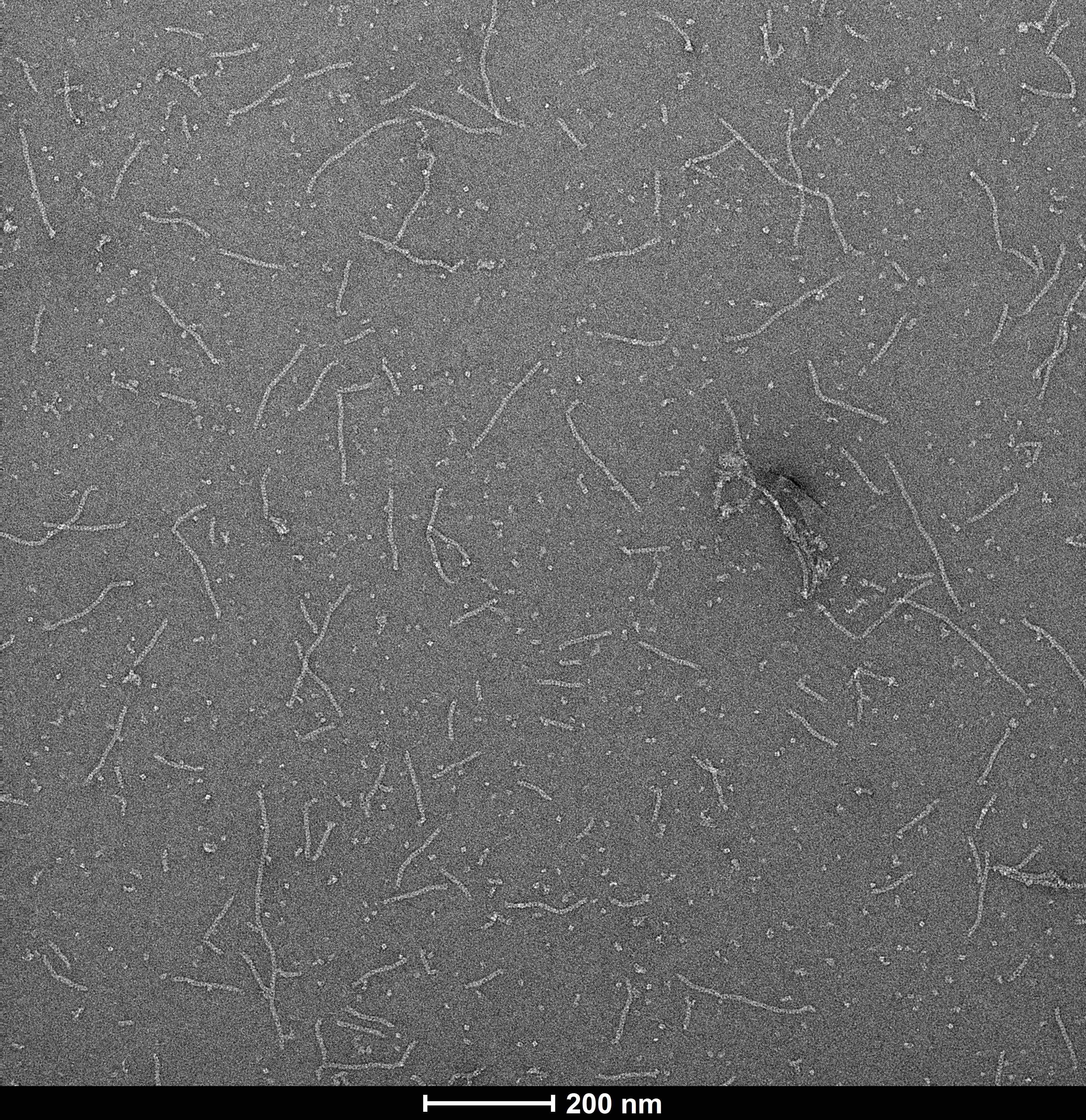

Supplement: Supplementary file 12 — The nsEM images. [file 41557_2023_1314_MOESM12_ESM.zip › ExtendedDataFigure6/WB14_1A_L41_4C_T7d_1_25_36k.jpg]

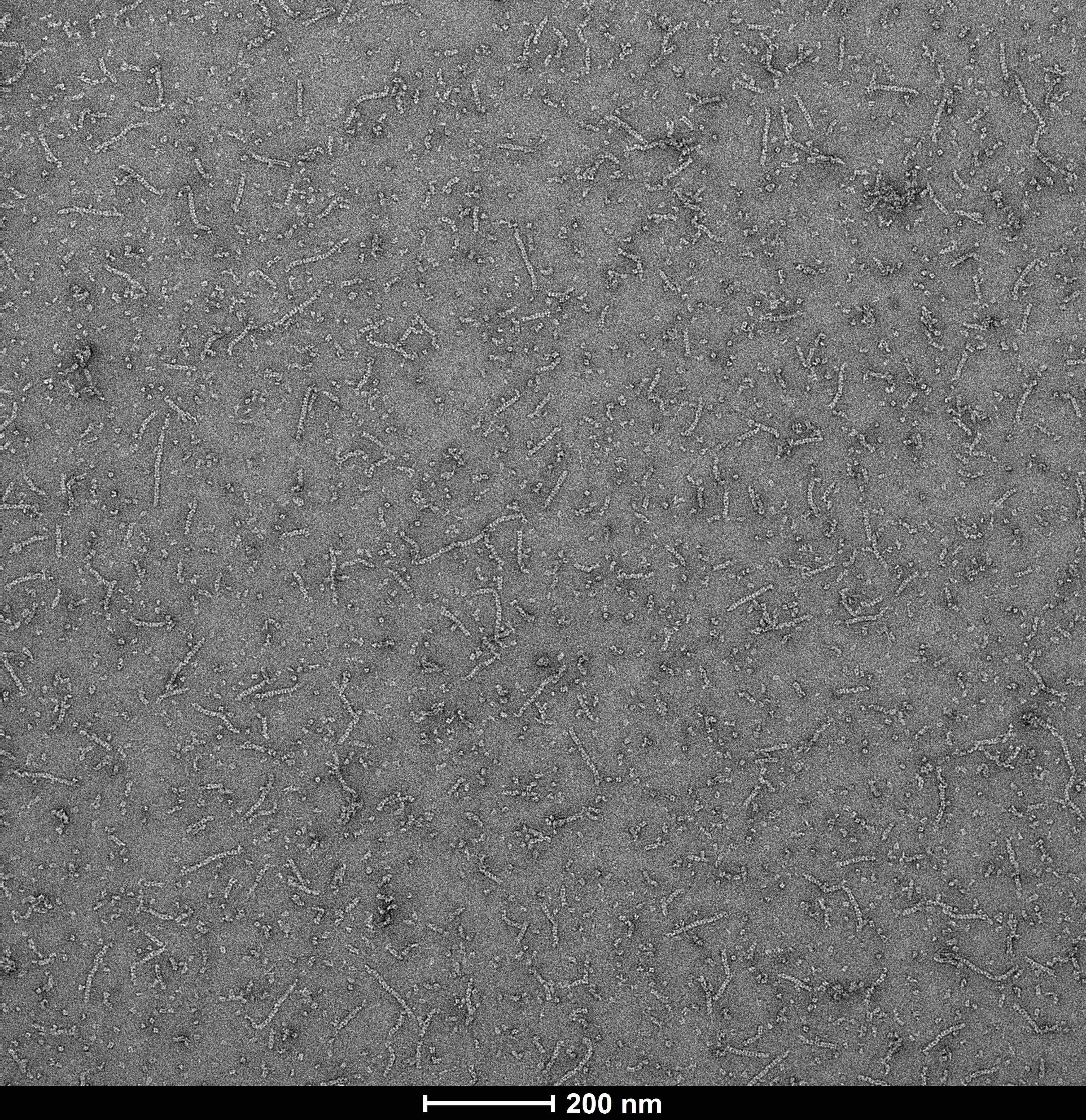

Supplement: Supplementary file 12 — The nsEM images. [file 41557_2023_1314_MOESM12_ESM.zip › ExtendedDataFigure6/WB13_8U_L41_22C_T24_1_25_36k.jpg]
